# Geographical mapping and temporal trends of *Acinetobacter baumannii* carbapenem resistance: A comprehensive meta-analysis

**DOI:** 10.1371/journal.pone.0311124

**Published:** 2024-12-16

**Authors:** Masoumeh Beig, Elnaz Parvizi, Tahereh Navidifar, Narjes Bostanghadiri, Maryam Mofid, Narges Golab, Mohammad Sholeh

**Affiliations:** 1 Department of Bacteriology, Pasteur Institute of Iran, Tehran, Iran; 2 Department of Microbiology, Science and Research Branch, Islamic Azad University, Fars, Iran; 3 Shoushtar Faculty of Medical Sciences, Department of Basic Sciences, Shoushtar, Iran; 4 Department of Microbiology, School of Medicine, Iran University of Medical Sciences, Tehran, Iran; 5 School of Medicine, Hamadan University of Medical Science, Hamadan, Iran; 6 Department of Microbiology, School of Medicine, Tehran University of Medical Sciences, Tehran, Iran; 7 Student Research Committee, Pasteur Institute of Iran, Tehran, Iran; Mahidol University Faculty of Pharmacy, THAILAND

## Abstract

**Background:**

Carbapenem-resistant *Acinetobacter baumannii* (CRAB) is of critical concern in healthcare settings, leading to limited treatment options. In this study, we conducted a comprehensive meta-analysis to assess the prevalence of CRAB by examining temporal, geographic, and bias-related variations.

**Methods:**

We systematically searched prominent databases, including Scopus, PubMed, Web of Science, and EMBASE. Quality assessment was performed using the JBI checklist. Subgroup analyses were performed based on the COVID-19 timeframes, years, countries, continents, and bias levels, antimicrobial susceptivity test method and guidelines.

**Results:**

Our comprehensive meta-analysis, which included 795 studies across 80 countries from 1995 to 2023, revealed a surge in carbapenem resistance among *A*. *baumannii*, imipenem (76.1%), meropenem (73.5%), doripenem (73.0%), ertapenem (83.7%), and carbapenems (74.3%). Temporally, 2020–2023 witnessed significant peaks, particularly in carbapenems (81.0%) and meropenem (80.7%), as confirmed by meta-regression, indicating a steady upward trend.

**Conclusion:**

This meta-analysis revealed an alarmingly high resistance rate to CRAB as a global challenge, emphasizing the urgent need for tailored interventions. Transparency, standardized methodologies, and collaboration are crucial for the accurate assessment and maintenance of carbapenem efficacy.

## 1. Introduction

The increasing prevalence of antibiotic resistance in *Acinetobacter baumannii* presents a formidable obstacle in healthcare settings, as this pathogen increasingly demonstrates resistance to a broad spectrum of antibiotics traditionally used to combat its infections [1]. A critical study in 2008 revealed that certain strains of *A*. *baumannii* develop resistance to all major classes of antibiotics, including β-lactams, aminoglycosides, and fluoroquinolones [2].

Carbapenems are pivotal in the treatment of *A*. *baumannii* infections, with studies highlighting them, along with aminoglycosides and fluoroquinolones, as preferred antibiotics [3]. Specifically, carbapenems like imipenem are valued as a last resort for cases resistant to multiple drugs due to their comprehensive antibacterial activity and effectiveness against *A*. *baumannii* [4,5].

Further evidence supporting the critical role of carbapenems, alongside other antibiotics such as tigecycline, colistin, and sulbactam, in treating infections by multidrug-resistant *A*. *baumannii* strains has been documented [6–8].

The increasing prevalence of CRAB is a significant and escalating global health issue characterized by varying and often rising resistance rates across different regions [9]. Notably, resistance rates in Latin America have been reported to range from 50% to 75% [10], whereas in China there has been a notable increase from 18% in 2012 to 60% in 2019 [11]. An overview of East Africa revealed a 64.8% prevalence of CRAB, reflecting the global spread of this resistance [12]. A comprehensive meta-analysis confirmed the global extent of this challenge, with carbapenem resistance rates exceeding 40% in *A*. *baumannii*, as identified by the World Health Organization (WHO) [13]. Further detailed studies showed that 71.4% of *A*. *baumannii* isolates in China were resistant in 2016 [14], and in the United States, 33% of strains from medical centers exhibited resistance [15], illustrating the widespread nature of carbapenem resistance.

Studies have revealed alarmingly high resistance rates across different regions, highlighting the urgent necessity for improved antimicrobial stewardship and infection control practices. The spread of specific clones, such as ST2, adds complexity to the resistance mechanisms, making tailored intervention strategies essential [16]. Moreover, the gap in updated global prevalence data, especially post-COVID-19, underscores the potential exacerbation of antimicrobial resistance challenges [17,18]. Continuous and comprehensive research into CRAB is crucial for developing effective treatment modalities and preventive measures to control this highly resistant pathogen.

This study aimed to shed light on the global prevalence of CRAB, with a focus on updating the data to reflect the current situation. The secondary objective was to dissect the heterogeneity of the CRAB prevalence through comprehensive subgroup analyses. These analyses cover geographical variations at both country and continent levels to highlight regional disparities. Temporal subgroup analysis was also employed to track the changes in the resistance patterns over time. Importantly, this study sought to fill a critical knowledge gap by investigating the potential impact of the COVID-19 pandemic on AMR through subgroup analyses and meta-regression, utilizing the largest possible sample size. This approach aims to provide a holistic understanding of CRAB prevalence and the evolving AMR landscape in the context of the recent global health challenges.

## 2. Methods

Our study rigorously adhered to the Preferred Reporting Items for Systematic Reviews and Meta-Analyses (PRISMA) guidelines to ensure a robust meta-analytical synthesis of findings on CRAB (CRAB). This adherence was underscored by our registration with the Prospero Registry (CRD42023482285), affirming our commitment to transparency and methodological integrity.

### 2.1. Eligibility criteria

We meticulously defined the eligibility criteria to include studies that offered insights into CRAB, detailed resistance proportions, and specified sample sizes and were published in full-text English. Studies not in English, reviews, case reports, single-arm studies, and those focusing solely on pharmacokinetics were excluded, ensuring a focused and relevant dataset for analysis.

### 2.2. Information sources

A comprehensive search was performed across the Scopus, PubMed, Web of Science, and EMBASE databases until April 18, 2023. These databases were chosen for their extensive coverage of the biomedical literature, which enhanced the breadth of our systematic review.

### 2.3. Search strategy

We developed a nuanced search strategy by adjusting the syntax for each database to maximize the retrieval of pertinent studies using *A*. *baommanni* and carbapenem resistance-related keywords. The detailed search strings and adaptations are documented in the S1 File, providing transparency and reproducibility.

### 2.4. Selection process

Upon retrieval, studies were collated using EndNote (version 20) for efficient duplication removal. Two authors (E.P. and T.N.) independently screened the studies, with N.B. resolving any discrepancies and upholding an unbiased selection principle.

### 2.5. Data collection process

Data extraction was conducted independently by E.P. and T.N., with M.Sh. serving as the arbiter for any dispute, ensuring accuracy and consistency in the data collated for analysis.

### 2.6. Data items and study risk of bias assessment

The extracted data encompassed critical information including the first author(s), publication year, study location, diagnostic methodology, sample source, number of positive tests, and total sample size. The risk of bias was appraised using the Joanna Briggs Institute (JBI) tool [18], with two independent evaluations conducted (N.B. and E.P.) and discrepancies adjudicated by M.Sh. This structured assessment allowed us to gauge study quality effectively and classify the studies into risk categories based on their scores.

### 2.7. Effect measures and synthesis methods

Our analytical framework centered on proportions as effect measures, employing a random-effects model and the DerSimonian-Laird estimator to account for study heterogeneity. This was complemented by a thorough exploration of heterogeneity using the Q-test and the I^2^ statistic. Meta-regression and subgroup analyses were initiated upon detecting significant heterogeneity, enriching our understanding of CRAB prevalence variations.

Outlier identification was performed using studentized residuals and Cook’s distances using R (version 4.2.1) [19], the metafor package (version 3.8.1) [20], tidyverse [21] for statistical analyses. This meticulous approach allowed us to maintain analytical rigor and reliability.

To synthesize our findings, we undertook a dual-focused analysis of specific carbapenem antibiotics and a generalized "carbapenem" category to accommodate the diversity in reporting across studies. This nuanced approach allowed us to capture both detailed and broad resistance patterns, thereby offering a comprehensive insight into carbapenem resistance.

### 2.8. Reporting bias assessment and certainty assessment

We applied the rank correlation test and Egger’s regression tests to assess funnel plot asymmetry, furthering our commitment to identify and address reporting bias. Fail-Safe N and Trim-and-Fill methods were employed to ensure the robustness of our conclusions, with the standard error of the observed outcomes guiding these analyses.

## 3. Results

### 3.1. Study selection

**Fig 1:**
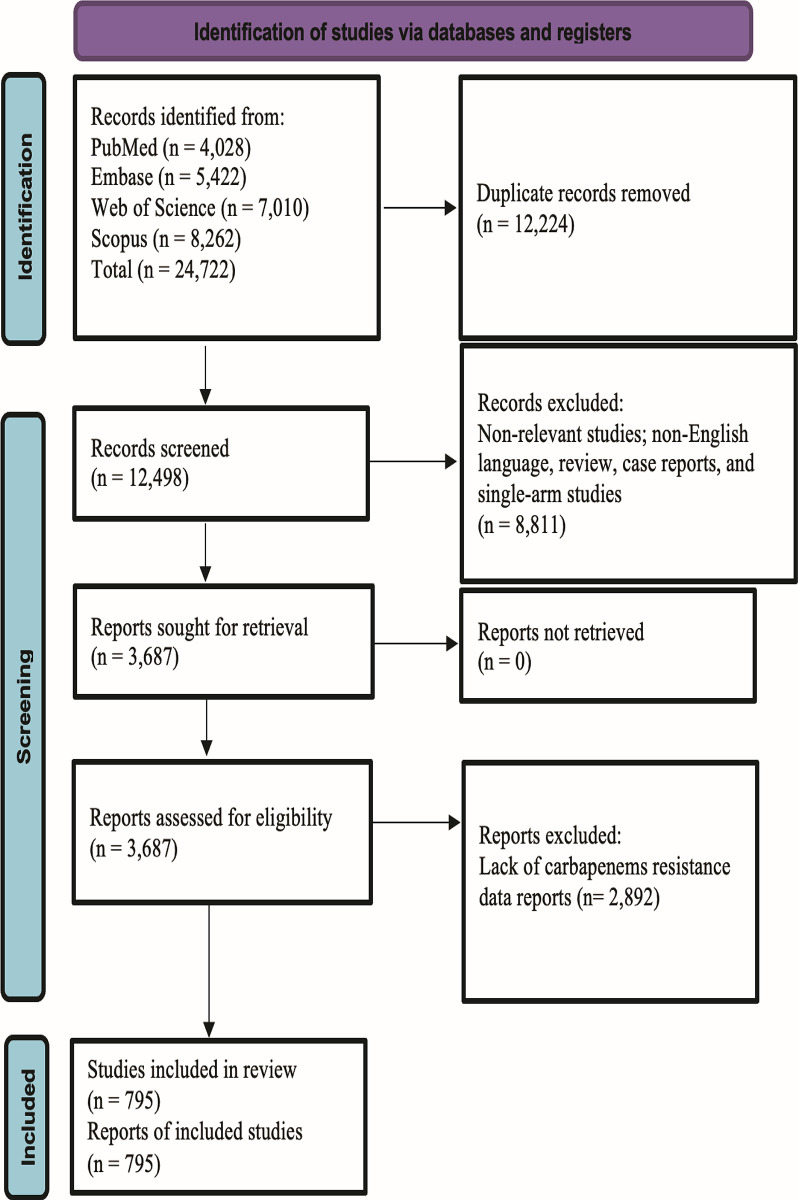
PRISMA flow chart summarizes the article selection procedure.

Our systematic search retrieved 24,722 records. These records were efficiently organized using EndNote version 20, which is a reference management software package. Through a thorough deduplication process, we identified and removed 12,224 duplicate entries, leaving 12,498 articles for the preliminary evaluation. The assessment process began with a review of the titles and abstracts, leading to the selection of 795 studies that met our stringent eligibility criteria [22–816]. These studies were included in the extensive systematic review and meta-analysis. The selection and screening processes, including each phase of article reduction and selection, were successfully visualized in the PRISMA flowchart (Fig 1), which provided a clear overview of our meticulous methodology.

### 3.2. Study characteristics

This study’s dataset is remarkable for its breadth, comprising reports from 80 countries across various continents, showing wide geographical diversity. Our study offers an in-depth look at the evolution and current state of knowledge from a global perspective from 1995 to 2023. For a detailed overview of the included articles and the data extracted for this meta-analysis, please refer to S1 Table in S2 File.

### 3.3. Risk of bias in studies

In our risk of bias assessment, a significant proportion of the studies (83.21%) exhibited a low risk of bias, indicating strong and reliable evidence (S2 Table in S2 File). A negligible number (0.50%) were assessed as high risk, indicating potential validity concerns (S1 Fig in S2 File). Studies with concerns accounted for 16.29% of the total, suggesting moderate issues affecting their interpretation. This distribution highlights the overall quality and credibility of the studies included in this meta-analysis.

### 3.4. Results of syntheses

**Fig 2:**
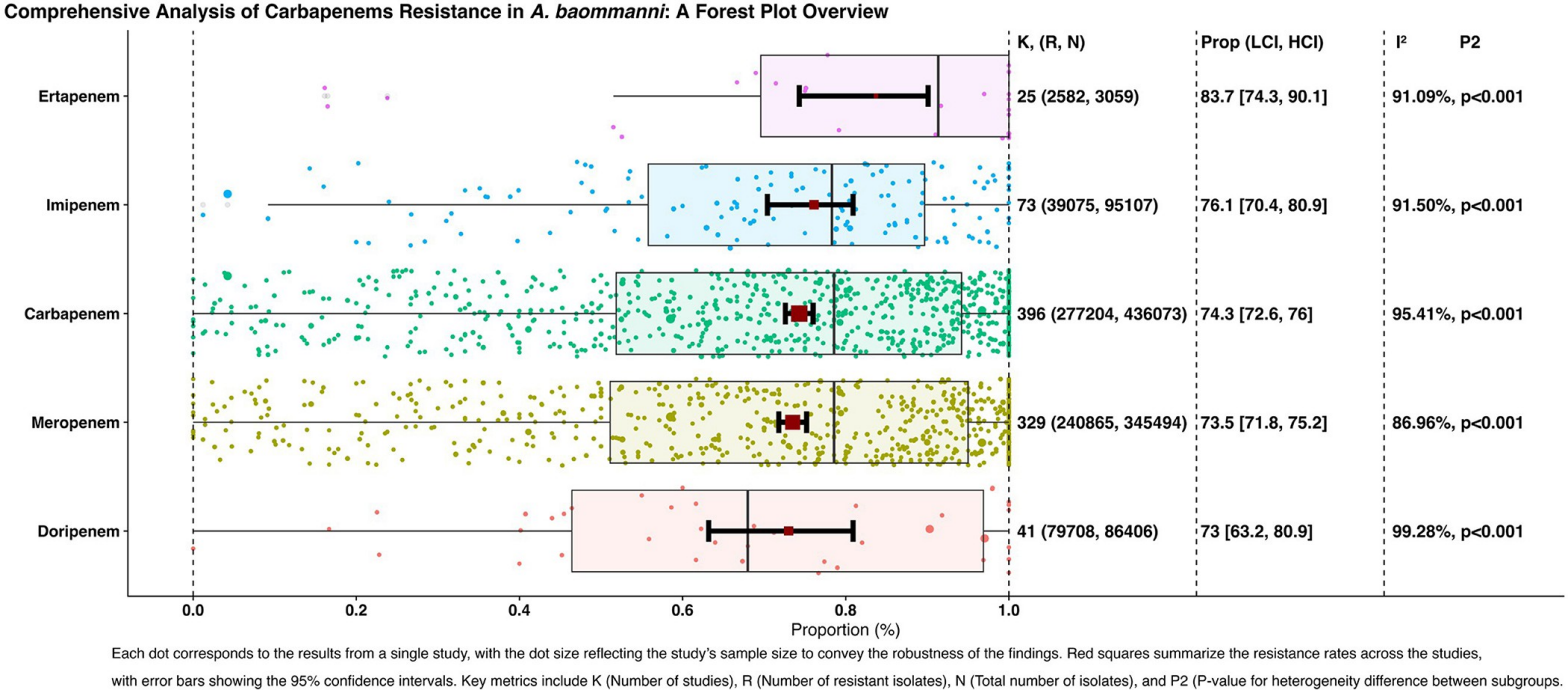
Worldwide weighted pooled proportion of CRAB isolates.

In a global analysis, the prevalence of CRAB isolates was found to be significantly higher (76.1% for imipenem, 73.5% for meropenem, 73% for doripenem, and 83.7% for ertapenem). These findings illustrate the extensive patterns of resistance to different carbapenem antibiotics, for a visual interpretation of these rates and a comparison of the observed outcomes with estimates from the random effects model (Fig 2), which features a detailed forest plot.

#### 3.4.1. Prevalence of imipenem resistance

Our meta-analysis included 95,107 isolates from 146 studies that evaluated imipenem resistance. The pooled average resistance proportion, derived from a random-effects model, was 76.1% (95% CI, 70.4% to 80.9%). Heterogeneity testing revealed considerable variation among the study outcomes (Q(145) = 28,309.594, I^2^ = 99.49%, p < 0.001). Implementation of the fill-and-trim method adjusted the estimated resistance proportion to 65.9% (95% CI, 59.7% to 71.6%). An analysis of studentized residuals identified a potential outlier in the one study which when excluded, maintained the adjusted proportion at 65.9%. Cook’s distance further pinpointed two studies as being overly influential. The exclusion of outliers confirms the adjusted proportions. No evidence of funnel plot asymmetry was found (rank correlation, p = 0.561; regression test, p = 0.134).

#### 3.4.2. Prevalence of meropenem resistance

Analysis of 345,494 isolates across 659 studies was conducted to assess meropenem resistance, with an estimated average proportion of 73.5% (95% CI, 71.8% to 75.2%). High heterogeneity was observed (Q(658) = 51,448.469, I^2^ = 98.72%, p < 0.001). Following fill-and-trim adjustment, the resistance proportion was recalibrated to 65.2% (95% CI, 63.1% to 67.2%). Several studies were identified as potential outliers, with values exceeding 3.957. The exclusion of these outliers results in a consistently recalibrated proportion. Notably, funnel plot tests suggested asymmetry (rank correlation, p < 0.001; regression test, p < 0.001).

#### 3.4.3. Prevalence of doripenem resistance

Doripenem resistance analysis included 86,406 isolates from 41 studies, yielding an average proportion of 73.0% (95% CI, 63.2% to 80.9%). Pronounced heterogeneity among the studies was noted (Q(40) = 5,548.703, I^2^ = 99.28%, p < 0.001), and the fill-and-trim method confirmed the initial proportion. No studies were identified as outliers and potential funnel plot asymmetry was detected (rank correlation, p < 0.001; regression test, p = 0.011).

#### 3.4.4. Prevalence of ertapenem resistance

Involving 3,059 isolates from 26 studies, the ertapenem resistance assessment produced an estimated average proportion of 83.7% (95% CI, 74.3% to 90.1%), with evident heterogeneity (Q(25) = 290.938, I^2^ = 91.41%, p < 0.001). After fill-and-trim adjustment, the resistance proportion was modified to 65.2% (95% CI, 51.4% to 76.9%). One study was flagged as a potential outlier, but its exclusion did not alter the adjusted proportion. The regression test indicated funnel plot asymmetry (p < 0.001) unlike the rank correlation test (p = 0.764).

#### 3.4.5. Prevalence of carbapenem resistance

This extensive analysis, incorporating 436,073 isolates from 793 studies, established an average carbapenem resistance rate of 74.3% (95% CI, 72.6% to 76.0%). The heterogeneity test confirmed substantial variability (Q(792) = 80,821.664, I^2^ = 99.02%, p < 0.001). The fill-and-trim method subsequently adjusted the resistance proportion to 65.5% (95% CI, 63.5% to 67.4%). The identification of several potential outliers and influential studies has led to a consistently adjusted resistance rate upon removal. Evidence of funnel plot asymmetry was observed (rank correlation, p = 0.005; regression test, p < 0.001).

### 3.5. Subgroup analysis

The subgrouping analysis results were summarized in S3 Table in S2 File.

#### 3.5.1. Geographical subgroup analysis

**Fig 3:**
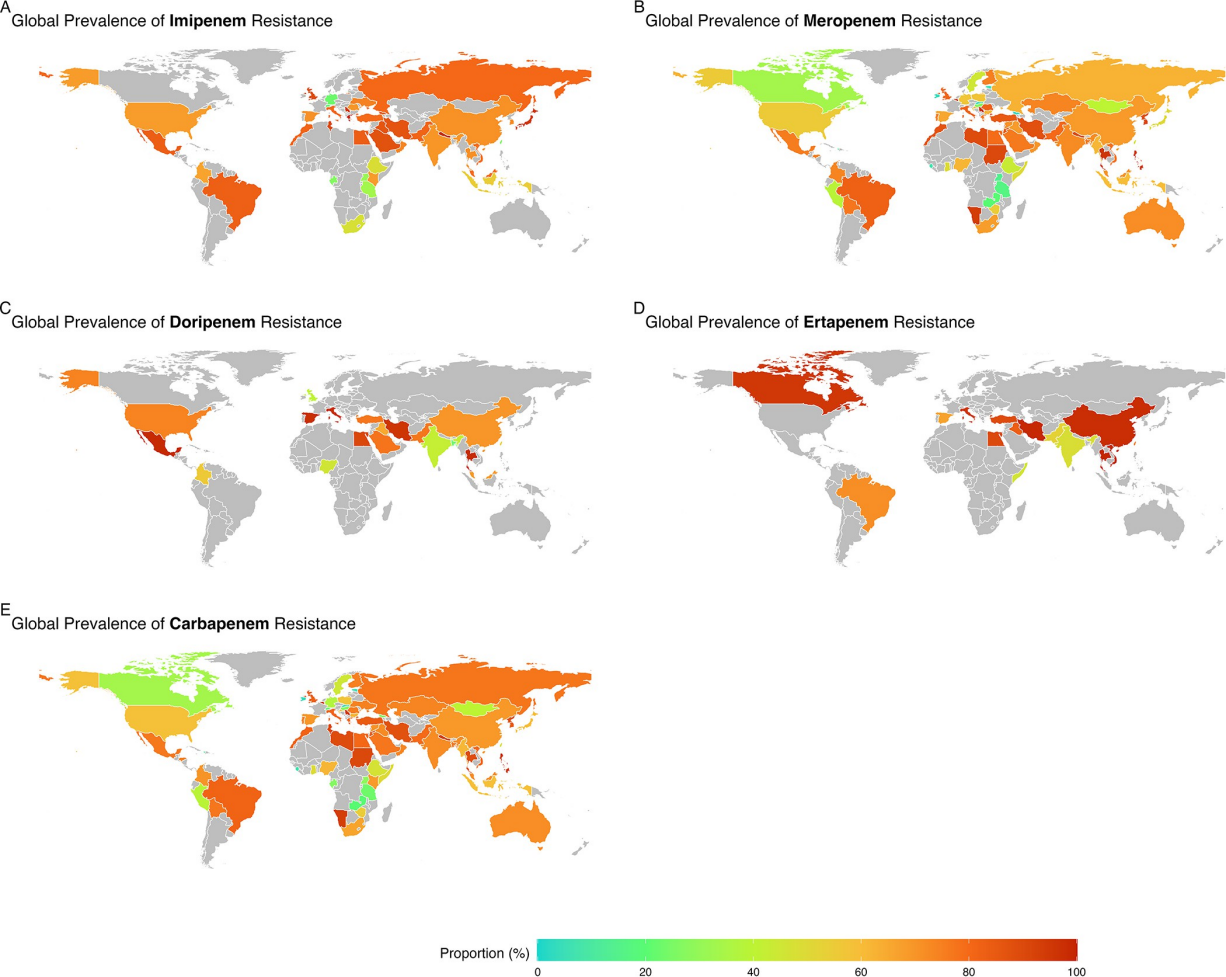
Worldwide Map for Prevalence of CRAB; A: Imipenem, B: Meropenem, C: Doripenem, D: Ertapenem, and E: Carbapenem. Global map visualization created using OpenStreetMap data, available under the Open Database License (ODbL). Map data © OpenStreetMap contributors, licensed under ODbL.

In our geographical subgroup analysis, we identified substantial disparities in the prevalence of antibiotic resistance, specifically carbapenem and meropenem, across an array of countries (Fig 3). Ireland notably presented a minimal occurrence of carbapenem resistance at a mere 4%, in stark contrast to the Philippines, where a staggering 96.1% resistance rate was observed. Similarly, Georgia demonstrated the lowest prevalence of meropenem resistance at only 2.3%, whereas Serbia faced a significantly higher challenge, with a resistance rate of 96.5%. These observations highlight the extensive global variation in antibiotic resistance, emphasizing the urgent need for region-specific approaches to effectively manage and curb bacterial resistance.

**Fig 4:**
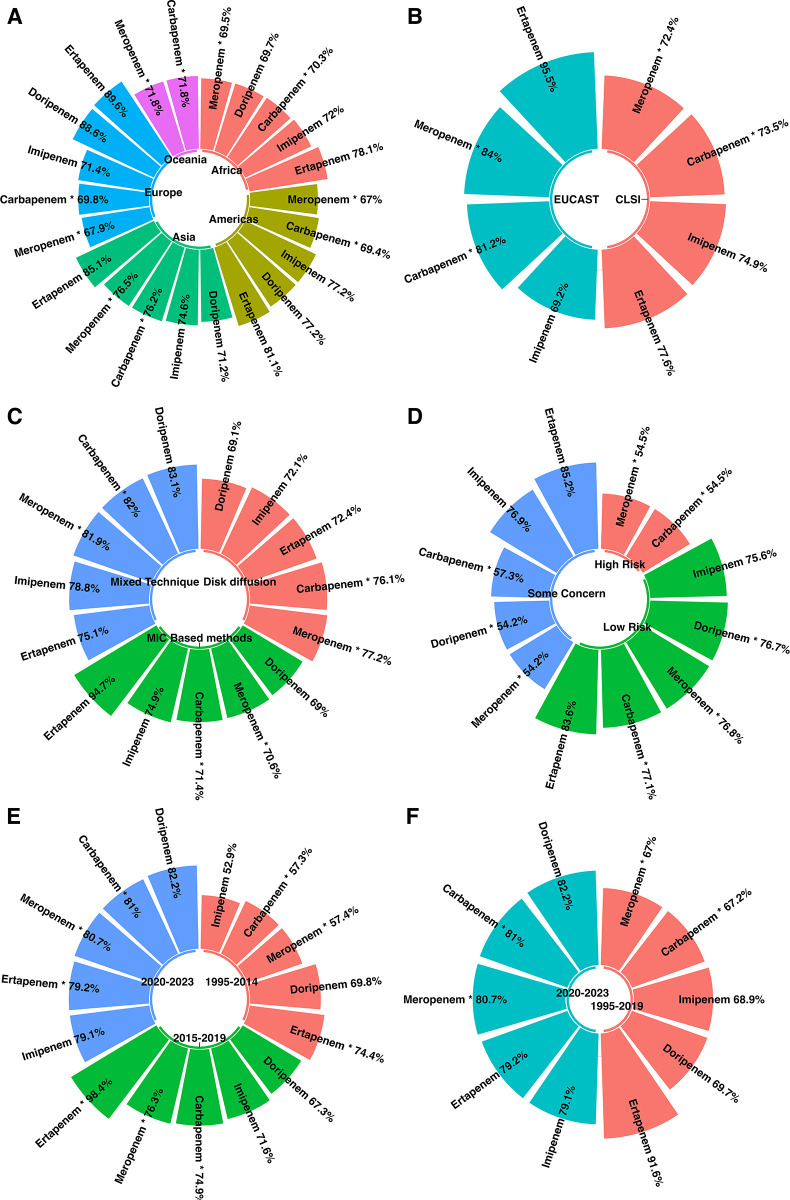
Subgroup analysis results are illustrated as follows: **A:** Prevalence of CRAB isolates across continents; **B:** Compression prevalence of CRAB isolates according to different AST guidelines; **C:** Compression prevalence of CRAB isolates using various AST techniques; **D:** Compression prevalence of CRAB isolates according to the risk of bias in the JBI checklist results. **E**: Compression prevalence of CRAB isolates between year subgroups. **F**: Compression prevalence of CRAB isolates before and after the COVID-19 pandemic (1995–2019, and 2020–2023).

Expanding our analysis to a continental perspective, we encountered pronounced variations in carbapenem and meropenem resistance rates across continents (Fig 4A). The Americas had the lowest carbapenem resistance rate (69.4%), whereas Asia had the highest rate at 76.2%. A similar pattern was observed for meropenem resistance, with the Americas displaying the lowest rate at 67%, in contrast to Asia, which recorded the highest rate of resistance at 76.5%. These findings underline the geographical influence of antibiotic resistance trends and reinforce the need for tailored public health strategies to address the specific challenges faced by each region in the battle against antibiotic resistance.

#### 3.5.2. Analysis by guideline standards

Resistance rates varied significantly across carbapenem guidelines. The Clinical and Laboratory Standards Institute (CLSI) guidelines reported a lower resistance prevalence of 73.5%, in contrast to the European Committee on Antimicrobial Susceptibility Testing (EUCAST) guidelines, which reported a higher prevalence of 81.2%. Similarly, meropenem resistance was less prevalent under the CLSI guidelines (72.4%), whereas the EUCAST guidelines indicated a more substantial resistance rate of 84% (Fig 4B).

#### 3.5.3. Analysis by Antimicrobial Susceptibility Testing (AST) methods

Disparities were evident when examining the resistance using the AST method. For carbapenem, Minimum Inhibitory Concentration (MIC)- based methods demonstrated the lowest resistance rate of 71.4%, whereas the Mixed Technique yielded a higher resistance rate of 82%. For meropenem, MIC-based methods also reported a lower resistance rate of 70.6% compared to the mixed Technique, which indicated a resistance rate of 81.9% (Fig 4C).

#### 3.5.4. Subgroup analysis stratified by quality assessments

In our subgroup analysis, we identified a significant variation in the prevalence of antibiotic resistance to both carbapenems and meropenem, underscoring differing levels of bias (Fig 4D). Regarding carbapenems, instances associated with a high risk of bias demonstrated the lowest recorded incidence of resistance (54.5%). Conversely, those with a low risk of bias exhibited the highest incidence of resistance (77.1%). A similar trend emerged for meropenem, where instances with high-risk bias showed a prevalence rate of 54.5%, whereas those with low-risk bias reported the highest resistance incidence at 76.8%.

#### 3.5.5. Temporal analysis

Our temporal analysis revealed significant changes in antibiotic resistance, especially for carbapenems and meropenem, highlighting concerns over various periods, particularly about the COVID-19 pandemic. Initially, we observed the lowest rates of carbapenem and meropenem resistance between 1995 and 2014, at 57.3% and 57.4%, respectively. This contrasts starkly with the data from 2020 to 2023, where resistance rates surged to 81.0% for carbapenem and 80.7% for meropenem, illustrating a dramatic escalation of resistance during and after the COVID-19 pandemic (Fig 4E).

**Fig 5:**
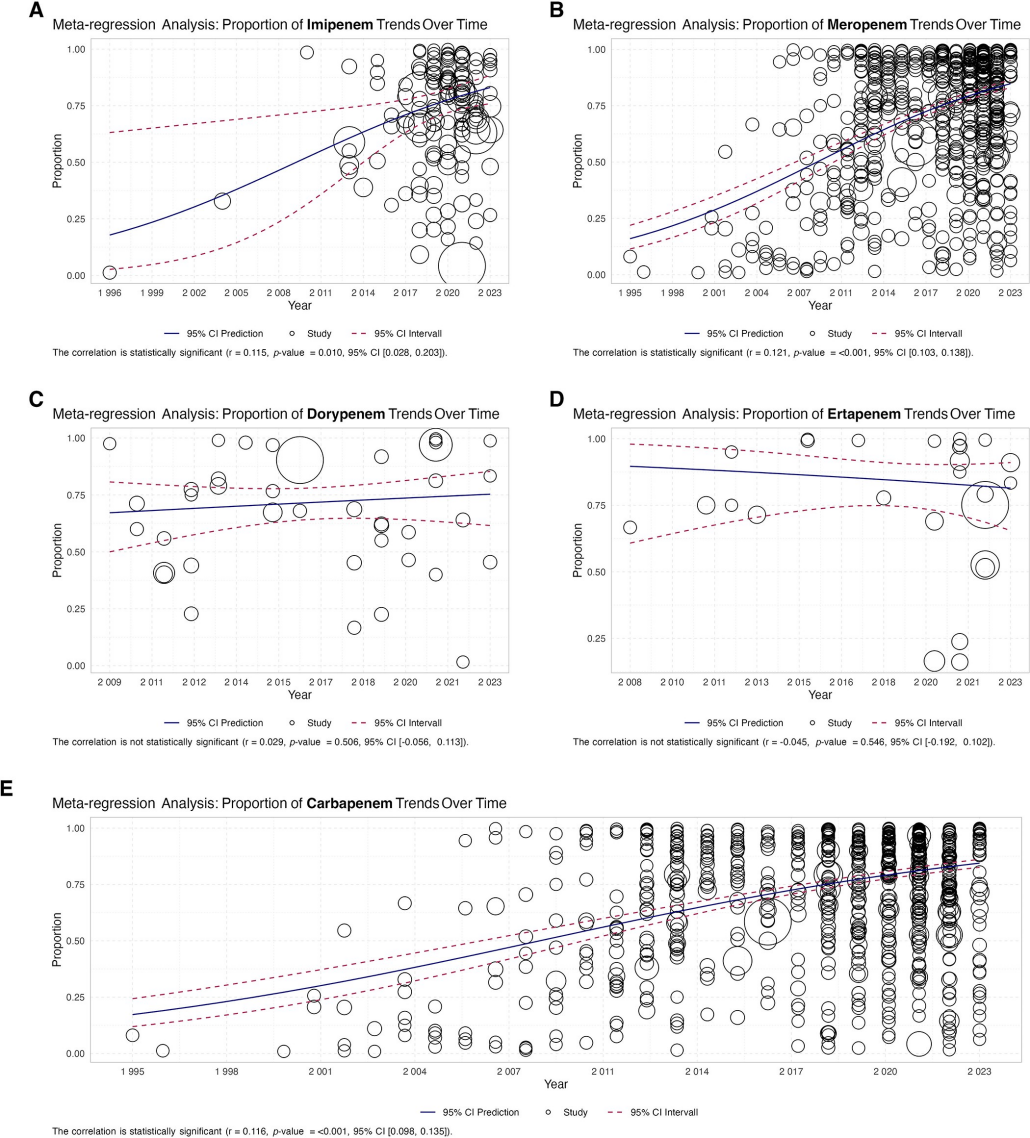
Meta regression of carbapenem resistance *A*. *baumannii* isolates during years; **A:** Imipenem, **B:** meropenem, **C:** Doripenem, **D:** Ertapenem **E:** carbapenem.

Expanding our examination to cover the years between 1995 and 2023, we found that resistance to carbapenems has increased from a lower rate of 67.2% in the period up to 2019 to 81.0% from 2020–2023. Meropenem resistance increased from 67.0% to 80.7% during the same period (Fig 4F). This upward trend was further supported by our meta-regression analyses, which assessed the temporal dynamics of resistance to various carbapenem antibiotics, including imipenem, meropenem, doripenem, and ertapenem, as well as the general category of carbapenems (Fig 5).

The analysis revealed a significant increase in resistance to imipenem (r = 0.115, p = 0.010) and meropenem (r = 0.121, p < 0.001) over time, indicating an alarming trend. In contrast, the resistance rates for doripenem and ertapenem remained stable or fluctuated without significant changes over the analyzed period (doripenem: r = 0.0298, p = 0.506; ertapenem: r = -0.045, p = 0.546). Overall, meta-regression analysis for carbapenems showed a significant positive correlation (r = 0.116, p < 0.001, 95% CI: 0.098–0.135), confirming a continuous increase in resistance rates throughout the study duration. These findings highlight the need for heightened vigilance and innovative strategies to combat the escalating threat of antibiotic resistance.

#### 3.5.6. Reporting biases

Table 1 presents a detailed assessment of publication bias in our meta-analysis through the application of the Egger and Begg tests across a spectrum of antibiotics and S2 Fig in S2 File illustrates the funnel plots. The Egger test revealed a significant publication bias for all studied antibiotics (imipenem, meropenem, doripenem, ertapenem, and carbapenem), suggesting the influence of unpublished studies or selective reporting on the results. The Begg test further supported these findings, with a particular emphasis on the pronounced bias for doripenem (p < 0.001), indicating varying degrees of publication bias among the antibiotics examined. This thorough examination enhances the credibility of our findings by actively identifying and addressing potential bias.

**Table 1:** Evaluation of publication bias in meta-analysis.

**Antibiotic**	**Egger Test**	**Begg test**	**Fail and safe**	**Trim and Fill**
**Imipenem**	< 0.001	0.005	113391	0.659 (0.597, 0.716)
**Meropenem**	< 0.001	0.291	2040938	0.652 (0.632, 0.672)
**Doripenem**	0.011	< 0.001	40043	0.730 (0.632, 0.809)
**Ertapenem**	< 0.001	0.631	1581	0.652 (0.514, 0.769)
**Carbapenem**	< 0.001	0.916	3016703	0.655 (0.635, 0.674)

This table provides a comprehensive assessment of potential publication bias in the meta-analysis using a range of statistical techniques. Included are statistics generated from Egger’s method, Begg’s Method, the Fail-Safe N (NFS), and the Trim-and-Fill Method. These methods are applied to investigate the presence of bias and its impact on the meta-analysis results, ensuring the robustness and reliability of the findings.

Subsequent subgroup analysis aimed at dissecting the variance in antibiotic resistance prevalence revealed statistically significant differences based on the sample size (larger versus smaller than average). Specifically, for carbapenems, larger sample sizes were associated with a lower resistance rate (69.3%), whereas smaller sample sizes demonstrated a higher resistance rate (75.2%). This nuanced analysis sheds light on the impact of sample size on resistance rates, further refining our understanding of antibiotic-resistance dynamics.

#### 3.5.7. Certainty of evidence

The Fail-Safe N method indicated that a large number of additional studies with non-significant findings would be required to overturn the observed significant results in our meta-analysis: 113,391 for imipenem, 2,040,938 for meropenem, 40,043 for doripenem, 1,581 for ertapenem, and 3,016,703 for carbapenem. This suggests the robustness of our findings against the potential influence of unpublished data. Furthermore, the Trim-and-Fill method adjustments resulted in corrected resistance rates, aligning closely with the confidence intervals of 65.9% for imipenem, 65.2% for meropenem, 73% for doripenem, 65.2% for ertapenem, and 65.5% for carbapenem (Table 1). These adjusted estimates further validated the integrity and strength of our meta-analysis, demonstrating the comprehensive and careful application of statistical analyses to ensure the accuracy of our results.

## 4. Discussion

This comprehensive meta-analysis investigated the global prevalence of CRAB and its variation across different geographical regions and periods. These findings underscore the gravity of the CRAB challenge, which poses a significant threat to patient safety, healthcare resources, and public health worldwide [18]. Furthermore, a meta-analysis highlighted that carbapenem resistance rates above 40% have been observed globally in *A*. *baumannii*, as reported by the WHO [13]. A study in China in 2016 found that 71.4% of *A*. *baumannii* isolates were resistant to carbapenems [14]. Additionally, a study in the United States showed that 33% of *A*. *baumannii* strains from medical centers exhibit carbapenem resistance [15]. These findings highlight the widespread nature of carbapenem resistance in *A*. *baumannii* across different regions.

The current study’s robust methodology, involving a systematic search of multiple databases and rigorous eligibility criteria, yielded a substantial dataset encompassing 795 studies from 80 countries across five continents. This extensive geographical representation highlights the universal nature of the CRAB problem faced by global healthcare systems.

The impact of carbapenem resistance on clinical outcomes is profound, with mortality rates for CRAB infections as high as 76% [817]. The findings from the meta-analysis highlight the upward trend in carbapenem resistance among *A*. *baumannii* strains, particularly from 2021 to 2023, raising significant concerns for healthcare professionals worldwide [818]. This trend underscores the adaptability and resilience of *A*. *baumannii*, a pathogen known for its ability to acquire resistance mechanisms [819]. The differential significance of resistance increases among carbapenems (imipenem, meropenem, and doripenem) offers a nuanced view of the evolving battle between antimicrobial agents and bacterial pathogens.

The observation that only the increase in meropenem resistance rates was statistically significant, while those against imipenem and doripenem, did not suggest a complex interplay of factors influencing resistance development. The lack of significant data on imipenem resistance prior to 2011 could skew the resistance trend’s interpretation. This highlights the critical role of longitudinal surveillance and robust data collection in the understanding and response to AMR (AMR). Without comprehensive data spanning many years, it has become challenging to assess the magnitude and significance of resistance trends accurately [820]. However, the superior efficacy of meropenem over imipenem against *A*. *baumannii*, due to its greater stability against the beta-lactamase enzymes produced by the bacterium, has led to its increased use in treating infections caused by this pathogen. This enhanced stability not only expands the effectiveness of meropenem but also makes it the preferred option in scenarios involving CRAB strains. As a result, frequent use of meropenem exerts selective pressure on *A*. *baumannii*, fostering the development of strains resistant to this drug [821]. This situation exemplifies the complex relationship between antibiotic use and the evolution of bacterial resistance, highlighting how the widespread adoption of an antibiotic driven by its superior performance can inadvertently contribute to the rise in resistance, particularly when it becomes the go-to choice to combat challenging infections [822].

Despite the observed increase in the prevalence of CRAB during the COVID-19 pandemic, particularly in the context of the increased use of meropenem and carbapenem combinations (imipenem and meropenem), a subgroup analysis (before and after 2020) did not reveal a significant change in the trajectory of resistance development. Meta-regression analysis indicated that the slope of the increasing trend in resistance rates remained stable, suggesting that the pandemic did not significantly impact the overall rate of CRAB resistance escalation. Studies have also indicated a correlation between carbapenem consumption and resistance rates, emphasizing the need for prudent antibiotic use to combat resistance [823]. Moreover, combination therapies have shown promise in treating CRAB infections, with higher microbiological response rates than monotherapy [824]. The high initial resistance rate observed for doripenem, coupled with its subsequent limited prescription, illustrates an important aspect of antimicrobial stewardship: the necessity of reserving certain antibiotics in cases where other treatments have failed. This approach, while prudent, may inadvertently contribute to a scenario in which resistance data become skewed owing to the low volume of usage. This situation exemplifies the delicate balance required in prescribing practices, where the need to preserve the efficacy of last-resort antibiotics must be weighed against the potential to underestimate their resistance trends due to infrequent use.

The lack of efficacy of ertapenem against *A*. *baumannii* and its consequent non-preference for treatment protocols reflects another critical dimension of AMR management—drug specificity. Not all carbapenems are equally effective against all pathogens, underscoring the importance of targeted antibiotic therapy based on susceptibility to specific pathogens. This selective approach not only optimizes patient outcomes but also contributes to the broader effort to curb the spread of resistance by avoiding unnecessary or ineffective antibiotic use.

The variation in resistance rates for meropenem can be attributed to differences in the AST guidelines and methodologies. Specifically, studies utilizing the CLSI guidelines for AST reported lower resistance rates for meropenem than those using other standards. This discrepancy stems from the CLSI’s lower breakpoint values for determining resistance, leading to a higher estimated resistance rate when compared to the EUCAST guidelines. The fundamental difference in breakpoint definitions between CLSI and EUCAST accounts for these variations in resistance interpretation.

Additionally, the choice of the AST method plays a significant role in the reported resistance rates for meropenem. The MIC method, which quantifies the lowest concentration of an antimicrobial that inhibits the visible growth of a microorganism, showed significantly higher resistance rates compared to the mixed methods or the disk diffusion method. The disk diffusion method, which assesses the inhibition of bacterial growth around antibiotic-impregnated disks on agar plates, and mixed methods, which combine various AST techniques, have generally reported lower resistance rates for meropenem. These differences underscore the impact of AST methodologies on resistance rate estimates, highlighting the importance of standardization and careful interpretation of AST results in clinical and research settings. Furthermore, the significant overall increase in CRAB prevalence is a clarion to call for enhanced infection control measures, innovative antimicrobial development, and global cooperation in AMR surveillance. It emphasizes the necessity for a multifaceted strategy combining clinical vigilance, robust data analysis, and global health policy interventions to manage and mitigate the risks posed by highly resistant pathogens, such as *A*. *baumannii*. This surge in resistance coincided with the COVID-19 pandemic, suggesting a potential link between increased antibiotic use during the pandemic and the rise in the prevalence of CRAB. Parallel studies have also documented a similar increase in carbapenem resistance during this period, lending further support to this observation.

Geographical analyses have revealed substantial disparities in CRAB prevalence across continents and countries. Asia exhibited the highest incidence of carbapenem resistance at 76.2% [825], whereas the Americas had the lowest incidence (69.4%). Recent studies have reported resistance rates ranging from 50% to 75% [826]. Similarly, in China, carbapenem resistance in *A*. *baumannii* has increased significantly from 18% in 2012 to 60% in 2019 [827]. Moreover, a study focusing on CRAB in East Africa revealed an overall prevalence of resistance to carbapenems of 64.8% [12]. These findings highlight the widespread nature of carbapenem resistance in *A*. *baumannii* across different regions.

At the country level, resistance rates varied remarkably, ranging from 4% in Ireland to 96.1% in the Philippines. These variations highlight the complex interplay of regional factors influencing antibiotic resistance patterns and underscore the need for tailored intervention strategies based on local contexts.

Notably, subgroup analyses based on bias risk yielded unexpected results. Instances with a high bias risk unexpectedly showed lower resistance rates than those with a low bias risk. This counterintuitive observation emphasizes the potential influence of bias on reported resistance incidences and underscores the importance of rigorous methodological approaches in future research to ensure accurate and reliable data.

Through meticulous examination of reporting biases via the Egger and Begg tests, we reveal notable biases across various antibiotics, underscoring the need for cautious interpretation. Additionally, our subgroup analysis sheds light on the impact of sample size on resistance rates, providing nuanced understandings essential for refining antibiotic-resistance dynamics. Furthermore, our study’s robustness is reaffirmed by the Fail-Safe N method, which highlights the substantial additional studies required to overturn our significant findings, affirming the reliability of our results. Overall, our findings contribute substantially to the field, offering a comprehensive understanding of carbapenem resistance trends and bolstering efforts towards effective antimicrobial stewardship.

Despite the extensive and meticulous nature of this study, several limitations must be acknowledged. First, the reliance on published literature introduces the possibility of publication bias, as indicated by the significant results in Egger’s test for all antibiotics. Despite attempts to address this through statistical methods such as trim-and-fill, the inherent bias in the available literature may still impact the generalizability of the findings. Secondly, the high heterogeneity observed in some meta-analyses, especially for imipenem and meropenem resistance, raises concerns about the consistency of study methodologies and population characteristics across different regions and time periods. Additionally, the identification of potential outliers and asymmetry in the funnel plots emphasizes the need for cautious interpretation, suggesting the presence of influential studies that could skew the results. Finally, the study’s reliance on reported prevalence rates from various countries may be influenced by variations in surveillance systems, laboratory methods, and reporting practices, introducing potential biases in the dataset. These limitations highlight the complexity of synthesizing diverse data sources and emphasize the importance of considering contextual factors when interpreting the results of the meta-analysis.

## 5. Conclusion

This meta-analysis urgently underscores the necessity of addressing CRAB, particularly focusing on escalating resistance globally, a concern intensified during the COVID-19 pandemic. Given the rising resistance levels, imipenem has emerged as the most effective carbapenem against *A*. *baumannii*, with an average resistance rate of 78.1% over the past three years. Consequently, the use of these antibiotics should be approached with caution and prioritized after conducting antimicrobial susceptibility tests. In addition, there is a pressing need to explore combination therapies and novel approaches for treating CRAB. Geographical and temporal disparities in resistance patterns further complicate this issue by advocating tailored strategies. Emphasis on meticulous research practices and the call for transparent reporting are pivotal in this study. It appeals to an integrated approach involving comprehensive surveillance, strategic interventions, and synergy among researchers, healthcare practitioners, and policymakers to safeguard carbapenem effectiveness and counteract resistant strains. These insights are vital in navigating the complexities of emerging global health threats, marking a critical step toward mitigating CRAB’s impact of CRAB.

## Supporting information

S1 Checklist PRISMA 2020 checklist outlining the necessary components for transparent reporting of systematic reviews and meta-analyses. This checklist was used to ensure that our study adheres to PRISMA guidelines.
(DOCX)

S1 File A supplementary excel file containing a comprehensive list of all included studies and the extracted data for each. This file includes details of the studies reviewed, along with relevant data points and results extracted for analysis.
(XLSX)

S2 File (DOCX)
