# Correction: Geographical mapping and temporal trends of *Acinetobacter baumannii* carbapenem resistance: A comprehensive meta-analysis

**DOI:** 10.1371/journal.pone.0322955

**Published:** 2025-04-22

**Authors:** The PLOS One Staff

There is an error in affiliation 3 for author Tahereh Navidifar. The correct affiliation 3 is: Department of Basic Sciences, Shoushtar Faculty of Medical Sciences, Shoushtar, Iran.

The fourth author’s name is spelled incorrectly. The correct name is: Narjess Bostanghadiri.

The publisher apologizes for the errors.
