# Supplementary material for: Geographical mapping and temporal trends of Acinetobacter baumannii carbapenem resistance: A comprehensive meta-analysis
Source: PLoS One. 2024 Dec 16;19(12):e0311124. doi: 10.1371/journal.pone.0311124 (PMC11649148; doi:10.1371/journal.pone.0311124)
Supplement: S2 File — (DOCX) [file pone.0311124.s003.docx]

[The search syntax that was used for literature review in each online database. 2](#_Toc162531162)

[PubMed query: 2](#_Toc162531163)

[Web Of Science query: 2](#_Toc162531164)

[Scopus query: 2](#_Toc162531165)

[Embase query: 3](#_Toc162531166)

[Supplementary Table 1: All included article and extracted data that are used in meta-analysis 3](#_Toc162531167)

[Supplementary Table 2: Risk of Bias Assessment 34](#_Toc162531168)

[Supplementary Table 3: Detailed results of meta-analysis and subgroup analyzing 63](#_Toc162531169)

[Supplementary Figure 1: summary of risk of bias assessment or quality assessment of included studies 76](#_Toc162531170)

[Supplementary Figure 2: Funnel plots 78](#_Toc162531171)

[References: 78](#_Toc162531172)

# The search syntax that was used for literature review in each online database.

## PubMed query:

("*acinetobacter baumannii*"[Title/Abstract] OR "*A. baumannii*"[Title/Abstract]) AND (resistan*[Title/Abstract] OR susceptib*[Title/Abstract]) AND (Carbapenem*[Title/Abstract] OR "Doribax"[Title/Abstract] OR "Doripenem"[Title/Abstract] OR "Ertapenem"[Title/Abstract] OR "Imipenem/cilastatin"[Title/Abstract] OR "Imipenem/cilastatin/relebactam"[Title/Abstract] OR "Invanz"[Title/Abstract] OR "Meropenem"[Title/Abstract] OR "Meropenem/vaborbactam"[Title/Abstract] OR "Merrem IV"[Title/Abstract] OR "Primaxin"[Title/Abstract] OR "Recarbrio"[Title/Abstract] OR "Sulopenem"[Title/Abstract] OR "Sulopenem etzadroxil/probenecid"[Title/Abstract] OR "Vabomere"[Title/Abstract])

## Web Of Science query:

(“*acinetobacter baumannii*” OR “*A. baumannii*”) AND (resistan* OR susceptib*) AND (Carbapenem* OR “Doribax” OR “Doripenem” OR “Ertapenem” OR “Imipenem/cilastatin” OR “Imipenem/cilastatin/relebactam” OR “Invanz” OR “Meropenem” OR “Meropenem/vaborbactam” OR “Merrem IV” OR “Primaxin” OR “Recarbrio” OR “Sulopenem” OR “Sulopenem etzadroxil/probenecid” OR “Vabomere”) (Topic)

## Scopus query:

TITLE-ABS-KEY (("*acinetobacter baumannii*" OR "*A. baumannii*" ) AND (resistan* OR susceptib* ) AND (carbapenem* OR "Doribax" OR "Doripenem" OR "Ertapenem" OR "Imipenem/cilastatin" OR "Imipenem/cilastatin/relebactam" OR "Invanz" OR "Meropenem" OR "Meropenem/vaborbactam" OR "Merrem IV" OR "Primaxin" OR "Recarbrio" OR "Sulopenem" OR "Sulopenem etzadroxil/probenecid" OR "Vabomere") )

## Embase query:

(("*acinetobacter baumannii*" OR "*A. baumannii*" ) AND (resistan* OR susceptib* ) AND (carbapenem* OR "Doribax" OR "Doripenem" OR "Ertapenem" OR "Imipenem/cilastatin" OR "Imipenem/cilastatin/relebactam" OR "Invanz" OR "Meropenem" OR "Meropenem/vaborbactam" OR "Merrem IV" OR "Primaxin" OR "Recarbrio" OR "Sulopenem" OR "Sulopenem etzadroxil/probenecid" OR "Vabomere" ) );ab,ti

# Supplementary Table 1: All included article and extracted data that are used in meta-analysis

| Author | Year | Country | GUIDELINE | AST METHOS | Total isolate | Carbapenem resistant | Doripenem resistant | Ertapenem resistant | Imipenem resistant | Meropenem resistant |
| --- | --- | --- | --- | --- | --- | --- | --- | --- | --- | --- |
| T. Yungyuen, et al. (1) | **2021** | **Thailand** | **CLSI** | **MIXED** | **42616** | **41210** | **41351** | **NA** | **NA** | **41210** |
| F. P. Hu, et al. (2) | **2016** | **China** | **CLSI** | **DISK** | **56515** | **33072** | **NA** | **NA** | **NA** | **33072** |
| W.-M. Liu, et al. (3) | **2018** | **China** | **CLSI** | **NA** | **18852** | **14948** | **NA** | **NA** | **NA** | **14948** |
| K. Dafopoulou, et al. (4) | **2018** | **Greece** | **CLSI** | **MIXED** | **12646** | **11380** | **NA** | **NA** | **NA** | **11380** |
| C.-M. Lee, et al. (5) | **2013** | **Taiwan** | **CLSI** | **DISK** | **12346** | **9803** | **NA** | **NA** | **NA** | **9803** |
| Y. Li, et al. (6) | **2018** | **China** | **NA** | **NA** | **8765** | **7058** | **NA** | **NA** | **7058** | **NA** |
| A. U. Guclu, et al. (7) | **2021** | **Turkey** | **EUCAST** | **MIC** | **7364** | **6856** | **NA** | **NA** | **NA** | **6856** |
| Y. Ramsamy, et al. (8) | **2018** | **South Africa** | **CLSI** | **MIC** | **8010** | **5847** | **NA** | **NA** | **NA** | **5847** |
| D. J. Hoban, et al. (9) | **2015** | **NA** | **NA** | **MIC** | **12983** | **5336** | **NA** | **NA** | **NA** | **5336** |
| X. Tang, et al. (10) | **2018** | **China** | **CLSI** | **MIC** | **5076** | **5040** | **NA** | **NA** | **NA** | **5040** |
| H. Ismail, et al. (11) | **2019** | **South Africa** | **CLSI** | **NA** | **6492** | **4983** | **NA** | **NA** | **NA** | **4983** |
| M. Zhong, et al. (12) | **2019** | **China** | **CLSI** | **MIXED** | **6358** | **4889** | **NA** | **NA** | **NA** | **4889** |
| Y. Liu, et al. (13) | **2022** | **China** | **CLSI** | **MIXED** | **8902** | **4684** | **NA** | **NA** | **NA** | **4684** |
| M. Kumari, et al. (14) | **2019** | **India** | **CLSI** | **MIC** | **4680** | **4213** | **NA** | **NA** | **NA** | **4213** |
| J. M. Pogue, et al. (15) | **2022** | **US** | **CLSI** | **DISK** | **5523** | **3476** | **NA** | **NA** | **3476** | **NA** |
| V. Tien Viet Dung, Et (16) | **2021** | **Vietnam** | **CLSI** | **DISK** | **3622** | **2855** | **NA** | **NA** | **2855** | **NA** |
| J. A. KarLsky, Et (17) | **2022** | **NA** | **CLSI** | **MIC** | **5225** | **2743** | **NA** | **NA** | **NA** | **2743** |
| J. A. KarLsky, Et(18) | **2022** | **NA** | **CLSI** | **MIC** | **4038** | **2516** | **NA** | **NA** | **NA** | **2516** |
| R. Morfin-Otero, et al. (19) | **2012** | **NA** | **CLSI** | **MIC** | **6436** | **2447** | **NA** | **NA** | **NA** | **2447** |
| H. Seifert, et al. (20) | **2022** | **NA** | **CLSI** | **MIC** | **2482** | **2390** | **NA** | **NA** | **NA** | **2390** |
| L. Xu, et al. (21) | **2020** | **China** | **CLSI** | **MIC** | **3410** | **2182** | **NA** | **NA** | **NA** | **2182** |
| T. Xu, et al. (22) | **2013** | **China** | **CLSI** | **DISK** | **2831** | **2170** | **NA** | **NA** | **NA** | **2170** |
| R. Morfin-Otero, et al. (23) | **2013** | **Mexico** | **CLSI** | **MIC** | **3680** | **2135** | **NA** | **NA** | **NA** | **2135** |
| Y.-L. Lee, et al. (24) | **2023** | **NA** | **CLSI** | **MIC** | **2674** | **1917** | **NA** | **NA** | **NA** | **1917** |
| D. Said, et al. (25) | **2021** | **Germany** | **CLSI** | **NA** | **43948** | **1856** | **NA** | **NA** | **1856** | **NA** |
| H. Seifert, et al. (26) | **2018** | **Germany** | **CLSI** | **MIC** | **2720** | **1791** | **NA** | **NA** | **1791** | **NA** |
| V. M. D. Carvalho Hessel Dias, Et(27) (27) | **2021** | **Brazil** | **CLSI** | **MIXED** | **1969** | **1588** | **NA** | **NA** | **1588** | **NA** |
| G. M. Rossolini, et al. (28) | **2021** | **NA** | **CLSI** | **NA** | **1911** | **1574** | **NA** | **NA** | **1574** | **NA** |
| S. S. Akcay, et al. (29) | **2014** | **Turkey** | **CLSI** | **DISK** | **2124** | **1537** | **NA** | **NA** | **NA** | **1537** |
| M. B. Sannathimmappa, et al. (30)Al.(30) | **2021** | **Oman** | **CLSI** | **DISK** | **1890** | **1323** | **NA** | **NA** | **NA** | **1323** |
| Z. Ruan, et al. (31) | **2013** | **China** | **CLSI** | **DISK** | **2197** | **1294** | **NA** | **NA** | **1294** | **1339** |
| S. Zhong, et al. (32) | **2021** | **China** | **CLSI** | **MIC** | **2418** | **1274** | **NA** | **NA** | **NA** | **1274** |
| N. Khursheed, et al. (33) | **2021** | **Pakistan** | **CLSI** | **DISK** | **1332** | **1209** | **NA** | **NA** | **NA** | **1209** |
| H. Duran, et al. (34) | **2021** | **Turkey** | **EUCAST** | **MIC** | **1143** | **1113** | **NA** | **NA** | **1113** | **NA** |
| Y. Chen, et al. (35) | **2022** | **China** | **CLSI** | **MIC** | **1654** | **1062** | **NA** | **NA** | **1062** | **NA** |
| S. Santajit, et al. (36) | **2023** | **Thailand** | **CLSI** | **DISK** | **995** | **995** | **NA** | **NA** | **NA** | **995** |
| C. Rodriguez-Lucas, et al. (37) | **2021** | **Spain** | **CLSI** | **MIC** | **1154** | **993** | **NA** | **NA** | **993** | **NA** |
| H. Zhang, et al. (38) | **2020** | **China** | **CLSI** | **MIC** | **1251** | **984** | **NA** | **NA** | **NA** | **984** |
| M. Castanheira, et al. (39) | **2009** | **NA** | **CLSI** | **MIC** | **2982** | **960** | **NA** | **NA** | **NA** | **960** |
| Z. Zhang, et al. (40) | **2022** | **China** | **CLSI** | **MIXED** | **1391** | **956** | **NA** | **NA** | **956** | **795** |
| S. M. Mcleod, et al. (41) | **2020** | **NA** | **CLSI** | **MIC** | **1420** | **914** | **NA** | **NA** | **NA** | **914** |
| D. J. Biedenbach, et al. (42) | **2016** | **Vietnam** | **CLSI** | **MIC** | **971** | **899** | **NA** | **NA** | **NA** | **899** |
| P. A. Moise, et al. (43) | **2021** | **US** | **CLSI** | **MIC** | **1005** | **888** | **NA** | **NA** | **NA** | **888** |
| L. Azimi, et al. (44) | **2023** | **Iran** | **CLSI** | **DISK** | **879** | **870** | **NA** | **NA** | **NA** | **870** |
| D. Man, et al. (45) | **2007** | **US** | **CLSI** | **NA** | **1286** | **840** | **NA** | **NA** | **NA** | **840** |
| Q. Yang, et al. (46) | **2020** | **China** | **CLSI** | **MIC** | **984** | **830** | **NA** | **NA** | **NA** | **830** |
| R. M. Humphries, et al. (47) | **2023** | **NA** | **CLSI** | **MIC** | **1267** | **815** | **NA** | **NA** | **815** | **NA** |
| S. Vijayakumar, et al. (48) | **2020** | **India** | **CLSI** | **DISK** | **763** | **763** | **NA** | **NA** | **NA** | **763** |
| R. C. Mashau, et al. (49) | **2022** | **South Africa** | **CLSI** | **DISK** | **5216** | **754** | **NA** | **NA** | **NA** | **754** |
| N. U. Tuzemen, et al. (50) | **2022** | **Turkey** | **CLSI** | **MIXED** | **864** | **714** | **NA** | **649** | **NA** | **714** |
| A. Sedaghat, et al. (51) | **2019** | **Iran** | **CLSI** | **DISK** | **1985** | **706** | **NA** | **NA** | **NA** | **706** |
| C. Liu, et al. (52) | **2022** | **China** | **CLSI** | **DISK** | **792** | **693** | **NA** | **NA** | **NA** | **693** |
| J. Chen, et al. (53) | **2021** | **China** | **CLSI** | **MIC** | **1062** | **676** | **NA** | **NA** | **NA** | **676** |
| Y.-M. Xu, et al. (54) | **2016** | **China** | **CLSI** | **MIC** | **1062** | **675** | **NA** | **NA** | **NA** | **675** |
| F. Marco, et al. (55) | **2016** | **Spain** | **EUCAST** | **DISK** | **1312** | **656** | **NA** | **NA** | **NA** | **656** |
| S. Maraki, et al. (56) | **2016** | **Greece** | **CLSI** | **MIC** | **914** | **656** | **NA** | **NA** | **NA** | **656** |
| X.-G. Hu, et al. (54, 57) | **2017** | **Greece** | **CLSI** | **MIXED** | **914** | **656** | **NA** | **NA** | **NA** | **656** |
| Q. Wang, et al. (58) | **2020** | **China** | **CLSI** | **MIC** | **926** | **640** | **NA** | **NA** | **NA** | **640** |
| D. Anggraini, et al. (59) | **2022** | **Indonesia** | **CLSI** | **MIC** | **1263** | **621** | **NA** | **NA** | **NA** | **621** |
| C.-H. Chen, et al. (60) | **2023** | **NA** | **CLSI** | **MIC** | **847** | **620** | **NA** | **NA** | **NA** | **620** |
| J. Nadia, et al. (61) | **2019** | **Tunisia** | **EUCAST** | **DISK** | **691** | **612** | **NA** | **NA** | **612** | **NA** |
| J. Tian, et al. (62) | **2018** | **China** | **CLSI** | **MIXED** | **848** | **604** | **NA** | **NA** | **NA** | **604** |
| L. Rodrigues Perez, et al. (63) | **2021** | **Brazil** | **CLSI** | **MIC** | **659** | **596** | **NA** | **NA** | **NA** | **596** |
| G. A≈üik, et al. (64) | **2014** | **Turkey** | **CLSI** | **DISK** | **763** | **582** | **NA** | **NA** | **NA** | **582** |
| D. Kim, et al. (65) | **2021** | **South Korea** | **CLSI** | **MIXED** | **559** | **542** | **NA** | **NA** | **542** | **NA** |
| H. Caskurlu, et al. (66) | **2020** | **Turkey** | **CLSI** | **DISK** | **580** | **540** | **NA** | **NA** | **NA** | **540** |
| A. Salmanov, et al. (66) | **2023** | **Ukraine** | **EUCAST** | **DISK** | **720** | **538** | **NA** | **NA** | **538** | **NA** |
| A. Balode, et al. (67) | **2013** | **NA** | **CLSI** | **MIC** | **1093** | **534** | **NA** | **NA** | **NA** | **534** |
| T. Mao, et al. (68) | **2019** | **China** | **CLSI** | **MIC** | **1108** | **534** | **NA** | **NA** | **NA** | **534** |
| S.-C. Kuo, et al. (69) | **2020** | **NA** | **CLSI** | **MIC** | **627** | **529** | **NA** | **NA** | **529** | **NA** |
| Y. Sun, et al. (70) | **2016** | **China** | **CLSI** | **MIC** | **510** | **502** | **NA** | **NA** | **NA** | **502** |
| Z. Zhang, et al. (71) | **2018** | **NA** | **CLSI** | **MIC** | **749** | **500** | **NA** | **NA** | **500** | **NA** |
| ≈û. Direkel, et al. (72) | **2015** | **Turkey** | **NA** | **DISK** | **531** | **484** | **NA** | **NA** | **NA** | **484** |
| A. Guzek, et al. (73) | **2017** | **Poland** | **EUCAST** | **MIC** | **764** | **473** | **NA** | **NA** | **NA** | **473** |
| M. Al-Tamimi, et al. (74) | **2022** | **Jordan** | **CLSI** | **MIC** | **622** | **467** | **NA** | **NA** | **NA** | **467** |
| S. Yang, et al. (75) | **2019** | **China** | **CLSI** | **MIC** | **750** | **440** | **NA** | **NA** | **NA** | **440** |
| E. Riccobono, et al. (76) | **2019** | **NA** | **EUCAST** | **MIC** | **462** | **424** | **NA** | **NA** | **424** | **NA** |
| H. S. Sader, et al. (77) | **2021** | **NA** | **CLSI** | **MIC** | **1250** | **423** | **NA** | **NA** | **NA** | **423** |
| Y. Yang, et al. (78) | **2020** | **China** | **CLSI** | **MIC** | **515** | **406** | **NA** | **NA** | **NA** | **406** |
| Y. Li, et al. (79) | **2015** | **China** | **NA** | **MIC** | **594** | **404** | **400** | **NA** | **NA** | **404** |
| V. M. Musyoki, et al. (80) | **2019** | **Kenya** | **CLSI** | **DISK** | **590** | **403** | **NA** | **NA** | **403** | **NA** |
| N. Jaidane, et al. (81) | **2018** | **Tunisia** | **CLSI** | **DISK** | **408** | **376** | **NA** | **NA** | **376** | **NA** |
| R. Han, et al. (82) | **2022** | **China** | **CLSI** | **MIC** | **536** | **372** | **NA** | **NA** | **NA** | **372** |
| W. Flores-Paredes, et al. (83) | **2021** | **Peru** | **CLSI** | **MIC** | **604** | **368** | **NA** | **NA** | **NA** | **368** |
| S. Kang, et al. (84) | **2022** | **South Korea** | **CLSI** | **DISK** | **528** | **366** | **NA** | **NA** | **366** | **NA** |
| M. T. Della Rocca, et al. (85) | **2023** | **Italy** | **EUCAST** | **MIC** | **374** | **361** | **NA** | **NA** | **NA** | **361** |
| R. I. Aloraifi, et al. (86) | **2023** | **Saudi Arabia** | **NA** | **NA** | **381** | **355** | **NA** | **NA** | **NA** | **355** |
| N. Jumroon, et al. (87) | **2013** | **Thailand** | **CLSI** | **DISK** | **353** | **353** | **NA** | **NA** | **NA** | **353** |
| K. Nafplioti, et al. (88) | **2020** | **Greece** | **CLSI** | **MIC** | **347** | **347** | **NA** | **NA** | **347** | **NA** |
| T. A. Davies, et al. (89) | **2011** | **US** | **CLSI** | **MIC** | **994** | **341** | **405** | **NA** | **NA** | **341** |
| M. Mhondoro, et al. (90) | **2019** | **Zimbabwe** | **CLSI** | **DISK** | **616** | **336** | **NA** | **NA** | **NA** | **336** |
| M. Chaudhary, et al. (91) | **2013** | **India** | **CLSI** | **DISK** | **371** | **332** | **293** | **NA** | **NA** | **332** |
| J. Houngsaitong, et al. (92) | **2020** | **Thailand** | **CLSI** | **MIC** | **412** | **326** | **NA** | **NA** | **NA** | **326** |
| E. Garza-Gonzalez, et al. (93) | **2010** | **Mexico** | **CLSI** | **MIC** | **550** | **324** | **NA** | **NA** | **NA** | **324** |
| E.-J. Yoon, et al. (94) | **2017** | **South Korea** | **CLSI** | **MIC** | **356** | **314** | **NA** | **NA** | **NA** | **314** |
| S. Sharma, et al. (95) | **2022** | **India** | **CLSI** | **DISK** | **356** | **311** | **NA** | **NA** | **NA** | **311** |
| A. Sangale, et al. (96) | **2021** | **India** | **CLSI** | **DISK** | **320** | **309** | **NA** | **NA** | **NA** | **309** |
| Q. Fu, et al. (97) | **2013** | **China** | **CLSI** | **MIXED** | **438** | **308** | **NA** | **NA** | **NA** | **308** |
| M. Jiang, et al. (98) | **2021** | **China** | **CLSI** | **MIC** | **295** | **293** | **NA** | **NA** | **NA** | **293** |
| J. M. Velasco, et al. (99) | **2020** | **Philippines** | **CLSI** | **MIC** | **293** | **292** | **NA** | **NA** | **NA** | **292** |
| F. Sana, et al. (100) | **2021** | **Pakistan** | **CLSI** | **DISK** | **310** | **286** | **NA** | **NA** | **NA** | **286** |
| N. Kashkouri, et al. (101) | **2022** | **Iran** | **CLSI** | **DISK** | **490** | **284** | **NA** | **NA** | **NA** | **284** |
| Y. Han, et al. (102) | **2022** | **China** | **NA** | **DISK** | **378** | **281** | **NA** | **NA** | **NA** | **281** |
| T. Paiboonvong, et al. (103) | **2020** | **Thailand** | **CLSI** | **MIC** | **350** | **278** | **NA** | **NA** | **278** | **NA** |
| M. Eslami, et al. (104) | **2019** | **Iran** | **CLSI** | **DISK** | **300** | **276** | **NA** | **NA** | **NA** | **276** |
| M. M. E. Meybodi, et al. (105) | **2021** | **Iran** | **CLSI** | **DISK** | **282** | **272** | **NA** | **NA** | **272** | **NA** |
| L. Wang, et al. (106) | **2021** | **China** | **CLSI** | **MIC** | **300** | **264** | **NA** | **NA** | **NA** | **264** |
| J. M. Khaled, et al. (107) | **2021** | **Saudi Arabia** | **CLSI** | **MIC** | **342** | **259** | **NA** | **NA** | **NA** | **259** |
| V. Rodjun, et al. (108) | **2020** | **Thailand** | **CLSI** | **MIC** | **300** | **258** | **NA** | **NA** | **258** | **NA** |
| M. Dolores Alcantar-Curiel, et al. (109) | **2014** | **Mexico** | **NA** | **MIC** | **303** | **254** | **NA** | **NA** | **NA** | **254** |
| H. S. Sader, et al. (110) | **2022** | **US** | **CLSI** | **MIC** | **349** | **249** | **NA** | **NA** | **NA** | **249** |
| R. Khoshbakht, et al. (111) | **2022** | **Iran** | **CLSI** | **DISK** | **334** | **248** | **NA** | **NA** | **NA** | **248** |
| H. Yi, et al. (112) | **2020** | **China** | **CLSI** | **MIXED** | **284** | **243** | **NA** | **NA** | **NA** | **243** |
| L. Principe, et al. (113) | **2014** | **Italy** | **NA** | **MIC** | **246** | **237** | **241** | **NA** | **NA** | **237** |
| G. Samonis, et al. (114) | **2012** | **Greece** | **CLSI** | **MIC** | **1242** | **235** | **NA** | **NA** | **NA** | **235** |
| A. Spiliopoulou, et al. (115) | **2015** | **Greece** | **CLSI** | **MIXED** | **295** | **235** | **NA** | **NA** | **NA** | **235** |
| B. Mirzaei, et al. (116) | **2020** | **Iran** | **CLSI** | **DISK** | **234** | **226** | **NA** | **NA** | **NA** | **226** |
| B. Lukovic, et al. (117) | **2020** | **Serbia** | **EUCAST** | **MIC** | **237** | **222** | **NA** | **NA** | **NA** | **222** |
| H. Wang, et al. (118) | **2007** | **China** | **CLSI** | **MIC** | **221** | **221** | **NA** | **NA** | **NA** | **221** |
| A. T. √ái√ßek, et al. (119) | **2013** | **Turkey** | **CLSI** | **MIC** | **281** | **220** | **NA** | **NA** | **NA** | **220** |
| U. Leungtongkam, et al. (120) | **2018** | **Thailand** | **CLSI** | **DISK** | **339** | **220** | **NA** | **NA** | **NA** | **220** |
| D. Alrahmany, et al. (121) | **2021** | **Oman** | **CLSI** | **DISK** | **260** | **216** | **NA** | **NA** | **NA** | **216** |
| M. Dolores Alcantar-Curiel, et al. (122) | **2019** | **Mexico** | **CLSI** | **DISK** | **252** | **210** | **NA** | **NA** | **210** | **NA** |
| Lopez-Hern, et al. (123) | **2020** | **Spain** | **EUCAST** | **MIC** | **425** | **208** | **NA** | **NA** | **208** | **NA** |
| J. Qu, et al. (124) | **2021** | **China** | **CLSI** | **MIC** | **208** | **207** | **NA** | **NA** | **NA** | **207** |
| J. Martin Llaca-Diaz, et al. (125) | **2012** | **Mexico** | **CLSI** | **MIC** | **268** | **202** | **NA** | **NA** | **NA** | **202** |
| O. Katoch, et al. (126) | **2022** | **India** | **CLSI** | **MIC** | **205** | **201** | **NA** | **NA** | **NA** | **201** |
| S. Kooti, et al. (127) | **2015** | **Iran** | **NA** | **DISK** | **200** | **199** | **NA** | **NA** | **NA** | **199** |
| S. M. Kareem(128) | **2020** | **Iraq** | **CLSI** | **DISK** | **205** | **195** | **NA** | **NA** | **195** | **NA** |
| A. Prasai, et al. (129) | **2021** | **Nepal** | **NA** | **DISK** | **196** | **193** | **NA** | **NA** | **193** | **NA** |
| N. A. Chitrabanu, et al. (130) | **2021** | **India** | **CLSI** | **DISK** | **393** | **193** | **NA** | **NA** | **NA** | **193** |
| M. C. Silveira, et al. (131) | **2021** | **Brazil** | **EUCAST** | **MIC** | **192** | **189** | **NA** | **NA** | **NA** | **189** |
| M. Guvenir, et al. (132) | **2021** | **Cyprus** | **EUCAST** | **MIC** | **218** | **189** | **NA** | **NA** | **NA** | **189** |
| C. Liu, et al. (133) | **2019** | **South Korea** | **CLSI** | **DISK** | **203** | **187** | **NA** | **NA** | **NA** | **187** |
| A. Alamri, et al. (134) | **2018** | **Saudi Arabia** | **NA** | **MIC** | **2096** | **186** | **NA** | **NA** | **NA** | **186** |
| A. R. Hamzeh, et al. (135) | **2012** | **Syria** | **CLSI** | **MIC** | **260** | **183** | **NA** | **NA** | **NA** | **183** |
| A. A. Alsultan, et al. (136) | **2013** | **Saudi Arabia** | **BSAC** | **MIC** | **196** | **181** | **NA** | **NA** | **181** | **186** |
| O. F. Nwabor, et al. (137) | **2021** | **Thailand** | **CLSI** | **DISK** | **193** | **180** | **NA** | **NA** | **NA** | **180** |
| H. S. Sader, et al. (138) | **2022** | **US** | **CLSI** | **MIC** | **550** | **180** | **NA** | **NA** | **NA** | **180** |
| S. V. Bharathi, et al. (139) | **2021** | **Greece** | **EUCAST** | **MIC** | **181** | **178** | **NA** | **NA** | **NA** | **178** |
| A. Lavrinenko, et al. (140) | **2021** | **Kazakhstan** | **EUCAST** | **MIC** | **224** | **176** | **NA** | **NA** | **NA** | **176** |
| G. Ziolkowski, et al. (141) | **2018** | **Poland** | **EUCAST** | **MIC** | **187** | **173** | **NA** | **NA** | **NA** | **173** |
| O. Perovic, et al. (142) | **2018** | **South Africa** | **CLSI** | **NA** | **304** | **171** | **NA** | **NA** | **NA** | **171** |
| S. S. Khoramrooz, et al. (143) | **2021** | **India** | **CLSI** | **DISK** | **174** | **170** | **NA** | **NA** | **NA** | **170** |
| S. Vijay, et al. (144) | **2021** | **India** | **CLSI** | **MIC** | **212** | **170** | **NA** | **NA** | **NA** | **170** |
| E. Cercenado, et al. (145) | **2021** | **Spain** | **EUCAST** | **MIC** | **175** | **169** | **NA** | **NA** | **NA** | **169** |
| N. Javaid, et al. (146) | **2021** | **Pakistan** | **CLSI** | **DISK** | **189** | **167** | **NA** | **NA** | **NA** | **167** |
| X. Meng, et al. (147) | **2021** | **China** | **NA** | **MIC** | **202** | **167** | **NA** | **NA** | **167** | **NA** |
| J. A. KarLsky, et al. (148) | **2022** | **Morocco** | **CLSI** | **MIC** | **191** | **165** | **NA** | **NA** | **NA** | **165** |
| A. Miller, et al. (149) | **2020** | **US** | **CLSI** | **NA** | **190** | **163** | **NA** | **NA** | **163** | **NA** |
| E. Caglan, et al. (150) | **2020** | **Turkey** | **CLSI** | **DISK** | **200** | **163** | **NA** | **NA** | **NA** | **163** |
| A. C. Yardimci, et al. (151) | **2022** | **Turkey** | **EUCAST** | **MIC** | **178** | **163** | **NA** | **NA** | **NA** | **163** |
| G. Bou, et al. (152) | **2012** | **Spain** | **CLSI** | **MIC** | **322** | **160** | **NA** | **NA** | **NA** | **160** |
| M. G. Donadu, et al. (153) | **2021** | **Italy** | **EUCAST** | **DISK** | **309** | **159** | **NA** | **NA** | **NA** | **159** |
| K. L. Li, et al. (154) | **2020** | **Italy** | **NA** | **MIC** | **197** | **157** | **NA** | **NA** | **NA** | **157** |
| H. Masoumi-Asl, et al. (155) | **2021** | **Iran** | **CLSI** | **DISK** | **195** | **156** | **NA** | **NA** | **156** | **NA** |
| R. Ranjbar, et al. (156) | **2019** | **Iran** | **CLSI** | **DISK** | **163** | **154** | **NA** | **NA** | **NA** | **154** |
| S. K. Yadav, et al. (157) | **2020** | **Nepal** | **CLSI** | **DISK** | **177** | **154** | **NA** | **NA** | **NA** | **154** |
| A. Rezaei, et al. (158) | **2018** | **Iran** | **CLSI** | **DISK** | **153** | **152** | **NA** | **NA** | **NA** | **152** |
| C. Hou, et al. (159) | **2015** | **China** | **NA** | **MIC** | **196** | **150** | **NA** | **196** | **NA** | **150** |
| S. H. Jun, et al. (160) | **2023** | **North Korea** | **CLSI** | **MIC** | **167** | **147** | **NA** | **NA** | **NA** | **147** |
| T. P. Gomes Chagas, et al. (161) | **2014** | **Brazil** | **NA** | **MIC** | **155** | **146** | **NA** | **NA** | **NA** | **146** |
| X. Zhen, et al. (162) | **2017** | **Colombia** | **CLSI** | **MIC** | **220** | **146** | **NA** | **NA** | **146** | **NA** |
| T. Naas, et al. (163) | **2021** | **France** | **CLSI** | **MIC** | **161** | **145** | **NA** | **NA** | **NA** | **145** |
| M.-H. Lee, et al. (164) | **2013** | **Taiwan** | **CLSI** | **MIC** | **291** | **142** | **NA** | **NA** | **142** | **NA** |
| M. Moosavian, et al. (165) | **2017** | **Iran** | **CLSI** | **DISK** | **152** | **142** | **NA** | **NA** | **NA** | **142** |
| Y. S. Huang, et al. (166) | **2013** | **Taiwan** | **CLSI** | **MIC** | **146** | **141** | **NA** | **NA** | **NA** | **141** |
| A. Alimohammadi, et al. (167) | **2023** | **Iran** | **CLSI** | **DISK** | **141** | **141** | **NA** | **NA** | **NA** | **141** |
| A. Camacho-Ortiz, et al. (168) | **2021** | **Mexico** | **CLSI** | **MIC** | **149** | **139** | **NA** | **NA** | **NA** | **139** |
| A. Shahid, et al. (169) | **2021** | **Pakistan** | **CLSI** | **DISK** | **148** | **139** | **NA** | **NA** | **NA** | **139** |
| S.-S. Jean, et al. (170) | **2013** | **Taiwan** | **CLSI** | **MIC** | **192** | **138** | **NA** | **NA** | **NA** | **138** |
| Z. Tayebi, et al. (171) | **2019** | **Iran** | **CLSI** | **DISK** | **150** | **138** | **NA** | **NA** | **NA** | **138** |
| S. Chusri, et al. (172) | **2017** | **Thailand** | **NA** | **MIXED** | **197** | **136** | **NA** | **NA** | **136** | **NA** |
| B. Hashemi, et al. (173) | **2020** | **Iran** | **NA** | **DISK** | **150** | **136** | **NA** | **NA** | **NA** | **136** |
| G. G. Gaspar, et al. (174) | **2021** | **Brazil** |  | **MIC** | **173** | **136** | **NA** | **NA** | **136** | **NA** |
| P. Khuntayaporn, et al. (175) | **2021** | **Thailand** | **CLSI** | **MIC** | **135** | **135** | **135** | **NA** | **NA** | **135** |
| Z. Mohammadtaheri, et al. (176) | **2010** | **Iran** | **CLSI** | **DISK** | **136** | **134** | **NA** | **NA** | **134** | **NA** |
| F. Jabeen, et al. (177) | **2022** | **Pakistan** | **CLSI** | **DISK** | **156** | **133** | **NA** | **82** | **NA** | **133** |
| M. R. Rao, et al. (178) | **2022** | **India** | **CLSI** | **DISK** | **149** | **132** | **NA** | **NA** | **NA** | **132** |
| J. Oteo, et al. (179) | **2007** | **Spain** | **CLSI** | **MIC** | **345** | **129** | **NA** | **NA** | **NA** | **129** |
| Y. Zhou, et al. (180) | **2016** | **China** | **CLSI** | **DISK** | **170** | **129** | **NA** | **NA** | **NA** | **129** |
| S. Mendoza-Olazaran, et al. (181) | **2014** | **Mexico** | **NA** | **MIC** | **149** | **128** | **NA** | **NA** | **NA** | **128** |
| S. Biglari, et al. (182) | **2015** | **Malaysia** | **NA** | **MIC** | **167** | **128** | **NA** | **NA** | **NA** | **128** |
| S. Biglari, et al. (183) | **2017** | **Malaysia** | **CLSI** | **NA** | **162** | **128** | **NA** | **NA** | **128** | **NA** |
| S. Farajnia, et al. (184) | **2022** | **Iran** | **CLSI** | **DISK** | **127** | **127** | **NA** | **NA** | **NA** | **127** |
| S. Ghasemi, et al. (185) | **2022** | **Iran** | **CLSI** | **DISK** | **133** | **126** | **NA** | **NA** | **NA** | **126** |
| X.-M. Xiao, et al. (186) | **2017** | **US** | **CLSI** | **MIC** | **433** | **125** | **NA** | **NA** | **NA** | **125** |
| P. Nordmann, et al. (187) | **2011** | **NA** | **CLSI** | **MIC** | **274** | **124** | **110** | **NA** | **NA** | **124** |
| B. Salehi, et al. (188) | **2018** | **Iran** | **CLSI** | **MIXED** | **125** | **124** | **NA** | **NA** | **NA** | **124** |
| M. Le, et al. (189) | **2018** | **South Africa** | **EUCAST** | **MIC** | **141** | **123** | **NA** | **NA** | **NA** | **123** |
| A. Chmielarczyk, et al. (190) | **2016** | **Poland** | **CLSI** | **DISK** | **125** | **122** | **NA** | **NA** | **NA** | **122** |
| J. M. Pogue, et al. (191) | **2019** | **Egypt** | **NA** | **MIC** | **120** | **120** | **NA** | **NA** | **120** | **NA** |
| S. Shrestha, et al. (192) | **2015** | **Nepal** | **CLSI** | **DISK** | **121** | **119** | **NA** | **NA** | **NA** | **119** |
| F. Zhang, et al. (193) | **2019** | **China** | **CLSI** | **MIC** | **160** | **118** | **NA** | **NA** | **NA** | **118** |
| R. Bawazeer, et al. (194) | **2020** | **Saudi Arabia** | **NA** | **MIC** | **203** | **118** | **NA** | **NA** | **118** | **NA** |
| T. B. Talizin, et al. (195) | **2020** | **Brazil** | **CLSI** | **DISK** | **121** | **118** | **NA** | **NA** | **118** | **NA** |
| A. Balkhair, et al. (196) | **2019** | **Oman** | **CLSI** | **MIC** | **166** | **117** | **NA** | **NA** | **117** | **NA** |
| S. M. Amudhan, et al. (197) | **2011** | **NA** | **CLSI** | **DISK** | **116** | **116** | **NA** | **NA** | **NA** | **116** |
| M. S. Amudhan, et al. (198) | **2012** | **India** | **CLSI** | **DISK** | **116** | **116** | **NA** | **NA** | **NA** | **116** |
| C. Vuotto, et al. (199) | **2018** | **NA** | **CLSI** | **MIC** | **128** | **115** | **NA** | **NA** | **115** | **NA** |
| H. Ejaz, et al. (200) | **2021** | **Saudi Arabia** | **CLSI** | **MIC** | **147** | **113** | **NA** | **NA** | **NA** | **113** |
| S. Bagheri Josheghani, et al. (201) | **2015** | **Iran** | **NA** | **DISK** | **124** | **112** | **NA** | **NA** | **NA** | **112** |
| A. Vahhabi, et al. (202) | **2021** | **Iran** | **CLSI** | **MIXED** | **112** | **112** | **NA** | **NA** | **NA** | **112** |
| K. Dobrovic, et al. (203) | **2023** | **Croatia** | **EUCAST** | **MIC** | **112** | **112** | **NA** | **NA** | **NA** | **112** |
| T. A. Hafiz, et al. (204) | **2023** | **Saudi Arabia** | **CLSI** | **MIXED** | **115** | **112** | **NA** | **NA** | **112** | **NA** |
| A. Smitran, et al. (205) | **2023** | **Serbia** | **CLSI** | **MIC** | **117** | **111** | **NA** | **NA** | **111** | **NA** |
| K. Jeannot, et al. (206) | **2014** | **France** | **NA** | **DISK** | **283** | **110** | **NA** | **NA** | **110** | **NA** |
| M. Gholami, et al. (207) | **2018** | **Iran** | **CLSI** | **DISK** | **110** | **110** | **NA** | **NA** | **NA** | **110** |
| A. Miller, et al. (208) | **2019** | **India** | **CLSI** | **DISK** | **121** | **110** | **NA** | **NA** | **NA** | **110** |
| P. Khuntayaporn, et al. (209) | **2021** | **Thailand** | **CLSI** | **MIC** | **123** | **110** | **NA** | **NA** | **110** | **NA** |
| Z. Meshkat, et al. (210) | **2019** | **Iran** | **CLSI** | **DISK** | **122** | **109** | **112** | **NA** | **109** | **107** |
| R. Rosales-Reyes, et al. (211) | **2017** | **Mexico** | **CLSI** | **MIXED** | **112** | **108** | **NA** | **NA** | **NA** | **108** |
| K. Yu, et al. (212) | **2021** | **China** | **CLSI** | **MIC** | **108** | **108** | **NA** | **NA** | **NA** | **108** |
| H. Ziglam, et al. (213) | **2012** | **Libya** | **CLSI** | **MIC** | **167** | **107** | **NA** | **NA** | **NA** | **107** |
| M. Nikibakhsh, et al. (214) | **2021** | **Iran** | **CLSI** | **DISK** | **106** | **106** | **NA** | **NA** | **NA** | **106** |
| E. A. De Oliveira, et al. (215) | **2019** | **Brazil** | **CLSI** | **DISK** | **114** | **105** | **NA** | **NA** | **NA** | **105** |
| F. Hu, et al. (216) | **2022** | **China** | **CLSI** | **MIC** | **148** | **105** | **NA** | **NA** | **NA** | **105** |
| A. Karmostaji, et al. (217) | **2013** | **Iran** | **CLSI** | **DISK** | **123** | **104** | **NA** | **NA** | **NA** | **104** |
| R. Chen, et al. (218) | **2016** | **China** | **CLSI** | **MIC** | **148** | **104** | **NA** | **NA** | **NA** | **104** |
| G. Mir, et al. (219) | **2020** | **Mexico** | **CLSI** | **DISK** | **153** | **104** | **NA** | **NA** | **NA** | **104** |
| S. Mostafavi, et al. (220) | **2021** | **Iran** | **CLSI** | **DISK** | **153** | **104** | **NA** | **NA** | **NA** | **104** |
| S. S. Mabrouk, et al. (221) | **2020** | **Egypt** | **CLSI** | **MIC** | **129** | **102** | **NA** | **NA** | **NA** | **102** |
| G. Al-Hashem, et al. (222) | **2021** | **Kuwait** | **CLSI** | **MIC** | **117** | **102** | **NA** | **NA** | **NA** | **102** |
| P. Simeon, et al. (223) | **2021** | **Namibia** | **CLSI** | **DISK** | **108** | **101** | **NA** | **NA** | **NA** | **101** |
| R. Abozahra, et al. (224) | **2021** | **Egypt** | **CLSI** | **DISK** | **120** | **101** | **NA** | **110** | **NA** | **101** |
| M. Saadati, et al. (225) | **2021** | **Iran** | **CLSI** | **DISK** | **100** | **100** | **NA** | **NA** | **NA** | **100** |
| Sepahv, et al. (226) | **2021** | **Iran** | **CLSI** | **DISK** | **100** | **100** | **NA** | **NA** | **NA** | **100** |
| F. Fallah, et al. (227) | **2014** | **Iran** | **NA** | **DISK** | **108** | **99** | **NA** | **NA** | **NA** | **99** |
| M. Noori, et al. (228) | **2019** | **Iran** | **CLSI** | **DISK** | **100** | **99** | **NA** | **NA** | **NA** | **99** |
| M. Alavi-Moghaddam, et al. (229) | **2020** | **Iran** | **CLSI** | **DISK** | **121** | **99** | **NA** | **NA** | **NA** | **99** |
| M. Moosavian, et al. (230) | **2020** | **Iran** | **CLSI** | **DISK** | **124** | **99** | **NA** | **NA** | **NA** | **99** |
| S. Mushtaq, et al. (231) | **2020** | **UK** | **CLSI** | **MIC** | **99** | **99** | **NA** | **NA** | **NA** | **99** |
| P. Jia, et al. (232) | **2022** | **China** | **CLSI** | **MIC** | **114** | **99** | **NA** | **NA** | **NA** | **99** |
| T. Y. Tan, et al. (233) | **2008** | **Singapore** | **CLSI** | **MIC** | **139** | **98** | **NA** | **NA** | **NA** | **98** |
| E. Uzunoglu, et al. (234) | **2017** | **Turkey** | **CLSI** | **MIC** | **135** | **98** | **NA** | **NA** | **NA** | **98** |
| F. Mohammadi, et al. (235) | **2017** | **Iran** | **CLSI** | **MIXED** | **100** | **98** | **NA** | **NA** | **NA** | **98** |
| J. Loraine, et al. (236) | **2020** | **Thailand** | **CLSI** | **MIC** | **115** | **98** | **NA** | **NA** | **98** | **NA** |
| Y.-C. Hsieh, et al. (237) | **2020** | **Taiwan** | **CLSI** | **MIC** | **237** | **98** | **NA** | **NA** | **NA** | **98** |
| E. Abbasi, et al. (238) | **2021** | **Iran** | **CLSI** | **DISK** | **100** | **98** | **98** | **NA** | **NA** | **98** |
| R. Ranjbar, et al. (239) | **2019** | **Iran** | **CLSI** | **DISK** | **101** | **97** | **NA** | **NA** | **NA** | **97** |
| H. S. Sader, et al. (240) | **2020** | **NA** | **CLSI** | **MIC** | **105** | **97** | **NA** | **NA** | **NA** | **97** |
| F. Karami, et al. (241) | **2021** | **Iran** | **CLSI** | **DISK** | **108** | **97** | **NA** | **NA** | **NA** | **97** |
| U. S. S. Coskun, et al. (242) | **2019** | **Turkey** | **CLSI** | **MIXED** | **96** | **96** | **NA** | **NA** | **NA** | **96** |
| U. S. Say Coskun, et al. (242) | **2019** | **Turkey** | **CLSI** | **MIXED** | **96** | **96** | **NA** | **NA** | **NA** | **96** |
| G. Petazzoni, et al. (243) | **2022** | **Italy** | **CLSI** | **MIC** | **96** | **96** | **NA** | **NA** | **NA** | **96** |
| G. Odewale, et al. (244) | **2016** | **Nigeria** | **CLSI** | **DISK** | **150** | **95** | **NA** | **NA** | **NA** | **95** |
| M. N. Moghadam, et al. (245) | **2016** | **Iran** | **CLSI** | **MIC** | **98** | **95** | **NA** | **NA** | **NA** | **95** |
| F. Ahmadikiya, et al. (246) | **2017** | **Iran** | **CLSI** | **DISK** | **102** | **95** | **NA** | **NA** | **NA** | **95** |
| T. K. Atik, et al. (247) | **2019** | **NA** | **CLSI** | **MIC** | **100** | **95** | **NA** | **NA** | **NA** | **95** |
| N.-D. Nogbou, et al. (248) | **2021** | **South Africa** | **CLSI** | **MIC** | **100** | **95** | **NA** | **NA** | **NA** | **95** |
| S. Ruekit, et al. (249) | **2022** | **Thailand** | **CLSI** | **MIC** | **97** | **95** | **NA** | **97** | **NA** | **95** |
| S. Maraki, et al. (250) | **2012** | **Greece** | **CLSI** | **MIC** | **137** | **94** | **NA** | **NA** | **NA** | **94** |
| M. Safari, et al. (251) | **2013** | **Iran** | **CLSI** | **DISK** | **100** | **94** | **NA** | **NA** | **NA** | **94** |
| A. C. Cicek, et al. (252) | **2014** | **Turkey** | **NA** | **MIC** | **101** | **94** | **NA** | **NA** | **NA** | **94** |
| H. Lee, et al. (253) | **2018** | **NA** | **CLSI** | **MIC** | **188** | **94** | **NA** | **NA** | **NA** | **94** |
| M. Mohammadi, et al. (254) | **2017** | **Iran** | **CLSI** | **MIXED** | **103** | **93** | **NA** | **NA** | **NA** | **93** |
| N. Sharma, et al. (255) | **2021** | **Nepal** | **CLSI** | **DISK** | **121** | **93** | **NA** | **NA** | **NA** | **93** |
| B. Soltani, et al. (256) | **2018** | **Iran** | **CLSI** | **DISK** | **92** | **92** | **NA** | **NA** | **92** | **NA** |
| E. Kirkgoz, et al. (257) | **2014** | **Turkey** | **NA** | **MIC** | **92** | **91** | **NA** | **NA** | **NA** | **91** |
| A. M. Sultan, et al. (258) | **2018** | **Egypt** | **CLSI** | **MIXED** | **124** | **91** | **NA** | **NA** | **91** | **NA** |
| Y. Zhang, et al. (259) | **2021** | **China** | **CLSI** | **MIC** | **112** | **91** | **NA** | **NA** | **91** | **NA** |
| I. Erdem, et al. (260) | **2022** | **Turkey** | **NA** | **MIXED** | **123** | **91** | **NA** | **NA** | **91** | **NA** |
| M. K. Almaghrabi, et al. (261) | **2018** | **Saudi Arabia** | **NA** | **MIC** | **94** | **90** | **NA** | **NA** | **NA** | **90** |
| M. H. Scheetz, et al. (262) | **2007** | **US** | **CLSI** | **MIC** | **93** | **89** | **NA** | **NA** | **NA** | **89** |
| M. Sadr, et al. (263) | **2021** | **Iran** | **CLSI** | **DISK** | **90** | **89** | **NA** | **NA** | **NA** | **89** |
| X. Xu, et al. (264) | **2021** | **China** | **EUCAST** | **MIC** | **131** | **89** | **NA** | **NA** | **NA** | **89** |
| F. M. Rani, et al. (265) | **2018** | **Malaysia** | **CLSI** | **DISK** | **128** | **88** | **88** | **NA** | **NA** | **88** |
| X. Shi, et al. (266) | **2021** | **China** | **CLSI** | **MIC** | **94** | **88** | **NA** | **NA** | **NA** | **88** |
| J. A. Garcia-Rodriguez, et al. (267) | **2002** | **NA** | **CLSI** | **MIC** | **426** | **87** | **NA** | **NA** | **NA** | **87** |
| S. Kumar, et al. (268) | **2019** | **India** | **CLSI** | **DISK** | **161** | **86** | **NA** | **NA** | **86** | **NA** |
| P.-Y. Liu, et al. (269) | **2020** | **Taiwan** | **CLSI** | **MIC** | **199** | **85** | **NA** | **NA** | **NA** | **85** |
| A. Sonnevend, et al. (270) | **2013** | **NA** | **CLSI** | **MIC** | **110** | **84** | **NA** | **NA** | **NA** | **84** |
| Z. Chen, et al. (271) | **2013** | **China** | **CLSI** | **MIC** | **179** | **84** | **NA** | **NA** | **NA** | **84** |
| Q. Cuong Hoang, et al. (272) | **2019** | **Vietnam** | **CLSI** | **MIC** | **97** | **84** | **NA** | **NA** | **NA** | **84** |
| A. Ergin, et al. (273) | **2013** | **Turkey** | **CLSI** | **MIC** | **100** | **83** | **82** | **NA** | **NA** | **83** |
| S. Bahrami, et al. (274) | **2021** | **Iran** | **CLSI** | **DISK** | **155** | **83** | **NA** | **NA** | **83** | **NA** |
| J. Gao, et al. (275) | **2022** | **China** | **CLSI** | **MIC** | **103** | **83** | **NA** | **NA** | **NA** | **83** |
| N. Moradi, et al. (276) | **2021** | **Iran** | **CLSI** | **DISK** | **82** | **82** | **NA** | **NA** | **NA** | **82** |
| M. N. Lucas Kurihara, et al. (277) | **2022** | **Brazil** | **CLSI** | **MIC** | **89** | **82** | **NA** | **NA** | **82** | **NA** |
| C.-L. Liu, et al. (278) | **2020** | **Vietnam** | **CLSI** | **DISK** | **133** | **81** | **NA** | **NA** | **NA** | **81** |
| M. Lings, et al. (279) | **2015** | **South Africa** | **NA** | **MIC** | **94** | **80** | **NA** | **NA** | **NA** | **80** |
| Z. Moazzen, et al. (280) | **2018** | **Iran** | **CLSI** | **MIC** | **80** | **80** | **NA** | **NA** | **80** | **80** |
| E. Zendegani, et al. (281) | **2020** | **Iran** | **CLSI** | **DISK** | **100** | **80** | **NA** | **NA** | **NA** | **80** |
| O. Acer, et al. (282) | **2022** | **Turkey** | **EUCAST** | **MIC** | **80** | **80** | **NA** | **NA** | **NA** | **80** |
| Q. You, et al. (283) | **2023** | **China** | **CLSI** | **MIC** | **89** | **80** | **NA** | **81** | **NA** | **80** |
| X. M. Nie, et al. (284) | **2015** | **China** | **NA** | **DISK** | **101** | **79** | **NA** | **NA** | **NA** | **79** |
| A. Kaur, et al. (285) | **2018** | **India** | **CLSI** | **DISK** | **116** | **79** | **NA** | **NA** | **NA** | **79** |
| M. Kafshnouchi, et al. (286) | **2022** | **Iran** | **CLSI** | **DISK** | **100** | **79** | **NA** | **NA** | **NA** | **79** |
| P. Mohajeri, et al. (287) | **2013** | **Iran** | **CLSI** | **DISK** | **104** | **78** | **NA** | **NA** | **NA** | **78** |
| H. Maspi, et al. (288) | **2016** | **Iran** | **CLSI** | **DISK** | **86** | **78** | **NA** | **NA** | **NA** | **78** |
| M. H. Soudeiha, et al. (289) | **2018** | **Lebanon** | **CLSI** | **DISK** | **100** | **78** | **NA** | **NA** | **78** | **84** |
| S. Levy-Blitchtein, et al. (290) | **2018** | **Peru** | **EUCAST** | **DISK** | **80** | **78** | **NA** | **NA** | **NA** | **78** |
| Y. Chen, et al. (291) | **2018** | **China** | **CLSI** | **MIC** | **101** | **78** | **NA** | **NA** | **NA** | **78** |
| I. Gheorghe, et al. (292) | **2019** | **Romania** | **CLSI** | **DISK** | **93** | **78** | **NA** | **NA** | **NA** | **78** |
| K. B. Said, et al. (293) | **2021** | **Saudi Arabia** | **CLSI** | **MIC** | **82** | **78** | **NA** | **NA** | **NA** | **78** |
| S. A. Khrulnova, et al. (294) | **2020** | **Russia** | **NA** | **NA** | **96** | **77** | **NA** | **NA** | **77** | **NA** |
| Z. Wei, et al. (295) | **2022** | **China** | **CLSI** | **MIC** | **88** | **77** | **NA** | **NA** | **77** | **59** |
| A. M. Somily, et al. (296) | **2012** | **Saudi Arabia** | **CLSI** | **MIC** | **84** | **76** | **65** | **NA** | **NA** | **76** |
| F. K. S. F. De Azevedo, et al. (297) | **2019** | **NA** | **NA** | **NA** | **83** | **76** | **NA** | **NA** | **NA** | **76** |
| I. Morrissey, et al. (298) | **2020** | **Iran** | **CLSI** | **MIC** | **80** | **76** | **NA** | **NA** | **NA** | **76** |
| Y. Li, et al. (299) | **2022** | **China** | **CLSI** | **MIC** | **103** | **76** | **NA** | **NA** | **NA** | **76** |
| S. Direkel, et al. (300) | **2016** | **Turkey** | **CLSI** | **DISK** | **79** | **75** | **NA** | **NA** | **NA** | **75** |
| M. W. Shah, et al. (301) | **2019** | **Saudi Arabia** | **CLSI** | **DISK** | **135** | **75** | **NA** | **NA** | **NA** | **75** |
| Y. J. Kim, et al. (302) | **2012** | **South Korea** | **CLSI** | **MIC** | **75** | **74** | **NA** | **NA** | **NA** | **74** |
| A. Karmostaj, et al. (303) | **2013** | **Iran** | **CLSI** | **DISK** | **84** | **74** | **NA** | **NA** | **NA** | **74** |
| M. Nasrolahei, et al. (304) | **2014** | **Iran** | **NA** | **DISK** | **100** | **74** | **NA** | **NA** | **NA** | **74** |
| M. F. M. Subagdja, et al. (305) | **2022** | **Indonesia** | **CLSI** | **MIC** | **87** | **74** | **NA** | **NA** | **NA** | **74** |
| M. Amin, et al. (306) | **2019** | **Iran** | **CLSI** | **DISK** | **85** | **73** | **NA** | **NA** | **73** | **NA** |
| V. T. T. Selvi, et al. (307) | **2021** | **China** | **CLSI** | **MIC** | **122** | **73** | **NA** | **NA** | **NA** | **73** |
| A. Thampithak, et al. (308) | **2022** | **Thailand** | **CLSI** | **DISK** | **73** | **73** | **NA** | **NA** | **NA** | **73** |
| J. S. Esterly, et al. (309) | **2010** | **US** | **CLSI** | **MIC** | **125** | **72** | **89** | **NA** | **NA** | **72** |
| X. Zhang, et al. (310) | **2015** | **China** | **CLSI** | **DISK** | **111** | **72** | **NA** | **NA** | **NA** | **72** |
| K. K. Ghaima, et al. (311) | **2016** | **Iraq** | **CLSI** | **DISK** | **96** | **72** | **NA** | **NA** | **NA** | **72** |
| R. Ranjbar, et al. (311, 312) | **2020** | **Iran** | **CLSI** | **DISK** | **79** | **72** | **NA** | **NA** | **NA** | **72** |
| A. Wolfensberger, et al. (313) | **2019** | **Switzerland** | **EUCAST** | **DISK** | **178** | **71** | **NA** | **NA** | **71** | **NA** |
| M. S. Siqueira, et al. (314) | **2015** | **Brazil** | **NA** | **NA** | **78** | **70** | **NA** | **NA** | **70** | **NA** |
| V. Whitley, et al. (315) | **2020** | **US** | **CLSI** | **MIC** | **138** | **70** | **NA** | **NA** | **70** | **NA** |
| G. Samonis, et al. (316) | **2010** | **Greece** | **CLSI** | **DISK** | **73** | **69** | **NA** | **NA** | **NA** | **69** |
| G. L. Genteluci, et al. (317) | **2020** | **Brazil** | **CLSI** | **DISK** | **72** | **69** | **NA** | **NA** | **69** | **NA** |
| F. Naeimi Mazraeh, et al. (318) | **2021** | **Iran** | **CLSI** | **DISK** | **70** | **69** | **NA** | **NA** | **NA** | **69** |
| Z. M. Afshar, et al. (319) | **2022** | **Iran** | **CLSI** | **DISK** | **74** | **69** | **NA** | **NA** | **NA** | **69** |
| M. Chakraborty, et al. (320) | **2023** | **India** | **CLSI** | **MIC** | **76** | **69** | **NA** | **NA** | **69** | **NA** |
| H.-R. Lin, et al. (321) | **2016** | **Taiwan** | **CLSI** | **MIC** | **103** | **68** | **NA** | **NA** | **68** | **NA** |
| G. M. Tran, et al. (322) | **2017** | **Vietnam** | **CLSI** | **DISK** | **75** | **68** | **NA** | **75** | **NA** | **68** |
| J. Armalyte, et al. (323) | **2018** | **Iran** | **CLSI** | **DISK** | **171** | **68** | **NA** | **NA** | **68** | **NA** |
| T. Zhang, et al. (324) | **2021** | **China** | **CLSI** | **MIC** | **105** | **68** | **NA** | **NA** | **68** | **NA** |
| S. Selim, et al. (325) | **2022** | **Saudi Arabia** | **CLSI** | **DISK** | **86** | **68** | **NA** | **NA** | **NA** | **68** |
| Z. Li, et al. (326) | **2021** | **China** | **CLSI** | **MIC** | **104** | **67** | **NA** | **NA** | **NA** | **67** |
| M. Anwar, et al. (327) | **2016** | **Pakistan** | **CLSI** | **DISK** | **112** | **66** | **NA** | **NA** | **NA** | **66** |
| Y. K. Park, et al. (328) | **2012** | **South Korea** | **CLSI** | **MIC** | **127** | **65** | **NA** | **NA** | **NA** | **65** |
| K. Sieniawski, et al. (329) | **2013** | **Poland** | **NA** | **MIC** | **152** | **65** | **NA** | **NA** | **NA** | **65** |
| M. Vranic-Ladavac, et al. (330) | **2014** | **Croatia** | **NA** | **MIC** | **185** | **65** | **NA** | **NA** | **NA** | **65** |
| N. Asgin, et al. (331) | **2019** | **Turkey** | **CLSI** | **MIC** | **69** | **65** | **NA** | **NA** | **NA** | **65** |
| A. Japoni-Nejad, et al. (332) | **2021** | **Iran** | **CLSI** | **DISK** | **80** | **65** | **NA** | **NA** | **NA** | **65** |
| M. A. Ababneh, et al. (333) | **2022** | **Jordan** | **CLSI** | **MIC** | **67** | **65** | **NA** | **NA** | **NA** | **65** |
| N. Al-Dabaibah, et al. (334) | **2012** | **Jordan** | **CLSI** | **DISK** | **64** | **64** | **NA** | **NA** | **NA** | **64** |
| L.-J. Zhu, et al. (335) | **2019** | **China** | **CLSI** | **DISK** | **84** | **64** | **NA** | **NA** | **NA** | **64** |
| C. Konca, et al. (336) | **2021** | **Turkey** | **CLSI** | **MIC** | **66** | **64** | **NA** | **64** | **NA** | **64** |
| K. Novovic, et al. (337) | **2023** | **Serbia** | **CLSI** | **MIC** | **64** | **64** | **NA** | **NA** | **NA** | **64** |
| B. Norozi, et al. (338) | **2014** | **Iran** | **NA** | **DISK** | **84** | **63** | **NA** | **NA** | **NA** | **63** |
| O. O. Gundeslioglu, et al. (339) | **2014** | **Turkey** | **NA** | **MIC** | **69** | **63** | **NA** | **NA** | **NA** | **63** |
| G. Jimenez-Guerra, et al. (340) | **2018** | **Spain** | **CLSI** | **MIC** | **85** | **63** | **NA** | **NA** | **NA** | **63** |
| N. Sohrabi, et al. (341) | **2012** | **Iran** | **CLSI** | **DISK** | **100** | **62** | **NA** | **NA** | **NA** | **62** |
| J. Xi, et al. (342) | **2022** | **China** | **CLSI** | **MIC** | **82** | **62** | **NA** | **NA** | **NA** | **62** |
| M. Pourabdollah, et al. (343) | **2023** | **Iran** | **CLSI** | **DISK** | **129** | **62** | **NA** | **NA** | **62** | **NA** |
| M. M. Kock, et al. (344) | **2013** | **South Africa** | **NA** | **MIC** | **97** | **61** | **NA** | **NA** | **NA** | **61** |
| O. Azizi, et al. (345) | **2015** | **Iran** | **NA** | **DISK** | **65** | **61** | **NA** | **NA** | **NA** | **61** |
| H. Ari, et al. (346) | **2019** | **Turkey** | **EUCAST** | **MIC** | **97** | **61** | **NA** | **NA** | **61** | **NA** |
| A. Brink, et al. (347) | **2007** | **South Africa** | **CLSI** | **MIXED** | **190** | **60** | **NA** | **NA** | **NA** | **60** |
| M. Gholami, et al. (348) | **2015** | **Iran** | **NA** | **DISK** | **60** | **60** | **NA** | **NA** | **NA** | **60** |
| S. Agarwal, et al. (349) | **2017** | **India** | **CLSI** | **MIC** | **63** | **60** | **NA** | **NA** | **NA** | **60** |
| S. Pan, et al. (350) | **2018** | **China** | **CLSI** | **DISK** | **61** | **60** | **NA** | **NA** | **NA** | **60** |
| O. Azizi, et al. (351) | **2021** | **Iran** | **CLSI** | **DISK** | **65** | **60** | **NA** | **NA** | **NA** | **60** |
| I. Mumcuoglu, et al. (352) | **2022** | **Turkey** | **EUCAST** | **MIC** | **603** | **60** | **NA** | **NA** | **NA** | **60** |
| B. Hasan, et al. (352) | **2013** | **Pakistan** | **CLSI** | **DISK** | **90** | **59** | **NA** | **NA** | **NA** | **59** |
| D. W. Wareham, et al. (353) | **2020** | **UK** | **CLSI** | **MIXED** | **66** | **59** | **NA** | **NA** | **59** | **NA** |
| N. Van An, et al. (354) | **2023** | **Vietnam** | **CLSI** | **MIXED** | **97** | **59** | **NA** | **NA** | **NA** | **59** |
| A. Y. Peleg, et al. (355) | **2006** | **Australia** | **NA** | **MIC** | **90** | **58** | **NA** | **NA** | **NA** | **58** |
| T. D. Van, et al. (356) | **2014** | **Vietnam** | **NA** | **MIC** | **66** | **58** | **NA** | **NA** | **NA** | **58** |
| A. Ramette, et al. (357) | **2018** | **Switzerland** | **EUCAST** | **NA** | **632** | **58** | **NA** | **NA** | **58** | **NA** |
| R. Aljindan, et al. (358) | **2018** | **Saudi Arabia** | **CLSI** | **MIXED** | **60** | **58** | **NA** | **NA** | **NA** | **58** |
| S. J. Nigro, et al. (359) | **2018** | **Australia** | **CLSI** | **DISK** | **90** | **58** | **NA** | **NA** | **NA** | **58** |
| A. Massik, et al. (360) | **2021** | **Morocco** | **CLSI** | **DISK** | **59** | **58** | **NA** | **NA** | **58** | **NA** |
| X. Jin, et al. (361) | **2021** | **China** | **CLSI** | **DISK** | **93** | **58** | **NA** | **NA** | **58** | **NA** |
| Z. Elnasser, et al. (362) | **2021** | **Jordan** | **CLSI** | **MIC** | **150** | **58** | **NA** | **NA** | **NA** | **58** |
| D. Depka, et al. (363) | **2022** | **Poland** | **EUCAST** | **MIC** | **58** | **58** | **NA** | **NA** | **NA** | **58** |
| H. S. I. Martins, et al. (364) | **2014** | **Brazil** | **NA** | **DISK** | **64** | **57** | **NA** | **NA** | **NA** | **57** |
| R. El-Sokkary, et al. (365) | **2021** | **NA** | **CLSI** | **MIXED** | **59** | **57** | **NA** | **NA** | **NA** | **57** |
| A. Rahman, et al. (366) | **2022** | **Bangladesh** | **CLSI** | **DISK** | **63** | **57** | **NA** | **NA** | **NA** | **57** |
| M. M. Feizabadi, et al. (367) | **2008** | **Iran** | **CLSI** | **DISK** | **108** | **56** | **NA** | **NA** | **NA** | **56** |
| A. Peymani, et al. (368) | **2011** | **Iran** | **CLSI** | **DISK** | **100** | **56** | **NA** | **NA** | **NA** | **56** |
| G. F. Viana, et al. (369) | **2013** | **Brazil** | **CLSI** | **MIXED** | **121** | **56** | **NA** | **NA** | **56** | **NA** |
| A. Slavcovici, et al. (370) | **2015** | **Romania** | **CLSI** | **DISK** | **66** | **56** | **NA** | **NA** | **56** | **NA** |
| L. Al-Hassan, et al. (371) | **2019** | **Egypt** | **CLSI** | **MIC** | **59** | **55** | **NA** | **NA** | **55** | **NA** |
| P. J. Turner, et al. (372) | **2003** | **NA** | **CLSI** | **MIC** | **490** | **54** | **NA** | **NA** | **NA** | **54** |
| Y. Cai, et al. (373) | **2010** | **China** | **CLSI** | **MIC** | **70** | **54** | **NA** | **NA** | **NA** | **54** |
| S. Dally, et al. (374) | **2013** | **Germany** | **EUCAST** | **MIC** | **60** | **54** | **NA** | **NA** | **NA** | **54** |
| P. Bocanegra-Ibarias, et al. (375) | **2015** | **Mexico** | **NA** | **MIC** | **152** | **54** | **NA** | **NA** | **NA** | **54** |
| S. S. Kara, et al. (376) | **2019** | **Turkey** | **NA** | **NA** | **58** | **54** | **NA** | **NA** | **54** | **NA** |
| F. Pei, et al. (377) | **2013** | **China** | **CLSI** | **DISK** | **111** | **53** | **NA** | **NA** | **NA** | **53** |
| H. Chen, et al. (378) | **2015** | **China** | **NA** | **MIC** | **100** | **53** | **NA** | **NA** | **NA** | **53** |
| Y. Yang, et al. (379) | **2015** | **China** | **CLSI** | **DISK** | **332** | **53** | **NA** | **NA** | **NA** | **53** |
| N. Y. Nojookambari, et al. (380) | **2021** | **Iran** | **CLSI** | **DISK** | **60** | **53** | **NA** | **NA** | **NA** | **53** |
| N. Yousefi Nojookambari, et al. (380) | **2021** | **Iran** | **CLSI** | **DISK** | **60** | **53** | **NA** | **NA** | **NA** | **53** |
| V. Gogou, et al. (381) | **2011** | **Greece** | **CLSI** | **MIC** | **94** | **52** | **NA** | **NA** | **NA** | **52** |
| H. R. Tawfeeq, et al. (382) | **2020** | **Iraq** | **CLSI** | **DISK** | **54** | **52** | **NA** | **NA** | **NA** | **52** |
| J. Li, et al. (383) | **2020** | **China** | **CLSI** | **MIC** | **57** | **52** | **NA** | **NA** | **NA** | **52** |
| C. Lasarte-Monterrubio, et al. (384) | **2021** | **Spain** | **CLSI** | **MIC** | **89** | **52** | **NA** | **NA** | **NA** | **52** |
| X. Bian, et al. (385) | **2021** | **China** | **CLSI** | **MIC** | **64** | **52** | **52** | **NA** | **NA** | **52** |
| S. Shabazi, et al. (386) | **2022** | **Iran** | **CLSI** | **DISK** | **70** | **52** | **NA** | **NA** | **NA** | **52** |
| P. F. Hou, et al. (387) | **2012** | **China** | **CLSI** | **MIC** | **80** | **51** | **NA** | **NA** | **NA** | **51** |
| M. Cerezales, et al. (388) | **2019** | **Bolivia** | **EUCAST** | **MIC** | **95** | **51** | **NA** | **NA** | **NA** | **51** |
| N. Karah, et al. (389) | **2020** | **Pakistan** | **CLSI** | **DISK** | **52** | **51** | **NA** | **NA** | **51** | **NA** |
| N. G. Banoub, et al. (390) | **2021** | **Egypt** | **CLSI** | **DISK** | **51** | **51** | **51** | **NA** | **NA** | **51** |
| T. Chen, et al. (391) | **2021** | **China** | **CLSI** | **MIC** | **54** | **51** | **NA** | **NA** | **NA** | **51** |
| K. C. Ashuthosh, et al. (392) | **2020** | **India** | **NA** | **MIC** | **100** | **50** | **NA** | **NA** | **NA** | **50** |
| N. Bankan, et al. (393) | **2021** | **India** | **CLSI** | **NA** | **100** | **50** | **NA** | **NA** | **NA** | **50** |
| R. U. Karthika, et al. (394) | **2009** | **India** | **CLSI** | **DISK** | **55** | **49** | **NA** | **NA** | **NA** | **49** |
| A. K. Mostachio, et al. (395) | **2012** | **Brazil** | **CLSI** | **MIC** | **49** | **49** | **NA** | **NA** | **NA** | **49** |
| T. Tada, et al. (396) | **2014** | **Japan** | **NA** | **MIC** | **49** | **49** | **NA** | **NA** | **NA** | **49** |
| S. A. Girija, et al. (397) | **2019** | **India** | **CLSI** | **DISK** | **73** | **49** | **45** | **NA** | **NA** | **49** |
| S. Girija As, et al. (398) | **2019** | **India** | **CLSI** | **DISK** | **73** | **49** | **45** | **NA** | **NA** | **49** |
| A. Fatima, et al. (399) | **2022** | **Pakistan** | **CLSI** | **DISK** | **61** | **49** | **NA** | **NA** | **NA** | **49** |
| P. Santoso, et al. (400) | **2022** | **Indonesia** | **CLSI** | **MIC** | **58** | **49** | **NA** | **NA** | **NA** | **49** |
| R. H. Shayea, et al. (401) | **2022** | **Iraq** | **CLSI** | **DISK** | **75** | **49** | **NA** | **NA** | **NA** | **49** |
| N. Ceyhan-Guvensen, et al. (402) | **2017** | **Turkey** | **EUCAST** | **MIC** | **50** | **48** | **NA** | **NA** | **NA** | **48** |
| F. Tarafdar, et al. (403) | **2020** | **Iran** | **CLSI** | **DISK** | **50** | **48** | **NA** | **NA** | **NA** | **48** |
| C.-K. Kim, et al. (404) | **2010** | **South Korea** | **CLSI** | **MIC** | **47** | **47** | **NA** | **NA** | **NA** | **47** |
| B. Abdalhamid, et al. (405) | **2014** | **Saudi Arabia** | **CLSI** | **MIC** | **141** | **47** | **NA** | **NA** | **NA** | **47** |
| H. Khalili, et al. (406) | **2018** | **Iran** | **CLSI** | **MIXED** | **47** | **47** | **NA** | **NA** | **NA** | **47** |
| M. A. Dehbalaei, et al. (407) | **2018** | **Iran** | **CLSI** | **DISK** | **55** | **47** | **NA** | **NA** | **NA** | **47** |
| S. Valadan Tahbaz, et al. (408) | **2019** | **Iran** | **CLSI** | **DISK** | **47** | **47** | **NA** | **NA** | **NA** | **47** |
| M. J. C. Noyal, et al. (409) | **2009** | **India** | **CLSI** | **DISK** | **78** | **46** | **NA** | **NA** | **NA** | **46** |
| Y. B. Long, et al. (410) | **2009** | **Australia** | **NA** | **MIC** | **53** | **46** | **NA** | **NA** | **NA** | **46** |
| F. Fang, et al. (411) | **2016** | **China** | **CLSI** | **MIC** | **205** | **46** | **NA** | **NA** | **NA** | **46** |
| Q. Chen, et al. (412) | **2018** | **China** | **CLSI** | **MIC** | **87** | **46** | **NA** | **NA** | **NA** | **46** |
| A. Nazir(413) | **2019** | **India** | **CLSI** | **MIC** | **48** | **46** | **NA** | **NA** | **NA** | **46** |
| Y. Huang, et al. (414) | **2019** | **China** | **CLSI** | **NA** | **110** | **46** | **NA** | **NA** | **NA** | **46** |
| V. Thao Nguyen, et al. (415) | **2020** | **South Korea** | **CLSI** | **DISK** | **73** | **46** | **NA** | **NA** | **NA** | **46** |
| H. S. Rusul, et al. (416) | **2022** | **Iraq** | **CLSI** | **DISK** | **65** | **46** | **NA** | **NA** | **NA** | **46** |
| Z. Ma, et al. (417) | **2015** | **China** | **NA** | **DISK** | **89** | **45** | **NA** | **NA** | **45** | **NA** |
| L. Han, et al. (418) | **2017** | **China** | **CLSI** | **MIXED** | **45** | **45** | **NA** | **NA** | **NA** | **45** |
| Z. Babaie, et al. (419) | **2020** | **Iran** | **NA** | **DISK** | **50** | **45** | **NA** | **NA** | **NA** | **45** |
| M. H. Namaei, et al. (420) | **2021** | **Iran** | **CLSI** | **DISK** | **50** | **45** | **NA** | **NA** | **NA** | **45** |
| N. J. Ahmed, et al. (421) | **2021** | **India** | **NA** | **NA** | **70** | **45** | **NA** | **NA** | **NA** | **45** |
| W. P. Ar, et al. (422) | **2021** | **Myanmar** | **CLSI** | **MIC** | **72** | **45** | **NA** | **NA** | **NA** | **45** |
| R. Farzana, et al. (423) | **2022** | **Bangladesh** | **EUCAST** | **DISK** | **49** | **45** | **NA** | **NA** | **NA** | **45** |
| M. T. S. Al-Ouqaili, et al. (424) | **2018** | **Iraq** | **NA** | **MIXED** | **44** | **44** | **NA** | **NA** | **44** | **NA** |
| L. L. Al-Hassan, et al. (425) | **2020** | **Egypt** | **CLSI** | **MIC** | **54** | **44** | **NA** | **54** | **NA** | **44** |
| Y. Qiu, et al. (426) | **2021** | **China** | **CLSI** | **MIXED** | **139** | **44** | **NA** | **NA** | **44** | **NA** |
| R. Zarrilli, et al. (427) | **2004** | **Italy** | **CLSI** | **DISK** | **131** | **43** | **NA** | **NA** | **43** | **NA** |
| A. A. Khodier, et al. (428) | **2020** | **Egypt** | **CLSI** | **DISK** | **49** | **43** | **NA** | **NA** | **43** | **NA** |
| A.-L. Golli, et al. (429) | **2022** | **Romania** | **CLSI** | **MIC** | **45** | **43** | **NA** | **NA** | **NA** | **43** |
| B. M. Forde, et al. (430) | **2022** | **Italy** | **CLSI** | **MIC** | **45** | **43** | **NA** | **NA** | **43** | **NA** |
| G. Mahajan, et al. (431) | **2010** | **India** | **CLSI** | **DISK** | **132** | **42** | **NA** | **NA** | **NA** | **42** |
| S.-S. Lean, et al. (432) | **2014** | **Malaysia** | **NA** | **MIC** | **54** | **42** | **NA** | **NA** | **NA** | **42** |
| M. Jajoo, et al. (433) | **2018** | **India** | **CLSI** | **MIC** | **46** | **42** | **NA** | **NA** | **42** | **NA** |
| A. S. Shahari, et al. (434) | **2019** | **Malaysia** | **CLSI** | **DISK** | **54** | **42** | **NA** | **NA** | **NA** | **42** |
| Y. Zhang, et al. (435) | **2020** | **China** | **CLSI** | **MIC** | **52** | **42** | **NA** | **NA** | **42** | **NA** |
| M. Kar, et al. (436) | **2023** | **India** | **CLSI** | **DISK** | **45** | **42** | **NA** | **NA** | **42** | **NA** |
| S. S. Jean, et al. (437) | **2009** | **Taiwan** | **CLSI** | **MIC** | **167** | **41** | **NA** | **NA** | **NA** | **41** |
| N. A. Al-Sweih, et al. (438) | **2012** | **Kuwait** | **CLSI** | **MIC** | **94** | **41** | **NA** | **NA** | **NA** | **41** |
| M. C. Jara, et al. (439) | **2021** | **Brazil** | **CLSI** | **MIC** | **46** | **41** | **NA** | **NA** | **41** | **NA** |
| A. Maleki, et al. (440) | **2022** | **Iran** | **CLSI** | **MIC** | **60** | **41** | **NA** | **NA** | **NA** | **41** |
| O. Ergonul, et al. (441) | **2022** | **Turkey** | **NA** | **NA** | **53** | **41** | **NA** | **NA** | **41** | **NA** |
| S.-X. Dong, et al. (442) | **2012** | **Taiwan** | **CLSI** | **MIC** | **100** | **40** | **44** | **NA** | **NA** | **40** |
| Ghajav, et al. (443) | **2015** | **Iran** | **NA** | **DISK** | **43** | **40** | **NA** | **NA** | **NA** | **40** |
| S. T. M. Tolba, et al. (444) | **2019** | **Egypt** | **CLSI** | **DISK** | **45** | **40** | **NA** | **NA** | **40** | **NA** |
| A. Y. Adjei, et al. (445) | **2021** | **Ghana** | **CLSI** | **MIC** | **40** | **40** | **NA** | **NA** | **NA** | **40** |
| L. Al-Hassan, et al. (446) | **2021** | **Sudan** | **EUCAST** | **MIC** | **42** | **40** | **NA** | **NA** | **NA** | **40** |
| M. G. Donadu, et al. (447) | **2021** | **Italy** | **EUCAST** | **DISK** | **62** | **40** | **NA** | **NA** | **NA** | **40** |
| N. M. Joseph, et al. (448) | **2011** | **India** | **CLSI** | **DISK** | **56** | **39** | **NA** | **NA** | **NA** | **39** |
| M. Fouad, et al. (449) | **2013** | **Egypt** | **CLSI** | **NA** | **39** | **39** | **NA** | **NA** | **NA** | **39** |
| M. Teresa Reguero, et al. (450) | **2013** | **Colombia** | **CLSI** | **DISK** | **51** | **39** | **NA** | **NA** | **NA** | **39** |
| Y. J. Li, et al. (451) | **2013** | **China** | **CLSI** | **DISK** | **42** | **39** | **NA** | **NA** | **NA** | **39** |
| G. Makke, et al. (452) | **2020** | **Lebanon** | **CLSI** | **MIC** | **41** | **39** | **NA** | **NA** | **NA** | **39** |
| J. Wang, et al. (453) | **2020** | **China** | **CLSI** | **MIC** | **49** | **39** | **NA** | **NA** | **39** | **NA** |
| M. F. El-Badawy, et al. (454) | **2021** | **Egypt** | **CLSI** | **MIC** | **40** | **39** | **NA** | **NA** | **NA** | **39** |
| M. D. Alcantar-Curiel, et al. (455) | **2023** | **Mexico** | **CLSI** | **MIC** | **39** | **39** | **39** | **NA** | **NA** | **39** |
| M. Purohit, et al. (456) | **2012** | **India** | **CLSI** | **DISK** | **130** | **38** | **NA** | **NA** | **NA** | **38** |
| C. G. Carvalhaes, et al. (457) | **2013** | **Brazil** | **CLSI** | **MIC** | **41** | **38** | **NA** | **NA** | **NA** | **38** |
| D. M. Khan, et al. (458) | **2018** | **India** | **CLSI** | **DISK** | **70** | **38** | **NA** | **NA** | **NA** | **38** |
| M. Rahman, et al. (459) | **2018** | **India** | **CLSI** | **MIC** | **106** | **38** | **NA** | **NA** | **38** | **NA** |
| B. Basatian-Tashkan, et al. (460) | **2020** | **Iran** | **CLSI** | **DISK** | **60** | **38** | **NA** | **NA** | **NA** | **38** |
| M. Hazhirkamal, et al. (461) | **2021** | **Iran** | **CLSI** | **DISK** | **40** | **38** | **NA** | **NA** | **NA** | **38** |
| S. H. Mostafa, et al. (462) | **2022** | **Egypt** | **CLSI** | **DISK** | **55** | **38** | **NA** | **NA** | **NA** | **38** |
| F. Akin, et al. (463) | **2018** | **Turkey** | **CLSI** | **MIC** | **60** | **37** | **NA** | **NA** | **NA** | **37** |
| V. Kondratiuk, et al. (464) | **2021** | **Ukraine** | **EUCAST** | **DISK** | **52** | **37** | **NA** | **NA** | **37** | **NA** |
| A. Balkhair, et al. (465) | **2023** | **Oman** | **CLSI** | **MIC** | **46** | **37** | **NA** | **NA** | **37** | **NA** |
| S. Park, et al. (466) | **2013** | **South Korea** | **CLSI** | **MIC** | **36** | **36** | **NA** | **NA** | **NA** | **36** |
| A. P. Petrova, et al. (467) | **2017** | **Bulgaria** | **CLSI** | **DISK** | **43** | **36** | **NA** | **NA** | **NA** | **36** |
| S. A. S. Girija, et al. (468) | **2018** | **India** | **CLSI** | **DISK** | **73** | **36** | **33** | **NA** | **NA** | **36** |
| C. Tellapragada, et al. (469) | **2020** | **Sweden** | **CLSI** | **MIC** | **80** | **36** | **NA** | **NA** | **NA** | **36** |
| M. Kumari, et al. (470) | **2021** | **India** | **CLSI** | **DISK** | **55** | **36** | **NA** | **NA** | **NA** | **36** |
| C. R. V. Kiffer, et al. (471) | **2004** | **NA** | **CLSI** | **MIC** | **128** | **35** | **NA** | **NA** | **NA** | **35** |
| X. Wang, et al. (472) | **2013** | **China** | **CLSI** | **MIC** | **67** | **35** | **NA** | **NA** | **NA** | **35** |
| H. A. Kadhom, et al. (473) | **2022** | **Iraq** | **CLSI** | **DISK** | **50** | **35** | **32** | **NA** | **NA** | **35** |
| S. Pourajam, et al. (474) | **2022** | **Iran** | **CLSI** | **DISK** | **35** | **35** | **NA** | **NA** | **NA** | **35** |
| I. Franolic-Kukina, et al. (475) | **2011** | **France** | **CLSI** | **MIC** | **34** | **34** | **NA** | **NA** | **NA** | **34** |
| A. G. Ozseven, et al. (476) | **2012** | **Turkey** | **CLSI** | **DISK** | **34** | **34** | **NA** | **NA** | **NA** | **34** |
| Z. Y√ºr√ºken, et al. (477) | **2016** | **Turkey** | **CLSI** | **DISK** | **48** | **34** | **NA** | **NA** | **34** | **34** |
| M. Sadeghi-Haddad-Zavareh, et al. (478) | **2018** | **Iran** | **CLSI** | **MIC** | **50** | **34** | **NA** | **NA** | **NA** | **34** |
| R. Wasfi, et al. (479) | **2021** | **Egypt** | **CLSI** | **MIC** | **48** | **34** | **NA** | **NA** | **34** | **NA** |
| S. E. R. Mohamed, et al. (480) | **2022** | **Sudan** | **NA** | **MIC** | **40** | **34** | **NA** | **NA** | **NA** | **34** |
| Y. Gu, et al. (481) | **2022** | **China** | **CLSI** | **MIC** | **47** | **34** | **NA** | **NA** | **NA** | **34** |
| B. Bedenic, et al. (482) | **2023** | **Croatia** | **CLSI** | **DISK** | **34** | **34** | **NA** | **NA** | **NA** | **34** |
| X.-L. Wei, et al. (483) | **2023** | **China** | **CLSI** | **MIC** | **82** | **34** | **NA** | **NA** | **NA** | **34** |
| J. Huang, et al. (484) | **2013** | **China** | **CLSI** | **NA** | **38** | **33** | **NA** | **NA** | **NA** | **33** |
| F. Akrami, et al. (485) | **2017** | **Iran** | **CLSI** | **DISK** | **35** | **33** | **NA** | **NA** | **NA** | **33** |
| M. Cerezales, et al. (486) | **2018** | **Bolivia** | **EUCAST** | **DISK** | **36** | **33** | **NA** | **NA** | **NA** | **33** |
| √ñ. Koca(487) (487) | **2019** | **Turkey** | **EUCAST** | **MIC** | **37** | **33** | **NA** | **NA** | **NA** | **33** |
| T. Y. Li, et al. (488) | **2019** | **Taiwan** | **CLSI** | **MIC** | **53** | **33** | **33** | **NA** | **NA** | **33** |
| P. Chhatwal, et al. (489) | **2021** | **Germany** | **EUCAST** | **MIC** | **40** | **33** | **NA** | **NA** | **NA** | **33** |
| P. R. Hsueh, et al. (490) | **2001** | **Taiwan** | **CLSI** | **MIC** | **155** | **32** | **NA** | **NA** | **NA** | **32** |
| W.-H. Sheng, et al. (491) | **2011** | **Taiwan** | **CLSI** | **MIC** | **32** | **32** | **NA** | **NA** | **NA** | **32** |
| I. N. Hakyemez, et al. (492) | **2013** | **Turkey** | **CLSI** | **MIC** | **56** | **32** | **NA** | **NA** | **NA** | **32** |
| J. Akhtar, et al. (493) | **2018** | **Pakistan** | **CLSI** | **DISK** | **32** | **32** | **NA** | **NA** | **NA** | **32** |
| E. L. Fonseca, et al. (494) | **2020** | **Brazil** | **CLSI** | **MIC** | **32** | **32** | **NA** | **NA** | **NA** | **32** |
| C. Ozkul, et al. (495) | **2021** | **Turkey** | **EUCAST** | **DISK** | **44** | **32** | **NA** | **NA** | **NA** | **32** |
| M. K. Jayanthi, et al. (496) | **2021** | **India** | **CLSI** | **DISK** | **50** | **32** | **NA** | **NA** | **NA** | **32** |
| J. Y. Sung, et al. (497) | **2008** | **South Korea** | **CLSI** | **DISK** | **31** | **31** | **NA** | **NA** | **NA** | **31** |
| E. S. Cetin, et al. (498) | **2009** | **Turkey** | **CLSI** | **DISK** | **66** | **31** | **NA** | **NA** | **NA** | **31** |
| K. Asadollahi, et al. (499) | **2011** | **Iran** | **CLSI** | **MIC** | **100** | **31** | **NA** | **NA** | **NA** | **31** |
| F. I. Sonbol, et al. (500) | **2019** | **Egypt** | **CLSI** | **DISK** | **69** | **31** | **NA** | **NA** | **NA** | **31** |
| S. Chusri, et al. (501) | **2019** | **Thailand** | **CLSI** | **MIC** | **60** | **31** | **NA** | **NA** | **31** | **NA** |
| B. Sobouti, et al. (502) | **2020** | **Iran** | **CLSI** | **DISK** | **62** | **31** | **NA** | **NA** | **NA** | **31** |
| F. R. Zaniani, et al. (503) | **2022** | **Iran** | **CLSI** | **DISK** | **33** | **31** | **NA** | **NA** | **NA** | **31** |
| H. G. A. Alshami, et al. (504) | **2022** | **Iran** | **CLSI** | **DISK** | **31** | **31** | **NA** | **NA** | **NA** | **31** |
| S. S. Jean, et al. (505) | **2002** | **Taiwan** | **CLSI** | **DISK** | **55** | **30** | **NA** | **NA** | **NA** | **30** |
| J. F. Turton, et al. (506) | **2004** | **UK** | **NA** | **MIC** | **45** | **30** | **NA** | **NA** | **NA** | **30** |
| S. Mahdian, et al. (507) | **2015** | **Iran** | **NA** | **DISK** | **37** | **30** | **NA** | **NA** | **NA** | **30** |
| R. A. Ramadan, et al. (508) | **2018** | **Egypt** | **CLSI** | **MIC** | **50** | **30** | **NA** | **NA** | **30** | **NA** |
| A. H. Uc-Cachon, et al. (509) | **2019** | **Mexico** | **CLSI** | **MIC** | **53** | **30** | **NA** | **NA** | **NA** | **30** |
| M. F. El-Badawy, et al. (510) | **2019** | **Saudi Arabia** | **CLSI** | **MIC** | **32** | **30** | **NA** | **NA** | **NA** | **30** |
| X. Niu, et al. (511) | **2019** | **China** | **CLSI** | **DISK** | **55** | **30** | **NA** | **NA** | **NA** | **30** |
| K. Paramita, et al. (512) | **2020** | **Indonesia** | **CLSI** | **MIC** | **55** | **30** | **NA** | **NA** | **30** | **NA** |
| A. A. Al-Sultan(513) | **2021** | **Saudi Arabia** | **CLSI** | **MIC** | **37** | **30** | **NA** | **NA** | **30** | **31** |
| B. G. Cabral, et al. (514) | **2021** | **Brazil** | **CLSI** | **MIC** | **49** | **30** | **NA** | **NA** | **NA** | **30** |
| S. N. Peerayeh, et al. (515) | **2015** | **Iran** | **NA** | **DISK** | **30** | **29** | **NA** | **NA** | **NA** | **29** |
| S. Chatterjee, et al. (516) | **2016** | **India** | **CLSI** | **MIC** | **49** | **29** | **NA** | **NA** | **NA** | **29** |
| I. Bado, et al. (517) | **2018** | **India** | **EUCAST** | **MIC** | **29** | **29** | **NA** | **NA** | **NA** | **29** |
| J. Yeongdon, et al. (518) | **2021** | **China** | **CLSI** | **MIC** | **34** | **29** | **NA** | **NA** | **29** | **NA** |
| N. Shi, et al. (519) | **2022** | **China** | **CLSI** | **MIC** | **38** | **29** | **NA** | **NA** | **29** | **NA** |
| E. L. Fonseca, et al. (520) | **2013** | **Brazil** | **CLSI** | **DISK** | **28** | **28** | **NA** | **20** | **NA** | **28** |
| L. Fan, et al. (521) | **2018** | **China** | **CLSI** | **MIC** | **60** | **28** | **NA** | **NA** | **NA** | **28** |
| M. M. Hamza, et al. (522) | **2020** | **Iraq** | **CLSI** | **MIXED** | **40** | **28** | **NA** | **NA** | **NA** | **28** |
| M. Osman, et al. (523) | **2020** | **Lebanon** | **EUCAST** | **DISK** | **28** | **28** | **NA** | **NA** | **NA** | **28** |
| A. Rizvi, et al. (524) | **2022** | **Pakistan** | **CLSI** | **DISK** | **47** | **28** | **NA** | **NA** | **NA** | **28** |
| S. Mahich, et al. (525) | **2022** | **India** | **CLSI** | **DISK** | **33** | **28** | **NA** | **17** | **NA** | **28** |
| S. D. Kotsakis, et al. (526) | **2013** | **Greece** | **EUCAST** | **MIC** | **28** | **27** | **NA** | **NA** | **NA** | **27** |
| P. Vu Dinh, et al. (527) | **2017** | **Vietnam** | **CLSI** | **DISK** | **32** | **27** | **NA** | **NA** | **27** | **NA** |
| M. Jain, et al. (528) | **2019** | **India** | **CLSI** | **MIC** | **28** | **27** | **NA** | **NA** | **27** | **NA** |
| R. V. Caldart, et al. (529) | **2019** | **Brazil** | **CLSI** | **DISK** | **101** | **27** | **NA** | **NA** | **27** | **NA** |
| Q. Xu, et al. (530) | **2020** | **China** | **CLSI** | **MIC** | **28** | **27** | **NA** | **NA** | **27** | **NA** |
| A. S. Jaloot, et al. (531) | **2021** | **Iraq** | **CLSI** | **MIC** | **31** | **27** | **NA** | **NA** | **NA** | **27** |
| M. Azad, et al. (532) | **2021** | **India** | **CLSI** | **DISK** | **69** | **27** | **NA** | **NA** | **NA** | **27** |
| H. U. Altun, et al. (533) | **2014** | **Turkey** | **CLSI** | **MIC** | **30** | **26** | **NA** | **NA** | **NA** | **26** |
| Y. J. Choe, et al. (534) | **2019** | **South Korea** | **CLSI** | **NA** | **74** | **26** | **NA** | **NA** | **26** | **NA** |
| M. Rahbar, et al. (535) | **2008** | **Iran** | **CLSI** | **DISK** | **65** | **25** | **NA** | **NA** | **NA** | **25** |
| A. H. Asghar, et al. (536) | **2009** | **Saudi Arabia** | **CLSI** | **NA** | **123** | **25** | **NA** | **NA** | **NA** | **25** |
| M. Gilani, et al. (537) | **2015** | **NA** | **NA** | **DISK** | **30** | **25** | **NA** | **NA** | **NA** | **25** |
| C. Vicentini, et al. (538) | **2020** | **US** | **CLSI** | **MIC** | **37** | **25** | **NA** | **NA** | **25** | **NA** |
| L. P. Nguyen, et al. (539) | **2021** | **South Korea** | **CLSI** | **MIC** | **25** | **25** | **NA** | **NA** | **NA** | **25** |
| S. Mohsin, et al. (540) | **2021** | **Iraq** | **CLSI** | **DISK** | **74** | **25** | **NA** | **NA** | **NA** | **25** |
| K. F. Schuertz, et al. (541) | **2018** | **Brazil** | **CLSI** | **MIC** | **26** | **24** | **NA** | **NA** | **NA** | **24** |
| M. Tamburro, et al. (542) | **2019** | **Italy** | **NA** | **MIC** | **24** | **24** | **NA** | **NA** | **24** | **NA** |
| A. Iregui, et al. (543) | **2020** | **US** | **CLSI** | **MIC** | **46** | **24** | **NA** | **NA** | **NA** | **24** |
| A. Iregui, et al. (544) | **2021** | **US** | **CLSI** | **MIC** | **47** | **24** | **NA** | **NA** | **NA** | **24** |
| A. A. Shah, et al. (545) | **2022** | **Pakistan** | **CLSI** | **DISK** | **100** | **24** | **NA** | **NA** | **24** | **NA** |
| S. J. Mun, et al. (546) | **2022** | **South Korea** | **CLSI** | **MIC** | **34** | **24** | **NA** | **NA** | **24** | **NA** |
| Y. K. Park, et al. (547) | **2010** | **South Korea** | **CLSI** | **MIC** | **52** | **23** | **NA** | **NA** | **NA** | **23** |
| K. S. Shin, et al. (548) | **2011** | **South Korea** | **CLSI** | **MIC** | **23** | **23** | **NA** | **NA** | **NA** | **23** |
| N. Mathlouthi, et al. (549) | **2015** | **Libya** | **NA** | **DISK** | **25** | **23** | **NA** | **NA** | **NA** | **23** |
| U. Singkham-In, et al. (550) | **2018** | **Thailand** | **CLSI** | **DISK** | **23** | **23** | **NA** | **NA** | **NA** | **23** |
| F. Licata, et al. (551) | **2020** | **Italy** | **EUCAST** | **MIC** | **91** | **23** | **NA** | **15** | **NA** | **23** |
| H. El Hafa, et al. (552) | **2020** | **Morocco** | **CLSI** | **MIC** | **81** | **23** | **NA** | **NA** | **23** | **NA** |
| B. Cherukuri(553) | **2021** | **India** | **NA** | **NA** | **34** | **23** | **NA** | **NA** | **NA** | **23** |
| S. Japoni, et al. (554) | **2011** | **Iran** | **NA** | **MIC** | **79** | **22** | **NA** | **NA** | **NA** | **22** |
| A. Chmielarczyk, et al. (554) | **2012** | **Poland** | **EUCAST** | **MIC** | **28** | **22** | **NA** | **NA** | **NA** | **22** |
| M. Dehghani, et al. (555) | **2012** | **Iran** | **CLSI** | **DISK** | **50** | **22** | **NA** | **NA** | **NA** | **22** |
| C. Moon, et al. (556) | **2013** | **South Korea** | **NA** | **MIC** | **40** | **22** | **NA** | **NA** | **22** | **NA** |
| R. Mirnejad, et al. (557) | **2013** | **Iran** | **CLSI** | **DISK** | **50** | **22** | **NA** | **NA** | **NA** | **22** |
| Y. Pan, et al. (558) | **2018** | **Thailand** | **CLSI** | **MIC** | **110** | **22** | **NA** | **NA** | **22** | **NA** |
| M. Giannella, et al. (559) | **2019** | **Italy** | **EUCAST** | **MIC** | **33** | **22** | **NA** | **NA** | **22** | **NA** |
| S. H. Radhi, et al. (560) | **2019** | **Iraq** | **CLSI** | **DISK** | **30** | **22** | **NA** | **NA** | **22** | **NA** |
| I. R. Ali, et al. (561) | **2021** | **Iraq** | **CLSI** | **DISK** | **26** | **22** | **NA** | **NA** | **NA** | **22** |
| H.-N. Wu, et al. (562) | **2022** | **China** | **CLSI** | **MIC** | **25** | **22** | **NA** | **NA** | **NA** | **22** |
| M. K. Devian, et al. (563) | **2022** | **Indonesia** | **NA** | **NA** | **37** | **22** | **NA** | **NA** | **NA** | **22** |
| S. Sharma, et al. (564) | **2022** | **India** | **CLSI** | **MIC** | **30** | **22** | **NA** | **NA** | **22** | **NA** |
| G. Metan, et al. (565) | **2020** | **Turkey** | **EUCAST** | **MIC** | **21** | **21** | **NA** | **NA** | **NA** | **21** |
| S. B. De Freitas, et al. (566) | **2020** | **Brazil** | **CLSI** | **DISK** | **22** | **21** | **NA** | **NA** | **NA** | **21** |
| C. Kaur, et al. (567) | **2022** | **India** | **CLSI** | **MIC** | **24** | **21** | **NA** | **NA** | **NA** | **21** |
| K. Slimene, et al. (568) | **2023** | **Libya** | **CLSI** | **MIC** | **21** | **21** | **NA** | **NA** | **NA** | **21** |
| P. D. Mugnier, et al. (569) | **2010** | **NA** | **CLSI** | **MIXED** | **20** | **20** | **NA** | **NA** | **NA** | **20** |
| Y.-C. Lin, et al. (570) | **2010** | **Taiwan** | **CLSI** | **MIC** | **53** | **20** | **NA** | **NA** | **NA** | **20** |
| S.-C. Lee, et al. (571) | **2011** | **Taiwan** | **CLSI** | **MIC** | **32** | **20** | **NA** | **24** | **NA** | **20** |
| H.-A. Terzi, et al. (572) | **2016** | **Turkey** | **NA** | **MIC** | **25** | **20** | **17** | **NA** | **NA** | **20** |
| N. Tafreshi, et al. (573) | **2019** | **Iran** | **CLSI** | **MIC** | **84** | **20** | **NA** | **NA** | **NA** | **20** |
| D. Ic-Pavlovic, et al. (574) | **2020** | **Croatia** | **CLSI** | **MIC** | **23** | **20** | **NA** | **NA** | **NA** | **20** |
| H. Q. Raheem, et al. (575) | **2020** | **Iraq** |  | **MIC** | **20** | **20** | **NA** | **NA** | **NA** | **20** |
| S. M. Kadom, et al. (576) | **2020** | **Iraq** | **CLSI** | **MIC** | **25** | **20** | **NA** | **NA** | **NA** | **20** |
| M. M. Zafer, et al. (577) | **2021** | **Egypt** | **CLSI** | **MIC** | **20** | **20** | **NA** | **NA** | **NA** | **20** |
| L. Lu, et al. (578) | **2022** | **China** | **CLSI** | **MIC** | **29** | **20** | **NA** | **NA** | **20** | **NA** |
| M. H. Legese, et al. (579) | **2022** | **Ethiopia** | **CLSI** | **DISK** | **34** | **20** | **NA** | **NA** | **NA** | **20** |
| S. Marti, et al. (580) | **2009** | **Spain** | **CLSI** | **MIC** | **19** | **19** | **19** | **NA** | **NA** | **19** |
| M. Virginia Villegas, et al. (581) | **2011** | **Colombia** | **CLSI** | **MIC** | **34** | **19** | **19** | **NA** | **NA** | **19** |
| N. N. Rakhi, et al. (582) | **2019** | **Bangladesh** | **CLSI** | **DISK** | **71** | **19** | **16** | **NA** | **NA** | **19** |
| D. Carcione, et al. (583) | **2021** | **Italy** | **EUCAST** | **MIC** | **20** | **19** | **NA** | **NA** | **NA** | **19** |
| N. Zahra, et al. (584) | **2021** | **Iran** | **CLSI** | **DISK** | **90** | **19** | **NA** | **NA** | **NA** | **19** |
| P. Di Carlo, et al. (585) | **2021** | **Italy** | **EUCAST** | **MIC** | **19** | **19** | **NA** | **19** | **NA** | **19** |
| A. A. K. Shali, et al. (586) | **2022** | **Iraq** | **CLSI** | **MIC** | **27** | **19** | **NA** | **NA** | **NA** | **19** |
| O. Aydemir, et al. (587) | **2022** | **Turkey** | **EUCAST** | **MIC** | **28** | **19** | **NA** | **NA** | **NA** | **19** |
| S.-S. Jean, et al. (588) | **2022** | **Taiwan** | **CLSI** | **MIC** | **35** | **19** | **NA** | **NA** | **NA** | **19** |
| G. Fadda, et al. (589) | **2004** | **Italy** | **CLSI** | **MIC** | **114** | **18** | **NA** | **NA** | **NA** | **18** |
| A. Karunasagar, et al. (590) | **2011** | **India** | **CLSI** | **DISK** | **48** | **18** | **NA** | **NA** | **NA** | **18** |
| L. Zhong, et al. (591) | **2012** | **China** | **(591)CLSI** | **MIC** | **18** | **18** | **NA** | **NA** | **NA** | **18** |
| P. Martinez, et al. (592) | **2012** | **Colombia** | **CLSI** | **MIC** | **20** | **18** | **NA** | **NA** | **NA** | **18** |
| F. Gao, et al. (593) | **2015** | **China** | **NA** | **DISK** | **24** | **18** | **NA** | **NA** | **NA** | **18** |
| P. H. Van, et al. (594) | **2017** | **NA** | **CLSI** | **MIC** | **84** | **18** | **NA** | **NA** | **NA** | **18** |
| A. OLo-Okere, et al. (595) | **2020** | **Nigeria** | **CLSI** | **MIC** | **21** | **18** | **NA** | **NA** | **NA** | **18** |
| E. A. Ribeiro, et al. (596) | **2020** | **Brazil** | **NA** | **MIC** | **18** | **18** | **NA** | **NA** | **NA** | **18** |
| M. Bori, et al. (597) | **2023** | **India** | **NA** | **NA** | **44** | **18** | **NA** | **NA** | **NA** | **18** |
| R. Khalifa, et al. (598) | **2023** | **Egypt** | **CLSI** | **MIC** | **20** | **18** | **NA** | **NA** | **NA** | **18** |
| P. Bogaerts, et al. (599) | **2006** | **Belgium** | **CLSI** | **DISK** | **18** | **17** | **NA** | **NA** | **NA** | **17** |
| M. Matsui, et al. (600) | **2018** | **Japan** | **CLSI** | **DISK** | **645** | **17** | **NA** | **NA** | **NA** | **17** |
| N. S. Ravi, et al. (601) | **2018** | **India** | **CLSI** | **DISK** | **17** | **17** | **NA** | **NA** | **NA** | **17** |
| W. Duszynska, et al. (602) | **2018** | **Poland** | **EUCAST** | **MIXED** | **183** | **17** | **NA** | **NA** | **NA** | **17** |
| G. Ayar, et al. (603) | **2019** | **Turkey** | **CLSI** | **DISK** | **84** | **17** | **NA** | **NA** | **17** | **NA** |
| N. Trapaidze, et al. (604) | **2019** | **Georgia** | **NA** | **MIC** | **20** | **17** | **NA** | **NA** | **17** | **NA** |
| A. Y, et al. (605) | **2020** | **Saudi Arabia** | **CLSI** | **MIC** | **17** | **17** | **NA** | **NA** | **NA** | **17** |
| B. Ingti, et al. (606) | **2020** | **India** | **CLSI** | **DISK** | **79** | **17** | **NA** | **NA** | **17** | **NA** |
| U. Ghimire, et al. (607) | **2021** | **Nepal** | **CLSI** | **DISK** | **19** | **17** | **NA** | **NA** | **NA** | **17** |
| T. San, et al. (608) | **2022** | **Myanmar** | **CLSI** | **MIC** | **29** | **17** | **NA** | **NA** | **NA** | **17** |
| M. Arbune, et al. (609) | **2021** | **Romania** | **CLSI** | **MIC** | **28** | **16** | **NA** | **NA** | **NA** | **16** |
| A. I. I. Aedh, et al. (610) | **2023** | **Saudi Arabia** | **NA** | **MIC** | **28** | **16** | **NA** | **NA** | **NA** | **16** |
| B. S. Alotaibi, et al. (611) | **2023** | **Saudi Arabia** | **CLSI** | **MIC** | **16** | **16** | **NA** | **NA** | **NA** | **16** |
| N. C. Cheng, et al. (612) | **2005** | **Taiwan** | **CLSI** | **MIC** | **72** | **15** | **NA** | **NA** | **NA** | **15** |
| T. K. W. Ling, et al. (613) | **2005** | **China** | **CLSI** | **MIC** | **212** | **15** | **NA** | **NA** | **NA** | **15** |
| S. Mostofi, et al. (614) | **2011** | **Iran** | **CLSI** | **DISK** | **50** | **15** | **NA** | **NA** | **NA** | **15** |
| Q. Zhong, et al. (615) | **2012** | **China** | **CLSI** | **MIC** | **33** | **15** | **NA** | **NA** | **NA** | **15** |
| B. Todorova, et al. (616) | **2014** | **Bulgaria** | **NA** | **MIC** | **15** | **15** | **NA** | **NA** | **NA** | **15** |
| Sjol, et al. (617) | **2014** | **NA** | **NA** | **DISK** | **15** | **15** | **NA** | **NA** | **NA** | **15** |
| S. S. Ahmed, et al. (618) | **2016** | **Libya** | **NA** | **MIC** | **16** | **15** | **NA** | **NA** | **NA** | **15** |
| G. M. M. Abesamis, et al. (619) | **2019** | **Philippines** | **NA** | **NA** | **21** | **15** | **NA** | **NA** | **NA** | **15** |
| A. Kabrah(620) | **2022** | **Saudi Arabia** | **CLSI** | **DISK** | **15** | **15** | **NA** | **NA** | **NA** | **15** |
| B. Balazs, et al. (621) | **2022** | **Hungary** | **EUCAST** | **DISK** | **55** | **15** | **NA** | **NA** | **NA** | **15** |
| D. Liu, et al. (622) | **2022** | **China** | **CLSI** | **MIC** | **15** | **15** | **NA** | **NA** | **NA** | **15** |
| A. Lavrinenko, et al. (623) | **2023** | **Kazakhstan** | **CLSI** | **DISK** | **17** | **15** | **NA** | **NA** | **NA** | **15** |
| Q. Meng, et al. (624) | **2023** | **China** | **CLSI** | **MIXED** | **147** | **15** | **NA** | **NA** | **NA** | **15** |
| P. S. Shareek, et al. (625) | **2012** | **India** | **CLSI** | **DISK** | **57** | **14** | **13** | **NA** | **NA** | **14** |
| M. A. Hashmi, et al. (626) | **2020** | **Pakistan** | **NA** | **NA** | **14** | **14** | **NA** | **NA** | **NA** | **14** |
| M. T. H. Talpur, et al. (627) | **2020** | **Pakistan** | **CLSI** | **DISK** | **16** | **14** | **NA** | **NA** | **14** | **NA** |
| H. A. Mohamed, et al. (628) | **2022** | **Egypt** | **CLSI** | **DISK** | **20** | **14** | **NA** | **NA** | **NA** | **14** |
| K. Petersen, et al. (629) | **2011** | **NA** | **CLSI** | **DISK** | **44** | **13** | **NA** | **NA** | **NA** | **13** |
| X. Wang, et al. (630) | **2017** | **China** | **CLSI** | **DISK** | **36** | **13** | **NA** | **NA** | **13** | **NA** |
| Y. Chen, et al. (631) | **2020** | **China** | **CLSI** | **MIC** | **21** | **13** | **NA** | **NA** | **NA** | **13** |
| T. Sewunet, et al. (632) | **2022** | **Ethiopia** | **CLSI** | **DISK** | **31** | **13** | **NA** | **NA** | **NA** | **13** |
| B. Chakraborty, et al. (633) | **2011** | **India** | **CLSI** | **DISK** | **88** | **12** | **NA** | **NA** | **NA** | **12** |
| P. Sacha, et al. (634) | **2012** | **Poland** | **CLSI** | **MIC** | **51** | **12** | **NA** | **NA** | **NA** | **12** |
| S. Nishida, et al. (635) | **2018** | **Japan** | **CLSI** | **MIC** | **16** | **12** | **NA** | **NA** | **NA** | **12** |
| M. H.-Y. Wong, et al. (636) | **2019** | **China** | **CLSI** | **MIC** | **14** | **12** | **NA** | **NA** | **NA** | **12** |
| J.-B. Chang, et al. (637) | **2020** | **China** | **CLSI** | **DISK** | **16** | **12** | **NA** | **NA** | **NA** | **12** |
| M. Shabban, et al. (638) | **2020** | **Egypt** | **CLSI** | **DISK** | **14** | **12** | **NA** | **NA** | **NA** | **12** |
| S. A. L. Al Meani, et al. (639) | **2020** | **Iraq** | **CLSI** | **DISK** | **22** | **12** | **NA** | **NA** | **NA** | **12** |
| W. El-Kazzaz, et al. (640) | **2020** | **Egypt** | **CLSI** | **DISK** | **29** | **12** | **17** | **20** | **NA** | **12** |
| Z.-J. Li, et al. (641) | **2021** | **China** | **CLSI** | **MIC** | **15** | **12** | **NA** | **NA** | **12** | **12** |
| A. H. Mohamed, et al. (642) | **2022** | **Somalia** | **CLSI** | **DISK** | **24** | **12** | **NA** | **19** | **NA** | **12** |
| E. E. Odih, et al. (643) | **2023** | **Nigeria** | **CLSI** | **MIC** | **33** | **12** | **15** | **NA** | **NA** | **12** |
| J. M. Adams-Haduch, et al. (643) | **2008** | **US** | **CLSI** | **DISK** | **49** | **11** | **NA** | **NA** | **NA** | **11** |
| Paj, et al. (644) | **2013** | **Iran** | **CLSI** | **MIC** | **75** | **11** | **NA** | **NA** | **NA** | **11** |
| J. E. Sam, et al. (645) | **2018** | **Malaysia** | **NA** | **NA** | **14** | **11** | **NA** | **NA** | **NA** | **11** |
| L. Yang, et al. (646) | **2018** | **Iraq** | **CLSI** | **DISK** | **11** | **11** | **NA** | **NA** | **NA** | **11** |
| S. Tehrani, et al. (647) | **2019** | **Iran** | **CLSI** | **DISK** | **11** | **11** | **NA** | **NA** | **NA** | **11** |
| A. Pal, et al. (648) | **2020** | **India** | **CLSI** | **MIC** | **11** | **11** | **NA** | **NA** | **NA** | **11** |
| N. El-Kattan, et al. (649) | **2021** | **India** | **CLSI** | **DISK** | **14** | **11** | **NA** | **NA** | **NA** | **11** |
| J. Zhang, et al. (650) | **2022** | **China** | **CLSI** | **DISK** | **11** | **11** | **NA** | **NA** | **NA** | **11** |
| P. Ioannou, et al. (651) | **2023** | **Greece** | **CLSI** | **MIC** | **16** | **11** | **NA** | **NA** | **NA** | **11** |
| E. J. Giamarellos-Bourboulis, et al. (652) | **2001** | **Greece** | **NA** | **DISK** | **39** | **10** | **NA** | **NA** | **NA** | **10** |
| N. Goel, et al. (653) | **2009** | **India** | **CLSI** | **DISK** | **38** | **10** | **NA** | **NA** | **NA** | **10** |
| F. F. Tuon, et al. (654) | **2010** | **Brazil** | **CLSI** | **DISK** | **22** | **10** | **NA** | **NA** | **NA** | **10** |
| B. Zhang, et al. (655) | **2012** | **China** | **CLSI** | **DISK** | **14** | **10** | **NA** | **NA** | **NA** | **10** |
| S. Notake, et al. (656) | **2013** | **Japan** | **CLSI** | **MIC** | **10** | **10** | **NA** | **NA** | **NA** | **10** |
| R. A. R. Aziz, et al. (657) | **2017** | **Iraq** | **CLSI** | **MIC** | **24** | **10** | **NA** | **NA** | **NA** | **10** |
| A. M. Gezmu, et al. (658) | **2021** | **South Africa** | **CLSI** | **MIC** | **21** | **10** | **NA** | **NA** | **10** | **NA** |
| K. Subramaniam, et al. (659) | **2021** | **Malaysia** | **CLSI** | **MIC** | **29** | **10** | **NA** | **NA** | **NA** | **10** |
| N. Kansak, et al. (658) | **2021** | **Turkey** | **CLSI** | **DISK** | **10** | **10** | **NA** | **NA** | **NA** | **10** |
| M. R. Taysi, et al. (660) | **2023** | **Turkey** | **NA** | **MIC** | **10** | **10** | **NA** | **NA** | **10** | **NA** |
| C. Mendes, et al. (646, 661) | **2005** | **Brazil** | **CLSI** | **MIC** | **89** | **9** | **NA** | **NA** | **NA** | **9** |
| A. Deveci, et al. (662) | **2012** | **Turkey** | **CLSI** | **MIC** | **10** | **9** | **NA** | **NA** | **NA** | **9** |
| L. F. Mataseje, et al. (663) | **2012** | **Canada** | **CLSI** | **MIC** | **9** | **9** | **NA** | **9** | **NA** | **9** |
| M. Chaudhary, et al. (664) | **2014** | **India** | **CLSI** | **DISK** | **52** | **9** | **NA** | **NA** | **NA** | **9** |
| R. Soltani, et al. (665) | **2014** | **Iran** | **NA** | **DISK** | **9** | **9** | **NA** | **NA** | **NA** | **9** |
| A. Yoshizumi, et al. (666) | **2015** | **Japan** | **NA** | **NA** | **9** | **9** | **NA** | **NA** | **9** | **NA** |
| T. Elkersh, et al. (667) | **2015** | **NA** | **NA** | **MIC** | **9** | **9** | **NA** | **NA** | **NA** | **9** |
| D. P. Kateete, et al. (668) | **2016** | **Uganda** | **CLSI** | **MIC** | **29** | **9** | **NA** | **NA** | **9** | **NA** |
| J. H. Woo, et al. (669) | **2017** | **South Korea** | **CLSI** | **MIC** | **9** | **9** | **NA** | **NA** | **NA** | **9** |
| B. Bedenic, et al. (670) | **2020** | **Croatia** | **CLSI** | **MIC** | **9** | **9** | **NA** | **NA** | **NA** | **9** |
| A. Szczypta, et al. (671) | **2021** | **NA** | **EUCAST** | **DISK** | **9** | **9** | **NA** | **NA** | **NA** | **9** |
| H. C. Yun, et al. (672) | **2006** | **Iraq** | **CLSI** | **MIC** | **89** | **8** | **NA** | **NA** | **NA** | **8** |
| B. Bedenic, et al. (673) | **2010** | **Croatia** | **CLSI** | **MIC** | **169** | **8** | **NA** | **NA** | **NA** | **8** |
| H. √ñzdemir, et al. (674) | **2011** | **Turkey** | **CLSI** | **DISK** | **15** | **8** | **NA** | **NA** | **NA** | **8** |
| I. Netsvyetayeva, et al. (675) | **2011** | **Poland** | **NA** | **MIC** | **16** | **8** | **NA** | **NA** | **NA** | **8** |
| M. Shanthi, et al. (676) | **2011** | **NA** | **CLSI** | **MIC** | **25** | **8** | **NA** | **NA** | **NA** | **8** |
| M. Hornsey, et al. (677) | **2013** | **UK** | **BSAC** | **MIXED** | **9** | **8** | **NA** | **NA** | **NA** | **8** |
| S. Niakan, et al. (678) | **2013** | **Iran** | **CLSI** | **DISK** | **60** | **8** | **NA** | **NA** | **NA** | **8** |
| W. Nageeb, et al. (679) | **2014** | **Egypt** | **NA** | **DISK** | **10** | **8** | **NA** | **NA** | **NA** | **8** |
| R. A. Al-Hindawi, et al. (680) | **2018** | **Iraq** | **CLSI** | **DISK** | **17** | **8** | **NA** | **NA** | **8** | **NA** |
| F. Moges, et al. (681) | **2019** | **Ethiopia** | **CLSI** | **DISK** | **15** | **8** | **NA** | **NA** | **8** | **NA** |
| Z. G. Luo, et al. (682) | **2020** | **China** | **CLSI** | **MIC** | **45** | **8** | **NA** | **NA** | **NA** | **8** |
| A. Thatrimontrichai, et al. (683) | **2021** | **Thailand** | **CLSI** | **DISK** | **12** | **8** | **NA** | **NA** | **8** | **NA** |
| N. Duan, et al. (684) | **2021** | **China** | **CLSI** | **MIXED** | **24** | **8** | **NA** | **NA** | **NA** | **8** |
| R. L. Boone, et al. (685) | **2021** | **US** | **ISO** | **NA** | **17** | **8** | **NA** | **NA** | **NA** | **8** |
| M. A. Chilon-Chavez, et al. (686) | **2022** | **Peru** | **CLSI** | **MIC** | **72** | **8** | **NA** | **NA** | **NA** | **8** |
| S. Hattab, et al. (687) | **2022** | **Palestine** | **CLSI** | **MIC** | **9** | **8** | **NA** | **NA** | **NA** | **8** |
| R. M. Abd El-Baky, et al. (688) | **2020** | **Egypt** | **NA** | **DISK** | **20** | **7** | **NA** | **NA** | **NA** | **7** |
| A. Mahmoud, et al. (689) | **2021** | **Egypt** | **CLSI** | **MIC** | **10** | **7** | **NA** | **NA** | **NA** | **7** |
| A. Petca, et al. (690) | **2021** | **Romania** | **EUCAST** | **MIC** | **7** | **7** | **NA** | **NA** | **7** | **NA** |
| A. Z. M. Maebed, et al. (691) | **2021** | **Egypt** | **CLSI** | **MIC** | **7** | **7** | **NA** | **NA** | **NA** | **7** |
| H. Mekonnen, et al. (692) | **2021** | **Ethiopia** | **CLSI** | **DISK** | **16** | **7** | **NA** | **NA** | **7** | **NA** |
| N. Palanisamy, et al. (693) | **2021** | **India** | **CLSI** | **DISK** | **21** | **7** | **NA** | **5** | **NA** | **7** |
| P. Manohar, et al. (694) | **2021** | **India** | **CLSI** | **DISK** | **7** | **7** | **NA** | **NA** | **NA** | **7** |
| K. Loivukene, et al. (695) | **2006** | **Estonia** | **CLSI** | **MIC** | **128** | **6** | **NA** | **NA** | **NA** | **6** |
| K. L√µivukene, et al. (696) | **2006** | **Estonia** | **CLSI** | **MIC** | **95** | **6** | **NA** | **NA** | **NA** | **6** |
| Y. Lee, et al. (404) | **2010** | **Taiwan** | **NA** | **DISK** | **6** | **6** | **NA** | **NA** | **NA** | **6** |
| K. R. Peck, et al. (697) | **2012** | **South Korea** | **CLSI** | **MIC** | **6** | **6** | **NA** | **NA** | **NA** | **6** |
| H. G. Wu, et al. (698) | **2018** | **China** | **CLSI** | **MIC** | **74** | **6** | **NA** | **NA** | **NA** | **6** |
| M. Babu, et al. (699) | **2018** | **India** | **CLSI** | **MIXED** | **7** | **6** | **NA** | **NA** | **NA** | **6** |
| P. Mule, et al. (700) | **2018** | **India** | **CLSI** | **MIC** | **36** | **6** | **6** | **NA** | **NA** | **6** |
| A. K. R. Purba, et al. (701) | **2019** | **Indonesia** | **CLSI** | **DISK** | **28** | **6** | **NA** | **NA** | **NA** | **6** |
| J.-D. Lu, et al. (702) | **2019** | **China** | **NA** | **NA** | **44** | **6** | **NA** | **NA** | **NA** | **6** |
| A. A. A. Bediako-Bowan, et al. (703) | **2020** | **Ghana** | **EUCAST** | **DISK** | **23** | **6** | **NA** | **NA** | **NA** | **6** |
| K. Murata, et al. (704) | **2020** | **Japan** | **CLSI** | **MIC** | **43** | **6** | **NA** | **NA** | **NA** | **6** |
| F. M. Al-Asady, et al. (705) | **2021** | **Iraq** | **NA** | **MIC** | **8** | **6** | **NA** | **NA** | **NA** | **6** |
| Z. Zhang, et al. (706) | **2021** | **China** | **CLSI** | **MIC** | **6** | **6** | **NA** | **NA** | **6** | **6** |
| P. Y. Liu, et al. (707) | **1995** | **Taiwan** | **CLSI** | **MIC** | **62** | **5** | **NA** | **NA** | **NA** | **5** |
| M. Mccracken, et al. (708) | **2011** | **Canada** | **CLSI** | **MIC** | **66** | **5** | **NA** | **NA** | **NA** | **5** |
| M. S. Alhaddad, et al. (709) | **2018** | **Saudi Arabia** | **NA** | **MIC** | **8** | **5** | **NA** | **NA** | **NA** | **5** |
| T. K. Atik, et al. (710) | **2018** | **Turkey** | **CLSI** | **MIC** | **5** | **5** | **NA** | **NA** | **NA** | **5** |
| D. Viderman, et al. (711) | **2019** | **Kazakhstan** | **CLSI** | **DISK** | **11** | **5** | **NA** | **NA** | **NA** | **5** |
| S. N. Gaber, et al. (712) | **2020** | **Egypt** | **CLSI** | **DISK** | **5** | **5** | **NA** | **NA** | **NA** | **5** |
| M. Inggraini, et al. (713) | **2021** | **India** | **CLSI** | **MIC** | **5** | **5** | **NA** | **NA** | **NA** | **5** |
| M. H. Haider, et al. (714) | **2022** | **Pakistan** | **CLSI** | **DISK** | **12** | **5** | **NA** | **NA** | **NA** | **5** |
| O. E. Khokhlova, et al. (715) | **2022** | **Russia** | **NA** | **MIXED** | **8** | **5** | **NA** | **NA** | **NA** | **5** |
| P. O. Barth, et al. (716) | **2023** | **Brazil** | **EUCAST** | **DISK** | **14** | **5** | **NA** | **NA** | **NA** | **5** |
| Y. Liu, et al. (717) | **2023** | **China** | **CLSI** | **MIC** | **18** | **5** | **NA** | **NA** | **NA** | **5** |
| C. Kiffer, et al. (718) | **2005** | **Brazil** | **CLSI** | **MIC** | **137** | **4** | **NA** | **NA** | **NA** | **4** |
| K. C. Guelfi, et al. (719) | **2008** | **Brazil** | **CLSI** | **MIC** | **9** | **4** | **NA** | **NA** | **NA** | **4** |
| D. M. Hensley (720) | **2010** | **US** | **NA** | **MIC** | **10** | **4** | **NA** | **NA** | **NA** | **4** |
| F. Khamesipour, et al. (721) | **2017** | **Iran** | **CLSI** | **DISK** | **121** | **4** | **NA** | **NA** | **NA** | **4** |
| V. Cafiso, et al. (722) | **2018** | **Italy** | **EUCAST** | **MIC** | **4** | **4** | **NA** | **NA** | **NA** | **4** |
| D. Usjak, et al. (723) | **2019** | **NA** | **CLSI** | **MIC** | **5** | **4** | **NA** | **NA** | **NA** | **4** |
| A. S. N. Haitham, et al. (724) | **2020** | **Romania** | **CLSI** | **MIC** | **25** | **4** | **NA** | **NA** | **4** | **NA** |
| M. Shahid, et al. (725) | **2020** | **India** | **EUCAST** | **DISK** | **4** | **4** | **NA** | **NA** | **NA** | **4** |
| Norafika, et al. (726) | **2020** | **Indonesia** | **CLSI** | **DISK** | **4** | **4** | **NA** | **NA** | **NA** | **4** |
| A. Mohamed, et al. (727) | **2021** | **Egypt** | **CLSI** | **DISK** | **4** | **4** | **NA** | **NA** | **NA** | **4** |
| H. Guan, et al. (728) | **2021** | **China** | **CLSI** | **MIC** | **15** | **4** | **NA** | **NA** | **NA** | **4** |
| Y. Takebayashi, et al. (729) | **2021** | **UK** | **CLSI** | **MIXED** | **10** | **4** | **4** | **NA** | **NA** | **4** |
| M. Li, et al. (730) | **2022** | **China** | **CLSI** | **MIC** | **15** | **4** | **NA** | **NA** | **NA** | **4** |
| A.-C. Dikoumba, et al. (731) | **2023** | **Gabon** | **EUCAST** | **MIC** | **15** | **4** | **NA** | **NA** | **4** | **NA** |
| Z. Y. Shi, et al. (732) | **1996** | **Taiwan** | **CLSI** | **MIC** | **248** | **3** | **NA** | **NA** | **3** | **3** |
| D. G. Dauner, et al. (733) | **2008** | **Georgia** | **NA** | **MIC** | **129** | **3** | **NA** | **NA** | **NA** | **3** |
| F. E. Al-Otaibi, et al. (734) | **2016** | **Saudi Arabia** | **CLSI** | **MIC** | **11** | **3** | **NA** | **NA** | **NA** | **3** |
| C. Hu, et al. (735) | **2018** | **China** | **CLSI** | **DISK** | **64** | **3** | **NA** | **NA** | **NA** | **3** |
| C. Rocha, et al. (736) | **2019** | **Peru** | **NA** | **NA** | **124** | **3** | **NA** | **NA** | **NA** | **3** |
| R. Furau, et al. (737) | **2019** | **Romania** | **CLSI** | **DISK** | **4** | **3** | **NA** | **NA** | **3** | **NA** |
| S. M. Mortazavi, et al. (738) | **2020** | **Ethiopia** | **CLSI** | **DISK** | **9** | **3** | **NA** | **NA** | **NA** | **3** |
| O. Akgul, et al. (739) | **2021** | **Turkey** | **NA** | **MIC** | **3** | **3** | **NA** | **3** | **NA** | **3** |
| R. Khodashahi, et al. (740) | **2022** | **Iran** | **CLSI** | **MIC** | **3** | **3** | **NA** | **NA** | **NA** | **3** |
| K. Yamba, et al. (741) | **2023** | **Zambia** | **CLSI** | **MIC** | **15** | **3** | **NA** | **NA** | **NA** | **3** |
| M. M. Mustafai, et al. (742) | **2023** | **Pakistan** | **CLSI** | **DISK** | **12** | **3** | **NA** | **NA** | **NA** | **3** |
| E. Savov, et al. (743) | **2002** | **Bulgaria** | **CLSI** | **DISK** | **52** | **2** | **NA** | **NA** | **NA** | **2** |
| J. Blahova, et al. (744) | **2004** | **Slovakia** | **CLSI** | **DISK** | **25** | **2** | **NA** | **NA** | **NA** | **2** |
| J. A. Patzer, et al. (745) | **2008** | **Poland** | **CLSI** | **MIC** | **137** | **2** | **NA** | **NA** | **NA** | **2** |
| M. Mccracken, et al. (746) | **2009** | **Canada** | **CLSI** | **MIC** | **26** | **2** | **NA** | **NA** | **NA** | **2** |
| J. Hrabak, et al. (747) | **2012** | **Egypt** | **EUCAST** | **MIC** | **2** | **2** | **NA** | **NA** | **NA** | **2** |
| L. Krizova, et al. (748) | **2013** | **NA** | **CLSI** | **MIC** | **17** | **2** | **NA** | **NA** | **NA** | **2** |
| H. N. Wen, et al. (749) | **2017** | **Indonesia** | **NA** | **NA** | **10** | **2** | **NA** | **NA** | **NA** | **2** |
| A. Ayibieke, et al. (750) | **2020** | **Ghana** | **CLSI** | **MIC** | **24** | **2** | **NA** | **NA** | **NA** | **2** |
| A. Chaurasia, et al. (751) | **2021** | **India** | **CLSI** | **DISK** | **3** | **2** | **NA** | **NA** | **2** | **NA** |
| B. Baljin, et al. (752) | **2021** | **Mongolia** | **EUCAST** | **DISK** | **5** | **2** | **NA** | **NA** | **NA** | **2** |
| S. J. Moyo, et al. (753) | **2021** | **Tanzania** | **CLSI** | **MIC** | **12** | **2** | **NA** | **NA** | **NA** | **2** |
| A. Shami, et al. (754) | **2022** | **Saudi Arabia** | **NA** | **MIC** | **2** | **2** | **NA** | **NA** | **NA** | **2** |
| S. S. Masoud, et al. (755) | **2022** | **Tanzania** | **CLSI** | **DISK** | **6** | **2** | **NA** | **NA** | **2** | **NA** |
| S. Srifuengfung, et al. (756) | **2005** | **Thailand** | **CLSI** | **NA** | **11** | **1** | **NA** | **NA** | **NA** | **1** |
| P. G. Higgins, et al. (757) | **2009** | **Brazil** | **CLSI** | **MIXED** | **1** | **1** | **NA** | **NA** | **NA** | **1** |
| T. W. Boo, et al. (758) | **2009** | **Ireland** | **CLSI** | **MIC** | **25** | **1** | **NA** | **NA** | **NA** | **1** |
| B. S. Lopes, et al. (759) | **2012** | **Portugal** | **BSAC** | **MIC** | **1** | **1** | **NA** | **NA** | **NA** | **1** |
| C. De Souza Gusatti, et al. (760) | **2012** | **Brazil** | **CLSI** | **MIC** | **1** | **1** | **NA** | **NA** | **NA** | **1** |
| S. Figueiredo, et al. (761) | **2012** | **France** | **CLSI** | **DISK** | **1** | **1** | **1** | **1** | **NA** | **1** |
| A. M. Asaad, et al. (762) | **2013** | **Saudi Arabia** | **CLSI** | **MIC** | **68** | **1** | **NA** | **NA** | **NA** | **1** |
| G. Peirano, et al. (763) | **2013** | **Canada** | **CLSI** | **MIC** | **1** | **1** | **NA** | **NA** | **NA** | **1** |
| P. E. Waterman, et al. (764) | **2013** | **Honduras** | **CLSI** | **MIC** | **1** | **1** | **NA** | **NA** | **NA** | **1** |
| P. Espinal, et al. (765) | **2013** | **Spain** | **CLSI** | **MIC** | **1** | **1** | **NA** | **NA** | **NA** | **1** |
| D. P. Kateete, et al. (766) | **2017** | **Uganda** | **NA** | **NA** | **7** | **1** | **NA** | **NA** | **NA** | **1** |
| S. Lakoh, et al. (767) | **2020** | **Sierra Leone** | **NA** | **MIC** | **14** | **1** | **NA** | **NA** | **NA** | **1** |
| P. Hannus, et al. (768) | **2021** | **Finland** | **EUCAST** | **MIXED** | **1** | **1** | **NA** | **NA** | **NA** | **1** |
| V.-T. Hoang, et al. (769) | **2021** | **France** | **EUCAST** | **DISK** | **1** | **1** | **NA** | **NA** | **1** | **NA** |
| N. Kumari, et al. (770) | **2022** | **India** | **CLSI** | **DISK** | **7** | **1** | **NA** | **NA** | **1** | **NA** |
| R. Rabayah, et al. (771) | **2022** | **Palestine** | **CLSI** | **MIC** | **6** | **1** | **NA** | **NA** | **NA** | **1** |
| U. Frank, et al. (772) | **2000** | **Germany** | **CLSI** | **MIC** | **53** | **0** | **NA** | **NA** | **NA** | **0** |
| A. Hostacka, et al. (773) | **2002** | **Slovakia** | **NA** | **MIC** | **42** | **0** | **NA** | **NA** | **NA** | **0** |
| M. Ayan, et al. (774) | **2003** | **Turkey** | **CLSI** | **DISK** | **52** | **0** | **NA** | **NA** | **NA** | **0** |
| N. Nagano, et al. (775) | **2004** | **Japan** | **CLSI** | **MIC** | **3** | **0** | **NA** | **NA** | **NA** | **0** |
| L. Mihaljevic, et al. (776) | **2007** | **Croatia** | **CLSI** | **NA** | **9** | **0** | **NA** | **NA** | **NA** | **0** |
| L. Mihaljevic, et al. (482) | **2007** | **Croatia** | **CLSI** | **NA** | **16** | **0** | **NA** | **NA** | **NA** | **0** |
| M. Al-Yaqoubi, et al. (777) | **2008** | **Oman** | **CLSI** | **MIC** | **17** | **0** | **NA** | **NA** | **NA** | **0** |
| A. Potron, et al. (778) | **2011** | **Haiti** | **CLSI** | **MIXED** | **3** | **0** | **NA** | **NA** | **NA** | **0** |
| D. K. Tiwari, et al. (779) | **2013** | **India** | **CLSI** | **DISK** | **1** | **0** | **NA** | **NA** | **NA** | **0** |
| H. D. Tran, et al. (780) | **2022** | **Vietnam** | **NA** | **MIC** | **9** | **0** | **NA** | **NA** | **NA** | **0** |
| K. I. Alqumaizi, et al. (781) | **2022** | **India** | **CLSI** | **DISK** | **30** | **0** | **0** | **NA** | **NA** | **0** |
| S. Lakoh, et al. (782) | **2022** | **Sierra Leone** | **NA** | **MIC** | **5** | **0** | **NA** | **NA** | **NA** | **0** |
| A. Bitew, et al. (783) | **2023** | **Ethiopia** | **EUCAST** | **DISK** | **7** | **0** | **NA** | **NA** | **NA** | **0** |
| Z. Kareem Raheem, et al. (784) | **2023** | **Iraq** | **NA** | **MIC** | **2** | **0** | **2** | **2** | **NA** | **0** |
| M. D. Zilberberg, et al. (785) | **2016** | **US** | **CLSI** | **NA** | **39320** | **NA** | **35505** | **NA** | **NA** | **NA** |
| H. Goudarzi, et al(786) | **2015** | **Iran** | **NA** | **DISK** | **128** | **NA** | **124** | **127** | **NA** | **NA** |
| S. A. Clock, et al. (787) | **2013** | **US** | **CLSI** | **MIC** | **48** | **NA** | **48** | **NA** | **NA** | **NA** |
| M. Gilani, et al. (788) | **2015** | **Pakistan** | **NA** | **MIC** | **30** | **NA** | **23** | **NA** | **NA** | **NA** |
| G. Bansal, et al. (789) | **2020** | **US** | **CLSI** | **MIC** | **28** | **NA** | **13** | **NA** | **NA** | **NA** |
| K. Amsler, et al. (790) | **2010** | **US** | **NA** | **MIC** | **20** | **NA** | **12** | **NA** | **NA** | **NA** |
| E. C.-M. Leung, et al. (791) | **2019** | **China** | **CLSI** | **MIC** | **20** | **NA** | **11** | **NA** | **NA** | **NA** |
| B. Santella, et al. (792) | **2021** | **Italy** | **EUCAST** | **MIC** | **879** | **NA** | **NA** | **879** | **NA** | **NA** |
| U. Devi, et al. (793) | **2018** | **India** | **CLSI** | **DISK** | **9** | **NA** | **NA** | **7** | **NA** | **NA** |
| A. H. Mohamed, et al. (794) | **2021** | **Somalia** | **CLSI** | **DISK** | **31** | **NA** | **NA** | **5** | **NA** | **NA** |
| M. Guembe, et al. (795) | **2008** | **Spain** | **CLSI** | **MIC** | **3** | **NA** | **NA** | **2** | **NA** | **NA** |

# Supplementary Table 2: Risk of Bias Assessment

| Column1 | Q1 | Q2 | Q3 | Q4 | Q5 | Q6 | Q7 | Q8 |
| --- | --- | --- | --- | --- | --- | --- | --- | --- |
| P. Y. Liu, et al. (1995) | **High** | **High** | **Low** | **Low** | **Low** | **High** | **Low** | **Low** |
| Z. Y. Shi, et al. (1996) | **High** | **Low** | **Low** | **Low** | **High** | **Low** | **Low** | **Low** |
| U. Frank, et al. (2000) | **High** | **Low** | **Low** | **High** | **Low** | **High** | **Low** | **Low** |
| E. J. Giamarellos-Bourboulis, et al. (2001) | **High** | **High** | **Low** | **High** | **High** | **Low** | **High** | **Low** |
| P. R. Hsueh, et al. (2001) | **High** | **Low** | **Low** | **Low** | **High** | **Low** | **Low** | **Low** |
| A. Hostacka, et al. (2002) | **High** | **High** | **Low** | **High** | **Low** | **Low** | **High** | **High** |
| E. Savov, et al. (2002) | **High** | **High** | **Low** | **High** | **Low** | **Low** | **Low** | **High** |
| J. A. Garcia-Rodriguez, et al. (2002) | **High** | **High** | **Low** | **High** | **High** | **Low** | **Low** | **High** |
| S. S. Jean, et al. (2002) | **High** | **Low** | **Low** | **Low** | **Low** | **Low** | **Low** | **Low** |
| M. Ayan, et al. (2003) | **High** | **Low** | **Low** | **High** | **Low** | **Low** | **Low** | **High** |
| P. J. Turner, et al. (2003) | **High** | **High** | **Low** | **Low** | **Low** | **Low** | **Low** | **Low** |
| C. R. V. Kiffer, et al. (2004) | **High** | **Low** | **Low** | **Low** | **Low** | **Low** | **High** | **High** |
| G. Fadda, et al. (2004) | **High** | **High** | **Low** | **Low** | **High** | **Low** | **Low** | **Low** |
| J. Blahova, et al. (2004) | **High** | **Low** | **Low** | **High** | **High** | **Low** | **Low** | **High** |
| J. F. Turton, et al. (2004) | **High** | **High** | **Low** | **High** | **High** | **Low** | **High** | **High** |
| N. Nagano, et al. (2004) | **High** | **Low** | **Low** | **High** | **Low** | **Low** | **Low** | **High** |
| R. Zarrilli, et al. (2004) | **High** | **Low** | **Low** | **High** | **Low** | **Low** | **Low** | **High** |
| C. Kiffer, et al. (2005) | **High** | **Low** | **Low** | **Low** | **Low** | **Low** | **Low** | **Low** |
| C. Mendes, et al. (2005) | **High** | **High** | **Low** | **Low** | **Low** | **Low** | **Low** | **Low** |
| N. C. Cheng, et al. (2005) | **High** | **Low** | **Low** | **Low** | **Low** | **Low** | **Low** | **Low** |
| S. Srifuengfung, et al. (2005) | **High** | **Low** | **High** | **High** | **Low** | **Low** | **Low** | **High** |
| T. K. W. Ling, et al. (2005) | **High** | **Low** | **Low** | **Low** | **Low** | **Low** | **Low** | **Low** |
| A. Y. Peleg, et al. (2006) | **High** | **Low** | **Low** | **High** | **Low** | **Low** | **High** | **High** |
| H. C. Yun, et al. (2006) | **High** | **Low** | **Low** | **High** | **Low** | **Low** | **Low** | **High** |
| K. Loivukene, et al. (2006) | **High** | **Low** | **Low** | **Low** | **Low** | **Low** | **Low** | **High** |
| K. Lõivukene, et al. (2006) | **High** | **Low** | **Low** | **Low** | **Low** | **High** | **Low** | **Low** |
| P. Bogaerts, et al. (2006) | **High** | **Low** | **Low** | **High** | **Low** | **Low** | **Low** | **High** |
| A. Brink, et al. (2007) | **High** | **High** | **Low** | **High** | **Low** | **High** | **Low** | **High** |
| D. man, et al. (2007) | **High** | **Low** | **High** | **High** | **Low** | **Low** | **Low** | **Low** |
| H. Wang, et al. (2007) | **High** | **Low** | **Low** | **Low** | **Low** | **Low** | **Low** | **Low** |
| J. Oteo, et al. (2007) | **High** | **Low** | **Low** | **Low** | **Low** | **Low** | **Low** | **Low** |
| L. Mihaljevic, et al. (2007) | **High** | **Low** | **High** | **High** | **Low** | **Low** | **Low** | **High** |
| L. Mihaljevic, et al. (2007) | **High** | **Low** | **High** | **High** | **Low** | **Low** | **Low** | **High** |
| M. H. Scheetz, et al. (2007) | **High** | **High** | **Low** | **High** | **Low** | **Low** | **Low** | **Low** |
| D. G. Dauner, et al. (2008) | **High** | **Low** | **Low** | **High** | **Low** | **Low** | **High** | **High** |
| J. A. Patzer, et al. (2008) | **High** | **High** | **Low** | **Low** | **Low** | **Low** | **Low** | **High** |
| J. M. Adams-Haduch, et al. (2008) | **High** | **Low** | **Low** | **High** | **Low** | **Low** | **Low** | **High** |
| J. Y. Sung, et al. (2008) | **High** | **High** | **Low** | **High** | **Low** | **Low** | **Low** | **High** |
| K. C. Guelfi, et al. (2008) | **High** | **High** | **Low** | **High** | **Low** | **High** | **Low** | **High** |
| M. Al-Yaqoubi, et al. (2008) | **High** | **Low** | **Low** | **Low** | **Low** | **High** | **Low** | **Low** |
| M. Guembe, et al. (2008) | **Low** | **Low** | **Low** | **Low** | **Low** | **Low** | **Low** | **Low** |
| M. M. Feizabadi, et al. (2008) | **High** | **Low** | **Low** | **Low** | **Low** | **Low** | **Low** | **High** |
| M. Rahbar, et al. (2008) | **High** | **Low** | **Low** | **Low** | **Low** | **Low** | **Low** | **High** |
| T. Y. Tan, et al. (2008) | **High** | **Low** | **Low** | **Low** | **Low** | **Low** | **Low** | **Low** |
| A. H. Asghar, et al. (2009) | **High** | **Low** | **High** | **High** | **Low** | **Low** | **Low** | **High** |
| E. S. Cetin, et al. (2009) | **High** | **Low** | **Low** | **Low** | **Low** | **Low** | **Low** | **High** |
| M. Castanheira, et al. (2009) | **High** | **Low** | **Low** | **Low** | **Low** | **Low** | **Low** | **Low** |
| M. J. C. Noyal, et al. (2009) | **High** | **Low** | **Low** | **High** | **Low** | **Low** | **Low** | **High** |
| M. McCracken, et al. (2009) | **High** | **Low** | **Low** | **High** | **High** | **Low** | **Low** | **Low** |
| N. Goel, et al. (2009) | **High** | **Low** | **Low** | **Low** | **Low** | **Low** | **Low** | **High** |
| P. G. Higgins, et al. (2009) | **High** | **High** | **Low** | **Low** | **Low** | **High** | **Low** | **High** |
| R. U. Karthika, et al. (2009) | **High** | **Low** | **Low** | **Low** | **Low** | **High** | **Low** | **High** |
| S. Marti, et al. (2009) | **High** | **High** | **Low** | **High** | **Low** | **High** | **Low** | **High** |
| S. S. Jean, et al. (2009) | **Low** | **Low** | **Low** | **Low** | **Low** | **Low** | **Low** | **Low** |
| T. W. Boo, et al. (2009) | **High** | **High** | **Low** | **High** | **Low** | **Low** | **Low** | **High** |
| Y. B. Long, et al. (2009) | **High** | **Low** | **Low** | **High** | **Low** | **Low** | **High** | **High** |
| B. Bedenic, et al. (2010) | **High** | **Low** | **Low** | **Low** | **Low** | **Low** | **Low** | **High** |
| C.-K. Kim, et al. (2010) | **High** | **High** | **Low** | **High** | **Low** | **Low** | **Low** | **High** |
| D. M. Hensley (2010) | **Low** | **Low** | **Low** | **High** | **Low** | **Low** | **Low** | **Low** |
| E. Garza-Gonzalez, et al. (2010) | **High** | **Low** | **Low** | **Low** | **Low** | **Low** | **Low** | **Low** |
| F. F. Tuon, et al. (2010) | **Low** | **Low** | **Low** | **Low** | **Low** | **Low** | **Low** | **High** |
| G. Mahajan, et al. (2010) | **High** | **Low** | **Low** | **Low** | **Low** | **Low** | **Low** | **High** |
| G. Samonis, et al. (2010) | **Low** | **Low** | **Low** | **High** | **Low** | **Low** | **Low** | **High** |
| J. S. Esterly, et al. (2010) | **High** | **Low** | **Low** | **High** | **Low** | **Low** | **Low** | **Low** |
| K. Amsler, et al. (2010) | **High** | **High** | **Low** | **High** | **Low** | **Low** | **Low** | **Low** |
| P. D. Mugnier, et al. (2010) | **High** | **Low** | **Low** | **High** | **Low** | **Low** | **Low** | **High** |
| Y. Cai, et al. (2010) | **High** | **High** | **Low** | **High** | **Low** | **Low** | **Low** | **Low** |
| Y. K. Park, et al. (2010) | **High** | **Low** | **Low** | **Low** | **Low** | **High** | **Low** | **Low** |
| Y. Lee, et al. (2010) | **High** | **Low** | **Low** | **High** | **Low** | **High** | **High** | **High** |
| Y.-C. Lin, et al. (2010) | **High** | **High** | **Low** | **High** | **Low** | **High** | **Low** | **High** |
| Z. Mohammadtaheri, et al. (2010) | **High** | **Low** | **Low** | **High** | **Low** | **Low** | **Low** | **High** |
| A. Karunasagar, et al. (2011) | **High** | **Low** | **Low** | **High** | **Low** | **Low** | **Low** | **High** |
| A. Peymani, et al. (2011) | **High** | **Low** | **Low** | **Low** | **Low** | **Low** | **Low** | **High** |
| A. Potron, et al. (2011) | **High** | **Low** | **Low** | **High** | **Low** | **Low** | **Low** | **High** |
| B. Chakraborty, et al. (2011) | **High** | **Low** | **Low** | **Low** | **Low** | **Low** | **Low** | **High** |
| H. Özdemir, et al. (2011) | **High** | **Low** | **Low** | **High** | **Low** | **Low** | **Low** | **High** |
| I. Franolic-Kukina, et al. (2011) | **High** | **Low** | **Low** | **Low** | **Low** | **Low** | **Low** | **High** |
| I. Netsvyetayeva, et al. (2011) | **High** | **Low** | **Low** | **High** | **Low** | **Low** | **High** | **High** |
| K. Asadollahi, et al. (2011) | **High** | **Low** | **Low** | **Low** | **Low** | **Low** | **Low** | **Low** |
| K. Petersen, et al. (2011) | **High** | **Low** | **Low** | **High** | **Low** | **Low** | **Low** | **High** |
| K. S. Shin, et al. (2011) | **High** | **Low** | **Low** | **Low** | **Low** | **Low** | **Low** | **High** |
| M. McCracken, et al. (2011) | **High** | **Low** | **Low** | **High** | **Low** | **High** | **Low** | **High** |
| M. Shanthi, et al. (2011) | **High** | **Low** | **Low** | **Low** | **Low** | **High** | **Low** | **Low** |
| M. Virginia Villegas, et al. (2011) | **High** | **Low** | **Low** | **High** | **Low** | **Low** | **Low** | **Low** |
| N. M. Joseph, et al. (2011) | **High** | **Low** | **Low** | **Low** | **Low** | **High** | **Low** | **Low** |
| P. Nordmann, et al. (2011) | **High** | **Low** | **Low** | **High** | **Low** | **High** | **Low** | **High** |
| S. Japoni, et al. (2011) | **High** | **High** | **Low** | **High** | **Low** | **High** | **High** | **High** |
| S. M. Amudhan, et al. (2011) | **High** | **Low** | **Low** | **High** | **Low** | **Low** | **Low** | **High** |
| S. Mostofi, et al. (2011) | **High** | **Low** | **Low** | **Low** | **Low** | **Low** | **Low** | **High** |
| S.-C. Lee, et al. (2011) | **High** | **Low** | **Low** | **Low** | **Low** | **Low** | **Low** | **Low** |
| T. A. Davies, et al. (2011) | **High** | **High** | **Low** | **High** | **Low** | **Low** | **Low** | **Low** |
| V. Gogou, et al. (2011) | **High** | **Low** | **Low** | **High** | **Low** | **Low** | **Low** | **High** |
| W.-H. Sheng, et al. (2011) | **High** | **Low** | **Low** | **Low** | **Low** | **Low** | **Low** | **Low** |
| A. Chmielarczyk, et al. (2012) | **High** | **Low** | **Low** | **High** | **Low** | **Low** | **Low** | **High** |
| A. Deveci, et al. (2012) | **High** | **Low** | **Low** | **High** | **Low** | **Low** | **Low** | **High** |
| A. G. Ozseven, et al. (2012) | **High** | **Low** | **Low** | **Low** | **Low** | **Low** | **Low** | **Low** |
| A. K. Mostachio, et al. (2012) | **High** | **Low** | **Low** | **Low** | **Low** | **Low** | **Low** | **High** |
| A. M. Somily, et al. (2012) | **High** | **Low** | **Low** | **Low** | **Low** | **Low** | **Low** | **High** |
| A. R. Hamzeh, et al. (2012) | **High** | **High** | **Low** | **High** | **Low** | **Low** | **Low** | **High** |
| B. S. Lopes, et al. (2012) | **High** | **Low** | **Low** | **Low** | **Low** | **Low** | **Low** | **High** |
| B. Zhang, et al. (2012) | **Low** | **Low** | **Low** | **Low** | **Low** | **Low** | **Low** | **High** |
| C. de Souza Gusatti, et al. (2012) | **High** | **High** | **High** | **High** | **Low** | **High** | **Low** | **High** |
| G. Bou, et al. (2012) | **High** | **High** | **Low** | **Low** | **Low** | **Low** | **Low** | **High** |
| G. Samonis, et al. (2012) | **High** | **High** | **Low** | **High** | **Low** | **Low** | **Low** | **High** |
| H. Ziglam, et al. (2012) | **High** | **Low** | **Low** | **Low** | **Low** | **Low** | **Low** | **High** |
| J. Hrabak, et al. (2012) | **High** | **High** | **High** | **High** | **Low** | **Low** | **Low** | **High** |
| J. Martin Llaca-Diaz, et al. (2012) | **High** | **Low** | **Low** | **High** | **Low** | **Low** | **Low** | **Low** |
| K. R. Peck, et al. (2012) | **High** | **Low** | **Low** | **Low** | **Low** | **Low** | **Low** | **High** |
| L. F. Mataseje, et al. (2012) | **High** | **Low** | **Low** | **Low** | **Low** | **Low** | **Low** | **Low** |
| L. Zhong, et al. (2012) | **Low** | **Low** | **Low** | **Low** | **Low** | **Low** | **Low** | **Low** |
| M. Dehghani, et al. (2012) | **High** | **Low** | **Low** | **Low** | **Low** | **Low** | **Low** | **High** |
| M. Purohit, et al. (2012) | **High** | **High** | **Low** | **High** | **Low** | **Low** | **Low** | **High** |
| M. S. Amudhan, et al. (2012) | **High** | **Low** | **Low** | **High** | **Low** | **High** | **Low** | **High** |
| N. A. Al-Sweih, et al. (2012) | **High** | **Low** | **Low** | **Low** | **Low** | **High** | **Low** | **Low** |
| N. Al-Dabaibah, et al. (2012) | **High** | **Low** | **Low** | **Low** | **Low** | **Low** | **Low** | **Low** |
| N. Sohrabi, et al. (2012) | **High** | **Low** | **Low** | **Low** | **Low** | **Low** | **Low** | **High** |
| P. F. Hou, et al. (2012) | **High** | **Low** | **Low** | **Low** | **Low** | **Low** | **Low** | **Low** |
| P. Martinez, et al. (2012) | **High** | **Low** | **Low** | **Low** | **Low** | **Low** | **Low** | **High** |
| P. S. Shareek, et al. (2012) | **High** | **High** | **Low** | **High** | **Low** | **Low** | **Low** | **High** |
| P. Sacha, et al. (2012) | **High** | **High** | **Low** | **High** | **Low** | **Low** | **Low** | **Low** |
| Q. Zhong, et al. (2012) | **High** | **Low** | **Low** | **Low** | **Low** | **Low** | **Low** | **High** |
| R. Morfin-Otero, et al. (2012) | **High** | **Low** | **Low** | **Low** | **Low** | **Low** | **Low** | **Low** |
| S. Figueiredo, et al. (2012) | **High** | **Low** | **Low** | **High** | **Low** | **Low** | **Low** | **High** |
| S. Maraki, et al. (2012) | **High** | **Low** | **Low** | **High** | **Low** | **High** | **Low** | **High** |
| S.-X. Dong, et al. (2012) | **High** | **Low** | **Low** | **Low** | **Low** | **High** | **Low** | **High** |
| Y. J. Kim, et al. (2012) | **High** | **Low** | **Low** | **Low** | **Low** | **Low** | **Low** | **High** |
| Y. K. Park, et al. (2012) | **High** | **Low** | **Low** | **Low** | **Low** | **Low** | **Low** | **High** |
| A. A. Alsultan, et al. (2013) | **Low** | **Low** | **Low** | **High** | **Low** | **Low** | **Low** | **High** |
| A. Balode, et al. (2013) | **Low** | **Low** | **Low** | **Low** | **Low** | **Low** | **Low** | **High** |
| A. Ergin, et al. (2013) | **High** | **Low** | **Low** | **Low** | **Low** | **Low** | **Low** | **Low** |
| A. Karmostaj, et al. (2013) | **High** | **Low** | **Low** | **Low** | **Low** | **Low** | **Low** | **High** |
| A. Karmostaji, et al. (2013) | **High** | **Low** | **Low** | **Low** | **Low** | **Low** | **Low** | **High** |
| A. M. Asaad, et al. (2013) | **High** | **Low** | **Low** | **Low** | **Low** | **Low** | **Low** | **Low** |
| A. Sonnevend, et al. (2013) | **High** | **Low** | **Low** | **Low** | **Low** | **Low** | **Low** | **High** |
| A. T. ÇIÇek, et al. (2013) | **High** | **Low** | **Low** | **High** | **Low** | **Low** | **Low** | **High** |
| B. Hasan, et al. (2013) | **High** | **Low** | **Low** | **Low** | **Low** | **High** | **Low** | **High** |
| C. G. Carvalhaes, et al. (2013) | **High** | **High** | **Low** | **Low** | **Low** | **Low** | **Low** | **High** |
| C. Moon, et al. (2013) | **Low** | **Low** | **Low** | **High** | **Low** | **Low** | **Low** | **High** |
| C.-M. Lee, et al. (2013) | **High** | **Low** | **Low** | **High** | **Low** | **High** | **Low** | **High** |
| D. K. Tiwari, et al. (2013) | **High** | **Low** | **Low** | **Low** | **Low** | **Low** | **Low** | **High** |
| E. L. Fonseca, et al. (2013) | **High** | **Low** | **Low** | **Low** | **Low** | **High** | **Low** | **High** |
| F. Pei, et al. (2013) | **High** | **Low** | **Low** | **Low** | **Low** | **Low** | **Low** | **High** |
| G. F. Viana, et al. (2013) | **High** | **High** | **Low** | **Low** | **Low** | **Low** | **Low** | **Low** |
| G. Peirano, et al. (2013) | **Low** | **Low** | **Low** | **High** | **Low** | **Low** | **Low** | **High** |
| I. N. Hakyemez, et al. (2013) | **High** | **Low** | **Low** | **High** | **Low** | **Low** | **Low** | **High** |
| J. Huang, et al. (2013) | **Low** | **Low** | **High** | **High** | **Low** | **Low** | **Low** | **High** |
| K. Sieniawski, et al. (2013) | **High** | **Low** | **Low** | **High** | **High** | **Low** | **High** | **High** |
| L. Krizova, et al. (2013) | **High** | **Low** | **Low** | **High** | **Low** | **Low** | **Low** | **High** |
| M. Chaudhary, et al. (2013) | **High** | **Low** | **Low** | **Low** | **Low** | **Low** | **Low** | **High** |
| M. Fouad, et al. (2013) | **High** | **High** | **High** | **High** | **Low** | **Low** | **Low** | **High** |
| M. Hornsey, et al. (2013) | **High** | **Low** | **Low** | **Low** | **Low** | **Low** | **Low** | **High** |
| M. M. Kock, et al. (2013) | **High** | **High** | **Low** | **High** | **Low** | **Low** | **High** | **High** |
| M. Safari, et al. (2013) | **High** | **Low** | **Low** | **Low** | **Low** | **Low** | **Low** | **Low** |
| M. Teresa Reguero, et al. (2013) | **High** | **Low** | **Low** | **Low** | **Low** | **Low** | **Low** | **High** |
| M.-H. Lee, et al. (2013) | **High** | **Low** | **Low** | **High** | **Low** | **Low** | **Low** | **Low** |
| N. Jumroon, et al. (2013) | **High** | **Low** | **Low** | **Low** | **Low** | **Low** | **Low** | **High** |
| P. E. Waterman, et al. (2013) | **Low** | **Low** | **Low** | **High** | **Low** | **Low** | **Low** | **High** |
| P. Espinal, et al. (2013) | **Low** | **Low** | **Low** | **High** | **Low** | **Low** | **Low** | **High** |
| P. Mohajeri, et al. (2013) | **High** | **Low** | **Low** | **High** | **Low** | **Low** | **Low** | **High** |
| Paj, et al. (2013) | **High** | **Low** | **Low** | **Low** | **Low** | **Low** | **Low** | **High** |
| Q. Fu, et al. (2013) | **High** | **High** | **High** | **High** | **Low** | **Low** | **Low** | **High** |
| R. Mirnejad, et al. (2013) | **High** | **Low** | **Low** | **Low** | **Low** | **Low** | **Low** | **High** |
| R. Morfin-Otero, et al. (2013) | **High** | **Low** | **Low** | **Low** | **Low** | **Low** | **Low** | **High** |
| S. A. Clock, et al. (2013) | **High** | **Low** | **Low** | **Low** | **Low** | **Low** | **Low** | **Low** |
| S. D. Kotsakis, et al. (2013) | **High** | **Low** | **Low** | **High** | **Low** | **Low** | **Low** | **High** |
| S. Dally, et al. (2013) | **Low** | **Low** | **Low** | **Low** | **Low** | **High** | **Low** | **High** |
| S. Niakan, et al. (2013) | **High** | **Low** | **Low** | **High** | **Low** | **Low** | **Low** | **High** |
| S. Notake, et al. (2013) | **High** | **Low** | **Low** | **High** | **High** | **Low** | **Low** | **High** |
| S. Park, et al. (2013) | **High** | **Low** | **Low** | **Low** | **Low** | **Low** | **Low** | **High** |
| S.-S. Jean, et al. (2013) | **High** | **Low** | **Low** | **Low** | **Low** | **Low** | **Low** | **Low** |
| T. Xu, et al. (2013) | **High** | **Low** | **Low** | **Low** | **Low** | **Low** | **Low** | **High** |
| X. Wang, et al. (2013) | **High** | **Low** | **Low** | **High** | **Low** | **Low** | **Low** | **High** |
| Y. J. Li, et al. (2013) | **High** | **Low** | **Low** | **Low** | **Low** | **Low** | **Low** | **High** |
| Y. S. Huang, et al. (2013) | **High** | **Low** | **Low** | **High** | **Low** | **Low** | **Low** | **Low** |
| Z. Chen, et al. (2013) | **High** | **Low** | **Low** | **Low** | **Low** | **Low** | **Low** | **High** |
| Z. Ruan, et al. (2013) | **High** | **Low** | **Low** | **High** | **Low** | **Low** | **Low** | **High** |
| A. C. Cicek, et al. (2014) | **Low** | **Low** | **Low** | **Low** | **Low** | **Low** | **Low** | **High** |
| B. Abdalhamid, et al. (2014) | **High** | **Low** | **Low** | **Low** | **Low** | **Low** | **Low** | **High** |
| B. Norozi, et al. (2014) | **Low** | **Low** | **Low** | **Low** | **Low** | **Low** | **Low** | **Low** |
| B. Todorova, et al. (2014) | **Low** | **Low** | **Low** | **Low** | **Low** | **Low** | **Low** | **Low** |
| E. Kirkgoz, et al. (2014) | **Low** | **Low** | **Low** | **Low** | **Low** | **Low** | **Low** | **High** |
| F. Fallah, et al. (2014) | **Low** | **Low** | **Low** | **Low** | **Low** | **Low** | **Low** | **High** |
| G. Aşik, et al. (2014) | **High** | **High** | **Low** | **Low** | **Low** | **Low** | **Low** | **High** |
| H. S. I. Martins, et al. (2014) | **Low** | **Low** | **Low** | **Low** | **Low** | **Low** | **Low** | **High** |
| H. U. Altun, et al. (2014) | **Low** | **Low** | **Low** | **Low** | **Low** | **Low** | **Low** | **High** |
| K. Jeannot, et al. (2014) | **Low** | **Low** | **Low** | **Low** | **Low** | **High** | **Low** | **High** |
| L. Principe, et al. (2014) | **Low** | **Low** | **Low** | **Low** | **Low** | **Low** | **Low** | **Low** |
| M. Chaudhary, et al. (2014) | **High** | **Low** | **Low** | **Low** | **Low** | **High** | **Low** | **High** |
| M. Dolores Alcantar-Curiel, et al. (2014) | **Low** | **Low** | **Low** | **Low** | **Low** | **Low** | **Low** | **High** |
| M. Nasrolahei, et al. (2014) | **Low** | **Low** | **Low** | **Low** | **Low** | **Low** | **Low** | **Low** |
| M. Vranic-Ladavac, et al. (2014) | **Low** | **Low** | **Low** | **Low** | **Low** | **Low** | **Low** | **Low** |
| O. O. Gundeslioglu, et al. (2014) | **Low** | **Low** | **Low** | **Low** | **Low** | **Low** | **Low** | **High** |
| R. Soltani, et al. (2014) | **Low** | **Low** | **Low** | **Low** | **Low** | **Low** | **Low** | **Low** |
| S. Mendoza-Olazaran, et al. (2014) | **Low** | **Low** | **Low** | **Low** | **Low** | **Low** | **Low** | **High** |
| S. S. Akcay, et al. (2014) | **High** | **Low** | **Low** | **Low** | **Low** | **Low** | **Low** | **High** |
| S.-S. Lean, et al. (2014) | **Low** | **Low** | **Low** | **Low** | **Low** | **Low** | **Low** | **Low** |
| Sjol, et al. (2014) | **Low** | **Low** | **Low** | **Low** | **Low** | **Low** | **Low** | **Low** |
| T. D. Van, et al. (2014) | **Low** | **Low** | **Low** | **Low** | **High** | **Low** | **Low** | **Low** |
| T. P. Gomes Chagas, et al. (2014) | **Low** | **Low** | **Low** | **Low** | **High** | **Low** | **Low** | **High** |
| T. Tada, et al. (2014) | **Low** | **Low** | **Low** | **Low** | **Low** | **Low** | **Low** | **High** |
| W. Nageeb, et al. (2014) | **Low** | **Low** | **Low** | **Low** | **Low** | **Low** | **Low** | **High** |
| A. Slavcovici, et al. (2015) | **Low** | **Low** | **Low** | **High** | **Low** | **Low** | **Low** | **Low** |
| A. Spiliopoulou, et al. (2015) | **Low** | **Low** | **High** | **Low** | **Low** | **Low** | **Low** | **Low** |
| A. Yoshizumi, et al. (2015) | **Low** | **Low** | **Low** | **High** | **High** | **Low** | **Low** | **Some** |
| C. Hou, et al. (2015) | **Low** | **Low** | **Low** | **Low** | **Low** | **Low** | **Low** | **Low** |
| D. J. Hoban, et al. (2015) | **Low** | **Low** | **Low** | **Low** | **Low** | **Low** | **Low** | **Low** |
| F. Gao, et al. (2015) | **Low** | **Low** | **Low** | **Low** | **Low** | **Low** | **Low** | **Low** |
| Ghajav, et al. (2015) | **Low** | **Low** | **Low** | **Low** | **Low** | **Low** | **Low** | **Low** |
| H. Chen, et al. (2015) | **Low** | **Low** | **Low** | **Low** | **Low** | **Low** | **Low** | **Low** |
| H. Goudarzi, et al. (2015) | **Low** | **Low** | **Low** | **High** | **High** | **Low** | **Low** | **Some** |
| M. Gholami, et al. (2015) | **Low** | **Low** | **Low** | **Low** | **Low** | **Low** | **Low** | **Low** |
| M. Gilani, et al. (2015) | **Low** | **Low** | **Low** | **Low** | **Low** | **Low** | **Low** | **Low** |
| M. Gilani, et al. (2015) | **Low** | **Low** | **Low** | **Low** | **Low** | **Low** | **Low** | **Low** |
| M. Lowings, et al. (2015) | **Low** | **Low** | **Low** | **Low** | **Low** | **Low** | **Low** | **Low** |
| M. S. Siqueira, et al. (2015) | **Low** | **Low** | **Low** | **High** | **High** | **Low** | **Low** | **Low** |
| N. Mathlouthi, et al. (2015) | **Low** | **Low** | **Low** | **Low** | **Low** | **Low** | **Low** | **Low** |
| O. Azizi, et al. (2015) | **Low** | **Low** | **Low** | **Low** | **Low** | **Low** | **Low** | **Low** |
| P. Bocanegra-Ibarias, et al. (2015) | **Low** | **Low** | **Low** | **Low** | **Low** | **Low** | **Low** | **Low** |
| S. Bagheri Josheghani, et al. (2015) | **Low** | **Low** | **Low** | **Low** | **Low** | **Low** | **Low** | **Low** |
| S. Biglari, et al. (2015) | **Low** | **Low** | **Low** | **Low** | **Low** | **Low** | **Low** | **Low** |
| Ş. Direkel, et al. (2015) | **Low** | **Low** | **Low** | **Low** | **Low** | **Low** | **Low** | **Low** |
| S. Kooti, et al. (2015) | **Low** | **Low** | **Low** | **Low** | **Low** | **Low** | **Low** | **Low** |
| S. Mahdian, et al. (2015) | **Low** | **Low** | **Low** | **Low** | **Low** | **Low** | **Low** | **Low** |
| S. N. Peerayeh, et al. (2015) | **Low** | **Low** | **Low** | **Low** | **Low** | **High** | **Low** | **Low** |
| S. Shrestha, et al. (2015) | **Low** | **Low** | **High** | **High** | **Low** | **Low** | **Low** | **Low** |
| T. Elkersh, et al. (2015) | **Low** | **Low** | **Low** | **Low** | **High** | **Low** | **Low** | **Low** |
| X. M. Nie, et al. (2015) | **Low** | **Low** | **Low** | **Low** | **High** | **Low** | **Low** | **Low** |
| X. Zhang, et al. (2015) | **Low** | **Low** | **Low** | **Low** | **Low** | **Low** | **Some** | **Low** |
| Y. Li, et al. (2015) | **Low** | **Low** | **Low** | **Low** | **High** | **Low** | **Low** | **Low** |
| Y. Yang, et al. (2015) | **Low** | **Low** | **Low** | **Low** | **Low** | **Low** | **Low** | **Low** |
| Z. Ma, et al. (2015) | **Low** | **Low** | **Low** | **Low** | **Low** | **Low** | **Low** | **Low** |
| A. Chmielarczyk, et al. (2016) | **Low** | **Low** | **Low** | **Low** | **Low** | **Low** | **Low** | **Low** |
| D. J. Biedenbach, et al. (2016) | **Low** | **Low** | **Low** | **Low** | **Low** | **Low** | **Low** | **Low** |
| D. P. Kateete, et al. (2016) | **High** | **Low** | **Low** | **Low** | **Low** | **Low** | **Low** | **High** |
| F. E. Al-Otaibi, et al. (2016) | **Low** | **Low** | **Low** | **Low** | **Low** | **Low** | **Low** | **High** |
| F. Fang, et al. (2016) | **Low** | **Low** | **Low** | **Low** | **Low** | **Low** | **Low** | **Low** |
| F. Marco, et al. (2016) | **Low** | **Low** | **Low** | **High** | **Low** | **Low** | **Low** | **Low** |
| F. P. Hu, et al. (2016) | **Low** | **Low** | **Low** | **Low** | **Low** | **Low** | **Low** | **Low** |
| G. Odewale, et al. (2016) | **Low** | **Low** | **Low** | **High** | **Low** | **Low** | **Low** | **Low** |
| H. Maspi, et al. (2016) | **Low** | **Low** | **Low** | **Low** | **Low** | **Low** | **Low** | **Low** |
| H.-A. Terzi, et al. (2016) | **Low** | **Low** | **Low** | **Low** | **Low** | **Low** | **High** | **Low** |
| H.-R. Lin, et al. (2016) | **Low** | **Low** | **Low** | **Low** | **Low** | **Low** | **Low** | **Low** |
| K. K. Ghaima, et al. (2016) | **Low** | **Low** | **Low** | **Low** | **Low** | **High** | **Low** | **Low** |
| M. Anwar, et al. (2016) | **Low** | **Low** | **Low** | **Low** | **Low** | **Low** | **Low** | **Low** |
| M. D. Zilberberg, et al. (2016) | **Low** | **Low** | **Low** | **High** | **High** | **Low** | **Low** | **Low** |
| M. N. Moghadam, et al. (2016) | **Low** | **Low** | **Low** | **Low** | **Low** | **Low** | **Low** | **Low** |
| R. Chen, et al. (2016) | **Low** | **Low** | **Low** | **Low** | **Low** | **Low** | **Low** | **Low** |
| S. Chatterjee, et al. (2016) | **Low** | **Low** | **Low** | **Low** | **High** | **Low** | **Low** | **Low** |
| S. Direkel, et al. (2016) | **Low** | **Low** | **Low** | **High** | **High** | **Low** | **Low** | **Low** |
| S. Maraki, et al. (2016) | **Low** | **Low** | **Low** | **Low** | **Low** | **Low** | **Low** | **Low** |
| S. S. Ahmed, et al. (2016) | **Low** | **Low** | **Low** | **Low** | **Low** | **Low** | **High** | **High** |
| Y. Sun, et al. (2016) | **Low** | **Low** | **Low** | **Low** | **Low** | **Low** | **Low** | **Low** |
| Y. Zhou, et al. (2016) | **Low** | **Low** | **Low** | **Low** | **Low** | **Low** | **Low** | **Low** |
| Y.-m. Xu, et al. (2016) | **Low** | **Low** | **Low** | **Low** | **Low** | **Low** | **Low** | **Low** |
| Z. Yürüken, et al. (2016) | **Low** | **Low** | **Low** | **Low** | **Low** | **Low** | **Low** | **High** |
| A. Guzek, et al. (2017) | **Low** | **Low** | **Low** | **Low** | **Low** | **Low** | **Low** | **Low** |
| A. P. Petrova, et al. (2017) | **Low** | **Low** | **Low** | **Low** | **Low** | **Low** | **Low** | **Low** |
| D. P. Kateete, et al. (2017) | **High** | **Low** | **High** | **High** | **Low** | **Low** | **High** | **High** |
| E. Uzunoglu, et al. (2017) | **Low** | **Low** | **Low** | **Low** | **Low** | **High** | **Low** | **Some** |
| E.-J. Yoon, et al. (2017) | **High** | **Low** | **Low** | **Low** | **Low** | **Low** | **Low** | **Low** |
| F. Ahmadikiya, et al. (2017) | **Low** | **Low** | **Low** | **Low** | **Low** | **Low** | **Low** | **Low** |
| F. Akrami, et al. (2017) | **Low** | **Low** | **Low** | **Low** | **Low** | **Low** | **Low** | **Low** |
| F. Khamesipour, et al. (2017) | **Low** | **Low** | **Low** | **Low** | **Low** | **Low** | **Low** | **Low** |
| F. Mohammadi, et al. (2017) | **Low** | **Low** | **Low** | **Low** | **Low** | **Low** | **Low** | **High** |
| G. M. Tran, et al. (2017) | **Low** | **Low** | **Low** | **Low** | **Low** | **Low** | **Low** | **Low** |
| H. N. Wen, et al. (2017) | **Low** | **Low** | **High** | **High** | **Low** | **Low** | **High** | **High** |
| J. H. Woo, et al. (2017) | **High** | **Low** | **Low** | **Low** | **Low** | **Low** | **Low** | **Low** |
| L. Han, et al. (2017) | **Low** | **Low** | **Low** | **Low** | **Low** | **Low** | **Low** | **Low** |
| M. Mohammadi, et al. (2017) | **Low** | **Low** | **Low** | **Low** | **Low** | **Low** | **Low** | **Low** |
| M. Moosavian, et al. (2017) | **Low** | **Low** | **Low** | **Low** | **Low** | **Low** | **Low** | **High** |
| N. Ceyhan-Guvensen, et al. (2017) | **Low** | **Low** | **Low** | **Low** | **Low** | **Low** | **Low** | **Low** |
| P. H. Van, et al. (2017) | **Low** | **Low** | **Low** | **High** | **Low** | **Low** | **Low** | **Low** |
| P. Vu Dinh, et al. (2017) | **Low** | **Low** | **Low** | **High** | **Low** | **Low** | **Low** | **Low** |
| R. A. R. Aziz, et al. (2017) | **Low** | **Low** | **Low** | **Low** | **Low** | **Low** | **Low** | **Low** |
| R. Rosales-Reyes, et al. (2017) | **Low** | **Low** | **Low** | **Low** | **Low** | **Low** | **Low** | **Low** |
| S. Agarwal, et al. (2017) | **Low** | **Low** | **Low** | **Low** | **Low** | **Low** | **Low** | **Low** |
| S. Biglari, et al. (2017) | **Low** | **Low** | **Low** | **Low** | **Low** | **Low** | **Low** | **Low** |
| S. Chusri, et al. (2017) | **Low** | **Low** | **Low** | **Low** | **Low** | **Low** | **Low** | **High** |
| X. Wang, et al. (2017) | **Low** | **Low** | **Low** | **High** | **Low** | **Low** | **Low** | **Low** |
| X. Zhen, et al. (2017) | **Low** | **Low** | **Low** | **High** | **Low** | **Low** | **Low** | **Low** |
| X.-g. Hu, et al. (2017) | **Low** | **Low** | **Low** | **Low** | **Low** | **High** | **Low** | **Low** |
| X.-m. Xiao, et al. (2017) | **Low** | **Low** | **Low** | **Low** | **Low** | **High** | **Low** | **Low** |
| A. Alamri, et al. (2018) | **Low** | **Low** | **Low** | **High** | **Low** | **Low** | **Low** | **Low** |
| A. Kaur, et al. (2018) | **Low** | **Low** | **Low** | **Low** | **Low** | **Low** | **Low** | **Low** |
| A. M. Sultan, et al. (2018) | **Low** | **Low** | **Low** | **High** | **High** | **Low** | **Low** | **Low** |
| A. Ramette, et al. (2018) | **Low** | **Low** | **High** | **High** | **High** | **Low** | **High** | **Low** |
| A. Rezaei, et al. (2018) | **Low** | **Low** | **Low** | **Low** | **High** | **Low** | **Low** | **Low** |
| B. Salehi, et al. (2018) | **Low** | **Low** | **Low** | **Low** | **Low** | **Low** | **High** | **Low** |
| B. Soltani, et al. (2018) | **Low** | **Low** | **Low** | **Low** | **Low** | **Low** | **High** | **Low** |
| C. Hu, et al. (2018) | **Low** | **Low** | **Low** | **Low** | **Low** | **Low** | **Low** | **Low** |
| C. Vuotto, et al. (2018) | **Low** | **Low** | **Low** | **Low** | **Low** | **Low** | **Low** | **Low** |
| D. M. Khan, et al. (2018) | **Low** | **Low** | **Low** | **Low** | **Low** | **Low** | **Low** | **Low** |
| F. Akin, et al. (2018) | **Low** | **Low** | **Low** | **High** | **Low** | **Low** | **Low** | **High** |
| F. M. Rani, et al. (2018) | **Low** | **Low** | **Low** | **Low** | **Low** | **Low** | **Low** | **Low** |
| G. Jimenez-Guerra, et al. (2018) | **Low** | **Low** | **Low** | **Low** | **Low** | **Low** | **Low** | **Low** |
| G. Ziolkowski, et al. (2018) | **Low** | **Low** | **Low** | **Low** | **Low** | **Low** | **Low** | **Low** |
| H. G. Wu, et al. (2018) | **Low** | **Low** | **Low** | **Low** | **Low** | **Low** | **Low** | **Low** |
| H. Khalili, et al. (2018) | **Low** | **Low** | **Low** | **Low** | **Low** | **Low** | **Low** | **Low** |
| H. Lee, et al. (2018) | **Low** | **Low** | **Low** | **Low** | **Low** | **Low** | **Low** | **Low** |
| H. Seifert, et al. (2018) | **Low** | **Low** | **Low** | **High** | **Low** | **Low** | **Low** | **Low** |
| I. Bado, et al. (2018) | **Low** | **Low** | **Low** | **Low** | **Low** | **Low** | **Low** | **Low** |
| J. Akhtar, et al. (2018) | **Low** | **Low** | **Low** | **Low** | **Low** | **Low** | **Low** | **Low** |
| J. Armalyte, et al. (2018) | **Low** | **High** | **High** | **High** | **Low** | **Low** | **Low** | **High** |
| J. E. Sam, et al. (2018) | **Low** | **Low** | **High** | **High** | **Low** | **Low** | **High** | **Low** |
| J. Tian, et al. (2018) | **Low** | **Low** | **Low** | **Low** | **Low** | **Low** | **Low** | **Low** |
| K. Dafopoulou, et al. (2018) | **Low** | **Low** | **Low** | **Low** | **Low** | **Low** | **Low** | **Low** |
| K. F. Schuertz, et al. (2018) | **Low** | **Low** | **Low** | **Low** | **Low** | **Low** | **Low** | **Low** |
| L. Fan, et al. (2018) | **Low** | **Low** | **Low** | **Low** | **Low** | **Low** | **Low** | **Low** |
| L. Yang, et al. (2018) | **Low** | **Low** | **Low** | **Low** | **Low** | **High** | **Low** | **High** |
| M. A. Dehbalaei, et al. (2018) | **Low** | **Low** | **Low** | **Low** | **Low** | **Low** | **High** | **Low** |
| M. Babu, et al. (2018) | **Low** | **Low** | **Low** | **Low** | **Low** | **Low** | **Low** | **Low** |
| M. Cerezales, et al. (2018) | **Low** | **Low** | **Low** | **Low** | **Low** | **Low** | **Low** | **High** |
| M. Gholami, et al. (2018) | **Low** | **Low** | **Low** | **Low** | **Low** | **Low** | **Low** | **Low** |
| M. H. Soudeiha, et al. (2018) | **High** | **Low** | **Low** | **Low** | **Low** | **Low** | **High** | **High** |
| M. Jajoo, et al. (2018) | **Low** | **Low** | **Low** | **Low** | **High** | **Low** | **Low** | **Low** |
| M. K. Almaghrabi, et al. (2018) | **Low** | **Low** | **High** | **High** | **Low** | **Low** | **High** | **High** |
| M. Lowe, et al. (2018) | **Low** | **Low** | **Low** | **Low** | **Low** | **Low** | **Low** | **Low** |
| M. Matsui, et al. (2018) | **Low** | **Low** | **Low** | **Low** | **Low** | **Low** | **Low** | **Low** |
| M. Rahman, et al. (2018) | **Low** | **Low** | **Low** | **Low** | **Low** | **Low** | **Low** | **Low** |
| M. S. Alhaddad, et al. (2018) | **Low** | **Low** | **Low** | **Low** | **Low** | **Low** | **Low** | **Low** |
| M. Sadeghi-Haddad-Zavareh, et al. (2018) | **Low** | **Low** | **Low** | **Low** | **Low** | **Low** | **Low** | **Low** |
| M. T. S. Al-Ouqaili, et al. (2018) | **Low** | **Low** | **Low** | **High** | **Low** | **Low** | **Low** | **High** |
| N. Jaidane, et al. (2018) | **Low** | **Low** | **Low** | **Low** | **Low** | **Low** | **Low** | **Low** |
| N. S. Ravi, et al. (2018) | **Low** | **Low** | **Low** | **Low** | **Low** | **Low** | **Low** | **Low** |
| O. Perovic, et al. (2018) | **Low** | **High** | **High** | **High** | **Low** | **Low** | **High** | **High** |
| P. Mule, et al. (2018) | **Low** | **Low** | **Low** | **Low** | **Low** | **Low** | **Low** | **Low** |
| Q. Chen, et al. (2018) | **High** | **Low** | **Low** | **Low** | **Low** | **Low** | **Low** | **High** |
| R. A. Al-Hindawi, et al. (2018) | **Some** | **Some** | **Some** | **Low** | **Some** | **Some** | **Low** | **Some** |
| R. A. Ramadan, et al. (2018) | **Low** | **Low** | **Low** | **Low** | **Low** | **Low** | **Low** | **Low** |
| R. Aljindan, et al. (2018) | **Low** | **Low** | **Low** | **Low** | **Low** | **Low** | **Low** | **Low** |
| S. A. S. Girija, et al. (2018) | **Low** | **Low** | **Low** | **Low** | **Low** | **Low** | **Low** | **High** |
| S. J. Nigro, et al. (2018) | **Low** | **Low** | **Low** | **Some** | **Low** | **Low** | **Low** | **Low** |
| S. Levy-Blitchtein, et al. (2018) | **High** | **Low** | **Low** | **Low** | **Low** | **Low** | **Low** | **Low** |
| S. Nishida, et al. (2018) | **Low** | **Low** | **Low** | **Low** | **Low** | **Low** | **Low** | **Low** |
| S. Pan, et al. (2018) | **Low** | **Low** | **Low** | **Low** | **Low** | **Low** | **Low** | **Low** |
| T. K. Atik, et al. (2018) | **Low** | **Low** | **Low** | **Low** | **Low** | **Low** | **High** | **High** |
| U. Devi, et al. (2018) | **Low** | **Low** | **Low** | **Low** | **Low** | **Low** | **Low** | **Low** |
| U. Leungtongkam, et al. (2018) | **Low** | **Low** | **Low** | **Low** | **Low** | **Low** | **Low** | **Low** |
| U. Singkham-in, et al. (2018) | **Low** | **Low** | **Low** | **Low** | **Low** | **Low** | **Low** | **Low** |
| V. Cafiso, et al. (2018) | **High** | **Low** | **Low** | **Low** | **Low** | **Low** | **Low** | **High** |
| W. Duszynska, et al. (2018) | **Low** | **Low** | **Low** | **Low** | **Low** | **Low** | **Low** | **Low** |
| W.-m. Liu, et al. (2018) | **Low** | **Low** | **High** | **Low** | **Low** | **Low** | **Low** | **Low** |
| X. Tang, et al. (2018) | **Low** | **Low** | **Low** | **Low** | **Low** | **Low** | **Low** | **Low** |
| Y. Chen, et al. (2018) | **Low** | **Low** | **Low** | **Low** | **Low** | **Low** | **Low** | **Low** |
| Y. Li, et al. (2018) | **Low** | **Low** | **High** | **High** | **Low** | **Low** | **High** | **Low** |
| Y. Pan, et al. (2018) | **Low** | **Low** | **Low** | **Low** | **Some** | **Some** | **Low** | **Some** |
| Y. Ramsamy, et al. (2018) | **Low** | **Low** | **Low** | **Low** | **Low** | **Low** | **Low** | **Low** |
| Z. Moazzen, et al. (2018) | **Low** | **Low** | **Low** | **Low** | **Low** | **Low** | **Low** | **Low** |
| Z. Zhang, et al. (2018) | **Low** | **Low** | **Low** | **Low** | **Low** | **Low** | **Low** | **Low** |
| A. Balkhair, et al. (2019) | **Low** | **Low** | **Low** | **Low** | **Low** | **Low** | **Low** | **Low** |
| A. H. Uc-Cachon, et al. (2019) | **Low** | **Low** | **Low** | **Low** | **Low** | **Low** | **Low** | **Low** |
| A. K. R. Purba, et al. (2019) | **Low** | **Low** | **Low** | **High** | **Low** | **Low** | **Low** | **Low** |
| A. Miller, et al. (2019) | **High** | **Low** | **Low** | **High** | **Low** | **Low** | **Low** | **Low** |
| A. Nazir (2019) | **Low** | **Low** | **Low** | **Low** | **Low** | **Low** | **Low** | **Low** |
| A. S. Shahari, et al. (2019) | **Low** | **Low** | **Low** | **Low** | **Low** | **Low** | **Low** | **Low** |
| A. Sedaghat, et al. (2019) | **Low** | **Low** | **Low** | **High** | **Low** | **Low** | **Low** | **Low** |
| A. Wolfensberger, et al. (2019) | **Low** | **Low** | **Low** | **Low** | **Low** | **Low** | **Low** | **Low** |
| C. Liu, et al. (2019) | **Low** | **Low** | **Low** | **Low** | **Low** | **Low** | **Low** | **Low** |
| C. Rocha, et al. (2019) | **Low** | **Low** | **Low** | **Low** | **Low** | **High** | **Low** | **Low** |
| C.-C. Lai, et al. (2019) | **Low** | **Low** | **Low** | **Low** | **Low** | **High** | **Low** | **Low** |
| D. Usjak, et al. (2019) | **Low** | **Low** | **Low** | **Low** | **Low** | **Low** | **Low** | **Low** |
| D. Viderman, et al. (2019) | **Low** | **Low** | **Low** | **High** | **Low** | **Low** | **Low** | **High** |
| E. A. de Oliveira, et al. (2019) | **Low** | **Low** | **High** | **High** | **Low** | **Low** | **Low** | **High** |
| E. C.-m. Leung, et al. (2019) | **Low** | **Low** | **Low** | **High** | **Low** | **Low** | **Low** | **Low** |
| E. Riccobono, et al. (2019) | **Low** | **Low** | **Low** | **High** | **Low** | **Low** | **Low** | **High** |
| F. I. Sonbol, et al. (2019) | **Low** | **Low** | **Low** | **Low** | **Low** | **Low** | **Low** | **Low** |
| F. K. S. F. de Azevedo, et al. (2019) | **Low** | **Low** | **Low** | **Low** | **Low** | **Low** | **Low** | **Low** |
| F. Moges, et al. (2019) | **Low** | **Low** | **Low** | **Low** | **Low** | **Low** | **Low** | **Low** |
| F. Zhang, et al. (2019) | **Low** | **Low** | **Low** | **Low** | **Low** | **Low** | **Low** | **Low** |
| G. Ayar, et al. (2019) | **Low** | **Low** | **Low** | **High** | **Low** | **Low** | **Low** | **Low** |
| G. M. M. Abesamis, et al. (2019) | **Low** | **Low** | **Some** | **Some** | **Low** | **Low** | **Some** | **Some** |
| H. Ari, et al. (2019) | **Low** | **Low** | **Low** | **High** | **Low** | **Low** | **Low** | **High** |
| H. Ismail, et al. (2019) | **Low** | **Low** | **Low** | **Low** | **Low** | **Low** | **Low** | **Low** |
| I. Gheorghe, et al. (2019) | **Low** | **Low** | **High** | **Low** | **Low** | **Low** | **Low** | **Low** |
| J. M. Pogue, et al. (2019) | **High** | **Low** | **High** | **High** | **Low** | **Low** | **High** | **High** |
| J. Nadia, et al. (2019) | **Low** | **Low** | **Low** | **High** | **Low** | **Low** | **Low** | **Low** |
| J.-D. Lu, et al. (2019) | **Low** | **Low** | **High** | **High** | **Low** | **Low** | **High** | **High** |
| L. Al-Hassan, et al. (2019) | **Low** | **Low** | **Low** | **Low** | **Low** | **Low** | **Low** | **High** |
| L.-j. Liu, et al. (2019) | **Low** | **Low** | **Low** | **Low** | **Low** | **Low** | **Low** | **Low** |
| L.-J. Zhu, et al. (2019) | **Low** | **Low** | **High** | **Low** | **Low** | **Low** | **Low** | **High** |
| M. Amin, et al. (2019) | **Low** | **Low** | **Low** | **Low** | **Low** | **Low** | **Low** | **Low** |
| M. Cerezales, et al. (2019) | **Low** | **Low** | **Low** | **Low** | **Low** | **High** | **Low** | **Low** |
| M. Dolores Alcantar-Curiel, et al. (2019) | **Low** | **Low** | **Low** | **Low** | **Low** | **Low** | **Low** | **Low** |
| M. Eslami, et al. (2019) | **Low** | **Low** | **Low** | **Low** | **Low** | **High** | **Low** | **Low** |
| M. F. El-Badawy, et al. (2019) | **Low** | **Low** | **Low** | **Low** | **Low** | **Low** | **Low** | **High** |
| M. Giannella, et al. (2019) | **Low** | **Low** | **Low** | **Low** | **Low** | **Low** | **Low** | **Low** |
| M. H.-y. Wong, et al. (2019) | **Low** | **Low** | **Low** | **Low** | **Low** | **Low** | **Low** | **Low** |
| M. Jain, et al. (2019) | **Low** | **Low** | **Low** | **Low** | **Low** | **Low** | **Low** | **High** |
| M. Kumari, et al. (2019) | **Low** | **Low** | **Low** | **High** | **Low** | **Low** | **Low** | **Low** |
| M. Mhondoro, et al. (2019) | **Low** | **Low** | **Low** | **High** | **Low** | **Low** | **Low** | **Low** |
| M. Noori, et al. (2019) | **Low** | **Low** | **High** | **Low** | **Low** | **Low** | **Low** | **Low** |
| M. Tamburro, et al. (2019) | **High** | **High** | **Low** | **Low** | **High** | **Low** | **Low** | **High** |
| M. W. Shah, et al. (2019) | **Low** | **Low** | **Low** | **Low** | **Low** | **Low** | **Low** | **Low** |
| M. Zhong, et al. (2019) | **Low** | **Low** | **Low** | **Low** | **Low** | **Low** | **Low** | **High** |
| N. Asgin, et al. (2019) | **Low** | **Low** | **Low** | **Low** | **Low** | **Low** | **Low** | **Low** |
| N. N. Rakhi, et al. (2019) | **Low** | **Low** | **Low** | **High** | **Low** | **Low** | **Low** | **Low** |
| N. Tafreshi, et al. (2019) | **Low** | **Low** | **Low** | **Low** | **Low** | **Low** | **Low** | **Low** |
| N. Trapaidze, et al. (2019) | **High** | **Low** | **Low** | **High** | **Low** | **Low** | **High** | **Low** |
| Ö. Koca (2019) | **Low** | **Low** | **Low** | **High** | **Low** | **Low** | **Low** | **High** |
| Q. Cuong Hoang, et al. (2019) | **Low** | **Low** | **Low** | **Low** | **Low** | **Low** | **Low** | **High** |
| R. Furau, et al. (2019) | **Low** | **Low** | **Low** | **High** | **Low** | **Low** | **Low** | **Low** |
| R. Ranjbar, et al. (2019) | **Low** | **Low** | **Low** | **Low** | **Low** | **Low** | **Low** | **Low** |
| R. Ranjbar, et al. (2019) | **Low** | **Low** | **Low** | **Low** | **Low** | **Low** | **Low** | **Low** |
| R. V. Caldart, et al. (2019) | **Low** | **Low** | **High** | **High** | **Low** | **Low** | **Low** | **Low** |
| S. A. Girija, et al. (2019) | **Low** | **Low** | **High** | **Low** | **Low** | **Low** | **Low** | **High** |
| S. Chusri, et al. (2019) | **Low** | **Low** | **Low** | **High** | **Low** | **Low** | **Low** | **Low** |
| S. Girija As, et al. (2019) | **Low** | **Low** | **Low** | **Low** | **Low** | **Low** | **Low** | **High** |
| S. H. Radhi, et al. (2019) | **Low** | **Low** | **Low** | **Low** | **Low** | **Low** | **Low** | **Low** |
| S. Kumar, et al. (2019) | **Low** | **Low** | **Low** | **Low** | **Low** | **High** | **Low** | **Low** |
| S. S. Kara, et al. (2019) | **Low** | **Low** | **Some** | **Some** | **Some** | **Some** | **Some** | **Some** |
| S. T. M. Tolba, et al. (2019) | **Low** | **Low** | **Low** | **High** | **Low** | **High** | **Low** | **Low** |
| S. Tehrani, et al. (2019) | **Low** | **Low** | **Low** | **Low** | **Low** | **Low** | **Low** | **Low** |
| S. Valadan Tahbaz, et al. (2019) | **Low** | **Low** | **Low** | **Low** | **Low** | **Low** | **Low** | **Low** |
| S. Yang, et al. (2019) | **Low** | **Low** | **Low** | **Low** | **Low** | **Low** | **Low** | **Low** |
| T. K. Atik, et al. (2019) | **Low** | **Low** | **Some** | **Low** | **Some** | **Some** | **Low** | **Some** |
| T. Mao, et al. (2019) | **High** | **High** | **Low** | **High** | **Low** | **Low** | **Low** | **Low** |
| T. Y. Li, et al. (2019) | **Low** | **Low** | **Low** | **High** | **Low** | **Low** | **Some** | **High** |
| U. S. S. Coskun, et al. (2019) | **Low** | **Low** | **Low** | **High** | **Low** | **Low** | **Low** | **Low** |
| U. S. Say Coskun, et al. (2019) | **Low** | **Low** | **Low** | **Low** | **High** | **Low** | **Low** | **Low** |
| V. M. Musyoki, et al. (2019) | **Low** | **Low** | **Low** | **High** | **Low** | **Low** | **Low** | **Low** |
| X. Niu, et al. (2019) | **Low** | **Low** | **High** | **Low** | **Low** | **Low** | **Low** | **Low** |
| Y. Huang, et al. (2019) | **Low** | **Low** | **Low** | **High** | **Low** | **Low** | **Low** | **Low** |
| Y. J. Choe, et al. (2019) | **Some** | **Some** | **Some** | **Some** | **Some** | **Some** | **Some** | **Some** |
| Z. Meshkat, et al. (2019) | **Low** | **Low** | **Low** | **High** | **Low** | **Low** | **Low** | **Low** |
| Z. Tayebi, et al. (2019) | **Low** | **Low** | **Low** | **Low** | **Low** | **Low** | **Low** | **Low** |
| A. A. A. Bediako-Bowan, et al. (2020) | **Low** | **Low** | **Low** | **Low** | **Low** | **Low** | **Low** | **Low** |
| A. A. Khodier, et al. (2020) | **Low** | **Low** | **Low** | **Low** | **Low** | **Low** | **Low** | **Low** |
| A. Ayibieke, et al. (2020) | **Low** | **Low** | **Low** | **Low** | **Low** | **Low** | **Low** | **Low** |
| A. Iregui, et al. (2020) | **Low** | **Low** | **Low** | **Low** | **Low** | **Low** | **Low** | **Low** |
| A. Miller, et al. (2020) | **Low** | **Low** | **Low** | **Low** | **Low** | **Low** | **Low** | **Low** |
| A. Olowo-okere, et al. (2020) | **Low** | **High** | **Low** | **High** | **Low** | **Low** | **Low** | **Low** |
| A. Pal, et al. (2020) | **Low** | **High** | **Low** | **Low** | **Low** | **Low** | **Low** | **Low** |
| A. S. N. Haitham, et al. (2020) | **Low** | **Low** | **Low** | **High** | **Low** | **Low** | **Low** | **High** |
| A. y, et al. (2020) | **Low** | **Low** | **Low** | **Low** | **Low** | **Low** | **Low** | **Low** |
| B. Basatian-Tashkan, et al. (2020) | **Low** | **Low** | **Low** | **Low** | **Low** | **Low** | **Low** | **Low** |
| B. Bedenic, et al. (2020) | **Low** | **Low** | **Low** | **Low** | **Low** | **Low** | **Low** | **Low** |
| B. Hashemi, et al. (2020) | **Low** | **Low** | **Low** | **Low** | **Low** | **Low** | **Low** | **Low** |
| B. Ingti, et al. (2020) | **Low** | **Low** | **Low** | **Low** | **Low** | **Low** | **Low** | **Low** |
| B. Lukovic, et al. (2020) | **Low** | **Low** | **Low** | **Low** | **Low** | **High** | **Low** | **Low** |
| B. Mirzaei, et al. (2020) | **Low** | **Low** | **Low** | **High** | **Low** | **Low** | **Low** | **Low** |
| B. Sobouti, et al. (2020) | **Low** | **Low** | **Low** | **Low** | **Low** | **Low** | **Low** | **Low** |
| C. Tellapragada, et al. (2020) | **Low** | **High** | **Low** | **High** | **Low** | **Low** | **Low** | **Low** |
| C. Vicentini, et al. (2020) | **Low** | **Low** | **Low** | **High** | **Low** | **Low** | **Low** | **Low** |
| C.-l. Liu, et al. (2020) | **Low** | **Low** | **Low** | **Low** | **Low** | **Low** | **Low** | **Low** |
| D. ic-Pavlovic, et al. (2020) | **Low** | **Low** | **Low** | **Low** | **Low** | **Low** | **Low** | **Low** |
| D. W. Wareham, et al. (2020) | **Low** | **Low** | **Low** | **Low** | **High** | **Low** | **Low** | **Low** |
| E. A. Ribeiro, et al. (2020) | **Low** | **Low** | **Low** | **High** | **Low** | **Low** | **Low** | **Low** |
| E. Caglan, et al. (2020) | **Low** | **High** | **Low** | **Low** | **Low** | **Low** | **Low** | **Low** |
| E. L. Fonseca, et al. (2020) | **Low** | **Low** | **Low** | **Low** | **Low** | **Low** | **Low** | **Low** |
| E. Zendegani, et al. (2020) | **Low** | **Low** | **Low** | **High** | **Low** | **Low** | **Low** | **Low** |
| F. Licata, et al. (2020) | **Low** | **Low** | **Low** | **Low** | **Low** | **Low** | **Low** | **Low** |
| F. Tarafdar, et al. (2020) | **Low** | **Low** | **Low** | **Low** | **Low** | **Low** | **Low** | **Low** |
| G. Bansal, et al. (2020) | **Low** | **Low** | **Low** | **Low** | **Low** | **Low** | **Low** | **Low** |
| G. L. Genteluci, et al. (2020) | **Low** | **Low** | **Low** | **High** | **Low** | **Low** | **Low** | **High** |
| G. Makke, et al. (2020) | **Low** | **Low** | **Low** | **High** | **Low** | **Low** | **Low** | **Low** |
| G. Metan, et al. (2020) | **Low** | **Low** | **Low** | **Low** | **Low** | **Low** | **Low** | **Low** |
| G. Mir, et al. (2020) | **Low** | **High** | **Low** | **High** | **Low** | **Low** | **Low** | **Low** |
| H. Caskurlu, et al. (2020) | **Low** | **Low** | **Low** | **High** | **Low** | **Low** | **Low** | **High** |
| H. El Hafa, et al. (2020) | **High** | **Low** | **Low** | **Low** | **Low** | **Low** | **Low** | **Low** |
| H. Q. Raheem, et al. (2020) | **Low** | **High** | **Low** | **High** | **Low** | **Low** | **Low** | **Low** |
| H. R. Tawfeeq, et al. (2020) | **Low** | **Low** | **Low** | **High** | **Low** | **Low** | **Low** | **Low** |
| H. S. Sader, et al. (2020) | **Low** | **Low** | **Low** | **High** | **Low** | **Low** | **Low** | **Low** |
| H. Yi, et al. (2020) | **Low** | **Low** | **Low** | **Low** | **Low** | **Low** | **Low** | **Low** |
| H. Zhang, et al. (2020) | **Low** | **Low** | **Low** | **Low** | **Low** | **Low** | **Low** | **Low** |
| I. Morrissey, et al. (2020) | **Low** | **Low** | **Low** | **Low** | **Low** | **Low** | **Low** | **Low** |
| J. Houngsaitong, et al. (2020) | **Low** | **Low** | **Low** | **Low** | **Low** | **High** | **Low** | **Low** |
| J. Li, et al. (2020) | **Low** | **Low** | **Low** | **High** | **Low** | **Low** | **Low** | **High** |
| J. Loraine, et al. (2020) | **Low** | **Low** | **Low** | **Low** | **Low** | **Low** | **Low** | **Low** |
| J. M. Velasco, et al. (2020) | **Low** | **High** | **Low** | **High** | **Low** | **Low** | **Low** | **Low** |
| J. Wang, et al. (2020) | **Low** | **Low** | **Low** | **High** | **Low** | **Low** | **Low** | **Low** |
| J.-b. Chang, et al. (2020) | **Low** | **Low** | **Low** | **High** | **Low** | **Low** | **Low** | **High** |
| K. C. Ashuthosh, et al. (2020) | **Low** | **Low** | **Low** | **Low** | **Low** | **Low** | **High** | **Low** |
| K. L. Li, et al. (2020) | **Low** | **Low** | **Low** | **High** | **Low** | **Low** | **Some** | **High** |
| K. Murata, et al. (2020) | **Low** | **Low** | **Low** | **High** | **Low** | **Low** | **Low** | **Low** |
| K. Nafplioti, et al. (2020) | **Low** | **Low** | **Low** | **High** | **Low** | **Low** | **Low** | **Low** |
| K. Paramita, et al. (2020) | **Low** | **Low** | **Low** | **High** | **Low** | **Low** | **Low** | **Low** |
| L. L. Al-Hassan, et al. (2020) | **Low** | **Low** | **Low** | **Low** | **Low** | **Low** | **Low** | **Low** |
| L. Xu, et al. (2020) | **Low** | **Low** | **Low** | **Low** | **Low** | **Low** | **Low** | **Low** |
| Lopez-Hern, et al. (2020) | **High** | **Low** | **Low** | **High** | **Low** | **Low** | **Low** | **Low** |
| M. A. Hashmi, et al. (2020) | **Low** | **High** | **High** | **High** | **Low** | **Low** | **High** | **Low** |
| M. Alavi-Moghaddam, et al. (2020) | **Low** | **Low** | **Low** | **Low** | **High** | **Low** | **Low** | **Low** |
| M. M. Hamza, et al. (2020) | **Low** | **Low** | **Low** | **High** | **Low** | **High** | **Low** | **Low** |
| M. Moosavian, et al. (2020) | **Low** | **Low** | **Low** | **Low** | **Low** | **Low** | **Low** | **Low** |
| M. Osman, et al. (2020) | **Low** | **Low** | **Low** | **High** | **Low** | **Low** | **Low** | **Low** |
| M. Shabban, et al. (2020) | **Low** | **Low** | **Low** | **Low** | **Low** | **Low** | **Low** | **Low** |
| M. Shahid, et al. (2020) | **Low** | **Low** | **Low** | **High** | **Low** | **Low** | **High** | **High** |
| M. T. H. Talpur, et al. (2020) | **Low** | **High** | **Low** | **High** | **Low** | **Low** | **Low** | **Low** |
| N. Karah, et al. (2020) | **Low** | **Low** | **Low** | **Low** | **Low** | **Low** | **Low** | **Low** |
| Norafika, et al. (2020) | **Low** | **Low** | **Low** | **Low** | **Low** | **Low** | **Low** | **Low** |
| P.-Y. Liu, et al. (2020) | **Low** | **Low** | **Low** | **Low** | **Low** | **Low** | **Low** | **Low** |
| Q. Wang, et al. (2020) | **Low** | **Low** | **Low** | **Low** | **Low** | **Low** | **Low** | **Low** |
| Q. Xu, et al. (2020) | **Low** | **High** | **Low** | **High** | **Low** | **Low** | **Low** | **Low** |
| Q. Yang, et al. (2020) | **Low** | **High** | **Low** | **High** | **Low** | **Low** | **Low** | **Low** |
| R. Bawazeer, et al. (2020) | **Low** | **Low** | **Low** | **Low** | **Low** | **Low** | **High** | **High** |
| R. M. Abd El-Baky, et al. (2020) | **Low** | **Low** | **Low** | **Low** | **Low** | **Low** | **Low** | **High** |
| R. Ranjbar, et al. (2020) | **Low** | **High** | **Low** | **Low** | **Low** | **Low** | **Low** | **Low** |
| S. A. Khrulnova, et al. (2020) | **Low** | **Low** | **Low** | **Low** | **Low** | **Low** | **Low** | **Low** |
| S. A. L. Al Meani, et al. (2020) | **Low** | **Low** | **Low** | **Low** | **Low** | **Low** | **Low** | **Low** |
| S. B. de Freitas, et al. (2020) | **Low** | **Low** | **Low** | **Low** | **Low** | **Low** | **Low** | **Low** |
| S. K. Yadav, et al. (2020) | **Low** | **Low** | **Low** | **High** | **Low** | **Low** | **Low** | **Low** |
| S. Lakoh, et al. (2020) | **Low** | **Low** | **Low** | **High** | **Low** | **Low** | **High** | **Low** |
| S. M. Kadom, et al. (2020) | **High** | **High** | **Low** | **Low** | **Low** | **Low** | **Low** | **High** |
| S. M. Kareem (2020) | **Low** | **Low** | **Low** | **Low** | **High** | **Low** | **Low** | **Low** |
| S. M. McLeod, et al. (2020) | **Low** | **Low** | **Low** | **Low** | **High** | **Low** | **Low** | **Low** |
| S. M. Mortazavi, et al. (2020) | **Low** | **Low** | **Low** | **High** | **Low** | **Low** | **Low** | **Low** |
| S. Mushtaq, et al. (2020) | **Low** | **Low** | **Low** | **High** | **Low** | **Low** | **Low** | **Low** |
| S. N. Gaber, et al. (2020) | **Low** | **Low** | **Low** | **Low** | **Low** | **Low** | **Low** | **Low** |
| S. S. Mabrouk, et al. (2020) | **Low** | **Low** | **Low** | **Low** | **Low** | **Low** | **Low** | **Low** |
| S. Vijayakumar, et al. (2020) | **Low** | **Low** | **Low** | **Low** | **Low** | **Low** | **Low** | **Low** |
| S.-C. Kuo, et al. (2020) | **Low** | **Low** | **Low** | **Low** | **Low** | **Low** | **Low** | **Low** |
| T. B. Talizin, et al. (2020) | **Low** | **High** | **Low** | **Low** | **Low** | **Low** | **Low** | **Low** |
| T. Paiboonvong, et al. (2020) | **Low** | **Low** | **Low** | **High** | **Low** | **High** | **Low** | **Low** |
| V. Rodjun, et al. (2020) | **Low** | **Low** | **Low** | **High** | **Low** | **Low** | **Low** | **Low** |
| V. Thao Nguyen, et al. (2020) | **Low** | **Low** | **Low** | **High** | **Low** | **Low** | **Low** | **Low** |
| V. Whitley, et al. (2020) | **Low** | **Low** | **Low** | **High** | **Low** | **Low** | **Low** | **Low** |
| W. El-Kazzaz, et al. (2020) | **Low** | **Low** | **High** | **Low** | **Low** | **Low** | **High** | **High** |
| Y. Chen, et al. (2020) | **Low** | **Low** | **Low** | **High** | **Low** | **Low** | **Low** | **Low** |
| Y. Yang, et al. (2020) | **Low** | **Low** | **Low** | **Low** | **Low** | **Low** | **Low** | **Low** |
| Y. Zhang, et al. (2020) | **Low** | **Low** | **Low** | **High** | **Low** | **Low** | **Low** | **Low** |
| Y.-C. Hsieh, et al. (2020) | **Low** | **Low** | **Low** | **High** | **Low** | **Low** | **Low** | **Low** |
| Z. Babaie, et al. (2020) | **High** | **High** | **Low** | **Low** | **Low** | **Low** | **Low** | **Low** |
| Z. G. Luo, et al. (2020) | **Low** | **Low** | **Low** | **Low** | **Low** | **Low** | **Low** | **Low** |
| A. A. Al-Sultan (2021) | **Low** | **High** | **Low** | **High** | **Low** | **Low** | **Low** | **Low** |
| A. Camacho-Ortiz, et al. (2021) | **Low** | **High** | **Low** | **High** | **Low** | **Low** | **Low** | **Low** |
| A. Chaurasia, et al. (2021) | **Low** | **Low** | **Low** | **High** | **Low** | **Low** | **Low** | **Low** |
| A. H. Mohamed, et al. (2021) | **Low** | **Low** | **Low** | **High** | **Low** | **Low** | **Low** | **Low** |
| A. Iregui, et al. (2021) | **Low** | **Low** | **Low** | **Low** | **Low** | **Low** | **Low** | **Low** |
| A. Japoni-Nejad, et al. (2021) | **Low** | **Low** | **Low** | **Low** | **Low** | **Low** | **Low** | **Low** |
| A. Lavrinenko, et al. (2021) | **Low** | **Low** | **Low** | **High** | **Low** | **Low** | **Low** | **Low** |
| A. M. Gezmu, et al. (2021) | **Low** | **Low** | **Low** | **Low** | **Low** | **Low** | **Low** | **Low** |
| A. Mahmoud, et al. (2021) | **Low** | **Low** | **Low** | **High** | **Low** | **Low** | **Low** | **Low** |
| A. Massik, et al. (2021) | **Low** | **High** | **Low** | **High** | **Low** | **Low** | **Low** | **Low** |
| A. Mohamed, et al. (2021) | **Low** | **Low** | **Low** | **High** | **Low** | **High** | **Low** | **Low** |
| A. Petca, et al. (2021) | **Low** | **Low** | **Low** | **Low** | **Low** | **Low** | **Low** | **Low** |
| A. Prasai, et al. (2021) | **Low** | **Low** | **Low** | **High** | **Low** | **Low** | **Low** | **Low** |
| A. S. Jaloot, et al. (2021) | **High** | **Low** | **Low** | **High** | **Low** | **Low** | **Low** | **Low** |
| A. Sangale, et al. (2021) | **Low** | **High** | **Low** | **Low** | **High** | **Low** | **Low** | **Low** |
| A. Shahid, et al. (2021) | **Low** | **High** | **Low** | **High** | **Low** | **Low** | **Low** | **Low** |
| A. Szczypta, et al. (2021) | **Low** | **High** | **Low** | **High** | **Low** | **Low** | **Low** | **Low** |
| A. Thatrimontrichai, et al. (2021) | **Low** | **High** | **Low** | **High** | **Low** | **Low** | **Low** | **Low** |
| A. U. Guclu, et al. (2021) | **Low** | **Low** | **Low** | **Low** | **Low** | **Low** | **Low** | **Low** |
| A. Vahhabi, et al. (2021) | **Low** | **High** | **Low** | **Low** | **Low** | **Low** | **Low** | **Low** |
| A. Y. Adjei, et al. (2021) | **Low** | **Low** | **Low** | **High** | **Low** | **Low** | **Low** | **Low** |
| A. Z. M. Maebed, et al. (2021) | **Low** | **Low** | **Low** | **Low** | **Low** | **Low** | **Low** | **Low** |
| B. Baljin, et al. (2021) | **Low** | **Low** | **Low** | **Low** | **Low** | **Low** | **Low** | **Low** |
| B. Cherukuri (2021) | **High** | **High** | **High** | **High** | **Low** | **Low** | **High** | **Low** |
| B. G. Cabral, et al. (2021) | **Low** | **High** | **Low** | **High** | **Low** | **Low** | **Low** | **Low** |
| B. Santella, et al. (2021) | **Low** | **High** | **Low** | **High** | **Low** | **Low** | **Low** | **Low** |
| C. Konca, et al. (2021) | **Low** | **High** | **Low** | **Low** | **Low** | **High** | **Low** | **Low** |
| C. Lasarte-Monterrubio, et al. (2021) | **Low** | **Low** | **Low** | **Low** | **Low** | **Low** | **Low** | **Low** |
| C. Ozkul, et al. (2021) | **Low** | **Low** | **Low** | **Low** | **Low** | **Low** | **Low** | **Low** |
| C. Rodriguez-Lucas, et al. (2021) | **Low** | **Low** | **Low** | **Low** | **Low** | **Low** | **Low** | **Low** |
| D. Alrahmany, et al. (2021) | **Low** | **Low** | **Low** | **High** | **Low** | **Low** | **Low** | **Low** |
| D. Carcione, et al. (2021) | **Low** | **Low** | **Low** | **High** | **Low** | **Low** | **Low** | **Low** |
| D. Kim, et al. (2021) | **Low** | **Low** | **Low** | **High** | **Low** | **Low** | **Low** | **Low** |
| D. Said, et al. (2021) | **Low** | **Low** | **Low** | **High** | **Low** | **Low** | **Low** | **Low** |
| E. Abbasi, et al. (2021) | **Low** | **Low** | **Low** | **Low** | **Low** | **Low** | **Low** | **Low** |
| E. Cercenado, et al. (2021) | **Low** | **Low** | **Low** | **High** | **Low** | **Low** | **Low** | **Low** |
| F. Karami, et al. (2021) | **Low** | **Low** | **Low** | **Low** | **Low** | **Low** | **Low** | **Low** |
| F. M. Al-Asady, et al. (2021) | **Low** | **High** | **Low** | **High** | **Low** | **Low** | **Low** | **Low** |
| F. Naeimi Mazraeh, et al. (2021) | **Low** | **High** | **Low** | **Low** | **Low** | **Low** | **Low** | **Low** |
| F. Sana, et al. (2021) | **High** | **High** | **Low** | **High** | **Low** | **Low** | **Low** | **Low** |
| G. Al-Hashem, et al. (2021) | **Low** | **Low** | **Low** | **Low** | **Low** | **Low** | **Low** | **Low** |
| G. G. Gaspar, et al. (2021) | **Low** | **Low** | **Low** | **High** | **Low** | **Low** | **Low** | **Low** |
| G. M. Rossolini, et al. (2021) | **Low** | **Low** | **Low** | **High** | **Low** | **Low** | **Low** | **Low** |
| H. Duran, et al. (2021) | **Low** | **High** | **Low** | **High** | **Low** | **Low** | **Low** | **Low** |
| H. Ejaz, et al. (2021) | **Low** | **Low** | **Low** | **Low** | **Low** | **Low** | **Low** | **Low** |
| H. Guan, et al. (2021) | **Low** | **Low** | **Low** | **High** | **Low** | **High** | **Low** | **Low** |
| H. Masoumi-Asl, et al. (2021) | **Low** | **Low** | **Low** | **Low** | **Low** | **Low** | **Low** | **Low** |
| H. Mekonnen, et al. (2021) | **Low** | **Low** | **Low** | **High** | **Low** | **Low** | **Low** | **Low** |
| H. S. Sader, et al. (2021) | **Low** | **Low** | **Low** | **High** | **High** | **Low** | **Low** | **Low** |
| I. R. Ali, et al. (2021) | **Low** | **High** | **Low** | **High** | **Low** | **Low** | **Low** | **Low** |
| J. Chen, et al. (2021) | **Low** | **Low** | **Low** | **Low** | **Low** | **Low** | **Low** | **Low** |
| J. M. Khaled, et al. (2021) | **Low** | **High** | **Low** | **Low** | **Low** | **Low** | **Low** | **Low** |
| J. Qu, et al. (2021) | **Low** | **Low** | **Low** | **Low** | **Low** | **Low** | **Low** | **Low** |
| J. Yeongdon, et al. (2021) | **Low** | **High** | **Low** | **Low** | **Low** | **Low** | **Low** | **Low** |
| K. B. Said, et al. (2021) | **Low** | **Low** | **Low** | **High** | **Low** | **Low** | **Low** | **Low** |
| K. Subramaniam, et al. (2021) | **Low** | **High** | **Low** | **High** | **High** | **Low** | **Low** | **Low** |
| K. Yu, et al. (2021) | **Low** | **High** | **Low** | **High** | **Low** | **Low** | **Low** | **Low** |
| L. Al-Hassan, et al. (2021) | **Low** | **Low** | **Low** | **High** | **Low** | **Low** | **Low** | **Low** |
| L. P. Nguyen, et al. (2021) | **Low** | **Low** | **Low** | **High** | **Low** | **Low** | **Low** | **Low** |
| L. Rodrigues Perez, et al. (2021) | **Low** | **Low** | **Low** | **High** | **Low** | **Low** | **Low** | **Low** |
| L. Wang, et al. (2021) | **Low** | **High** | **Low** | **Low** | **Low** | **Low** | **Low** | **Low** |
| M. Arbune, et al. (2021) | **Low** | **High** | **Low** | **Low** | **High** | **Low** | **Low** | **Low** |
| M. Azad, et al. (2021) | **Low** | **Low** | **Low** | **High** | **Low** | **Low** | **Low** | **Low** |
| M. B. Sannathimmappa, et al. (2021) | **Low** | **Low** | **Low** | **High** | **Low** | **Low** | **Low** | **Low** |
| M. C. Jara, et al. (2021) | **Low** | **Low** | **Low** | **High** | **High** | **Low** | **Low** | **Low** |
| M. C. Silveira, et al. (2021) | **Low** | **High** | **Low** | **High** | **Low** | **Low** | **Low** | **Low** |
| M. F. El-Badawy, et al. (2021) | **Low** | **High** | **Low** | **High** | **Low** | **Low** | **Low** | **Low** |
| M. G. Donadu, et al. (2021) | **Low** | **Low** | **Low** | **High** | **Low** | **Low** | **Low** | **Low** |
| M. G. Donadu, et al. (2021) | **Low** | **High** | **Low** | **High** | **Low** | **Low** | **Low** | **Low** |
| M. Guvenir, et al. (2021) | **Low** | **High** | **Low** | **Low** | **Low** | **Low** | **Low** | **Low** |
| M. H. Namaei, et al. (2021) | **Low** | **Low** | **Low** | **Low** | **Low** | **Low** | **Low** | **Low** |
| M. Hazhirkamal, et al. (2021) | **Low** | **Low** | **Low** | **Low** | **Low** | **Low** | **Low** | **Low** |
| M. Inggraini, et al. (2021) | **Low** | **Low** | **Low** | **High** | **Low** | **Low** | **Low** | **Low** |
| M. Jiang, et al. (2021) | **Low** | **Low** | **Low** | **Low** | **Low** | **Low** | **Low** | **Low** |
| M. K. Jayanthi, et al. (2021) | **Low** | **Low** | **Low** | **High** | **Low** | **High** | **Low** | **Low** |
| M. Kumari, et al. (2021) | **Low** | **Low** | **Low** | **High** | **Low** | **High** | **Low** | **Low** |
| M. M. E. Meybodi, et al. (2021) | **Low** | **Low** | **Low** | **Low** | **High** | **Low** | **Low** | **Low** |
| M. M. Zafer, et al. (2021) | **Low** | **High** | **Low** | **Low** | **High** | **Low** | **Low** | **Low** |
| M. Nikibakhsh, et al. (2021) | **Low** | **High** | **Low** | **High** | **Low** | **Low** | **Low** | **Low** |
| M. Saadati, et al. (2021) | **Low** | **Low** | **Low** | **Low** | **Low** | **Low** | **Low** | **Low** |
| M. Sadr, et al. (2021) | **Low** | **Low** | **Low** | **Low** | **Low** | **Low** | **Low** | **Low** |
| N. A. Chitrabanu, et al. (2021) | **Low** | **High** | **Low** | **Low** | **Low** | **Low** | **Low** | **Low** |
| N. Bankan, et al. (2021) | **High** | **High** | **Low** | **High** | **Low** | **Low** | **High** | **Low** |
| N. Duan, et al. (2021) | **Low** | **Low** | **Low** | **High** | **Low** | **Low** | **Low** | **Low** |
| N. El-Kattan, et al. (2021) | **Low** | **Low** | **Low** | **High** | **Low** | **Low** | **Low** | **Low** |
| N. G. Banoub, et al. (2021) | **Low** | **Low** | **Low** | **Low** | **Low** | **Low** | **Low** | **Low** |
| N. J. Ahmed, et al. (2021) | **High** | **High** | **High** | **High** | **High** | **Low** | **High** | **High** |
| N. Javaid, et al. (2021) | **Low** | **High** | **Low** | **High** | **Low** | **Low** | **Low** | **Low** |
| N. Kansak, et al. (2021) | **Low** | **Low** | **Low** | **Low** | **Low** | **Low** | **Low** | **Low** |
| N. Khursheed, et al. (2021) | **Low** | **Low** | **Low** | **Low** | **Low** | **Low** | **Low** | **Low** |
| N. Moradi, et al. (2021) | **Low** | **Low** | **Low** | **Low** | **Low** | **Low** | **Low** | **Low** |
| N. Palanisamy, et al. (2021) | **Low** | **Low** | **Low** | **High** | **Low** | **Low** | **Low** | **Low** |
| N. Sharma, et al. (2021) | **Low** | **High** | **Low** | **High** | **Low** | **Low** | **Low** | **Low** |
| N. Y. Nojookambari, et al. (2021) | **Low** | **Low** | **Low** | **High** | **Low** | **Low** | **Low** | **Low** |
| N. Yousefi Nojookambari, et al. (2021) | **Low** | **High** | **Low** | **Low** | **Low** | **Low** | **Low** | **Low** |
| N. Zahra, et al. (2021) | **Low** | **High** | **Low** | **High** | **Low** | **Low** | **Low** | **Low** |
| N.-D. Nogbou, et al. (2021) | **Low** | **Low** | **Low** | **High** | **Low** | **Low** | **Low** | **Low** |
| O. Akgul, et al. (2021) | **Low** | **Low** | **Low** | **High** | **Low** | **Low** | **Low** | **Low** |
| O. Azizi, et al. (2021) | **Low** | **Low** | **Low** | **Low** | **Low** | **Low** | **Low** | **Low** |
| O. F. Nwabor, et al. (2021) | **Low** | **Low** | **Low** | **Low** | **Low** | **Low** | **Low** | **Low** |
| P. A. Moise, et al. (2021) | **Low** | **Low** | **Low** | **Low** | **High** | **Low** | **Low** | **Low** |
| P. Chhatwal, et al. (2021) | **Low** | **Low** | **Low** | **High** | **High** | **Low** | **Low** | **Low** |
| P. Di Carlo, et al. (2021) | **Low** | **Low** | **Low** | **High** | **Low** | **Low** | **Low** | **Low** |
| P. Hannus, et al. (2021) | **Low** | **Low** | **Low** | **High** | **Low** | **Low** | **Low** | **Low** |
| P. Khuntayaporn, et al. (2021) | **Low** | **High** | **Low** | **Low** | **Low** | **High** | **Low** | **Low** |
| P. Khuntayaporn, et al. (2021) | **Low** | **Low** | **Low** | **High** | **Low** | **Low** | **Low** | **Low** |
| P. Manohar, et al. (2021) | **Low** | **High** | **Low** | **High** | **Low** | **Low** | **Low** | **Low** |
| P. Simeon, et al. (2021) | **Low** | **High** | **Low** | **High** | **Low** | **Low** | **Low** | **Low** |
| R. Abozahra, et al. (2021) | **Low** | **Low** | **Low** | **High** | **Low** | **Low** | **Low** | **Low** |
| R. El-Sokkary, et al. (2021) | **Low** | **Low** | **Low** | **Low** | **Low** | **Low** | **Low** | **Low** |
| R. L. Boone, et al. (2021) | **High** | **High** | **High** | **High** | **Low** | **Low** | **High** | **Low** |
| R. Wasfi, et al. (2021) | **Low** | **High** | **Low** | **High** | **Low** | **Low** | **Low** | **Low** |
| S. Bahrami, et al. (2021) | **Low** | **Low** | **Low** | **Low** | **Low** | **Low** | **Low** | **Low** |
| S. J. Moyo, et al. (2021) | **Low** | **Low** | **Low** | **High** | **Low** | **Low** | **Low** | **Low** |
| S. Mohsin, et al. (2021) | **Low** | **Low** | **Low** | **High** | **Low** | **Low** | **Low** | **Low** |
| S. Mostafavi, et al. (2021) | **Low** | **Low** | **Low** | **Low** | **Low** | **Low** | **Low** | **Low** |
| S. S. Khoramrooz, et al. (2021) | **Low** | **Low** | **Low** | **High** | **High** | **Low** | **Low** | **Low** |
| S. V. Bharathi, et al. (2021) | **Low** | **High** | **Low** | **High** | **High** | **Low** | **Low** | **Low** |
| S. Vijay, et al. (2021) | **Low** | **High** | **Low** | **Low** | **Low** | **Low** | **Low** | **Low** |
| S. Zhong, et al. (2021) | **Low** | **Low** | **Low** | **High** | **Low** | **Low** | **Low** | **Low** |
| Sepahv, et al. (2021) | **Low** | **High** | **Low** | **Low** | **Low** | **Low** | **Low** | **Low** |
| T. Chen, et al. (2021) | **Low** | **Low** | **Low** | **High** | **Low** | **Low** | **Low** | **Low** |
| T. Naas, et al. (2021) | **Low** | **Low** | **Low** | **Low** | **Low** | **Low** | **Low** | **Low** |
| T. Yungyuen, et al. (2021) | **Low** | **High** | **Low** | **Low** | **Low** | **Low** | **Low** | **Low** |
| T. Zhang, et al. (2021) | **Low** | **High** | **Low** | **Low** | **Low** | **Low** | **Low** | **Low** |
| U. Ghimire, et al. (2021) | **Low** | **Low** | **Low** | **High** | **Low** | **Low** | **Low** | **Low** |
| V. Kondratiuk, et al. (2021) | **Low** | **Low** | **Low** | **High** | **Low** | **Low** | **Low** | **Low** |
| V. M. d. Carvalho Hessel Dias, et al. (2021) | **Low** | **Low** | **Low** | **Low** | **Low** | **Low** | **Low** | **Low** |
| V. T. T. Selvi, et al. (2021) | **Low** | **High** | **Low** | **High** | **Low** | **Low** | **Low** | **Low** |
| V. Tien Viet Dung, et al. (2021) | **Low** | **High** | **Low** | **Low** | **Low** | **Low** | **Low** | **Low** |
| V.-T. Hoang, et al. (2021) | **Low** | **Low** | **Low** | **High** | **Low** | **Low** | **Low** | **Low** |
| W. Flores-Paredes, et al. (2021) | **High** | **Low** | **Low** | **Low** | **Low** | **Low** | **Low** | **Low** |
| W. P. ar, et al. (2021) | **Low** | **Low** | **Low** | **High** | **Low** | **Low** | **Low** | **Low** |
| X. Bian, et al. (2021) | **Low** | **Low** | **Low** | **High** | **Low** | **Low** | **Low** | **Low** |
| X. Jin, et al. (2021) | **Low** | **Low** | **Low** | **High** | **Low** | **Low** | **Low** | **Low** |
| X. Meng, et al. (2021) | **Low** | **High** | **Low** | **High** | **Low** | **Low** | **Low** | **Low** |
| X. Shi, et al. (2021) | **Low** | **High** | **Low** | **High** | **Low** | **Low** | **Low** | **Low** |
| X. Xu, et al. (2021) | **Low** | **Low** | **Low** | **Low** | **Low** | **Low** | **Low** | **Low** |
| Y. Qiu, et al. (2021) | **Low** | **Low** | **Low** | **Low** | **Low** | **Low** | **Low** | **Low** |
| Y. Takebayashi, et al. (2021) | **Low** | **High** | **Low** | **High** | **Low** | **Low** | **Low** | **Low** |
| Y. Zhang, et al. (2021) | **Low** | **High** | **Low** | **Low** | **Low** | **High** | **Low** | **Low** |
| Z. Elnasser, et al. (2021) | **Low** | **Low** | **Low** | **High** | **Low** | **Low** | **Low** | **Low** |
| Z. Li, et al. (2021) | **Low** | **Low** | **Low** | **High** | **Low** | **Low** | **Low** | **Low** |
| Z. Zhang, et al. (2021) | **Low** | **Low** | **Low** | **High** | **Low** | **Low** | **Low** | **Low** |
| Z.-J. Li, et al. (2021) | **Low** | **Low** | **Low** | **Low** | **Low** | **Low** | **Low** | **Low** |
| A. A. K. Shali, et al. (2022) | **Low** | **Low** | **Low** | **High** | **Low** | **Low** | **Low** | **Low** |
| A. A. Shah, et al. (2022) | **Low** | **High** | **Low** | **High** | **Low** | **Low** | **Low** | **Low** |
| A. C. Yardimci, et al. (2022) | **Low** | **High** | **Low** | **High** | **Low** | **Low** | **Low** | **Low** |
| A. Fatima, et al. (2022) | **Low** | **High** | **Low** | **High** | **Low** | **Low** | **Low** | **Low** |
| A. H. Mohamed, et al. (2022) | **Low** | **Low** | **Low** | **High** | **Low** | **Low** | **Low** | **Low** |
| A. Kabrah (2022) | **Low** | **Low** | **Low** | **High** | **High** | **Low** | **Low** | **Low** |
| A. Maleki, et al. (2022) | **Low** | **High** | **Low** | **Low** | **Low** | **Low** | **Low** | **Low** |
| A. Rahman, et al. (2022) | **Low** | **High** | **Low** | **High** | **Low** | **Low** | **Low** | **Low** |
| A. Rizvi, et al. (2022) | **Low** | **Low** | **Low** | **Low** | **Low** | **Low** | **Low** | **Low** |
| A. Shami, et al. (2022) | **Low** | **High** | **Low** | **High** | **Low** | **Low** | **High** | **Low** |
| A. Thampithak, et al. (2022) | **Low** | **Low** | **Low** | **High** | **Low** | **Low** | **Low** | **Low** |
| A.-L. Golli, et al. (2022) | **Low** | **Low** | **Low** | **High** | **Low** | **Low** | **Low** | **Low** |
| B. Balazs, et al. (2022) | **Low** | **High** | **Low** | **High** | **Low** | **Low** | **Low** | **Low** |
| B. M. Forde, et al. (2022) | **Low** | **Low** | **Low** | **High** | **Low** | **Low** | **Low** | **Low** |
| C. Kaur, et al. (2022) | **Low** | **Low** | **Low** | **High** | **Low** | **Low** | **Low** | **Low** |
| C. Liu, et al. (2022) | **Low** | **High** | **Low** | **High** | **Low** | **Low** | **Low** | **Low** |
| D. Anggraini, et al. (2022) | **Low** | **Low** | **Low** | **High** | **Low** | **Low** | **Low** | **Low** |
| D. Depka, et al. (2022) | **Low** | **High** | **Low** | **Low** | **High** | **Low** | **Low** | **Low** |
| D. Liu, et al. (2022) | **Low** | **Low** | **Low** | **High** | **Low** | **Low** | **Low** | **Low** |
| F. Hu, et al. (2022) | **Low** | **Low** | **Low** | **High** | **Low** | **Low** | **Low** | **Low** |
| F. Jabeen, et al. (2022) | **Low** | **High** | **Low** | **High** | **Low** | **Low** | **Low** | **Low** |
| F. R. Zaniani, et al. (2022) | **Low** | **High** | **Low** | **High** | **Low** | **Low** | **Low** | **Low** |
| G. Petazzoni, et al. (2022) | **Low** | **High** | **Low** | **High** | **Low** | **Low** | **Low** | **Low** |
| H. A. Kadhom, et al. (2022) | **Low** | **High** | **Low** | **High** | **Low** | **Low** | **Low** | **Low** |
| H. A. Mohamed, et al. (2022) | **Low** | **Low** | **Low** | **High** | **Low** | **Low** | **High** | **Low** |
| H. D. Tran, et al. (2022) | **Low** | **Low** | **Low** | **High** | **Low** | **High** | **High** | **Low** |
| H. G. A. Alshami, et al. (2022) | **Low** | **High** | **Low** | **High** | **Low** | **Low** | **Low** | **Low** |
| H. S. Rusul, et al. (2022) | **Low** | **High** | **Low** | **High** | **Low** | **Low** | **Low** | **Low** |
| H. S. Sader, et al. (2022) | **Low** | **High** | **Low** | **High** | **Low** | **Low** | **Low** | **Low** |
| H. S. Sader, et al. (2022) | **Low** | **High** | **Low** | **Low** | **Low** | **Low** | **Low** | **Low** |
| H. Seifert, et al. (2022) | **Low** | **Low** | **Low** | **Low** | **Low** | **Low** | **Low** | **Low** |
| H.-N. Wu, et al. (2022) | **Low** | **Low** | **Low** | **High** | **Low** | **Low** | **Low** | **Low** |
| I. Erdem, et al. (2022) | **Low** | **Low** | **Low** | **High** | **Low** | **Low** | **Low** | **Low** |
| I. Mumcuoglu, et al. (2022) | **Low** | **High** | **Low** | **Low** | **Low** | **Low** | **Low** | **Low** |
| J. A. Karlowsky, et al. (2022) | **Low** | **Low** | **Low** | **High** | **Low** | **Low** | **Low** | **Low** |
| J. A. Karlowsky, et al. (2022) | **Low** | **Low** | **Low** | **High** | **Low** | **Low** | **Low** | **Low** |
| J. A. Karlowsky, et al. (2022) | **Low** | **Low** | **Low** | **High** | **Low** | **Low** | **Low** | **Low** |
| J. Gao, et al. (2022) | **Low** | **High** | **Low** | **Low** | **Low** | **Low** | **Low** | **Low** |
| J. M. Pogue, et al. (2022) | **Low** | **Low** | **Low** | **High** | **Low** | **Low** | **Low** | **Low** |
| J. Xi, et al. (2022) | **Low** | **Low** | **Low** | **Low** | **Low** | **Low** | **Low** | **Low** |
| J. Zhang, et al. (2022) | **Low** | **High** | **Low** | **High** | **Low** | **Low** | **Low** | **Low** |
| K. I. AlQumaizi, et al. (2022) | **Low** | **High** | **Low** | **High** | **High** | **Low** | **Low** | **Low** |
| L. Lu, et al. (2022) | **Low** | **Low** | **Low** | **High** | **Low** | **Low** | **Low** | **Low** |
| M. A. Ababneh, et al. (2022) | **Low** | **Low** | **Low** | **High** | **Low** | **Low** | **Low** | **Low** |
| M. A. Chilon-Chavez, et al. (2022) | **Low** | **High** | **Low** | **High** | **Low** | **Low** | **Low** | **Low** |
| M. Al-Tamimi, et al. (2022) | **Low** | **High** | **Low** | **High** | **Low** | **Low** | **Low** | **Low** |
| M. F. M. Subagdja, et al. (2022) | **Low** | **Low** | **Low** | **High** | **Low** | **Low** | **Low** | **Low** |
| M. H. Haider, et al. (2022) | **Low** | **High** | **Low** | **High** | **Low** | **Low** | **Low** | **Low** |
| M. H. Legese, et al. (2022) | **Low** | **High** | **Low** | **High** | **Low** | **Low** | **Low** | **Low** |
| M. K. Devian, et al. (2022) | **Low** | **High** | **High** | **High** | **Low** | **Low** | **High** | **High** |
| M. Kafshnouchi, et al. (2022) | **Low** | **Low** | **Low** | **High** | **Low** | **Low** | **Low** | **Low** |
| M. Li, et al. (2022) | **Low** | **Low** | **Low** | **High** | **Low** | **Low** | **Low** | **Low** |
| M. N. Lucas Kurihara, et al. (2022) | **Low** | **Low** | **Low** | **High** | **Low** | **Low** | **Low** | **Low** |
| M. R. Rao, et al. (2022) | **Low** | **High** | **Low** | **Low** | **Low** | **Low** | **Low** | **Low** |
| N. Kashkouri, et al. (2022) | **Low** | **Low** | **Low** | **High** | **Low** | **Low** | **Low** | **Low** |
| N. Kumari, et al. (2022) | **Low** | **Low** | **Low** | **High** | **Low** | **High** | **Low** | **Low** |
| N. Shi, et al. (2022) | **Low** | **High** | **Low** | **High** | **Low** | **Low** | **Low** | **Low** |
| N. U. Tuzemen, et al. (2022) | **Low** | **Low** | **Low** | **High** | **Low** | **Low** | **High** | **Low** |
| O. Acer, et al. (2022) | **Low** | **Low** | **Low** | **High** | **Low** | **Low** | **Low** | **Low** |
| O. Aydemir, et al. (2022) | **Low** | **High** | **Low** | **High** | **Low** | **Low** | **Low** | **Low** |
| O. E. Khokhlova, et al. (2022) | **Low** | **Low** | **Low** | **Low** | **Low** | **Low** | **High** | **Low** |
| O. Ergonul, et al. (2022) | **High** | **High** | **High** | **High** | **Low** | **Low** | **High** | **Low** |
| O. Katoch, et al. (2022) | **Low** | **Low** | **Low** | **High** | **Low** | **Low** | **Low** | **Low** |
| P. Jia, et al. (2022) | **Low** | **High** | **Low** | **High** | **Low** | **Low** | **Low** | **Low** |
| P. Santoso, et al. (2022) | **Low** | **Low** | **Low** | **High** | **Low** | **Low** | **Low** | **Low** |
| R. C. Mashau, et al. (2022) | **Low** | **High** | **Low** | **High** | **Low** | **Low** | **Low** | **Low** |
| R. Farzana, et al. (2022) | **Low** | **Low** | **Low** | **Low** | **High** | **Low** | **Low** | **Low** |
| R. H. Shayea, et al. (2022) | **Low** | **High** | **Low** | **High** | **Low** | **Low** | **Low** | **Low** |
| R. Han, et al. (2022) | **Low** | **High** | **Low** | **High** | **Low** | **Low** | **Low** | **Low** |
| R. Khodashahi, et al. (2022) | **Low** | **Low** | **Low** | **High** | **Low** | **Low** | **Low** | **Low** |
| R. Khoshbakht, et al. (2022) | **Low** | **Low** | **Low** | **High** | **Low** | **Low** | **Low** | **Low** |
| R. Rabayah, et al. (2022) | **Low** | **High** | **Low** | **Low** | **Low** | **Low** | **Low** | **Low** |
| S. E. R. Mohamed, et al. (2022) | **Low** | **Low** | **Low** | **Low** | **Low** | **Low** | **High** | **Low** |
| S. Farajnia, et al. (2022) | **Low** | **High** | **Low** | **Low** | **Low** | **Low** | **Low** | **Low** |
| S. Ghasemi, et al. (2022) | **Low** | **High** | **Low** | **Low** | **Low** | **Low** | **Low** | **Low** |
| S. H. Mostafa, et al. (2022) | **Low** | **Low** | **Low** | **Low** | **Low** | **Low** | **Low** | **Low** |
| S. Hattab, et al. (2022) | **Low** | **Low** | **Low** | **High** | **Low** | **Low** | **Low** | **Low** |
| S. J. Mun, et al. (2022) | **Low** | **Low** | **Low** | **High** | **Low** | **Low** | **Low** | **Low** |
| S. Kang, et al. (2022) | **Low** | **Low** | **Low** | **High** | **Low** | **Low** | **Low** | **Low** |
| S. Lakoh, et al. (2022) | **Low** | **Low** | **Low** | **High** | **Low** | **Low** | **High** | **Low** |
| S. Mahich, et al. (2022) | **Low** | **Low** | **Low** | **High** | **Low** | **Low** | **Low** | **Low** |
| S. Pourajam, et al. (2022) | **Low** | **Low** | **Low** | **High** | **Low** | **Low** | **Low** | **Low** |
| S. Ruekit, et al. (2022) | **Low** | **High** | **Low** | **High** | **Low** | **Low** | **Low** | **Low** |
| S. S. Masoud, et al. (2022) | **Low** | **High** | **Low** | **High** | **Low** | **Low** | **Low** | **Low** |
| S. Selim, et al. (2022) | **High** | **High** | **Low** | **High** | **Low** | **Low** | **Low** | **Low** |
| S. Shabazi, et al. (2022) | **Low** | **Low** | **Low** | **High** | **Low** | **Low** | **Low** | **Low** |
| S. Sharma, et al. (2022) | **Low** | **Low** | **Low** | **High** | **Low** | **Low** | **Low** | **Low** |
| S. Sharma, et al. (2022) | **Low** | **Low** | **Low** | **High** | **Low** | **High** | **Low** | **Low** |
| S.-S. Jean, et al. (2022) | **Low** | **High** | **Low** | **Low** | **Low** | **Low** | **Low** | **Low** |
| T. San, et al. (2022) | **Low** | **High** | **Low** | **High** | **Low** | **Low** | **Low** | **Low** |
| T. Sewunet, et al. (2022) | **Low** | **Low** | **Low** | **High** | **Low** | **Low** | **Low** | **Low** |
| Y. Chen, et al. (2022) | **Low** | **High** | **Low** | **Low** | **Low** | **Low** | **Low** | **Low** |
| Y. Gu, et al. (2022) | **Low** | **High** | **Low** | **High** | **Low** | **Low** | **Low** | **Low** |
| Y. Han, et al. (2022) | **Low** | **High** | **Low** | **Low** | **Low** | **High** | **Low** | **Low** |
| Y. Li, et al. (2022) | **Low** | **High** | **Low** | **High** | **High** | **Low** | **Low** | **Low** |
| Y. Liu, et al. (2022) | **Low** | **High** | **Low** | **High** | **Low** | **Low** | **Low** | **Low** |
| Z. M. Afshar, et al. (2022) | **Low** | **Low** | **Low** | **High** | **Low** | **Low** | **Low** | **Low** |
| Z. Wei, et al. (2022) | **Low** | **Low** | **Low** | **High** | **Low** | **Low** | **Low** | **Low** |
| Z. Zhang, et al. (2022) | **Low** | **High** | **Low** | **High** | **Low** | **Low** | **Low** | **Low** |
| A. AliMohammadi, et al. (2023) | **Low** | **High** | **Low** | **Low** | **Low** | **Low** | **Low** | **Low** |
| A. Balkhair, et al. (2023) | **Low** | **High** | **Low** | **High** | **Low** | **Low** | **Low** | **Low** |
| A. Bitew, et al. (2023) | **Low** | **High** | **Low** | **Low** | **Low** | **Low** | **Low** | **Low** |
| A. I. I. Aedh, et al. (2023) | **High** | **High** | **Low** | **High** | **Low** | **Low** | **High** | **Low** |
| A. Lavrinenko, et al. (2023) | **Low** | **Low** | **Low** | **Low** | **Low** | **Low** | **Low** | **Low** |
| A. Salmanov, et al. (2023) | **Low** | **High** | **Low** | **Low** | **Low** | **Low** | **Low** | **Low** |
| A. Smitran, et al. (2023) | **High** | **High** | **Low** | **High** | **Low** | **Low** | **Low** | **Low** |
| A.-C. Dikoumba, et al. (2023) | **Low** | **Low** | **Low** | **High** | **High** | **Low** | **Low** | **Low** |
| B. Bedenic, et al. (2023) | **Low** | **High** | **Low** | **High** | **Low** | **Low** | **Low** | **Low** |
| B. S. Alotaibi, et al. (2023) | **Low** | **Low** | **Low** | **High** | **Low** | **Low** | **Low** | **Low** |
| C.-H. Chen, et al. (2023) | **Low** | **Low** | **Low** | **High** | **Low** | **Low** | **Low** | **Low** |
| E. E. Odih, et al. (2023) | **High** | **Low** | **Low** | **High** | **Low** | **Low** | **Low** | **Low** |
| K. Dobrovic, et al. (2023) | **Low** | **Low** | **Low** | **High** | **Low** | **Low** | **Low** | **Low** |
| K. Novovic, et al. (2023) | **Low** | **Low** | **Low** | **Low** | **Low** | **Low** | **Low** | **Low** |
| K. Slimene, et al. (2023) | **Low** | **High** | **Low** | **High** | **Low** | **Low** | **Low** | **Low** |
| K. Yamba, et al. (2023) | **Low** | **Low** | **Low** | **Low** | **Low** | **Low** | **Low** | **Low** |
| L. Azimi, et al. (2023) | **Low** | **High** | **Low** | **Low** | **Low** | **High** | **Low** | **Low** |
| M. Bori, et al. (2023) | **Low** | **Low** | **High** | **High** | **Low** | **Low** | **High** | **High** |
| M. Chakraborty, et al. (2023) | **Low** | **High** | **Low** | **High** | **Low** | **Low** | **Low** | **Low** |
| M. D. Alcantar-Curiel, et al. (2023) | **Low** | **High** | **Low** | **Low** | **Low** | **Low** | **Low** | **Low** |
| M. Kar, et al. (2023) | **High** | **High** | **Low** | **High** | **Low** | **High** | **High** | **Low** |
| M. M. Mustafai, et al. (2023) | **Low** | **Low** | **Low** | **High** | **Low** | **Low** | **Low** | **Low** |
| M. Pourabdollah, et al. (2023) | **High** | **Low** | **Low** | **Low** | **Low** | **Low** | **Low** | **Low** |
| M. R. Taysi, et al. (2023) | **Low** | **Low** | **Low** | **High** | **High** | **Low** | **High** | **Low** |
| M. T. Della Rocca, et al. (2023) | **Low** | **Low** | **Low** | **High** | **Low** | **Low** | **Low** | **Low** |
| N. Van An, et al. (2023) | **Low** | **Low** | **Low** | **High** | **High** | **Low** | **Low** | **Low** |
| P. Ioannou, et al. (2023) | **High** | **High** | **Low** | **Low** | **Low** | **High** | **Low** | **Low** |
| P. O. Barth, et al. (2023) | **Low** | **High** | **Low** | **Low** | **Low** | **Low** | **Low** | **Low** |
| Q. Meng, et al. (2023) | **Low** | **Low** | **Low** | **Low** | **Low** | **High** | **Low** | **Low** |
| Q. You, et al. (2023) | **High** | **High** | **Low** | **Low** | **Low** | **Low** | **Low** | **Low** |
| R. I. Aloraifi, et al. (2023) | **High** | **High** | **High** | **High** | **Low** | **Low** | **High** | **Low** |
| R. Khalifa, et al. (2023) | **Low** | **Low** | **Low** | **Low** | **Low** | **Low** | **Low** | **Low** |
| R. M. Humphries, et al. (2023) | **Low** | **High** | **Low** | **High** | **Low** | **Low** | **Low** | **Low** |
| S. H. Jun, et al. (2023) | **Low** | **Low** | **Low** | **Low** | **Low** | **Low** | **Low** | **Low** |
| S. Santajit, et al. (2023) | **High** | **High** | **Low** | **High** | **Low** | **Low** | **Low** | **Low** |
| T. A. Hafiz, et al. (2023) | **Low** | **Low** | **Low** | **High** | **Low** | **Low** | **Low** | **Low** |
| X.-L. Wei, et al. (2023) | **Low** | **Low** | **Low** | **High** | **Low** | **Low** | **Low** | **Low** |
| Y. Liu, et al. (2023) | **Low** | **Low** | **Low** | **Low** | **Low** | **Low** | **Low** | **Low** |
| Y.-L. Lee, et al. (2023) | **Low** | **Low** | **Low** | **High** | **Low** | **Low** | **Low** | **Low** |
| Z. Kareem Raheem, et al. (2023) | **Low** | **Low** | **Low** | **High** | **Low** | **Low** | **High** | **Low** |

Table Caption: Risk of Bias Assessment Categories: High; High risk, Low; low risk, some; some concern

# Supplementary Table 3: Detailed results of meta-analysis and subgroup analyzing

| Category | Subgroup | K_n_N | Proportion_LCI_HCI | I. | P1 | P2 | P3 |
| --- | --- | --- | --- | --- | --- | --- | --- |
| Overall | **NA** | **73 (39075, 95107)** | **0.761 (0.704, 0.809)** | **91.50%** | **p=0.001** | **p=0.001** | **NA** |
| Year group | **1995-2014** | **9 (1985, 3643)** | **0.529 (0.392, 0.662)** | **96.69%** | **p=0.679** | **p=0.001** | **p=0.156** |
|  | **2015-2019** | **54 (13891, 18970)** | **0.716 (0.659, 0.766)** | **97.25%** | **p=0.001** | **p=0.001** |  |
|  | **2020-2023** | **83 (23199, 72494)** | **0.791 (0.708, 0.855)** | **99.65%** | **p=0.001** | **p=0.001** |  |
| Pre-post COVID-19 | **1995-2019** | **63 (15876, 22613)** | **0.689 (0.636, 0.738)** | **97.54%** | **p=0.001** | **p=0.001** | **p=0.204** |
|  | **2020-2023** | **83 (23199, 72494)** | **0.791 (0.708, 0.855)** | **99.65%** | **p=0.001** | **p=0.001** |  |
| Countries | **Taiwan** | **3 (213, 642)** | **0.251 (0.069, 0.602)** | **97.05%** | **p=0.155** | **p=0.001** | **p=0.221** |
|  | **Italy** | **4 (132, 233)** | **0.802 (0.423, 0.957)** | **92.88%** | **p=0.108** | **p=0.001** |  |
|  | **Iran** | **10 (1129, 1447)** | **0.865 (0.731, 0.937)** | **96.29%** | **p=0.001** | **p=0.001** |  |
|  | **Saudi Arabia** | **4 (441, 551)** | **0.872 (0.615, 0.967)** | **95.96%** | **p=0.010** | **p=0.001** |  |
|  | **South Korea** | **5 (980, 1235)** | **0.721 (0.435, 0.897)** | **97.38%** | **p=0.124** | **p=0.001** |  |
|  | **Brazil** | **9 (2187, 2770)** | **0.829 (0.695, 0.911)** | **96.29%** | **p=0.001** | **p=0.001** |  |
|  | **China** | **20 (11137, 15122)** | **0.710 (0.644, 0.768)** | **97.35%** | **p=0.001** | **p=0.001** |  |
|  | **France** | **2 (111, 284)** | **0.391 (0.336, 0.449)** | **0.00%** | **p=0.001** | **p=0.343** |  |
|  | **Romania** | **4 (70, 102)** | **0.695 (0.210, 0.951)** | **89.75%** | **p=0.453** | **p=0.001** |  |
|  | **Japan** | **1 (9, 9)** | **0.950 (0.525, 0.997)** | **0.00%** | **p=0.042** | **p=0.999** |  |
|  | **Uganda** | **1 (9, 29)** | **0.310 (0.170, 0.497)** | **0.00%** | **p=0.047** | **p=0.999** |  |
|  | **Turkey** | **8 (1421, 1616)** | **0.795 (0.530, 0.930)** | **97.47%** | **p=0.032** | **p=0.001** |  |
|  | **Vietnam** | **2 (2882, 3654)** | **0.789 (0.775, 0.802)** | **0.00%** | **p=0.001** | **p=0.446** |  |
|  | **Malaysia** | **1 (128, 162)** | **0.790 (0.721, 0.846)** | **0.00%** | **p=0.001** | **p=0.999** |  |
|  | **Thailand** | **8 (941, 1267)** | **0.713 (0.546, 0.837)** | **95.86%** | **p=0.014** | **p=0.001** |  |
|  | **Colombia** | **1 (146, 220)** | **0.664 (0.599, 0.723)** | **0.00%** | **p=0.001** | **p=0.999** |  |
|  | **Egypt** | **7 (413, 495)** | **0.832 (0.718, 0.906)** | **81.57%** | **p=0.001** | **p=0.001** |  |
|  | **Switzerland** | **2 (129, 810)** | **0.205 (0.039, 0.621)** | **98.80%** | **p=0.151** | **p=0.001** |  |
|  | **Germany** | **2 (3647, 46668)** | **0.226 (0.007, 0.922)** | **99.98%** | **p=0.514** | **p=0.001** |  |
|  | **Lebanon** | **1 (78, 100)** | **0.780 (0.688, 0.851)** | **0.00%** | **p=0.001** | **p=0.999** |  |
|  | **India** | **10 (346, 581)** | **0.698 (0.496, 0.845)** | **92.64%** | **p=0.054** | **p=0.001** |  |
|  | **Iraq** | **4 (269, 296)** | **0.863 (0.535, 0.972)** | **91.63%** | **p=0.034** | **p=0.001** |  |
|  | **Tunisia** | **2 (988, 1099)** | **0.903 (0.861, 0.933)** | **72.22%** | **p=0.001** | **p=0.058** |  |
|  | **Oman** | **2 (154, 212)** | **0.739 (0.633, 0.823)** | **43.40%** | **p=0.001** | **p=0.184** |  |
|  | **Ethiopia** | **2 (15, 31)** | **0.484 (0.316, 0.656)** | **0.00%** | **p=0.859** | **p=0.594** |  |
|  | **Mexico** | **1 (210, 252)** | **0.833 (0.782, 0.874)** | **0.00%** | **p=0.001** | **p=0.999** |  |
|  | **Georgia** | **1 (17, 20)** | **0.850 (0.624, 0.951)** | **0.00%** | **p=0.006** | **p=0.999** |  |
|  | **Kenya** | **1 (403, 590)** | **0.683 (0.644, 0.719)** | **0.00%** | **p=0.001** | **p=0.999** |  |
|  | **US** | **4 (3734, 5888)** | **0.681 (0.538, 0.797)** | **93.49%** | **p=0.014** | **p=0.001** |  |
|  | **UK** | **1 (59, 66)** | **0.894 (0.794, 0.949)** | **0.00%** | **p=0.001** | **p=0.999** |  |
|  | **Morocco** | **2 (81, 140)** | **0.813 (0.032, 0.998)** | **95.66%** | **p=0.555** | **p=0.001** |  |
|  | **Greece** | **1 (347, 347)** | **0.999 (0.977, 1.000)** | **0.00%** | **p=0.001** | **p=0.999** |  |
|  | **Indonesia** | **1 (30, 55)** | **0.545 (0.414, 0.671)** | **0.00%** | **p=0.501** | **p=0.999** |  |
|  | **Spain** | **2 (1201, 1579)** | **0.709 (0.282, 0.938)** | **99.52%** | **p=0.340** | **p=0.001** |  |
|  | **Pakistan** | **3 (89, 168)** | **0.813 (0.159, 0.990)** | **94.59%** | **p=0.358** | **p=0.001** |  |
|  | **Russia** | **1 (77, 96)** | **0.802 (0.710, 0.870)** | **0.00%** | **p=0.001** | **p=0.999** |  |
|  | **South Africa** | **1 (10, 21)** | **0.476 (0.279, 0.682)** | **0.00%** | **p=0.827** | **p=0.999** |  |
|  | **Nepal** | **1 (193, 196)** | **0.985 (0.954, 0.995)** | **0.00%** | **p=0.001** | **p=0.999** |  |
|  | **Ukraine** | **2 (575, 772)** | **0.745 (0.713, 0.774)** | **0.00%** | **p=0.001** | **p=0.569** |  |
|  | **Tanzania** | **1 (2, 6)** | **0.333 (0.084, 0.732)** | **0.00%** | **p=0.423** | **p=0.999** |  |
|  | **Serbia** | **1 (111, 117)** | **0.949 (0.891, 0.977)** | **0.00%** | **p=0.001** | **p=0.999** |  |
|  | **Gabon** | **1 (4, 15)** | **0.267 (0.104, 0.533)** | **0.00%** | **p=0.083** | **p=0.999** |  |
| Continents | **Asia** | **85 (20457, 27333)** | **0.746 (0.710, 0.779)** | **96.33%** | **p=0.001** | **p=0.001** | **p=0.665** |
|  | **Europe** | **22 (6459, 51074)** | **0.714 (0.465, 0.878)** | **99.81%** | **p=0.089** | **p=0.001** |  |
|  | **Americas** | **15 (6277, 9130)** | **0.772 (0.698, 0.832)** | **96.88%** | **p=0.001** | **p=0.001** |  |
|  | **Africa** | **18 (1925, 2426)** | **0.720 (0.601, 0.815)** | **94.31%** | **p=0.001** | **p=0.001** |  |
|  | **NA** | **6 (3957, 5144)** | **0.816 (0.725, 0.882)** | **98.07%** | **p=0.001** | **p=0.001** |  |
| Guideline | **CLSI** | **112 (27196, 79672)** | **0.749 (0.679, 0.808)** | **99.54%** | **p=0.001** | **p=0.001** | **p=0.612** |
|  | **EUCAST** | **13 (3156, 4456)** | **0.692 (0.477, 0.847)** | **98.90%** | **p=0.079** | **p=0.001** |  |
| Ast.methos.class | **MIC Based methods** | **69 (11435, 15781)** | **0.749 (0.709, 0.785)** | **95.26%** | **p=0.001** | **p=0.001** | **p=0.387** |
|  | **Disk diffusion** | **54 (12807, 18502)** | **0.721 (0.674, 0.763)** | **96.50%** | **p=0.001** | **p=0.001** |  |
|  | **Mixed Technique** | **11 (3719, 4848)** | **0.788 (0.693, 0.860)** | **97.04%** | **p=0.001** | **p=0.001** |  |
| Risk.of.bias | **Low Risk** | **128 (29880, 82428)** | **0.756 (0.691, 0.811)** | **99.50%** | **p=0.001** | **p=0.001** | **p=0.568** |
|  | **Some Concern** | **18 (9195, 12679)** | **0.769 (0.650, 0.856)** | **98.72%** | **p=0.001** | **p=0.001** |  |
| Sample.size | **Small sample size** | **127 (10167, 13933)** | **0.758 (0.718, 0.795)** | **94.84%** | **p=0.001** | **p=0.001** | **p=0.286** |
|  | **Larg sample size** | **19 (28908, 81174)** | **0.683 (0.487, 0.831)** | **99.93%** | **p=0.066** | **p=0.001** |  |
| Overall | **NA** | **329 (240865, 345494)** | **0.735 (0.718, 0.752)** | **86.96%** | **p=0.001** | **p=0.001** | **NA** |
| Year group | **1995-2014** | **192 (31971, 54382)** | **0.574 (0.534, 0.613)** | **97.91%** | **p=0.001** | **p=0.001** | **p=0.001** |
|  | **2015-2019** | **167 (107233, 160253)** | **0.763 (0.735, 0.789)** | **99.03%** | **p=0.001** | **p=0.001** |  |
|  | **2020-2023** | **300 (101661, 130859)** | **0.807 (0.781, 0.831)** | **98.75%** | **p=0.001** | **p=0.001** |  |
| Pre-post COVID-19 | **1995-2019** | **359 (139204, 214635)** | **0.670 (0.647, 0.693)** | **98.66%** | **p=0.001** | **p=0.001** | **p=0.001** |
|  | **2020-2023** | **300 (101661, 130859)** | **0.807 (0.781, 0.831)** | **98.75%** | **p=0.001** | **p=0.001** |  |
| Countries | **Taiwan** | **18 (10561, 14190)** | **0.478 (0.324, 0.635)** | **98.31%** | **p=0.787** | **p=0.001** | **p=0.001** |
|  | **Germany** | **3 (87, 153)** | **0.571 (0.131, 0.922)** | **90.67%** | **p=0.797** | **p=0.001** |  |
|  | **Greece** | **12 (13603, 16579)** | **0.761 (0.566, 0.886)** | **99.55%** | **p=0.011** | **p=0.001** |  |
|  | **Slovakia** | **2 (2, 67)** | **0.044 (0.007, 0.222)** | **35.84%** | **p=0.001** | **p=0.212** |  |
|  | **Bulgaria** | **3 (53, 110)** | **0.624 (0.039, 0.986)** | **94.77%** | **p=0.790** | **p=0.001** |  |
|  | **Turkey** | **39 (12626, 15065)** | **0.849 (0.787, 0.895)** | **97.81%** | **p=0.001** | **p=0.001** |  |
|  | **Italy** | **11 (1133, 1532)** | **0.818 (0.619, 0.926)** | **97.09%** | **p=0.004** | **p=0.001** |  |
|  | **UK** | **4 (141, 163)** | **0.813 (0.462, 0.957)** | **80.10%** | **p=0.075** | **p=0.002** |  |
|  | **Japan** | **6 (94, 766)** | **0.505 (0.099, 0.905)** | **95.06%** | **p=0.986** | **p=0.001** |  |
|  | **Brazil** | **20 (1367, 1722)** | **0.824 (0.656, 0.920)** | **94.61%** | **p=0.001** | **p=0.001** |  |
|  | **Thailand** | **11 (43611, 45247)** | **0.957 (0.878, 0.986)** | **98.81%** | **p=0.001** | **p=0.001** |  |
|  | **China** | **93 (82970, 126185)** | **0.685 (0.654, 0.714)** | **98.61%** | **p=0.001** | **p=0.001** |  |
|  | **Australia** | **3 (162, 233)** | **0.718 (0.573, 0.828)** | **77.23%** | **p=0.004** | **p=0.012** |  |
|  | **Iraq** | **18 (462, 739)** | **0.684 (0.556, 0.789)** | **87.61%** | **p=0.006** | **p=0.001** |  |
|  | **Estonia** | **2 (12, 223)** | **0.054 (0.031, 0.093)** | **0.00%** | **p=0.001** | **p=0.595** |  |
|  | **Belgium** | **1 (17, 18)** | **0.944 (0.693, 0.992)** | **0.00%** | **p=0.006** | **p=0.999** |  |
|  | **South Africa** | **9 (12174, 20644)** | **0.679 (0.461, 0.840)** | **99.82%** | **p=0.105** | **p=0.001** |  |
|  | **US** | **13 (2855, 5004)** | **0.556 (0.411, 0.692)** | **98.60%** | **p=0.453** | **p=0.001** |  |
|  | **Spain** | **8 (1249, 2348)** | **0.655 (0.538, 0.756)** | **93.84%** | **p=0.010** | **p=0.001** |  |
|  | **Croatia** | **8 (248, 557)** | **0.597 (0.222, 0.885)** | **93.09%** | **p=0.641** | **p=0.001** |  |
|  | **Georgia** | **1 (3, 129)** | **0.023 (0.008, 0.070)** | **0.00%** | **p=0.001** | **p=0.999** |  |
|  | **Poland** | **10 (952, 1701)** | **0.575 (0.346, 0.775)** | **96.94%** | **p=0.529** | **p=0.001** |  |
|  | **South Korea** | **13 (886, 1063)** | **0.901 (0.796, 0.955)** | **92.35%** | **p=0.001** | **p=0.001** |  |
|  | **Oman** | **3 (1539, 2167)** | **0.700 (0.501, 0.844)** | **92.91%** | **p=0.049** | **p=0.001** |  |
|  | **Iran** | **93 (8720, 11674)** | **0.877 (0.839, 0.906)** | **96.16%** | **p=0.001** | **p=0.001** |  |
|  | **Singapore** | **1 (98, 139)** | **0.705 (0.624, 0.775)** | **0.00%** | **p=0.001** | **p=0.999** |  |
|  | **Saudi Arabia** | **23 (1752, 4201)** | **0.764 (0.570, 0.888)** | **98.53%** | **p=0.010** | **p=0.001** |  |
|  | **India** | **52 (8116, 9920)** | **0.722 (0.636, 0.794)** | **96.87%** | **p=0.001** | **p=0.001** |  |
|  | **Canada** | **4 (17, 102)** | **0.337 (0.057, 0.810)** | **82.14%** | **p=0.533** | **p=0.001** |  |
|  | **Ireland** | **1 (1, 25)** | **0.040 (0.006, 0.235)** | **0.00%** | **p=0.002** | **p=0.999** |  |
|  | **Mexico** | **11 (3517, 5608)** | **0.755 (0.667, 0.825)** | **96.07%** | **p=0.001** | **p=0.001** |  |
|  | **Haiti** | **1 (0, 3)** | **0.125 (0.007, 0.734)** | **0.00%** | **p=0.198** | **p=0.999** |  |
|  | **France** | **3 (180, 196)** | **0.912 (0.788, 0.967)** | **18.92%** | **p=0.001** | **p=0.291** |  |
|  | **Colombia** | **3 (76, 105)** | **0.739 (0.525, 0.879)** | **73.01%** | **p=0.030** | **p=0.025** |  |
|  | **Syria** | **1 (183, 260)** | **0.704 (0.646, 0.756)** | **0.00%** | **p=0.001** | **p=0.999** |  |
|  | **Portugal** | **1 (1, 1)** | **0.750 (0.109, 0.987)** | **0.00%** | **p=0.501** | **p=0.999** |  |
|  | **Libya** | **4 (166, 229)** | **0.889 (0.623, 0.975)** | **79.20%** | **p=0.010** | **p=0.002** |  |
|  | **Egypt** | **20 (561, 718)** | **0.794 (0.697, 0.866)** | **79.07%** | **p=0.001** | **p=0.001** |  |
|  | **Kuwait** | **2 (143, 211)** | **0.695 (0.213, 0.950)** | **97.47%** | **p=0.449** | **p=0.001** |  |
|  | **Jordan** | **4 (654, 903)** | **0.843 (0.584, 0.954)** | **96.64%** | **p=0.014** | **p=0.001** |  |
|  | **Pakistan** | **13 (2190, 2515)** | **0.801 (0.693, 0.877)** | **93.61%** | **p=0.001** | **p=0.001** |  |
|  | **Honduras** | **1 (1, 1)** | **0.750 (0.109, 0.987)** | **0.00%** | **p=0.501** | **p=0.999** |  |
|  | **Malaysia** | **6 (321, 446)** | **0.703 (0.593, 0.793)** | **76.19%** | **p=0.001** | **p=0.001** |  |
|  | **Vietnam** | **7 (1249, 1448)** | **0.783 (0.591, 0.901)** | **95.85%** | **p=0.006** | **p=0.001** |  |
|  | **Nepal** | **4 (383, 438)** | **0.893 (0.777, 0.952)** | **83.05%** | **p=0.001** | **p=0.001** |  |
|  | **Nigeria** | **3 (125, 204)** | **0.619 (0.372, 0.818)** | **84.28%** | **p=0.346** | **p=0.002** |  |
|  | **Uganda** | **1 (1, 7)** | **0.143 (0.020, 0.581)** | **0.00%** | **p=0.097** | **p=0.999** |  |
|  | **Indonesia** | **7 (778, 1487)** | **0.598 (0.393, 0.774)** | **91.34%** | **p=0.350** | **p=0.001** |  |
|  | **Bolivia** | **2 (84, 131)** | **0.769 (0.269, 0.968)** | **91.98%** | **p=0.285** | **p=0.001** |  |
|  | **Lebanon** | **3 (151, 169)** | **0.922 (0.764, 0.977)** | **61.69%** | **p=0.001** | **p=0.074** |  |
|  | **Peru** | **4 (457, 880)** | **0.389 (0.066, 0.852)** | **97.32%** | **p=0.688** | **p=0.001** |  |
|  | **Kazakhstan** | **3 (196, 252)** | **0.738 (0.502, 0.888)** | **70.18%** | **p=0.049** | **p=0.035** |  |
|  | **Philippines** | **2 (307, 314)** | **0.961 (0.190, 1.000)** | **94.54%** | **p=0.176** | **p=0.001** |  |
|  | **Romania** | **3 (137, 166)** | **0.825 (0.564, 0.945)** | **86.21%** | **p=0.019** | **p=0.001** |  |
|  | **Zimbabwe** | **1 (336, 616)** | **0.545 (0.506, 0.584)** | **0.00%** | **p=0.024** | **p=0.999** |  |
|  | **Bangladesh** | **3 (121, 183)** | **0.768 (0.217, 0.975)** | **96.72%** | **p=0.344** | **p=0.001** |  |
|  | **Ghana** | **3 (48, 87)** | **0.495 (0.062, 0.935)** | **88.87%** | **p=0.987** | **p=0.001** |  |
|  | **Serbia** | **2 (286, 301)** | **0.965 (0.802, 0.995)** | **55.49%** | **p=0.001** | **p=0.134** |  |
|  | **Sweden** | **1 (36, 80)** | **0.450 (0.345, 0.560)** | **0.00%** | **p=0.372** | **p=0.999** |  |
|  | **Sierra Leone** | **2 (1, 19)** | **0.075 (0.015, 0.300)** | **0.00%** | **p=0.003** | **p=0.926** |  |
|  | **Ethiopia** | **4 (36, 81)** | **0.430 (0.262, 0.615)** | **49.89%** | **p=0.460** | **p=0.112** |  |
|  | **Mongolia** | **1 (2, 5)** | **0.400 (0.100, 0.800)** | **0.00%** | **p=0.657** | **p=0.999** |  |
|  | **Sudan** | **2 (74, 82)** | **0.903 (0.736, 0.969)** | **54.66%** | **p=0.001** | **p=0.138** |  |
|  | **Cyprus** | **1 (189, 218)** | **0.867 (0.815, 0.906)** | **0.00%** | **p=0.001** | **p=0.999** |  |
|  | **Finland** | **1 (1, 1)** | **0.750 (0.109, 0.987)** | **0.00%** | **p=0.501** | **p=0.999** |  |
|  | **Namibia** | **1 (101, 108)** | **0.935 (0.870, 0.969)** | **0.00%** | **p=0.001** | **p=0.999** |  |
|  | **Tanzania** | **1 (2, 12)** | **0.167 (0.042, 0.477)** | **0.00%** | **p=0.038** | **p=0.999** |  |
|  | **Myanmar** | **2 (62, 101)** | **0.614 (0.516, 0.703)** | **0.00%** | **p=0.024** | **p=0.717** |  |
|  | **Somalia** | **1 (12, 24)** | **0.500 (0.310, 0.690)** | **0.00%** | **p=0.999** | **p=0.999** |  |
|  | **Hungary** | **1 (15, 55)** | **0.273 (0.172, 0.404)** | **0.00%** | **p=0.001** | **p=0.999** |  |
|  | **Morocco** | **1 (165, 191)** | **0.864 (0.808, 0.906)** | **0.00%** | **p=0.001** | **p=0.999** |  |
|  | **Russia** | **1 (5, 8)** | **0.625 (0.285, 0.875)** | **0.00%** | **p=0.484** | **p=0.999** |  |
|  | **Palestine** | **2 (9, 15)** | **0.561 (0.033, 0.979)** | **82.91%** | **p=0.894** | **p=0.016** |  |
|  | **Zambia** | **1 (3, 15)** | **0.200 (0.066, 0.470)** | **0.00%** | **p=0.032** | **p=0.999** |  |
|  | **North Korea** | **1 (147, 167)** | **0.880 (0.822, 0.921)** | **0.00%** | **p=0.001** | **p=0.999** |  |
| Continents | **Asia** | **433 (178523, 240617)** | **0.765 (0.745, 0.783)** | **98.55%** | **p=0.001** | **p=0.001** | **p=0.001** |
|  | **Europe** | **78 (18159, 24284)** | **0.679 (0.597, 0.750)** | **98.48%** | **p=0.001** | **p=0.001** |  |
|  | **NA** | **32 (21842, 43767)** | **0.598 (0.531, 0.662)** | **99.17%** | **p=0.004** | **p=0.001** |  |
|  | **Americas** | **59 (8374, 13556)** | **0.670 (0.607, 0.728)** | **96.91%** | **p=0.001** | **p=0.001** |  |
|  | **Oceania** | **3 (162, 233)** | **0.718 (0.573, 0.828)** | **77.23%** | **p=0.004** | **p=0.012** |  |
|  | **Africa** | **54 (13805, 23037)** | **0.695 (0.608, 0.770)** | **98.89%** | **p=0.001** | **p=0.001** |  |
| Guideline | **CLSI** | **518 (218475, 308866)** | **0.724 (0.705, 0.742)** | **98.78%** | **p=0.001** | **p=0.001** | **p=0.002** |
|  | **EUCAST** | **50 (11245, 14034)** | **0.840 (0.764, 0.895)** | **98.33%** | **p=0.001** | **p=0.001** |  |
| Ast.methos.class | **MIC Based methods** | **341 (78796, 125046)** | **0.706 (0.680, 0.730)** | **98.34%** | **p=0.001** | **p=0.001** | **p=0.001** |
|  | **Disk diffusion** | **256 (73450, 114917)** | **0.772 (0.749, 0.794)** | **97.74%** | **p=0.001** | **p=0.001** |  |
|  | **Mixed Technique** | **36 (66884, 77251)** | **0.819 (0.731, 0.883)** | **99.68%** | **p=0.001** | **p=0.001** |  |
| Risk.of.bias | **Some Concern** | **113 (19368, 29278)** | **0.542 (0.486, 0.597)** | **97.52%** | **p=0.140** | **p=0.001** | **p=0.001** |
|  | **Low Risk** | **542 (221399, 316021)** | **0.768 (0.751, 0.785)** | **98.85%** | **p=0.001** | **p=0.001** |  |
|  | **High Risk** | **4 (98, 195)** | **0.545 (0.303, 0.767)** | **88.06%** | **p=0.730** | **p=0.001** |  |
| Sample.size | **Small sample size** | **589 (38045, 54018)** | **0.751 (0.730, 0.771)** | **94.59%** | **p=0.001** | **p=0.001** | **p=0.050** |
|  | **Larg sample size** | **70 (202820, 291476)** | **0.689 (0.638, 0.736)** | **99.83%** | **p=0.001** | **p=0.001** |  |
| Overall | **NA** | **41 (79708, 86406)** | **0.730 (0.632, 0.809)** | **99.28%** | **p=0.001** | **p=0.001** | **NA** |
| Year group | **1995-2014** | **14 (1441, 2473)** | **0.698 (0.565, 0.805)** | **96.12%** | **p=0.004** | **p=0.001** | **p=0.426** |
|  | **2015-2019** | **14 (36458, 40746)** | **0.673 (0.511, 0.802)** | **98.41%** | **p=0.037** | **p=0.001** |  |
|  | **2020-2023** | **13 (41809, 43187)** | **0.822 (0.567, 0.942)** | **97.41%** | **p=0.017** | **p=0.001** |  |
| Pre-post COVID-19 | **1995-2019** | **28 (37899, 43219)** | **0.697 (0.565, 0.802)** | **99.07%** | **p=0.004** | **p=0.001** | **p=0.268** |
|  | **2020-2023** | **13 (41809, 43187)** | **0.822 (0.567, 0.942)** | **97.41%** | **p=0.017** | **p=0.001** |  |
| Countries | **Spain** | **1 (19, 19)** | **0.975 (0.702, 0.998)** | **0.00%** | **p=0.011** | **p=0.999** | **p=0.078** |
|  | **US** | **6 (36072, 40535)** | **0.741 (0.397, 0.926)** | **99.69%** | **p=0.161** | **p=0.001** |  |
|  | **Colombia** | **1 (19, 34)** | **0.559 (0.392, 0.714)** | **0.00%** | **p=0.494** | **p=0.999** |  |
|  | **Saudi Arabia** | **1 (65, 84)** | **0.774 (0.672, 0.851)** | **0.00%** | **p=0.001** | **p=0.999** |  |
|  | **India** | **7 (435, 713)** | **0.419 (0.234, 0.630)** | **94.66%** | **p=0.456** | **p=0.001** |  |
|  | **France** | **1 (1, 1)** | **0.750 (0.109, 0.987)** | **0.00%** | **p=0.501** | **p=0.999** |  |
|  | **Taiwan** | **2 (77, 153)** | **0.526 (0.349, 0.696)** | **78.04%** | **p=0.781** | **p=0.033** |  |
|  | **Turkey** | **2 (99, 125)** | **0.771 (0.617, 0.875)** | **56.74%** | **p=0.001** | **p=0.128** |  |
|  | **Italy** | **1 (241, 246)** | **0.980 (0.952, 0.992)** | **0.00%** | **p=0.001** | **p=0.999** |  |
|  | **Iran** | **3 (334, 350)** | **0.957 (0.900, 0.982)** | **61.33%** | **p=0.001** | **p=0.075** |  |
|  | **Pakistan** | **1 (23, 30)** | **0.767 (0.585, 0.884)** | **0.00%** | **p=0.006** | **p=0.999** |  |
|  | **China** | **3 (463, 678)** | **0.697 (0.567, 0.802)** | **69.62%** | **p=0.004** | **p=0.037** |  |
|  | **Malaysia** | **1 (88, 128)** | **0.688 (0.602, 0.762)** | **0.00%** | **p=0.001** | **p=0.999** |  |
|  | **Bangladesh** | **1 (16, 71)** | **0.225 (0.143, 0.337)** | **0.00%** | **p=0.001** | **p=0.999** |  |
|  | **Egypt** | **2 (68, 80)** | **0.907 (0.129, 0.998)** | **88.24%** | **p=0.286** | **p=0.004** |  |
|  | **Thailand** | **2 (41486, 42751)** | **0.983 (0.902, 0.997)** | **55.11%** | **p=0.001** | **p=0.136** |  |
|  | **UK** | **1 (4, 10)** | **0.400 (0.158, 0.703)** | **0.00%** | **p=0.530** | **p=0.999** |  |
|  | **Iraq** | **2 (34, 52)** | **0.648 (0.511, 0.765)** | **0.00%** | **p=0.035** | **p=0.512** |  |
|  | **Nigeria** | **1 (15, 33)** | **0.455 (0.296, 0.623)** | **0.00%** | **p=0.602** | **p=0.999** |  |
|  | **Mexico** | **1 (39, 39)** | **0.987 (0.829, 0.999)** | **0.00%** | **p=0.002** | **p=0.999** |  |
| Continents | **Europe** | **4 (265, 276)** | **0.886 (0.350, 0.991)** | **90.30%** | **p=0.132** | **p=0.001** | **p=0.786** |
|  | **Americas** | **8 (36130, 40608)** | **0.772 (0.483, 0.925)** | **99.57%** | **p=0.063** | **p=0.001** |  |
|  | **NA** | **1 (110, 274)** | **0.401 (0.345, 0.461)** | **0.00%** | **p=0.001** | **p=0.999** |  |
|  | **Asia** | **25 (43120, 45135)** | **0.712 (0.529, 0.845)** | **99.12%** | **p=0.025** | **p=0.001** |  |
|  | **Africa** | **3 (83, 113)** | **0.697 (0.360, 0.903)** | **81.96%** | **p=0.246** | **p=0.004** |  |
| Ast.methos.class | **MIC Based methods** | **23 (1880, 3103)** | **0.690 (0.599, 0.769)** | **93.60%** | **p=0.001** | **p=0.001** | **p=0.344** |
|  | **Disk diffusion** | **15 (968, 1357)** | **0.691 (0.546, 0.807)** | **93.63%** | **p=0.011** | **p=0.001** |  |
|  | **Mixed Technique** | **2 (41355, 42626)** | **0.831 (0.098, 0.996)** | **97.24%** | **p=0.413** | **p=0.001** |  |
| Risk.of.bias | **Some Concern** | **11 (778, 1705)** | **0.542 (0.423, 0.656)** | **89.30%** | **p=0.491** | **p=0.001** | **p=0.024** |
|  | **Low Risk** | **30 (78930, 84701)** | **0.767 (0.682, 0.835)** | **99.01%** | **p=0.001** | **p=0.001** |  |
| Sample.size | **Small sample size** | **37 (2445, 3474)** | **0.694 (0.618, 0.761)** | **92.16%** | **p=0.001** | **p=0.001** | **p=0.197** |
|  | **Larg sample size** | **4 (77263, 82932)** | **0.854 (0.607, 0.957)** | **99.91%** | **p=0.009** | **p=0.001** |  |
| Overall | **NA** | **25 (2582, 3059)** | **0.837 (0.743, 0.901)** | **91.09%** | **p=0.001** | **p=0.001** | **NA** |
| Year group | **1995-2014** | **5 (56, 73)** | **0.744 (0.629, 0.833)** | **0.00%** | **p=0.001** | **p=0.753** | **p=0.013** |
|  | **2015-2019** | **4 (405, 408)** | **0.984 (0.853, 0.998)** | **78.32%** | **p=0.001** | **p=0.003** |  |
|  | **2020-2023** | **17 (2121, 2578)** | **0.792 (0.657, 0.883)** | **93.62%** | **p=0.001** | **p=0.001** |  |
| Pre-post COVID-19 | **1995-2019** | **9 (461, 481)** | **0.916 (0.778, 0.972)** | **74.71%** | **p=0.001** | **p=0.001** | **p=0.119** |
|  | **2020-2023** | **17 (2121, 2578)** | **0.792 (0.657, 0.883)** | **93.62%** | **p=0.001** | **p=0.001** |  |
| Countries | **Spain** | **1 (2, 3)** | **0.667 (0.154, 0.957)** | **0.00%** | **p=0.571** | **p=0.999** | **p=0.493** |
|  | **Taiwan** | **1 (24, 32)** | **0.750 (0.574, 0.870)** | **0.00%** | **p=0.007** | **p=0.999** |  |
|  | **Canada** | **1 (9, 9)** | **0.950 (0.525, 0.997)** | **0.00%** | **p=0.042** | **p=0.999** |  |
|  | **France** | **1 (1, 1)** | **0.750 (0.109, 0.987)** | **0.00%** | **p=0.501** | **p=0.999** |  |
|  | **Brazil** | **1 (20, 28)** | **0.714 (0.524, 0.850)** | **0.00%** | **p=0.028** | **p=0.999** |  |
|  | **China** | **2 (277, 285)** | **0.980 (0.584, 0.999)** | **84.00%** | **p=0.032** | **p=0.012** |  |
|  | **Iran** | **1 (127, 128)** | **0.992 (0.947, 0.999)** | **0.00%** | **p=0.001** | **p=0.999** |  |
|  | **Vietnam** | **1 (75, 75)** | **0.993 (0.903, 1.000)** | **0.00%** | **p=0.001** | **p=0.999** |  |
|  | **India** | **3 (29, 63)** | **0.484 (0.230, 0.747)** | **72.74%** | **p=0.911** | **p=0.026** |  |
|  | **Italy** | **3 (913, 989)** | **0.955 (0.052, 1.000)** | **96.09%** | **p=0.314** | **p=0.001** |  |
|  | **Egypt** | **3 (184, 203)** | **0.899 (0.649, 0.977)** | **85.31%** | **p=0.006** | **p=0.001** |  |
|  | **Somalia** | **2 (24, 55)** | **0.460 (0.044, 0.941)** | **94.48%** | **p=0.915** | **p=0.001** |  |
|  | **Turkey** | **3 (716, 933)** | **0.891 (0.573, 0.980)** | **81.77%** | **p=0.023** | **p=0.004** |  |
|  | **Pakistan** | **1 (82, 156)** | **0.526 (0.447, 0.603)** | **0.00%** | **p=0.522** | **p=0.999** |  |
|  | **Thailand** | **1 (97, 97)** | **0.995 (0.924, 1.000)** | **0.00%** | **p=0.001** | **p=0.999** |  |
|  | **Iraq** | **1 (2, 2)** | **0.833 (0.194, 0.990)** | **0.00%** | **p=0.299** | **p=0.999** |  |
| Continents | **Europe** | **5 (916, 993)** | **0.896 (0.218, 0.996)** | **92.71%** | **p=0.219** | **p=0.001** | **p=0.820** |
|  | **Asia** | **14 (1429, 1771)** | **0.851 (0.746, 0.918)** | **89.77%** | **p=0.001** | **p=0.001** |  |
|  | **Americas** | **2 (29, 37)** | **0.811 (0.425, 0.961)** | **44.56%** | **p=0.104** | **p=0.179** |  |
|  | **Africa** | **5 (208, 258)** | **0.781 (0.421, 0.946)** | **92.62%** | **p=0.117** | **p=0.001** |  |
| Guideline | **CLSI** | **19 (1341, 1741)** | **0.776 (0.672, 0.854)** | **88.47%** | **p=0.001** | **p=0.001** | **p=0.763** |
|  | **EUCAST** | **3 (913, 989)** | **0.955 (0.052, 1.000)** | **96.09%** | **p=0.314** | **p=0.001** |  |
| Ast.methos.class | **MIC Based methods** | **13 (1445, 1540)** | **0.947 (0.800, 0.988)** | **92.71%** | **p=0.001** | **p=0.001** | **p=0.086** |
|  | **Disk diffusion** | **12 (488, 655)** | **0.724 (0.544, 0.853)** | **89.50%** | **p=0.016** | **p=0.001** |  |
|  | **Mixed Technique** | **1 (649, 864)** | **0.751 (0.721, 0.779)** | **0.00%** | **p=0.001** | **p=0.999** |  |
| Risk.of.bias | **Low Risk** | **22 (2414, 2873)** | **0.836 (0.729, 0.906)** | **92.37%** | **p=0.001** | **p=0.001** | **p=0.871** |
|  | **Some Concern** | **4 (168, 186)** | **0.852 (0.578, 0.960)** | **79.48%** | **p=0.017** | **p=0.002** |  |
| Sample.size | **Small sample size** | **23 (1052, 1314)** | **0.835 (0.712, 0.912)** | **91.12%** | **p=0.001** | **p=0.001** | **p=0.260** |
|  | **Larg sample size** | **3 (1530, 1745)** | **0.963 (0.366, 0.999)** | **90.15%** | **p=0.093** | **p=0.001** |  |
| Overall | **NA** | **396 (277204, 436073)** | **0.743 (0.726, 0.760)** | **95.41%** | **p=0.001** | **p=0.001** | **NA** |
| Year group | **1995-2014** | **198 (32428, 55384)** | **0.573 (0.534, 0.611)** | **97.87%** | **p=0.001** | **p=0.001** | **p=0.001** |
|  | **2015-2019** | **217 (120819, 178873)** | **0.749 (0.723, 0.772)** | **98.89%** | **p=0.001** | **p=0.001** |  |
|  | **2020-2023** | **378 (123957, 201816)** | **0.810 (0.782, 0.834)** | **99.27%** | **p=0.001** | **p=0.001** |  |
| Pre-post COVID-19 | **1995-2019** | **415 (153247, 234257)** | **0.672 (0.651, 0.693)** | **98.59%** | **p=0.001** | **p=0.001** | **p=0.001** |
|  | **2020-2023** | **378 (123957, 201816)** | **0.810 (0.782, 0.834)** | **99.27%** | **p=0.001** | **p=0.001** |  |
| Countries | **Taiwan** | **20 (10771, 14584)** | **0.486 (0.349, 0.625)** | **98.28%** | **p=0.847** | **p=0.001** | **p=0.001** |
|  | **Germany** | **5 (3734, 46821)** | **0.373 (0.050, 0.869)** | **99.94%** | **p=0.674** | **p=0.001** |  |
|  | **Greece** | **13 (13950, 16926)** | **0.803 (0.630, 0.907)** | **99.52%** | **p=0.002** | **p=0.001** |  |
|  | **Slovakia** | **2 (2, 67)** | **0.044 (0.007, 0.222)** | **35.84%** | **p=0.001** | **p=0.212** |  |
|  | **Bulgaria** | **3 (53, 110)** | **0.624 (0.039, 0.986)** | **94.77%** | **p=0.790** | **p=0.001** |  |
|  | **Turkey** | **46 (14013, 16633)** | **0.842 (0.783, 0.887)** | **97.76%** | **p=0.001** | **p=0.001** |  |
|  | **Italy** | **15 (1265, 1765)** | **0.810 (0.650, 0.907)** | **96.52%** | **p=0.001** | **p=0.001** |  |
|  | **UK** | **5 (200, 229)** | **0.829 (0.578, 0.945)** | **82.76%** | **p=0.014** | **p=0.001** |  |
|  | **Japan** | **7 (103, 775)** | **0.601 (0.149, 0.928)** | **94.76%** | **p=0.710** | **p=0.001** |  |
|  | **Brazil** | **29 (3554, 4492)** | **0.816 (0.727, 0.882)** | **95.14%** | **p=0.001** | **p=0.001** |  |
|  | **Thailand** | **19 (44552, 46514)** | **0.886 (0.774, 0.946)** | **98.99%** | **p=0.001** | **p=0.001** |  |
|  | **China** | **108 (91896, 137610)** | **0.691 (0.662, 0.718)** | **98.62%** | **p=0.001** | **p=0.001** |  |
|  | **Australia** | **3 (162, 233)** | **0.718 (0.573, 0.828)** | **77.23%** | **p=0.004** | **p=0.012** |  |
|  | **Iraq** | **22 (731, 1035)** | **0.723 (0.602, 0.819)** | **89.57%** | **p=0.001** | **p=0.001** |  |
|  | **Estonia** | **2 (12, 223)** | **0.054 (0.031, 0.093)** | **0.00%** | **p=0.001** | **p=0.595** |  |
|  | **Belgium** | **1 (17, 18)** | **0.944 (0.693, 0.992)** | **0.00%** | **p=0.006** | **p=0.999** |  |
|  | **South Africa** | **10 (12184, 20665)** | **0.662 (0.452, 0.823)** | **99.80%** | **p=0.127** | **p=0.001** |  |
|  | **US** | **17 (6589, 10892)** | **0.587 (0.488, 0.679)** | **98.38%** | **p=0.084** | **p=0.001** |  |
|  | **Spain** | **10 (2450, 3927)** | **0.692 (0.546, 0.807)** | **98.17%** | **p=0.011** | **p=0.001** |  |
|  | **Croatia** | **8 (248, 557)** | **0.597 (0.222, 0.885)** | **93.09%** | **p=0.641** | **p=0.001** |  |
|  | **Georgia** | **2 (20, 149)** | **0.268 (0.002, 0.987)** | **97.55%** | **p=0.713** | **p=0.001** |  |
|  | **Poland** | **10 (952, 1701)** | **0.575 (0.346, 0.775)** | **96.94%** | **p=0.529** | **p=0.001** |  |
|  | **South Korea** | **18 (1866, 2298)** | **0.846 (0.746, 0.912)** | **94.58%** | **p=0.001** | **p=0.001** |  |
|  | **Oman** | **5 (1693, 2379)** | **0.737 (0.639, 0.817)** | **86.71%** | **p=0.001** | **p=0.001** |  |
|  | **Iran** | **101 (9662, 12919)** | **0.873 (0.837, 0.902)** | **96.16%** | **p=0.001** | **p=0.001** |  |
|  | **Singapore** | **1 (98, 139)** | **0.705 (0.624, 0.775)** | **0.00%** | **p=0.001** | **p=0.999** |  |
|  | **Saudi Arabia** | **25 (1976, 4519)** | **0.770 (0.598, 0.882)** | **98.47%** | **p=0.003** | **p=0.001** |  |
|  | **India** | **62 (8462, 10501)** | **0.719 (0.639, 0.786)** | **96.71%** | **p=0.001** | **p=0.001** |  |
|  | **Canada** | **4 (17, 102)** | **0.337 (0.057, 0.810)** | **82.14%** | **p=0.533** | **p=0.001** |  |
|  | **Ireland** | **1 (1, 25)** | **0.040 (0.006, 0.235)** | **0.00%** | **p=0.002** | **p=0.999** |  |
|  | **Mexico** | **12 (3727, 5860)** | **0.765 (0.680, 0.832)** | **96.34%** | **p=0.001** | **p=0.001** |  |
|  | **Haiti** | **1 (0, 3)** | **0.125 (0.007, 0.734)** | **0.00%** | **p=0.198** | **p=0.999** |  |
|  | **France** | **5 (291, 480)** | **0.818 (0.406, 0.967)** | **95.71%** | **p=0.118** | **p=0.001** |  |
|  | **Colombia** | **4 (222, 325)** | **0.700 (0.582, 0.796)** | **61.97%** | **p=0.001** | **p=0.048** |  |
|  | **Syria** | **1 (183, 260)** | **0.704 (0.646, 0.756)** | **0.00%** | **p=0.001** | **p=0.999** |  |
|  | **Portugal** | **1 (1, 1)** | **0.750 (0.109, 0.987)** | **0.00%** | **p=0.501** | **p=0.999** |  |
|  | **Libya** | **4 (166, 229)** | **0.889 (0.623, 0.975)** | **79.20%** | **p=0.010** | **p=0.002** |  |
|  | **Egypt** | **27 (974, 1213)** | **0.803 (0.733, 0.859)** | **79.24%** | **p=0.001** | **p=0.001** |  |
|  | **Kuwait** | **2 (143, 211)** | **0.695 (0.213, 0.950)** | **97.47%** | **p=0.449** | **p=0.001** |  |
|  | **Jordan** | **4 (654, 903)** | **0.843 (0.584, 0.954)** | **96.64%** | **p=0.014** | **p=0.001** |  |
|  | **Pakistan** | **16 (2279, 2683)** | **0.796 (0.671, 0.882)** | **95.55%** | **p=0.001** | **p=0.001** |  |
|  | **Honduras** | **1 (1, 1)** | **0.750 (0.109, 0.987)** | **0.00%** | **p=0.501** | **p=0.999** |  |
|  | **Malaysia** | **7 (449, 608)** | **0.720 (0.632, 0.795)** | **75.12%** | **p=0.001** | **p=0.001** |  |
|  | **Vietnam** | **9 (4131, 5102)** | **0.803 (0.702, 0.876)** | **94.93%** | **p=0.001** | **p=0.001** |  |
|  | **Romania** | **7 (207, 268)** | **0.757 (0.521, 0.899)** | **86.86%** | **p=0.034** | **p=0.001** |  |
|  | **Nepal** | **5 (576, 634)** | **0.929 (0.828, 0.972)** | **88.54%** | **p=0.001** | **p=0.001** |  |
|  | **Uganda** | **2 (10, 36)** | **0.285 (0.160, 0.455)** | **0.00%** | **p=0.015** | **p=0.389** |  |
|  | **Nigeria** | **3 (125, 204)** | **0.619 (0.372, 0.818)** | **84.28%** | **p=0.346** | **p=0.002** |  |
|  | **Indonesia** | **8 (808, 1542)** | **0.591 (0.425, 0.739)** | **89.92%** | **p=0.281** | **p=0.001** |  |
|  | **Switzerland** | **2 (129, 810)** | **0.205 (0.039, 0.621)** | **98.80%** | **p=0.151** | **p=0.001** |  |
|  | **Bolivia** | **2 (84, 131)** | **0.769 (0.269, 0.968)** | **91.98%** | **p=0.285** | **p=0.001** |  |
|  | **Lebanon** | **3 (145, 169)** | **0.917 (0.686, 0.983)** | **75.71%** | **p=0.004** | **p=0.016** |  |
|  | **Tunisia** | **2 (988, 1099)** | **0.903 (0.861, 0.933)** | **72.22%** | **p=0.001** | **p=0.058** |  |
|  | **Peru** | **4 (457, 880)** | **0.389 (0.066, 0.852)** | **97.32%** | **p=0.688** | **p=0.001** |  |
|  | **Kazakhstan** | **3 (196, 252)** | **0.738 (0.502, 0.888)** | **70.18%** | **p=0.049** | **p=0.035** |  |
|  | **Ethiopia** | **6 (51, 112)** | **0.466 (0.357, 0.578)** | **20.47%** | **p=0.552** | **p=0.279** |  |
|  | **Philippines** | **2 (307, 314)** | **0.961 (0.190, 1.000)** | **94.54%** | **p=0.176** | **p=0.001** |  |
|  | **Zimbabwe** | **1 (336, 616)** | **0.545 (0.506, 0.584)** | **0.00%** | **p=0.024** | **p=0.999** |  |
|  | **Bangladesh** | **3 (121, 183)** | **0.768 (0.217, 0.975)** | **96.72%** | **p=0.344** | **p=0.001** |  |
|  | **Kenya** | **1 (403, 590)** | **0.683 (0.644, 0.719)** | **0.00%** | **p=0.001** | **p=0.999** |  |
|  | **Ghana** | **3 (48, 87)** | **0.495 (0.062, 0.935)** | **88.87%** | **p=0.987** | **p=0.001** |  |
|  | **Serbia** | **3 (397, 418)** | **0.945 (0.911, 0.966)** | **14.45%** | **p=0.001** | **p=0.311** |  |
|  | **Sweden** | **1 (36, 80)** | **0.450 (0.345, 0.560)** | **0.00%** | **p=0.372** | **p=0.999** |  |
|  | **Morocco** | **3 (246, 331)** | **0.817 (0.298, 0.979)** | **97.62%** | **p=0.213** | **p=0.001** |  |
|  | **Russia** | **2 (82, 104)** | **0.772 (0.626, 0.872)** | **24.14%** | **p=0.001** | **p=0.251** |  |
|  | **Sierra Leone** | **2 (1, 19)** | **0.075 (0.015, 0.300)** | **0.00%** | **p=0.003** | **p=0.926** |  |
|  | **Mongolia** | **1 (2, 5)** | **0.400 (0.100, 0.800)** | **0.00%** | **p=0.657** | **p=0.999** |  |
|  | **Sudan** | **2 (74, 82)** | **0.903 (0.736, 0.969)** | **54.66%** | **p=0.001** | **p=0.138** |  |
|  | **Cyprus** | **1 (189, 218)** | **0.867 (0.815, 0.906)** | **0.00%** | **p=0.001** | **p=0.999** |  |
|  | **Finland** | **1 (1, 1)** | **0.750 (0.109, 0.987)** | **0.00%** | **p=0.501** | **p=0.999** |  |
|  | **Namibia** | **1 (101, 108)** | **0.935 (0.870, 0.969)** | **0.00%** | **p=0.001** | **p=0.999** |  |
|  | **Tanzania** | **2 (4, 18)** | **0.231 (0.088, 0.482)** | **0.00%** | **p=0.037** | **p=0.430** |  |
|  | **Ukraine** | **2 (575, 772)** | **0.745 (0.713, 0.774)** | **0.00%** | **p=0.001** | **p=0.569** |  |
|  | **Myanmar** | **2 (62, 101)** | **0.614 (0.516, 0.703)** | **0.00%** | **p=0.024** | **p=0.717** |  |
|  | **Somalia** | **1 (12, 24)** | **0.500 (0.310, 0.690)** | **0.00%** | **p=0.999** | **p=0.999** |  |
|  | **Hungary** | **1 (15, 55)** | **0.273 (0.172, 0.404)** | **0.00%** | **p=0.001** | **p=0.999** |  |
|  | **Palestine** | **2 (9, 15)** | **0.561 (0.033, 0.979)** | **82.91%** | **p=0.894** | **p=0.016** |  |
|  | **Gabon** | **1 (4, 15)** | **0.267 (0.104, 0.533)** | **0.00%** | **p=0.083** | **p=0.999** |  |
|  | **Zambia** | **1 (3, 15)** | **0.200 (0.066, 0.470)** | **0.00%** | **p=0.032** | **p=0.999** |  |
|  | **North Korea** | **1 (147, 167)** | **0.880 (0.822, 0.921)** | **0.00%** | **p=0.001** | **p=0.999** |  |
| Continents | **Asia** | **506 (196244, 263422)** | **0.762 (0.745, 0.779)** | **98.42%** | **p=0.001** | **p=0.001** | **p=0.001** |
|  | **Europe** | **100 (24618, 75358)** | **0.698 (0.592, 0.786)** | **99.62%** | **p=0.001** | **p=0.001** |  |
|  | **NA** | **38 (25799, 48911)** | **0.653 (0.590, 0.711)** | **99.28%** | **p=0.001** | **p=0.001** |  |
|  | **Americas** | **74 (14651, 22686)** | **0.694 (0.647, 0.737)** | **97.03%** | **p=0.001** | **p=0.001** |  |
|  | **Oceania** | **3 (162, 233)** | **0.718 (0.573, 0.828)** | **77.23%** | **p=0.004** | **p=0.012** |  |
|  | **Africa** | **72 (15730, 25463)** | **0.703 (0.631, 0.766)** | **98.63%** | **p=0.001** | **p=0.001** |  |
| Guideline | **CLSI** | **619 (243121, 384206)** | **0.735 (0.715, 0.753)** | **99.11%** | **p=0.001** | **p=0.001** | **p=0.034** |
|  | **EUCAST** | **63 (14401, 18490)** | **0.812 (0.740, 0.867)** | **98.48%** | **p=0.001** | **p=0.001** |  |
| Ast.methos.class | **MIC Based methods** | **403 (89854, 140157)** | **0.714 (0.692, 0.736)** | **98.20%** | **p=0.001** | **p=0.001** | **p=0.001** |
|  | **Disk diffusion** | **306 (84693, 130952)** | **0.761 (0.741, 0.781)** | **97.63%** | **p=0.001** | **p=0.001** |  |
|  | **Mixed Technique** | **46 (69808, 80708)** | **0.820 (0.747, 0.875)** | **99.59%** | **p=0.001** | **p=0.001** |  |
| Risk.of.bias | **Some Concern** | **129 (27140, 39660)** | **0.573 (0.522, 0.622)** | **97.85%** | **p=0.005** | **p=0.001** | **p=0.001** |
|  | **Low Risk** | **660 (249966, 396218)** | **0.771 (0.753, 0.787)** | **99.11%** | **p=0.001** | **p=0.001** |  |
|  | **High Risk** | **4 (98, 195)** | **0.545 (0.303, 0.767)** | **88.06%** | **p=0.730** | **p=0.001** |  |
| Sample.size | **Small sample size** | **706 (47610, 67011)** | **0.752 (0.733, 0.770)** | **94.63%** | **p=0.001** | **p=0.001** | **p=0.030** |
|  | **Larg sample size** | **87 (229594, 369062)** | **0.693 (0.639, 0.742)** | **99.87%** | **p=0.001** | **p=0.001** |  |


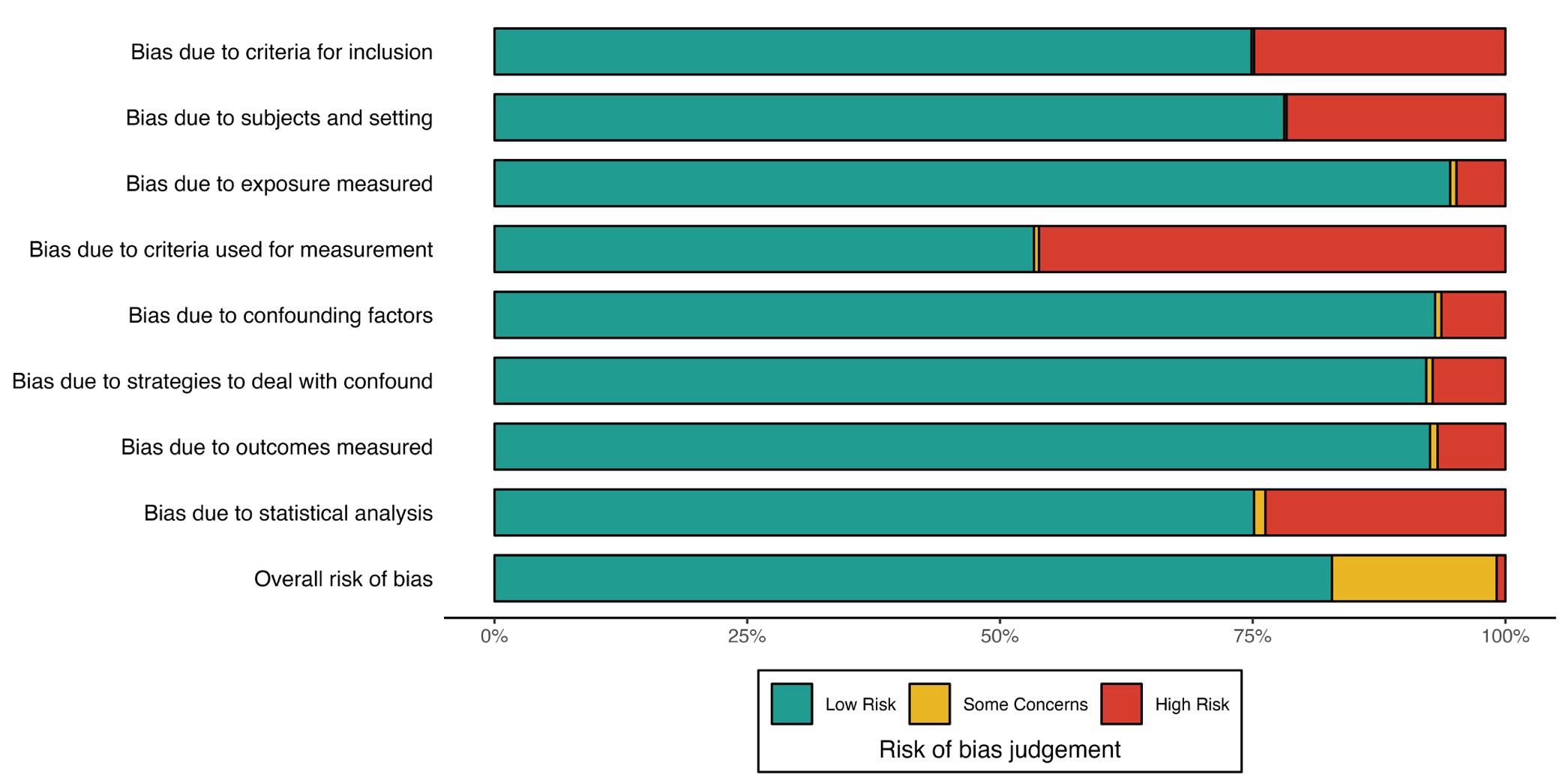


# Supplementary Figure 1: summary of risk of bias assessment or quality assessment of included studies

| 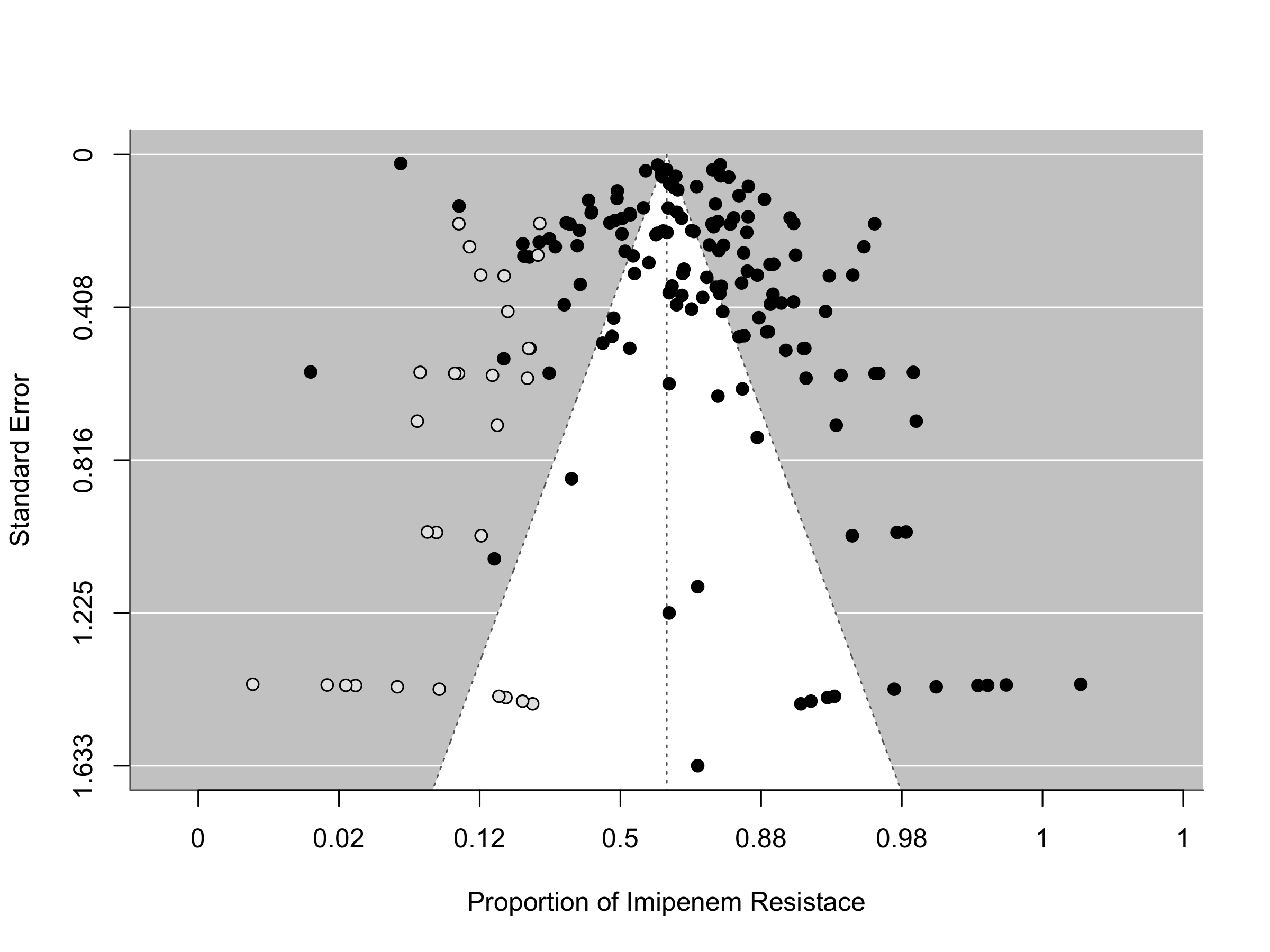  **A** | 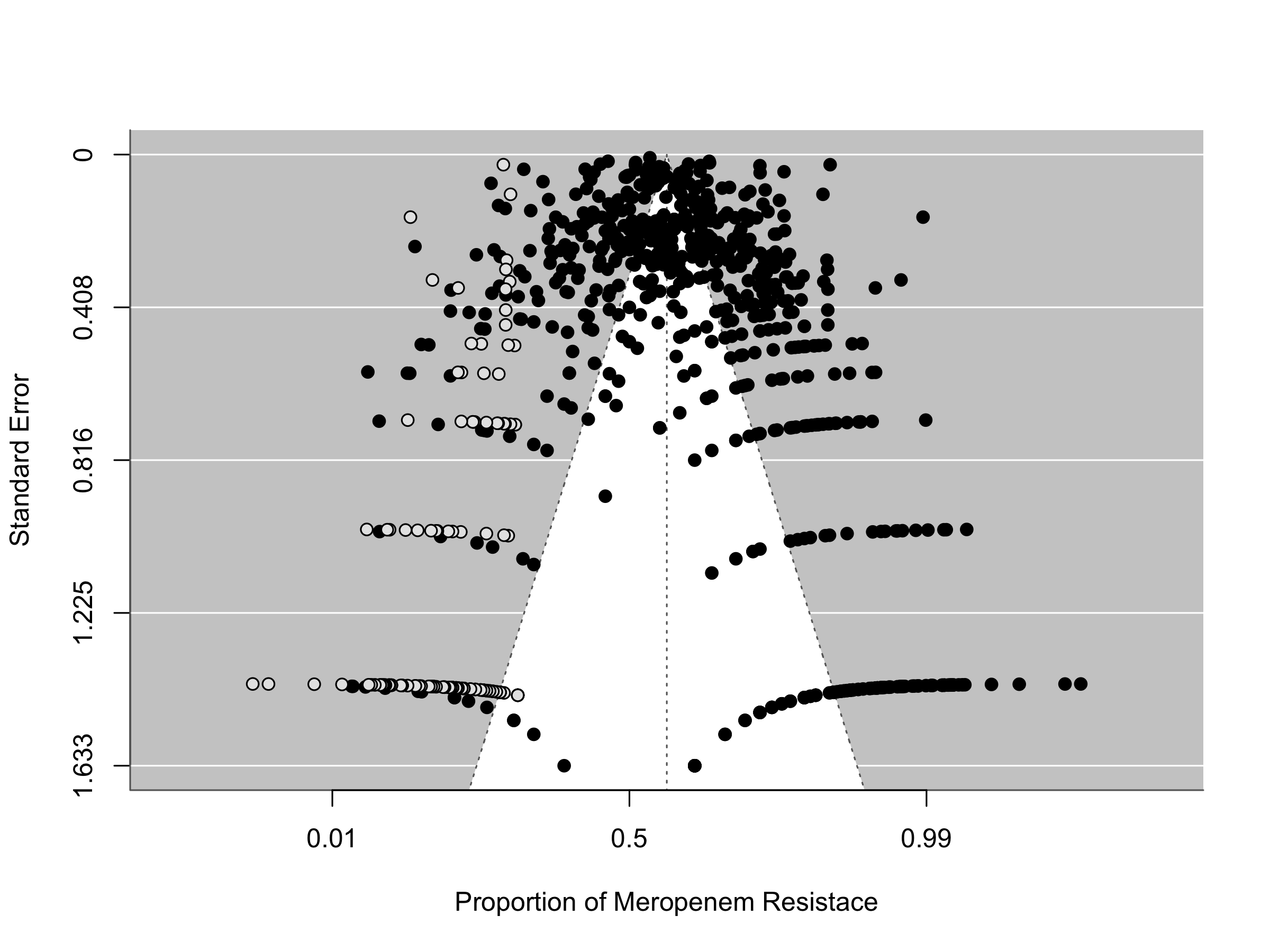  **B** | |
| --- | --- | --- |
| 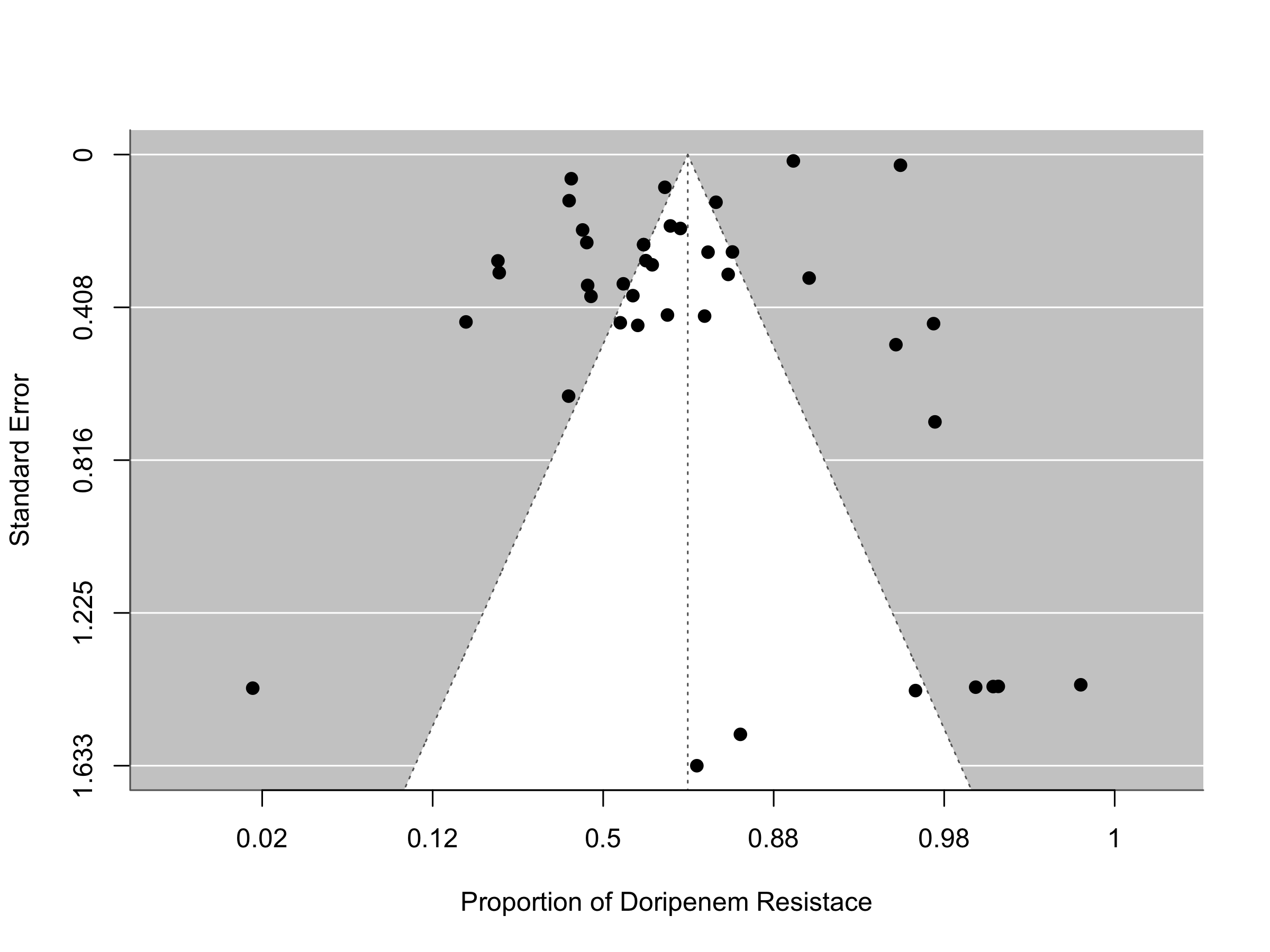  **C** | | 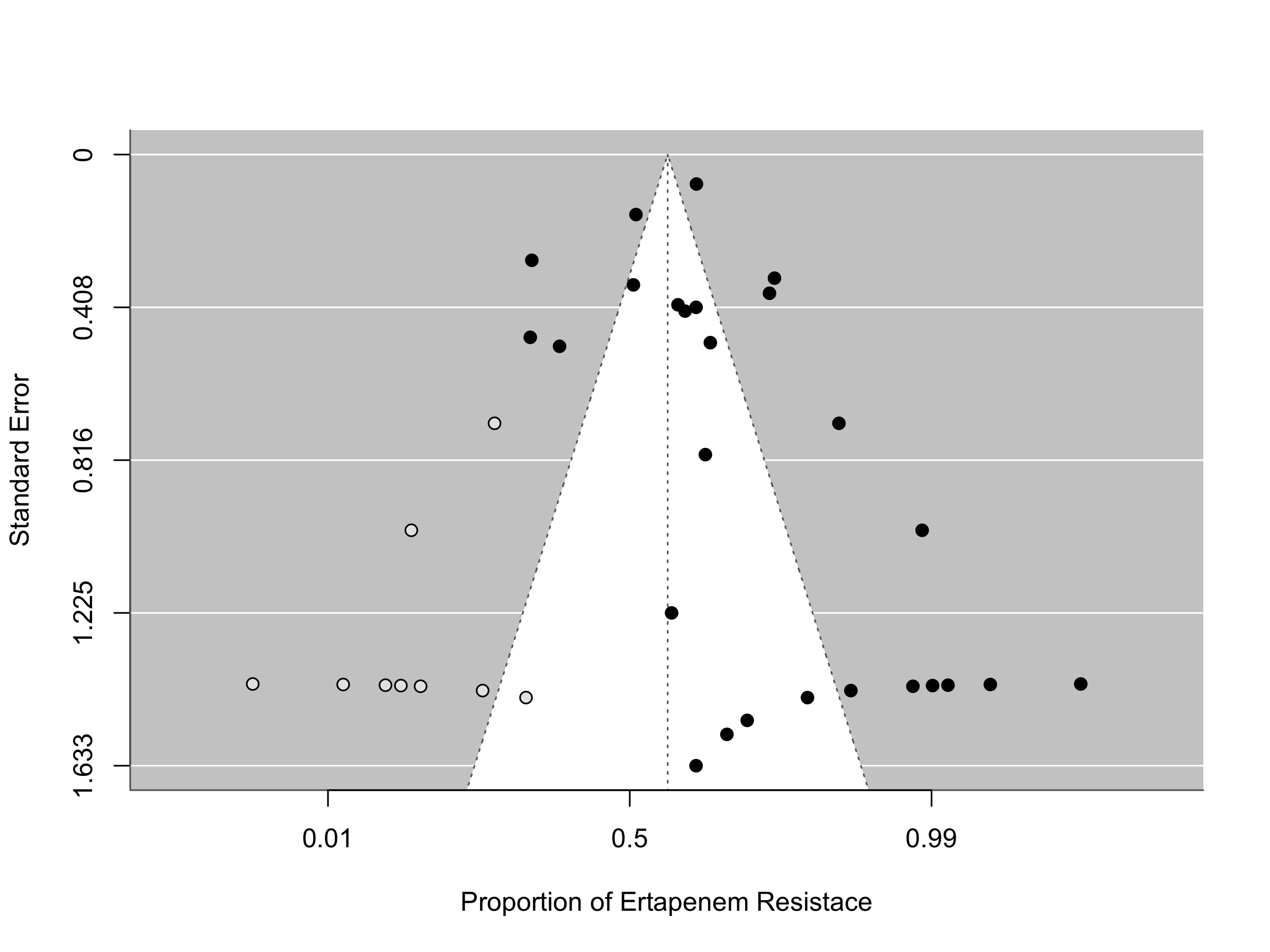  **D** |
| 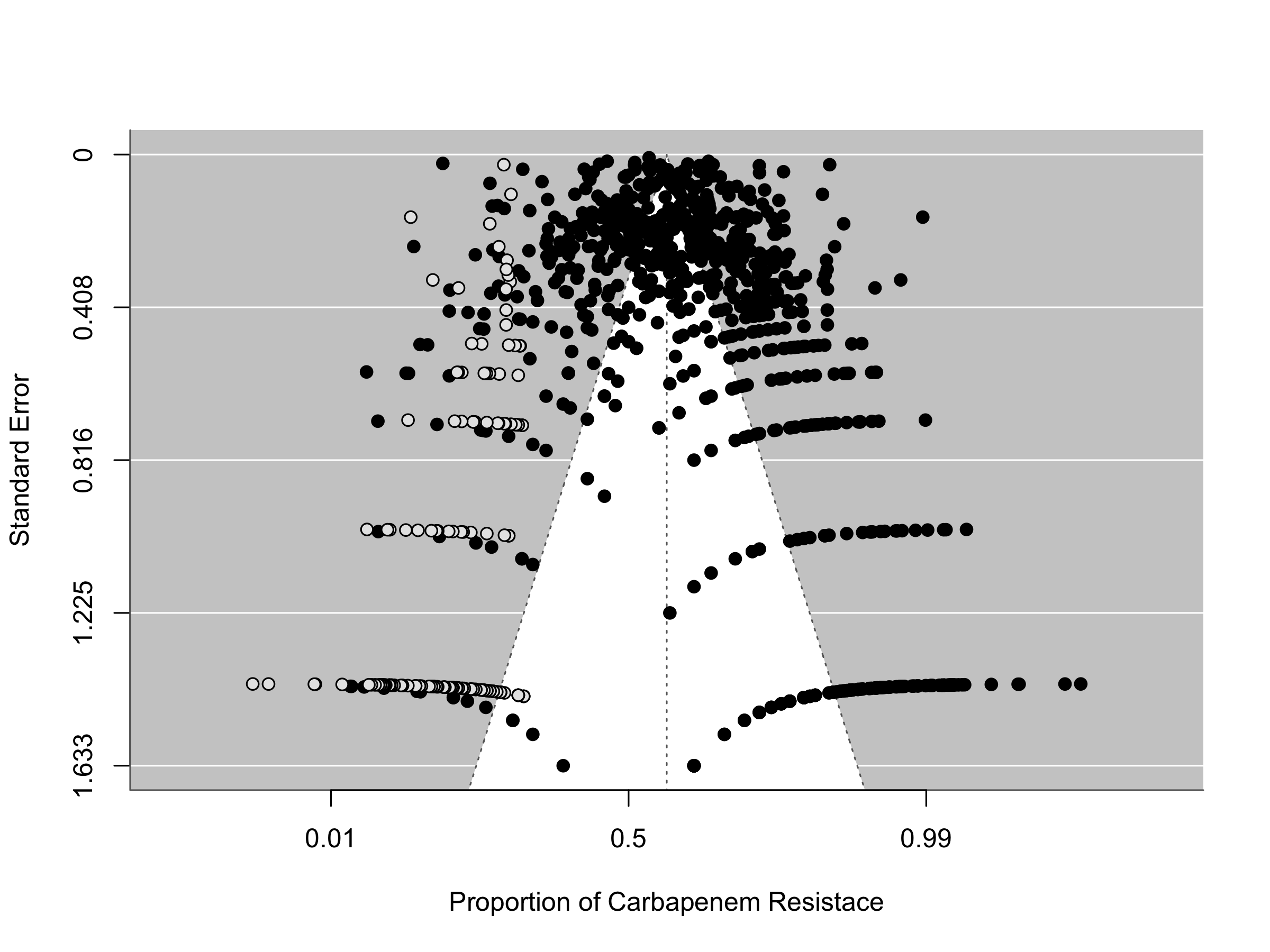  **E** | | |

Supplementary Figure 2: Funnel plots; A: Funnel plot of Imipenem resistance proportion; B: Funnel plot of meropenem resistance proportion; C: Funnel plot of doripenem resistance proportion D: Funnel plot of ertapenem resistance proportion; E: Funnel plot of carbapenem resistance proportion.

# References:

1. Yungyuen T, Chatsuwan T, Plongla R, Kanthawong S, Yordpratum U, Voravuthikunchai SP, et al. Nationwide Surveillance and Molecular Characterization of Critically Drug-Resistant Gram-Negative Bacteria: Results of the Research University Network Thailand Study. Antimicrobial Agents and Chemotherapy. 2021;65(9).

2. Hu FP, Guo Y, Zhu DM, Wang F, Jiang XF, Xu YC, et al. Resistance trends among clinical isolates in China reported from CHINET surveillance of bacterial resistance, 2005-2014. Clinical Microbiology and Infection. 2016;22:S9-S14.

3. Liu Y, Qian Y, Huang XC, Wan YX, Ma W, Li YZ, et al. Distribution and drug susceptibility of pathogens causing nosocomial infection in organ transplant patients. Academic Journal of Second Military Medical University. 2018;39(7):753-7.

4. Dafopoulou K, Tsakris A, Pournaras S. Changes in antimicrobial resistance of clinical isolates of acinetobacter baumannii group isolated in Greece, 2010–2015. Journal of Medical Microbiology. 2018;67(4):496-8.

5. Lee C-M, Lai C-C, Wang Y-Y, Lee M-C, Hsueh P-R. Impact of susceptibility profiles of Gram-negative bacteria before and after the introduction of ertapenem at a medical center in northern Taiwan from 2004 to 2010. Diagnostic Microbiology and Infectious Disease. 2013;75(1):94-100.

6. Li Y, Cao X, Ge H, Jiang Y, Zhou H, Zheng W. Targeted surveillance of nosocomial infection in intensive care units of 176 hospitals in Jiangsu province, China. Journal of Hospital Infection. 2018;99(1):36-41.

7. Guclu AU, Kocak AA, Ok MA, Tutluoglu B, Basustaoglu A, Resp Study G. Antibacterial Resistance in Lower Respiratory Tract Bacterial Pathogens: A Multicenter Analysis from Turkey. Journal of Infection in Developing Countries. 2021;15(2):254-62.

8. Ramsamy Y, ree, Essack SY, Sartorius B, Patel M, Mlisana KP. Antibiotic resistance trends of ESKAPE pathogens in Kwazulu-Natal, South Africa: A five-year retrospective analysis. African journal of laboratory medicine. 2018;7(2):887-.

9. Hoban DJ, Reinert RR, Bouchillon SK, Dowzicky MJ. Global in vitro activity of tigecycline and comparator agents: Tigecycline Evaluation and Surveillance Trial 2004-2013. Annals of Clinical Microbiology and Antimicrobials. 2015;14.

10. Tang X, Xiao M, Zhuo C, Xu Y, Zhong N. Multi-level analysis of bacteria isolated from inpatients in respiratory departments in China. Journal of Thoracic Disease. 2018;10(5):2666-+.

11. Ismail H, Lowman W, Govind CN, Swe-Han KS, Maloba MRB, Bamford C, Perovic O. Surveillance and comparison of antimicrobial susceptibility patterns of ESKAPE organisms isolated from patients with bacteraemia in South Africa, 2016-2017. Samj South African Medical Journal. 2019;109(12):934-40.

12. Zhong M, Long S-s, Huang X-n, Yang Y-c, Yin L, Zhang K, Yu H. Analysis on the distribution and drug resistance of bacteria of sterile body fluids in Sichuan region from 2015 to 2018. Zhongguo Kangshengsu Zazhi. 2019;44(10):1181-8.

13. Liu Y, Wang W, Guo M, Xu Z, Yang Y, Yu L, et al. The Analysis of Drug-Resistant Bacteria from Different Regions of Anhui in 2021. Infection and Drug Resistance. 2022;15:7537-53.

14. Kumari M, Batra P, Malhotra R, Mathur P. A 5-year surveillance on antimicrobial resistance of Acinetobacter isolates at a level-I trauma centre of India. Journal of laboratory physicians. 2019;11(1):34-8.

15. Pogue JM, Zhou Y, Kanakamedala H, Cai B. Burden of illness in carbapenem-resistant Acinetobacter baumannii infections in US hospitals between 2014 and 2019. Bmc Infectious Diseases. 2022;22(1).

16. Tien Viet Dung V, Choisy M, Thi Thuy Nga D, Van Minh Hoang N, Campbell JI, Thi Hoi L, et al. Antimicrobial susceptibility testing results from 13 hospitals in Viet Nam: VINARES 2016-2017. Antimicrobial Resistance and Infection Control. 2021;10(1).

17. Karlowsky JA, Hackel MA, Takemura M, Yamano Y, Echols R, Sahm DF. In Vitro Susceptibility of Gram-Negative Pathogens to Cefiderocol in Five Consecutive Annual Multinational SIDERO-WT Surveillance Studies, 2014 to 2019. Antimicrobial Agents and Chemotherapy. 2022;66(2).

18. Karlowsky JA, Hackel MA, McLeod SM, Miller AA. In Vitro Activity of Sulbactam-Durlobactam against Global Isolates of Acinetobacter baumannii-calcoaceticus Complex Collected from 2016 to 2021. Antimicrobial Agents and Chemotherapy. 2022;66(9).

19. Morfin-Otero R, Dowzicky MJ. Changes in MIC Within a Global Collection of Acinetobacter baumannii Collected as Part of the Tigecycline Evaluation and Surveillance Trial, 2004 to 2009. Clinical Therapeutics. 2012;34(1):101-12.

20. Seifert H, Blondeau J, Lucassen K, Utt EA. Global update on the in vitro activity of tigecycline and comparators against isolates of Acinetobacter baumannii and rates of resistant phenotypes (2016-2018). Journal of Global Antimicrobial Resistance. 2022;31:82-9.

21. Xu L, Zeng Z, Ding Y, Song M, Yang K, Liu J. Distribution and Antimicrobial Susceptibility of Gram-Positive and Gram-Negative Pathogens Isolated from Patients Hospitalized in a Tertiary Teaching Hospital in Southwestern China. Jundishapur Journal of Microbiology. 2020;13(12).

22. Xu T, Xia W, Rong G, Pan S, Huang P, Gu B. A 4-year surveillance of antimicrobial resistance patterns of Acinetobacter baumanni in a university-affiliated hospital in China. Journal of Thoracic Disease. 2013;5(4):506-12.

23. Morfin-Otero R, Alcantar-Curiel MD, Rocha MJ, Alpuche-Ar, a CM, Santos-Preciado JI, et al. Acinetobacter baumannii Infections in a Tertiary Care Hospital in Mexico over the Past 13 Years. Chemotherapy. 2013;59(1):57-65.

24. Lee Y-L, Ko W-C, Hsueh P-R. Geographic patterns of Acinetobacter baumannii and carbapenem resistance in the Asia-Pacific Region: results from the Antimicrobial Testing Leadership and Surveillance (ATLAS) program, 2012-2019. International journal of infectious diseases : IJID : official publication of the International Society for Infectious Diseases. 2023;127:48-55.

25. Said D, Willrich N, Ayobami O, Noll I, Eckmanns T, Markwart R. The epidemiology of carbapenem resistance in Acinetobacter baumannii complex in Germany (2014-2018): an analysis of data from the national Antimicrobial Resistance Surveillance system. Antimicrobial Resistance and Infection Control. 2021;10(1).

26. Seifert H, Stefanik D, Sutcliffe JA, Higgins PG. In-vitro activity of the novel fluorocycline eravacycline against carbapenem non-susceptible Acinetobacter baumannii. International Journal of Antimicrobial Agents. 2018;51(1):62-4.

27. Carvalho Hessel Dias VMd, da Silva DMW, Burger M, de Oliveira AAS, Capelo PdJ, Rocha Specian FAd, et al. Active surveillance of carbapenem-resistant Gram-negative healthcare-associated infections in a low-middle-income country city. Brazilian Journal of Infectious Diseases. 2021;25(2).

28. Rossolini GM, Bochenska M, Fumagalli L, Dowzicky M. Trends of major antimicrobial resistance phenotypes in enterobacterales and gram-negative non-fermenters from ATLAS and EARS-net surveillance systems: Italian vs. European and global data, 2008-2018. Diagnostic Microbiology and Infectious Disease. 2021;101(4).

29. Akcay SS, Inan A, Cevan S, Ozaydin AN, Cobanoglu N, Ozyurek SC, Aksaray S. Gram-negative bacilli causing infections in an intensive care unit of a tertiary care hospital in Istanbul, Turkey. Journal of Infection in Developing Countries. 2014;8(5):597-604.

30. Sannathimmappa MB, Nambiar V, Aravindakshan R. Antibiotic Resistance Pattern of Acinetobacter baumannii Strains: A Retrospective Study from Oman. Saudi journal of medicine & medical sciences. 2021;9(3):254-60.

31. Ruan Z, Chen Y, Jiang Y, Zhou H, Zhou Z, Fu Y, et al. Wide distribution of CC92 carbapenem-resistant and OXA-23-producing Acinetobacter baumannii in multiple provinces of China. International Journal of Antimicrobial Agents. 2013;42(4):322-8.

32. Zhong S, He S. Distribution and Carbapenem Susceptibility of Gram-Negative ESKAPE Pathogens in Hospitalized Patients from Three General Hospitals. Clinical Laboratory. 2021;67(2):361-9.

33. Khursheed N, Qurat ul A, Ali MM, Adnan F. ANTIMICROBIAL SUSCEPTIBILITY PATTERN AND EMERGING ANTIBIOGRAM TREND OF ACINETOBACTER BAUMANNII ISOLATES FROM A TERTIARY CARE HOSPITAL. International Journal of Biology and Biotechnology. 2021;18(4):681-6.

34. Duran H, Çeken N, Kula Atik T. Bacteria isolated from endotracheal aspirate samples and antibiotic resistance rates: 5-year analysis: 5-year retrospective analysis. Turkiye Klinikleri Journal of Medical Sciences. 2021;41(3):327-34.

35. Chen Y, Ji J, Ying C, Liu Z, Yang Q, Kong H, et al. Blood bacterial resistant investigation collaborative system (BRICS) report: a national surveillance in China from 2014 to 2019. Antimicrobial Resistance and Infection Control. 2022;11(1).

36. Santajit S, Bhoopong P, Kong-Ngoen T, Tunyong W, Horpet D, Paehoh-ele W, et al. Phenotypic and Genotypic Investigation of Carbapenem-Resistant Acinetobacter baumannii in Maharaj Nakhon Si Thammarat Hospital, Thailand. Antibiotics-Basel. 2023;12(3).

37. Rodriguez-Lucas C, Rosario Rodicio M, Vazquez X, Escudero D, Quindos B, Alaguero M, et al. Extensively drug-resistantAcinetobacter baumanniicarryingbla(OXA-23-like)andarmAin a hospital after an intervention in the intensive care unit which ended a long-standing endemicity. European Journal of Clinical Microbiology & Infectious Diseases. 2021;40(2):385-9.

38. Zhang H, Zhang G, Zhang J, Duan S, Kang Y, Yang Q, Xu Y. Antimicrobial Activity of Colistin Against Contemporary (2015-2017)P. aeruginosaandA. baumanniiIsolates From a Chinese Surveillance Program. Frontiers in Microbiology. 2020;11.

39. Castanheira M, Jones RN, Livermore DM. Antimicrobial activities of doripenem and other carbapenems against Pseudomonas aeruginosa, other nonfermentative bacilli, and Aeromonas spp. Diagnostic Microbiology and Infectious Disease. 2009;63(4):426-33.

40. Zhang Z, Sun Z, Tian L. Antimicrobial Resistance Among Pathogens Causing Bloodstream Infections: A Multicenter Surveillance Report Over 20 Years (1998–2017). Infection and Drug Resistance. 2022;15:249-60.

41. McLeod SM, Moussa SH, Hackel MA, Miller AA. In Vitro Activity of Sulbactam-Durlobactam against Acinetobacter baumannii-calcoaceticus Complex Isolates Collected Globally in 2016 and 2017. Antimicrobial Agents and Chemotherapy. 2020;64(4).

42. Biedenbach DJ, Phan Trong G, Pham Hung V, Nguyen Su Minh T, Tran Thi Thanh N, Doan Mai P, et al. Antimicrobial-resistant Pseudomonas aeruginosa and Acinetobacter baumannii From Patients With Hospital-acquired or Ventilator-associated Pneumonia in Vietnam. Clinical Therapeutics. 2016;38(9):2098-105.

43. Moise PA, Gonzalez M, Alekseeva I, Lopez D, Akrich B, DeRyke CA, et al. Collective assessment of antimicrobial susceptibility among the most common Gram-negative respiratory pathogens driving therapy in the ICU. JAC-Antimicrobial Resistance. 2021;3(1):dlaa129-Article No.: dlaa.

44. Azimi L, Fallah F, Karimi A, Shirvani F, Tehrani NA, Armin S, et al. Prevalence and Antimicrobial Resistance Patterns in ESKAPE Pathogens in Iran. Archives of Pediatric Infectious Diseases. 2023;11(1).

45. man D, Bratu S, Kochar S, Panwar M, Trehan M, Doymaz M, Quale J. Evolution of antimicrobial resistance among Pseudomonas aeruginosa, Acinetobacter baumannii and Klebsiella pneumoniae in Brooklyn, NY. J Antimicrob Chemother. 2007;60(1):78-82.

46. Yang Q, Xu Y, Jia P, Zhu Y, Zhang J, Zhang G, et al. In vitro activity of sulbactam/durlobactam against clinical isolates of Acinetobacter baumannii collected in China. Journal of Antimicrobial Chemotherapy. 2020;75(7):1833-9.

47. Humphries RM, Janssen H, Hey-Hadavi JH, Hackel M, Sahm D. Multidrug-resistant Gram-negative bacilli recovered from respiratory and blood specimens from adults: the ATLAS surveillance program in European hospitals, 2018-2020. International Journal of Antimicrobial Agents. 2023;61(2).

48. Vijayakumar S, An, an S, Prabaa MSD, Kanthan K, Vijayabaskar S, et al. Insertion sequences and sequence types profile of clinical isolates of carbapenem-resistant A. baumannii collected across India over four year period. Journal of Infection and Public Health. 2020;13(7):1022-8.

49. Mashau RC, Meiring ST, Dramowski A, Magobo RE, Quan VC, Perovic O, et al. Culture-confirmed neonatal bloodstream infections and meningitis in South Africa, 2014–19: a cross-sectional study. The Lancet Global Health. 2022;10(8):e1170-e8.

50. Tuzemen NU, Payaslioglu M, Ozakin C, Ener B, Akalin H. Trends of Bloodstream Infections in a University Hospital During 12 Years. Polish Journal of Microbiology. 2022;71(3):443-52.

51. Sedaghat A, Khadem-Rezaiyan M, Ahmadabadi A, Abbaspour H, Youssefi M, Shirzad MM, et al. Antibacterial Resistance Pattern of Acinetobacter baumannii in Burn Patients in Northeast of Iran. Jundishapur Journal of Microbiology. 2019;12(10).

52. Liu C, Xu M, Li X, Dong H, Ming L. Trends in antimicrobial resistance in bloodstream infections at a large tertiary-care hospital in China: a 10-year retrospective study (2010-2019). Journal of Global Antimicrobial Resistance. 2022;29:413-9.

53. Chen J, Li F, Tian S, Sun G, Chu Y. Surveillance of bacterial resistance in The First Hospital of China Medical University in 2019. Journal of China Medical University. 2021;50(3):245-9,53.

54. Xu Y-m, Chen R-c, Du Y, Wang Y-j, Guo X, Shan B. Bacterial resistance surveillance in Yunnan province in 2014. Zhongguo Kangshengsu Zazhi. 2016;41(9):684-9.

55. Marco F, Dowzicky MJ. Antimicrobial susceptibility among important pathogens collected as part of the Tigecycline Evaluation and Surveillance Trial (TEST) in Spain, 2004-2014. Journal of Global Antimicrobial Resistance. 2016;6:50-6.

56. Maraki S, Mantadakis E, Mavromanolaki VE, Kofteridis DP, Samonis G. A 5-year Surveillance Study on Antimicrobial Resistance of Acinetobacter baumannii Clinical Isolates from a Tertiary Greek Hospital. Infection & chemotherapy. 2016;48(3):190-8.

57. Hu X-g, Yu G-c, Liu J-z. Analysis of the antimicrobial resistance of Acinetobacter baumannii isolated from 2010 to 2016. Zhongguo Kangshengsu Zazhi. 2017;42(7):592-5.

58. Wang Q, Wang Z, Zhang F, Zhao C, Yang B, Sun Z, et al. Long-Term Continuous Antimicrobial Resistance Surveillance Among Nosocomial Gram-Negative Bacilli in China from 2010 to 2018 (CMSS). Infection and Drug Resistance. 2020;13:2617-29.

59. Anggraini D, Santosaningsih D, Saharman YR, Endraswari PD, Cahyarini C, Saptawati L, et al. Distribution of Carbapenemase Genes among Carbapenem-Non-Susceptible Acinetobacter baumanii Blood Isolates in Indonesia: A Multicenter Study. Antibiotics-Basel. 2022;11(3).

60. Chen C-H, Wu P-H, Lu M-C, Ho M-W, Hsueh P-R. Geographic patterns of carbapenem-resistant, multi-drug-resistant and difficult-to-treat Acinetobacter baumannii in the Asia-Pacific region: results from the Antimicrobial Testing Leadership and Surveillance (ATLAS) program, 2020. International journal of antimicrobial agents. 2023;61(2):106707-.

61. Nadia J, Wejdene M, Bonnin RA, Meriam G, Cherifa C, Rachida G, et al. Temporal variation in antibiotic resistance of acinetobacter baumannii in a teaching hospital in Tunisia: Correlation with antimicrobial consumption. Open Microbiology Journal. 2019;13(1):106-11.

62. Tian J, Zhang G, Ju Y, Tang N, Li J, Jia R, Feng J. Five novel carbapenem-hydrolysing OXA-type beta-lactamase groups are intrinsic in Acinetobacter spp. Journal of Antimicrobial Chemotherapy. 2018;73(12):3279-84.

63. Rodrigues Perez L, Reus r, Carniel E, Dalpiaz G, Vetter M, Narvaez GA, Dias CG. A four-year follow-up survey of antimicrobial resistance among Acinetobacter baumannii complex from inpatients in Southern Brazil. American Journal of Infection Control. 2021;49(12):1503-5.

64. Aşik G, Özdemir M, Kurtoğlu MG, Yağci S, Öksüz L, Gül M, et al. Detection of the frequency of PER-1 type extended-spectrum β-lactamase-producing Acinetobacter baumannii clinical isolates in Turkey: a multicenter study. Turk J Med Sci. 2014;44(6):1041-6.

65. Kim D, Yoon EJ, Hong JS, Choi MH, Kim HS, Kim YR, et al. Major Bloodstream Infection-Causing Bacterial Pathogens and Their Antimicrobial Resistance in South Korea, 2017-2019: Phase I Report From Kor-GLASS. Front Microbiol. 2021;12:799084.

66. Caskurlu H, Davarci I, Kocoglu ME, Cag Y. Examination of Blood and Tracheal Aspirate Culture Results in Intensive Care Patients: 5-year analysis. Medeniyet medical journal. 2020;35(2):128-35.

67. Balode A, Punda-Polic V, Dowzicky MJ. Antimicrobial susceptibility of Gram-negative and Gram-positive bacteria collected from countries in Eastern Europe: results from the Tigecycline Evaluation and Surveillance Trial (TEST) 2004-2010. International Journal of Antimicrobial Agents. 2013;41(6):527-35.

68. Mao T, Zhai H, Duan G, Yang H. Patterns of Drug-Resistant Bacteria in a General Hospital, China, 2011-2016. Polish Journal of Microbiology. 2019;68(2):225-32.

69. Kuo S-C, Liu C-E, Lu P-L, Chen Y-S, Lu M-C, Ko W-C, et al. Activity of ceftolozane-tazobactam against Gram-negative pathogens isolated from lower respiratory tract infections in the Asia-Pacific region: SMART 2015-2016. International Journal of Antimicrobial Agents. 2020;55(3).

70. Sun Y, Li M, Chen L, Chen H, Yu X, Ye J, et al. Prevalence and molecular characterization of carbapenemase-producing gram-negative bacteria from a university hospital in China. Infectious Diseases. 2016;48(2):138-46.

71. Zhang Z, Chen M, Yu Y, Pan S, Liu Y. Antimicrobial susceptibility among gram-positive and gram-negative blood-borne pathogens collected between 2012-2016 as part of the Tigecycline Evaluation and Surveillance Trial. Antimicrob Resist Infect Control. 2018;7:152.

72. Direkel Ş, Uzunoʇlu E, Keleş S, Yapar K. Antibiotic resistance rates of Acinetobacter Baumannii strains isolated from various clinical samples in Giresun Prof. Dr. Atilla Ilhan Ozdemir State Hospital. Gazi Medical Journal. 2015;26(3):92-6.

73. Guzek A, Korzeniewski K, Tomaszewski D, Rybicki Z, Zwolińska E. Bacteriological assessment of pneumonia caused by gram-negative bacteria in patients hospitalized in intensive care unit. 2017. p. 39-46.

74. Al-Tamimi M, Albalawi H, Alkhawaldeh M, Alazzam A, Ramadan H, Altalalwah M, et al. Multidrug-Resistant Acinetobacter baumannii in Jordan. Microorganisms. 2022;10(5).

75. Yang S, Xu H, Sun J, Sun S. Shifting trends and age distribution of ESKAPEEc resistance in bloodstream infection, Southwest China, 2012-2017. Antimicrobial Resistance and Infection Control. 2019;8.

76. Riccobono E, Bogaerts P, Antonelli A, Evrard S, Giani T, Rossolini GM, Glupczynski Y. Evaluation of the OXA-23 K-SeT (R) immunochromatographic assay for the rapid detection of OXA-23-like carbapenemase-producing Acinetobacter spp. Journal of Antimicrobial Chemotherapy. 2019;74(5):1455-7.

77. Sader HS, Streit JM, Carvalhaes CG, Hub, MD, Shortridge D, et al. Frequency of occurrence and antimicrobial susceptibility of bacteria isolated from respiratory samples of patients hospitalized with pneumonia in Western Europe, Eastern Europe and the USA: results from the SENTRY Antimicrobial Surveillance Program (2016-19). JAC-Antimicrobial Resistance. 2021;3(3):dlab117-Article No.: dlab.

78. Yang Y, Guo Y, Yin D, Zheng Y, Wu S, Zhu D, Hu F. In Vitro Activity of Cefepime-Zidebactam, Ceftazidime-Avibactam, and Other Comparators against Clinical Isolates of Enterobacterales, Pseudomonas aeruginosa, and Acinetobacter baumannii: Results from China Antimicrobial Surveillance Network (CHINET) in 2018. Antimicrob Agents Chemother. 2020;65(1).

79. Li Y, Lv Y, Xue F, Zheng B, Liu J, Zhang J. Antimicrobial resistance surveillance of doripenem in China. Journal of Antibiotics. 2015;68(8):496-500.

80. Musyoki VM, Masika MM, Mutai W, Wilfred G, Kuria A, Muthini F. Antimicrobial susceptibility pattern of Acinetobacter isolates from patients in Kenyatta National Hospital, Nairobi, Kenya. The Pan African medical journal. 2019;33:146-.

81. Jaidane N, Naas T, Oueslati S, Bernabeu S, rine, Boujaafar N, et al. Whole-genome sequencing of NDM-1-producing ST85 Acinetobacter baumannii isolates from Tunisia. International Journal of Antimicrobial Agents. 2018;52(6):916-21.

82. Han R, Ding L, Yang Y, Guo Y, Yin D, an, et al. In Vitro Activity of KBP-7072 against 536 Acinetobacter baumannii Complex Isolates Collected in China. Microbiology Spectrum. 2022;10(1).

83. Flores-Paredes W, Luque N, Albornoz R, Rojas N, Espinoza M, Pons MJ, Ruiz J. Evolution of Antimicrobial Resistance Levels of ESKAPE Microorganisms in a Peruvian IV-Level Hospital. Infection & chemotherapy. 2021;53(3):449-62.

84. Kang S, Jeong IS. Epidemiological characteristics of carbapenem-resistant Enterobacteriaceae and carbapenem-resistant Acinetobacter baumannii in a tertiary referral hospital in Korea. Osong public health and research perspectives. 2022;13(3):221-9.

85. Della Rocca MT, Panetta V, Durante A, Bucci L, Matano A, Annecchiarico A, Greco R. Pathogens distribution and antimicrobial resistance pattern of blood stream infections in Southern Italian hospital, 2016-2021 surveillance. The new microbiologica. 2023;46(1):29-36.

86. Aloraifi RI, Alharthi AF, Almefleh AA, Alamri AH, Alobud AS, Bawazeer RA, et al. Prevalence of Carbapenem Non-susceptible Gram-Negative Bacteria at Tertiary Care Hospitals in Saudi Arabia. Cureus. 2023;15(1):e33767-e.

87. Jumroon N, Santanir, P. Multiple combination patterns of OXA-type carbapenemhydrolyzing and metallo-β-lactamases encoding genes among clinically isolated Acinetobacter Baumannii. International Journal of Pharma and Bio Sciences. 2013;4(2):B908-B17.

88. Nafplioti K, Galani I, Angelidis E, Adamou P, Moraitou E, Giannopoulou P, et al. Dissemination of International Clone II Acinetobacter baumannii Strains Coproducing OXA-23 Carbapenemase and 16S rRNA Methylase ArmA in Athens, Greece. Microbial Drug Resistance. 2020;26(1):9-13.

89. Davies TA, Queenan AM, Morrow BJ, Shang W, Amsler K, He W, et al. Longitudinal survey of carbapenem resistance and resistance mechanisms in Enterobacteriaceae and non-fermenters from the USA in 2007-09. Journal of Antimicrobial Chemotherapy. 2011;66(10):2298-307.

90. Mhondoro M, Ndlovu N, Donewell B, Juru T, Tafara GN, Gerald S, et al. Trends in antimicrobial resistance of bacterial pathogens in Harare, Zimbabwe, 2012-2017: a secondary dataset analysis. Bmc Infectious Diseases. 2019;19(1).

91. Chaudhary M, Payasi A. Incidence, prevalence and control of multidrug resistant (MDR) carbapenemase producing Acinetobacter baumanii in Indian intensive care units. Journal of Pharmacy Research. 2013;7(2):175-80.

92. Houngsaitong J, Montakantikul P, Paiboonwong T, Chomnawang M, Khuntayaporn P, Chulavatnatol S. In vitro activity of biapenem and comparators against multidrug-resistant and carbapenem-resistant Acinetobacter baumannii isolated from tertiary care hospitals in Thailand. Pharmaceutical Sciences Asia. 2020;47(4):378-86.

93. Garza-Gonzalez E, Martin Llaca-Diaz J, Javier Bosques-Padilla F, Gonzalez GM. Prevalence of Multidrug-Resistant Bacteria at a Tertiary-Care Teaching Hospital in Mexico: Special Focus on Acinetobacter baumannii. Chemotherapy. 2010;56(4):275-9.

94. Yoon E-J, Kim JO, Yang JW, Kim HS, Lee KJ, Jeong SH, et al. The bla(OXA-23)-associated transposons in the genome of Acinetobacter spp. represent an epidemiological situation of the species encountering carbapenems. Journal of Antimicrobial Chemotherapy. 2017;72(10):2708-14.

95. Sharma S, Banerjee T, Yadav G, Kumar A. Susceptibility profile of bla (OXA-23) and metallo-β-lactamases co-harbouring isolates of carbapenem resistant Acinetobacter baumannii (CRAB) against standard drugs and combinations. Front Cell Infect Microbiol. 2022;12:1068840.

96. Sangale A, Vivek B, Kelkar R, Biswas S. Microbiology of Ventilator-associated Pneumonia in a Tertiary Care Cancer Hospital. Indian journal of critical care medicine : peer-reviewed, official publication of Indian Society of Critical Care Medicine. 2021;25(4):421-8.

97. Fu Q, Zheng S. The distribution and antimicrobial resistance of common bacteria of nosocomial infection. Life Science Journal. 2013;10(3):1658-61.

98. Jiang M, Chen X, Liu S, Zhang Z, Li N, Dong C, et al. Epidemiological Analysis of Multidrug-Resistant Acinetobacter baumannii Isolates in a Tertiary Hospital Over a 12-Year Period in China. Frontiers in Public Health. 2021;9.

99. Velasco JM, Valderama T, Margulieux K, Diones PC, Peacock T, Navarro FC, et al. Comparison of Carbapenem-Resistant Microbial Pathogens in Combat and Non-combat Wounds of Military and Civilian Patients Seen at a Tertiary Military Hospital, Philippines (2013-2017). Military Medicine. 2020;185(1):E197-E202.

100. Sana F, Hussain A, Hussain W, Zaman G, Abbas MW, Imtiaz A, Satti L. Frequency And Clinical Spectrum Of Multidrug Resistant Acinetobacter Baumannii As A Significant Nosocomial Pathogen In Intensive Care Unit Patients. Journal of Ayub Medical College, Abbottabad : JAMC. 2021;33(4):S752-S6.

101. Kashkouri N, Tabarsi P, Toutkaboni MP, Dizaji MK, Bahrami N, Narimani A, et al. The Prevalence of Carbapenemase Genes in Carbapenem-resistant Gram-negative Bacilli, Masih Daneshvari Hospital, Tehran, Iran, 2019-2020. Iranian Journal of Medical Microbiology. 2022;16(6):573-80.

102. Han Y, Zhang J, Zhang H-Z, Zhang X-Y, Wang Y-M. Multidrug-resistant organisms in intensive care units and logistic analysis of risk factors. World Journal of Clinical Cases. 2022;10(6):1795-805.

103. Paiboonvong T, Rodjun V, Houngsaitong J, Chomnawang M, Montakantikul P, Chulavatnatol S. Comparative in vitro activity of sitafloxacin against multidrug-resistant and carbapenem-resistant acinetobacter baumannii clinical isolates in Thailand. Pharmaceutical Sciences Asia. 2020;47(1):37-42.

104. Eslami M, Shafiei M, Mirforughi SA, Rajabi A. Multiple carbapenemase gene production by Acinetobacter baumannii isolates from burn patients in Iran. Reviews and Research in Medical Microbiology. 2019;30(2):90-4.

105. Meybodi MME, Foroushani AR, Zolfaghari M, Abdollahi A, Alipour A, Mohammadnejad E, et al. Antimicrobial resistance pattern in healthcare-associated infections: investigation of in-hospital risk factors. Iranian journal of microbiology. 2021;13(2):178-82.

106. Wang L, Chen Y, Han R, Huang Z, Zhang X, Hu F, Yang F. Sulbactam Enhances in vitro Activity of beta-Lactam Antibiotics Against Acinetobacter baumannii. Infection and Drug Resistance. 2021;14:3971-7.

107. Khaled JM, Alharbi NS, Siddiqi MZ, Alobaidi AS, Nauman K, Alahmedi S, et al. A synergic action of colistin, imipenem, and silver nanoparticles against pandrug-resistant Acinetobacter baumannii isolated from patients. Journal of Infection and Public Health. 2021;14(11):1679-85.

108. Rodjun V, Houngsaitong J, Montakantikul P, Paiboonvong T, Khuntayaporn P, Yanyongchaikit P, Sriyant P. In Vitro Activities of Colistin and Sitafloxacin Combinations against Multidrug-, Carbapenem-, and Colistin-ResistantAcinetobacter baumanniiUsing the Broth Microdilution Checkerboard and Time-Kill Methods. Antibiotics-Basel. 2020;9(8).

109. Dolores Alcantar-Curiel M, Francisco Garcia-Torres L, Ines Gonzalez-Chavez M, Morfin-Otero R, Gayosso-Vazquez C, Dolores Jarillo-Quijada M, et al. Molecular Mechanisms Associated with Nosocomial Carbapenem-resistant Acinetobacter baumannii in Mexico. Archives of Medical Research. 2014;45(7):553-60.

110. Sader HS, Mendes RE, Streit JM, Carvalhaes CG, Castanheira M. Antimicrobial susceptibility of Gram-negative bacteria from intensive care unit and non-intensive care unit patients from United States hospitals (2018-2020). Diagnostic Microbiology and Infectious Disease. 2022;102(1).

111. Khoshbakht R, Kabiri M, Neshani A, Khaksari MN, Sadrzadeh SM, Mousavi SM, et al. Assessment of antibiotic resistance changes during the Covid-19 pandemic in northeast of Iran during 2020–2022: an epidemiological study. Antimicrobial Resistance and Infection Control. 2022;11(1).

112. Yi H, Huang J, Guo L, Zhang Q, Qu J, Zhou M. Increased Antimicrobial Resistance among Sputum Pathogens from Patients with Hyperglycemia. Infection and Drug Resistance. 2020;13:1723-33.

113. Principe L, Piazza A, Giani T, Bracco S, Caltagirone MS, Arena F, et al. Epidemic Diffusion of OXA-23-Producing Acinetobacter baumannii Isolates in Italy: Results of the First Cross-Sectional Countrywide Survey. Journal of Clinical Microbiology. 2014;52(8):3004-10.

114. Samonis G, Maraki S, Vouloumanou EK, Georgantzi GG, Kofteridis DP, Falagas ME. Antimicrobial susceptibility of non-fermenting Gram-negative isolates to isepamicin in a region with high antibiotic resistance. European Journal of Clinical Microbiology & Infectious Diseases. 2012;31(11):3191-8.

115. Spiliopoulou A, Jelastopulu E, Vamvakopoulou S, Bartzavali C, Kolonitsiou F, Anastassiou ED, Christofidou M. In vitro activity of tigecycline and colistin against A. baumannii clinical bloodstream isolates during an 8-year period. Journal of Chemotherapy. 2015;27(5):266-70.

116. Mirzaei B, Bazgir ZN, Goli HR, Iranpour F, Mohammadi F, Babaei R. Prevalence of multi-drug resistant (MDR) and extensively drug-resistant (XDR) phenotypes of Pseudomonas aeruginosa and Acinetobacter baumannii isolated in clinical samples from Northeast of Iran. BMC research notes. 2020;13(1):380-.

117. Lukovic B, Gajic I, Dimkic I, Kekic D, Zornic S, Pozder T, et al. The first nationwide multicenter study ofAcinetobacter baumanniirecovered in Serbia: emergence of OXA-72, OXA-23 and NDM-1-producing isolates. Antimicrobial Resistance and Infection Control. 2020;9(1).

118. Wang H, Guo P, Sun H, Wang H, Yang Q, Chen M, et al. Molecular epidemiology of clinical isolates of carbapenem-resistant Acinetobacter spp. from chinese Hospitals del. Antimicrobial Agents and Chemotherapy. 2007;51(11):4022-8.

119. ÇIÇek AT, Düzgün AT, Saral A, Kayman T, ÇIzmecI Z, Balci PT, et al. Detection of class 1 integron in Acinetobacter baumannii isolates collected from nine hospitals in Turkey. Asian Pacific Journal of Tropical Biomedicine. 2013;3(9):743-7.

120. Leungtongkam U, Thummeepak R, Wongprachan S, Thongsuk P, Kitti T, Ketwong K, et al. Dissemination of bla(OXA-23), bla(OXA-24), bla(OXA-58), and bla(NDM-1) Genes of Acinetobacter baumannii Isolates from Four Tertiary Hospitals in Thailand. Microbial Drug Resistance. 2018;24(1):55-62.

121. Alrahmany D, Omar AF, Harb G, El Nekidy WS, Ghazi IM. Acinetobacter baumannii Infections in Hospitalized Patients, Treatment Outcomes. Antibiotics-Basel. 2021;10(6).

122. Dolores Alcantar-Curiel M, Rosales-Reyes R, Dolores Jarillo-Quijada M, Gayosso-Vazquez C, Fern L, ez-Vazquez J, et al. Carbapenem-Resistant Acinetobacter baumannii in Three Tertiary Care Hospitals in Mexico: Virulence Profiles, Innate Immune Response and Clonal Dissemination. Frontiers in Microbiology. 2019;10.

123. Lopez-Hern, ez I, Delgado-Valverde M, Fern, ez-Cuenca F, Lopez-Cerero L, et al. Carbapenemase-Producing Gram-Negative Bacteria in Andalusia, Spain, 2014-2018. Emerging Infectious Diseases. 2020;26(9):2218-22.

124. Qu J, Feng C, Li H, Lv X. Antibiotic strategies and clinical outcomes for patients with carbapenem-resistant Gram-negative bacterial bloodstream infection. International Journal of Antimicrobial Agents. 2021;57(3).

125. Martin Llaca-Diaz J, Mendoza-Olazaran S, Camacho-Ortiz A, Flores S, Garza-Gonzalez E. One-Year Surveillance of ESKAPE Pathogens in an Intensive Care Unit of Monterrey, Mexico. Chemotherapy. 2012;58(6):475-81.

126. Katoch O, Sharad N, Singh P, Srivastav S, Aggrawal R, Malhotra R, Mathur P. High Prevalence of Fungal and NDM-OXA Producing Gram-Negative Bacterial Superinfections in the Second Wave of Coronavirus Disease 2019 in India: Experience from a Dedicated Coronavirus Disease 2019 Hospital in North India. Journal of global infectious diseases. 2022;14(4):154-61.

127. Kooti S, Motamedifar M, Sarvari J. Antibiotic Resistance Profile and Distribution of Oxacillinase Genes Among Clinical Isolates of Acinetobacter baumannii in Shiraz Teaching Hospitals, 2012-2013. Jundishapur Journal of Microbiology. 2015;8(8).

128. Kareem SM. Emergence of mcr- and fosA3-mediated colistin and fosfomycin resistance among carbapenem-resistant Acinetobacter baumannii in Iraq. Meta Gene. 2020;25.

129. Prasai A, Pant A, Neupane A, Pant S, Pradhan S. Extensive drug resistant acinetobacter species isolates in sputum sample of patient admitted in intensive care unit of a tertiary care centre: A descriptive cross-sectional study. Journal of the Nepal Medical Association. 2021;59(242):996-69.

130. Chitrabanu NA, Mallya S. Identification, Speciation and Antibiogram along with Detection of Metallo Beta-lactamase Production in Acinetobacter Isolated from Clinical Samples in a Tertiary Care Hospital. Journal of Pure and Applied Microbiology. 2021;15(2):839-44.

131. Silveira MC, Rocha-de-Souza CM, de Oliveira Santos IC, Pontes LDS, Oliveira TRTE, Tavares-Teixeira CB, et al. Genetic Basis of Antimicrobial Resistant Gram-Negative Bacteria Isolated From Bloodstream in Brazil. Frontiers in Medicine. 2021;8.

132. Guvenir M, Guler E, Suer K. Do Seasonal Changes and Climate Effect the Prevalence of Antibiotic Resistance of Acinetobacter calcoaceticus-baumannii Complex? Polish Journal of Environmental Studies. 2021;30(2):1155-9.

133. Liu C, Yoon E-J, Kim D, Shin JH, Shin JH, Shin KS, et al. Antimicrobial resistance in South Korea: A report from the Korean global antimicrobial resistance surveillance system (Kor-GLASS) for 2017. Journal of Infection and Chemotherapy. 2019;25(11):845-59.

134. Alamri A, Hamid ME, Abid M, Alwahhabi AM, Alqahtani KM, Alqarni MS, Abomughaid M. Trend analysis of bacterial uropathogens and their susceptibility pattern: A 4-year (2013-2016) study from Aseer region, Saudi Arabia. Urology annals. 2018;10(1):41-6.

135. Hamzeh AR, Al Najjar M, Mahfoud M. Prevalence of antibiotic resistance among Acinetobacter baumannii isolates from Aleppo, Syria. American Journal of Infection Control. 2012;40(8):776-7.

136. Alsultan AA, Evans BA, Elsayed EA, Al-Thawadi SI, Al-Taher AY, Amyes SGB, et al. High frequency of carbapenem-resistant Acinetobacter baumannii in patients with diabetes mellitus in Saudi Arabia. Journal of Medical Microbiology. 2013;62:885-8.

137. Nwabor OF, Terbtothakun P, Voravuthikunchai SP, Chusri S. Evaluation of the Synergistic Antibacterial Effects of Fosfomycin in Combination with Selected Antibiotics against Carbapenem-Resistant Acinetobacter baumannii. Pharmaceuticals. 2021;14(3).

138. Sader HS, Carvalhaes CG, Mendes RE, Castanheira M. Antimicrobial activity of high-dose cefepime-tazobactam (WCK 4282) against a large collection of gram-negative organisms collected worldwide in 2018 and 2019. International Journal of Infectious Diseases. 2022;116:306-12.

139. Bharathi SV, Venkataramaiah M, Rajamohan G. Genotypic and Phenotypic Characterization of Novel Sequence Types of Carbapenem-Resistant Acinetobacter baumannii, With Heterogeneous Resistance Determinants and Targeted Variations in Efflux Operons. Frontiers in Microbiology. 2021;12.

140. Lavrinenko A, Sheck E, Kolesnichenko S, Azizov I, Turmukhambetova A. Antibiotic Resistance and Genotypes of Nosocomial Strains of Acinetobacter baumannii in Kazakhstan. Antibiotics-Basel. 2021;10(4).

141. Ziolkowski G, Pawlowska I, Krawczyk L, Wojkowska-Mach J. Antibiotic consumption versus the prevalence of multidrug-resistant Acinetobacter baumannii and Clostridium difficile infections at an ICU from 2014-2015. Journal of Infection and Public Health. 2018;11(5):626-30.

142. Perovic O, Ismail H, Van Schalkwyk E, Lowman W, Prentice E, Senekal M, Govind CN. Antimicrobial resistance surveillance in the South African private sector report for 2016. Southern African Journal of Infectious Diseases. 2018;33(4):114-7.

143. Khoramrooz SS, Eslami S, Motamedifar M, Bazargani A, Zomorodian K. High Frequency of Class I and II Integrons and the Presence of aadA2 and dfrA12 Gene Cassettes in the Clinical Isolates of Acinetobacter baumannii from Shiraz, Southwest of Iran. Jundishapur Journal of Microbiology. 2021;14(12).

144. Vijay S, Bansal N, Rao BK, Veeraraghavan B, Rodrigues C, Wattal C, et al. Secondary Infections in Hospitalized COVID-19 Patients: Indian Experience. Infection and Drug Resistance. 2021;14:1893-903.

145. Cercenado E, Cardenoso L, Penin R, Longshaw C, Henriksen AS, Pascual A. In vitro activity of cefiderocol and comparators against isolates of Gram-negative bacterial pathogens from a range of infection sources: SIDERO-WT-2014-2018 studies in Spain. Journal of Global Antimicrobial Resistance. 2021;26:292-300.

146. Javaid N, Sultana Q, Rasool K, ra S, Ahmad F, Chaudhary SU, Mirza S. Trends in antimicrobial resistance amongst pathogens isolated from blood and cerebrospinal fluid cultures in Pakistan (2011-2015): A retrospective cross-sectional study. PLoS ONE. 2021;16(4).

147. Meng X, Fu J, Zheng Y, Qin W, Yang H, Cao D, et al. Ten-Year Changes in Bloodstream Infection With Acinetobacter Baumannii Complex in Intensive Care Units in Eastern China: A Retrospective Cohort Study. Frontiers in Medicine. 2021;8.

148. Karlowsky JA, Bouchillon SK, Benaouda A, Soraa N, Zerouali K, Mohamed N, et al. Antimicrobial susceptibility testing of clinical isolates of Gram-negative bacilli collected in Morocco by the ATLAS Global Surveillance Program from 2018 to 2020. Journal of Global Antimicrobial Resistance. 2022;30:23-30.

149. Miller A, McLeod S, Moussa S, Hackel M. Sulbactam-durlobactam is active against recent, multi-drug resistant Acinetobacter baumannii clinical isolates from the Middle East. Open Forum Infectious Diseases. 2020;7:S662.

150. Caglan E, Nigiz S, Sancak B, Gur D. Resistance and heteroresistance to colistin among clinical isolates of Acinetobacter baumannii. Acta Microbiologica Et Immunologica Hungarica. 2020;67(2):107-11.

151. Yardimci AC, Arman D. Prevalence and Antimicrobial Resistance of Bloodstream Infections Caused by ESKAPEEc Pathogens: A Five-Year Analysis. Jundishapur Journal of Microbiology. 2022;15(7).

152. Bou G, Maria Otero F, Santiso R, Tamayo M, Fern dC, ez M, et al. Fast Assessment of Resistance to Carbapenems and Ciprofloxacin of Clinical Strains of Acinetobacter baumannii. Journal of Clinical Microbiology. 2012;50(11):3609-13.

153. Donadu MG, Mazzarello V, Cappuccinelli P, Zanetti S, Madlena M, Nagy AL, et al. Relationship between the Biofilm-Forming Capacity and Antimicrobial Resistance in Clinical Acinetobacter baumannii Isolates: Results from a Laboratory-Based In Vitro Study. Microorganisms. 2021;9(11).

154. Li KL, Abad CLR. The clinical profile and outcomes of adult patients given intravenous colistin for multidrug-resistant gram negative infections in a Philippine tertiary hospital. International Journal of Infectious Diseases. 2020;93:9-14.

155. Masoumi-Asl H, Heravi FS, Badamchi A, Khanaliha K, Farsimadan M, Naghadalipoor M, et al. Molecular characterization and antibiotic resistance pattern of isolated Acinetobacter baumannii in Iran. Gene Reports. 2021;24.

156. Ranjbar R, Farahani A. Study of genetic diversity, biofilm formation, and detection of Carbapenemase, MBL, ESBL, and tetracycline resistance genes in multidrug-resistant Acinetobacter baumannii isolated from burn wound infections in Iran. Antimicrobial Resistance and Infection Control. 2019;8(1).

157. Yadav SK, Bhujel R, Hamal P, Mishra SK, Sharma S, Sherch, JB. Burden of Multidrug-Resistant Acinetobacter baumannii Infection in Hospitalized Patients in a Tertiary Care Hospital of Nepal. Infection and Drug Resistance. 2020;13:725-32.

158. Rezaei A, Fazeli H, Moghadampour M, Halaji M, Faghri J. Determination of antibiotic resistance pattern and prevalence of OXA-type carbapenemases among Acinetobacter baumannii clinical isolates from inpatients in Isfahan, central Iran. Le infezioni in medicina. 2018;26(1):61-6.

159. Hou C, Yang F. Drug-resistant gene of blaOXA-23, blaOXA-24, blaOXA-51 and blaOXA-58 in Acinetobacter baumannii. International Journal of Clinical and Experimental Medicine. 2015;8(8):13859-63.

160. Jun SH, Lee DE, Hwang HR, Kim N, Kwon KT, Kim YK, Lee JC. Clonal evolution and antimicrobial resistance of Acinetobacter baumannii isolates from Korean hospitals over the last decade. Infection, genetics and evolution : journal of molecular epidemiology and evolutionary genetics in infectious diseases. 2023;108:105404-.

161. Gomes Chagas TP, Carvalho KR, de Oliveira Santos IC, D'Alincourt Carvalho-Assef AP, Asensi MD. Characterization of carbapenem-resistant Acinetobacter baumannii in Brazil (2008-2011): countrywide spread of OXA-23-producing clones (CC15 and CC79). Diagnostic Microbiology and Infectious Disease. 2014;79(4):468-72.

162. Zhen X, Chen Y, Li Y, Zhang H, Hu X, Dong P, Dong H. IMPACT OF CARBAPENEM RESISTANCE ON CLINICAL AND ECONOMIC OUTCOMES AMONG INPATIENTS WITH ACINETOBACTER BAUMANNII INFECTION OR COLONIZATION IN A HOSPITAL OF ZHEJIANG PROVINCE CHINA. Value in Health. 2017;20(5):A74-A.

163. Naas T, Lina G, Henriksen AS, Longshaw C, Jehl F. In vitro activity of cefiderocol and comparators against isolates of Gram-negative pathogens from a range of infection sources: SIDERO-WT-2014-2018 studies in France. JAC-Antimicrobial Resistance. 2021;3(2):dlab081-Article No.: dlab.

164. Lee M-H, Chen T-L, Lee Y-T, Huang L, Kuo S-C, Yu K-W, et al. Dissemination of multidrug-resistant Acinetobacter baumannii carrying Bla(OxA-23) from hospitals in central Taiwan. Journal of Microbiology Immunology and Infection. 2013;46(6):419-24.

165. Moosavian M, Sirous M, okht, Shams N. Phenotypic and Genotypic Detection of Extended Spectrum beta-lactamase and Carbapenemases Production Including bla TEM, bla PER and bla NDM-1 Genes Among Acinetobacter baumannii Clinical Isolates. Jundishapur Journal of Microbiology. 2017;10(12).

166. Huang YS, Wang JT, Sheng WH, Chuang YC, Chang SC. Comparative in vitro activity of sitafloxacin against bacteremic isolates of carbapenem resistant Acinetobacter baumannii complex. Journal of Microbiology, Immunology and Infection. 2013;48(5):545-51.

167. AliMohammadi A, Chezani-Sharahi N, Hezaveh ZA, Abbasi E, Shariati A, Ghaznavi-Rad E. The significant role of Carbapenems-resistant Acinetobacter Baumannii in mortality rate of patients with COVID-19. Vacunas. 2023;24(1):13-8.

168. Camacho-Ortiz A, Lara-Medrano R, Martínez-Reséndez MF, Mendoza-Olazarán S, Flores-Treviño S, Garza-González E. Efecto de la azitromicina en la producción de biopelículas y la composición de Acinetobacter baumannii resistente a múltiples fármacos. Gaceta medica de Mexico. 2021;157(5):478-83.

169. Shahid A, Muzammil S, Rasheed F, Aslam B, Ali MA, Haider SZ, et al. Emergence of armA Mediated Aminoglycoside Resistance in Multidrug-Resistant Acinetobacter baumannii in Pakistani Hospitals. Pakistan Journal of Zoology. 2021;53(6):2507-10.

170. Jean S-S, Hsueh P-R, Lee W-S, Yu K-W, Liao C-H, Chang F-Y, et al. Carbapenem susceptibilities and non-susceptibility concordance to different carbapenems amongst clinically important Gram-negative bacteria isolated from intensive care units in Taiwan: Results from the Surveillance of Multicentre Antimicrobial Resistance in Taiwan (SMART) in 2009. International Journal of Antimicrobial Agents. 2013;41(5):457-62.

171. Tayebi Z, Doust RH, Rahimi MK, Siadat SD, Goudarzi M. Distribution of different carbapenemase genes in carbapenem-resistant Acinetobacter baumannii strains isolated from intensive care: A two year multi-center study in Tehran, Iran. Gene Reports. 2019;15.

172. Chusri S, Chongsuvivatwong V, Rivera JI, Silpapojakul K, Singkhamanan K, McNeil E, Doi Y. Molecular epidemiology and spatiotemporal analysis of hospital-acquired Acinetobacter baumannii infection in a tertiary care hospital in southern Thailand. Journal of Hospital Infection. 2017;95(1):53-8.

173. Hashemi B, Afkhami H, Khaledi M, Kiani M, Bialvaei AZ, Fathi J, et al. Frequency of Metalo beta Lactamase genes, bla IMP1, INT 1 in Acinetobacter baumanii isolated from burn patients North of Iran. Gene Reports. 2020;21:100800-Article No.: .

174. Gaspar GG, Ferreira LR, Feliciano CS, Campos Júnior CP, Molina FMR, Vendruscolo ACS, et al. Pre-and post-covid-19 evaluation of antimicrobial susceptibility for healthcare-associated infections in the intensive care unit of a tertiary hospital. Revista da Sociedade Brasileira de Medicina Tropical. 2021;54.

175. Khuntayaporn P, Kanathum P, Houngsaitong J, Montakantikul P, Thirapanmethee K, Chomnawang MT. Predominance of international clone 2 multidrug-resistant Acinetobacter baumannii clinical isolates in Thailand: a nationwide study. Annals of Clinical Microbiology and Antimicrobials. 2021;20(1).

176. Mohammadtaheri Z, Pourpaki M, Mohammadi F, Namdar R, Masjedi M-R. Surveillance of Antimicrobial Susceptibility among Bacterial Isolates from Intensive Care Unit Patients of a Tertiary-Care University Hospital in Iran: 2006-2009. Chemotherapy. 2010;56(6):478-84.

177. Jabeen F, Khan Z, Sohail M, Tahir A, Tipu I, Murtaza Saleem HG. Antibiotic Resistance Pattern Of Acinetobacter Baumannii Isolated From Bacteremia Patients In Pakistan. Journal of Ayub Medical College, Abbottabad : JAMC. 2022;34(1):95-100.

178. Rao MR, Urs TA, Chitharagi VB, Shivappa S, Mahale RP, Gowda RS, Shree K. Rapid identification of carbapenemases by CarbAcineto NP test and the rate of beta-lactamases among Acinetobacter baumannii from a teaching hospital. Iranian journal of microbiology. 2022;14(2):174-80.

179. Oteo J, Garcia-Estebanez C, Miguelanez S, Campos J, Marti S, Vila J, et al. Genotypic diversity of imipenem resistant isolates of Acinetobacter baumannii in Spain. Journal of Infection. 2007;55(3):260-6.

180. Zhou Y, Cong Y, Xu Y, Gong M, Deng X, Chen S, Qu F. Cross-sectional and longitudinal studies on antimicrobial susceptibility profiles and the genomic diversity of acinetobacter baumannii isolates from senile patients. International Journal of Clinical and Experimental Medicine. 2016;9(6):11137-46.

181. Mendoza-Olazaran S, Camacho-Ortiz A, Martinez-Resendez MF, Llaca-Diaz JM, Perez-Rodriguez E, Garza-Gonzalez E. Influence of whole-body washing of critically ill patients with chlorhexidine on Acinetobacter baumannii isolates. American Journal of Infection Control. 2014;42(8):874-8.

182. Biglari S, Alfizah H, Ramliza R, Rahman MM. Molecular characterization of carbapenemase and cephalosporinase genes among clinical isolates of Acinetobacter baumannii in a tertiary medical centre in Malaysia. Journal of Medical Microbiology. 2015;64:53-8.

183. Biglari S, Hanafiah A, Puzi SM, Ramli R, Rahman MM, Lopes BS. Antimicrobial Resistance Mechanisms and Genetic Diversity of Multidrug-Resistant Acinetobacter baumannii Isolated from a Teaching Hospital in Malaysia. Microbial Drug Resistance. 2017;23(5):545-55.

184. Farajnia S, Lotfi F, Dehnad A, Shojaie M, Raisi R, Rahbarnia L, et al. The molecular characterization of colistin-resistant isolates of Acinetobacter baumannii from patients at intensive care units. Iranian Journal of Microbiology. 2022;14(3):319-27.

185. Ghasemi S, Shoja S, Mazloomirad F, Ghatee MA, Rashidpoor F, Khoramrooz SS, et al. Prevalence of Aminoglycoside and Carbapenemase Resistance Genes and Biofilm Formation among Clinical Isolates of Acinetobacter baumannii in Iran. Mediterranean Journal of Infection, Microbes and Antimicrobials. 2022;11(1).

186. Xiao X-m, Gao S, Duan J-j, Yao B, Zhang J. Resistant mechanisms and molecular epidemiological characteristics of carbapenem-resistant Acinetobacter baumannii. Zhongguo Kangshengsu Zazhi. 2017;42(8):704-10.

187. Nordmann P, Picazo JJ, Mutters R, Korten V, Quintana A, Laeuffer JM, et al. Comparative activity of carbapenem testing: the COMPACT study. Journal of Antimicrobial Chemotherapy. 2011;66(5):1070-8.

188. Salehi B, Goudarzi H, Nikmanesh B, Houri H, Alavi-Moghaddam M, Ghalav, Z. Emergence and characterization of nosocomial multidrug-resistant and extensively drug-resistant Acinetobacter baumannii isolates in Tehran, Iran. Journal of Infection and Chemotherapy. 2018;24(7):515-23.

189. Lowe M, Ehlers MM, Ismail F, Peirano G, Becker PJ, Pitout JDD, Kock MM. Acinetobacter baumannii: Epidemiological and Beta-Lactamase Data From Two Tertiary Academic Hospitals in Tshwane, South Africa. Frontiers in Microbiology. 2018;9.

190. Chmielarczyk A, Pilarczyk-Zurek M, Kaminska W, Pobiega M, Romaniszyn D, Ziolkowski G, et al. Molecular Epidemiology and Drug Resistance of Acinetobacter baumannii Isolated from Hospitals in Southern Poland: ICU as a Risk Factor for XDR Strains. Microbial Drug Resistance. 2016;22(4):328-35.

191. Pogue JM, Kanakamedala H, Zhou Y, Cai B. Burden of illness in carbapenem-resistant acinetobacter baumannii infections in us hospitals (2014 to 2018). Open Forum Infectious Diseases. 2019;6:S262.

192. Shrestha S, Tada T, Miyoshi-Akiyama T, Ohara H, Shimada K, Satou K, et al. Molecular epidemiology of multidrug-resistant Acinetobacter baumannii isolates in a university hospital in Nepal reveals the emergence of a novel epidemic clonal lineage. International Journal of Antimicrobial Agents. 2015;46(5):526-31.

193. Zhang F, Li Y, Lv Y, Zheng B, Xue F. Bacterial susceptibility in bloodstream infections: Results from China Antimicrobial Resistance Surveillance Trial (CARST) Program, 2015-2016. Journal of Global Antimicrobial Resistance. 2019;17:276-82.

194. Bawazeer R, Algoribi M, Abujamel T, Okdah L, Alzayer M, Alarfaj R, et al. Phenotypic and molecular characterization of Acinetobacter baumannii clinical isolates in Saudi Arabia. Journal of Infection and Public Health. 2020;13(2):327.

195. Talizin TB, Dantas de Maio Carrilho CM, Carvalho Grion CM, Queiroz Cardoso LT, Tanita MT, Boll KM, et al. Polymyxin for treatment of ventilator-associated pneumonia in a setting of high carbapenem resistance. Plos One. 2020;15(8).

196. Balkhair A, Al-Muharrmi Z, Al'Adawi B, Al Busaidi I, Taher HB, Al-Siyabi T, et al. Prevalence and 30-day all-cause mortality of carbapenem-and colistin-resistant bacteraemia caused by Acinetobacter baumannii, Pseudomonas aeruginosa, and Klebsiella pneumoniae: Description of a decade-long trend. International Journal of Infectious Diseases. 2019;85:10-5.

197. Amudhan SM, Sekar U, Arunagiri K, Sekar B. OXA betaβ-lactamase-mediated carbapenem resistance in Acinetobacter baumannii. Indian Journal of Medical Microbiology. 2011;29(3):269-74.

198. Amudhan MS, Sekar U, Kamalanathan A, Balaraman S. bla(IMP) and bla(VIM) mediated carbapenem resistance in Pseudomonas and Acinetobacter species in India. Journal of Infection in Developing Countries. 2012;6(11):757-62.

199. Vuotto C, Grosso F, Longo F, Balice MP, de Barros MC, Peixe L, Donelli G. Biofilm-forming ability and clonality in acinetobacter baumannii strains isolated from urine samples and urinary catheters in different European hospitals. 2018. p. 73-83.

200. Ejaz H, Ahmad M, Younas S, Junaid K, Abosalif KOA, Abdalla AE, et al. Molecular Epidemiology of Extensively-Drug Resistant Acinetobacter baumannii Sequence Type 2 Co-Harboring bla(NDM) and bla(OXA) From Clinical Origin. Infection and Drug Resistance. 2021;14:1931-9.

201. Bagheri Josheghani S, Moniri R, Firoozeh F, Sehat M, Dasteh Goli Y. Susceptibility Pattern and Distribution of Oxacillinases and bla PER-1 Genes among Multidrug Resistant Acinetobacter baumannii in a Teaching Hospital in Iran. Journal of pathogens. 2015;2015:957259-.

202. Vahhabi A, Hasani A, Rezaee MA, Baradaran B, Hasani A, Kafil HS, Soltani E. Carbapenem resistance in Acinetobacter baumannii clinical isolates from northwest Iran: high prevalence of OXA genes in sync. Iranian journal of microbiology. 2021;13(3):282-93.

203. Dobrovic K, Skrobo T, Selec K, Jelic M, Civljak R, Persec J, et al. Healthcare-Associated Bloodstream Infections Due to Multidrug-Resistant Acinetobacter baumannii in COVID-19 Intensive Care Unit: A Single-Center Retrospective Study. Microorganisms. 2023;11(3).

204. Hafiz TA, Alghamdi SS, Mubaraki MA, Alghamdi SSM, Alothaybi A, Aldawood E, Alotaibi F. A two-year retrospective study of multidrug-resistant Acinetobacter baumannii respiratory infections in critically Ill patients: Clinical and microbiological findings. Journal of Infection and Public Health. 2023;16(3):313-9.

205. Smitran A, ra, Lukovic B, Bozic L, Jelic D, Jovicevic M, et al. Carbapenem-Resistant Acinetobacter baumannii: Biofilm-Associated Genes, Biofilm-Eradication Potential of Disinfectants, and Biofilm-Inhibitory Effects of Selenium Nanoparticles. Microorganisms. 2023;11(1).

206. Jeannot K, Diancourt L, Vaux S, Thouverez M, Ribeiro A, ina, et al. Molecular Epidemiology of Carbapenem Non-Susceptible Acinetobacter baumannii in France. Plos One. 2014;9(12).

207. Gholami M, Moshiri M, Ahanjan M, Salimi Chirani A, Hasannejad Bibalan M, Asadi A, et al. The diversity of class B and class D carbapenemases in clinical Acinetobacter baumannii isolates. Le infezioni in medicina. 2018;26(4):329-35.

208. Miller A, McLeod S, Mathur T, Morrissey I. In vitro antibacterial activity of sulbactam-durlobactam (ETX2514SUL) against 121 recent acinetobacter baumannii isolated from patients in India. Open Forum Infectious Diseases. 2019;6:S314.

209. Khuntayaporn P, Thirapanmethee K, Kanathum P, Chitsombat K, Chomnawang MT. Comparative study of phenotypic-based detection assays for carbapenemase-producing Acinetobacter baumannii with a proposed algorithm in resource-limited settings. Plos One. 2021;16(11).

210. Meshkat Z, Amini Y, Sadeghian H, Salimiz, H. ISAba1/bla(OXA-23-like) family is the predominant cause of carbapenem resistance in Acinetobacter baumannii and Acinetobacter nosocomialis in Iran. Infection Genetics and Evolution. 2019;71:60-6.

211. Rosales-Reyes R, Gayosso-Vazquez C, Fern L, ez-Vazquez J, Dolores Jarillo-Quijada M, Rivera-Benitez C, et al. Virulence profiles and innate immune responses against highly lethal, multidrug-resistant nosocomial isolates of Acinetobacter baumannii from a tertiary care hospital in Mexico. Plos One. 2017;12(8).

212. Yu K, Zeng W, Xu Y, Liao W, Xu W, Zhou T, et al. Bloodstream infections caused by ST2 Acinetobacter baumannii: risk factors, antibiotic regimens, and virulence over 6 years period in China. Antimicrobial Resistance and Infection Control. 2021;10(1).

213. Ziglam H, Elahmer O, Amri S, Shareef F, Grera A, Labeeb M, Zorgani A. Antimicrobial Resistance Patterns Among Acinetobacter Baumannii Isolated From Burn Intensive Care Unit In Tripoli, Libya. International Arabic Journal of Antimicrobial Agents. 2012;2(3).

214. Nikibakhsh M, Firoozeh F, Badmasti F, Kabir K, Zibaei M. Molecular study of metallo-beta-lactamases and integrons in Acinetobacter baumannii isolates from burn patients. Bmc Infectious Diseases. 2021;21(1).

215. de Oliveira EA, de Paula GR, Juan Mondino PJ, Gomes Chagas TP, Bona de Mondino SS, Vieira de Mendonca-Souza CR. High rate of detection of OXA-23-producing Acinetobacter from two general hospitals in Brazil. Revista Da Sociedade Brasileira De Medicina Tropical. 2019;52.

216. Hu F, Yuan L, Yang Y, Xu Y, Huang Y, Hu Y, et al. A multicenter investigation of 2,773 cases of bloodstream infections based on China antimicrobial surveillance network (CHINET). Frontiers in Cellular and Infection Microbiology. 2022;12.

217. Karmostaji A, Najar Peerayeh S, Hatef Salmanian A. Distribution of OXA-type class D β-lactamase genes among nosocomial multi drug resistant Acinetobacter baumannii isolated in Tehran hospitals. Jundishapur Journal of Microbiology. 2013;6(5).

218. Chen R, Rui Q, Guo T, Wang T, Li J, Yang Z, et al. Clinical and microbiological features of nosocomial blood stream infections in intensive care units. Chinese Journal of Infection and Chemotherapy. 2016;16(6):673-9.

219. Mir G, a-Novales M, Flores-Moreno K, Lopez-Vidal Y, Rodriguez-Alvarez M, Solorzano-Santos F, et al. Antimicrobial resistance and antibiotic consumption in Mexican hospitals. Salud Publica De Mexico. 2020;62(1):42-9.

220. Mostafavi S, Rostami S, Nokhodian Z, Ataei B, Cheraghi A, Ataabadi P, et al. Antibacterial resistance patterns of Acinetobacter baumannii complex: The results of Isfahan Antimicrobial Resistance Surveillance-1 Program. Asian Pacific Journal of Tropical Medicine. 2021;14(7):316-22.

221. Mabrouk SS, Abdellatif GR, El-Ansary MR, Aboshanab KM, Ragab YM. Carbapenemase Producers Among Extensive Drug-Resistant Gram-Negative Pathogens Recovered from Febrile Neutrophilic Patients in Egypt. Infection and Drug Resistance. 2020;13:3113-24.

222. Al-Hashem G, Rotimi VO, Albert MJ. Antimicrobial Resistance of Serial Isolates of Acinetobacter baumannii Colonizing the Rectum of Adult Intensive Care Unit Patients in a Teaching Hospital in Kuwait. Microbial Drug Resistance. 2021;27(1):64-72.

223. Simeon P, Godman B, Kalemeera F. Antibiotics' susceptibility patterns of bacterial isolates causing lower respiratory tract infections in ICU patients at referral hospitals in Namibia. Hospital practice (1995). 2021;49(5):356-63.

224. Abozahra R, Abdelhamid SM, Elsheredy AG, Abdulwahab KE, Baraka K. Genotyping and Molecular Characterization of Carbapenem-resistant Acinetobacter baumannii Strains Isolated from Intensive Care Unit Patients. Microbiology and Biotechnology Letters. 2021;49(2):239-48.

225. Saadati M, Rahbarnia L, Farajnia S, Naghili B, Mohammadzadeh R. The prevalence of biofilm encoding genes in multidrug-resistant Acinetobacter baumannii isolates. Gene Reports. 2021;23:101094-Article No.: .

226. Sepahv, S, Madani M, Davarpanah MA, Gh, ehari F. Evaluation antibiotic resistance and presence of bla(OXA-51), bla(OXA-58) and blaOXA-23 genes in Acinetobacter baumannii strains via multiplex PCR. Pakistan Journal of Pharmaceutical Sciences. 2021;34(5):1667-71.

227. Fallah F, Noori M, Hashemi A, Goudarzi H, Karimi A, Erfanimanesh S, Alimehr S. Prevalence of bla NDM, bla PER, bla VEB, bla IMP, and bla VIM Genes among Acinetobacter baumannii Isolated from Two Hospitals of Tehran, Iran. Scientifica. 2014;2014:245162-.

228. Noori M, Mohsenzadeh B, Bahramian A, Shahi F, Mirzaei H, Khoshnood S. Characterization and frequency of antibiotic resistance related to membrane porin and efflux pump genes among Acinetobacter baumannii strains obtained from burn patients in Tehran, Iran. Journal of Acute Disease. 2019;8(2):63-6.

229. Alavi-Moghaddam M, Dolati M, Javadi A, Saki B, Karami-Zar, i M, Khoshnood S. Molecular detection of oxacillinase genes and typing of clinical isolates of Acinetobacter baumannii in Tehran, Iran. Journal of Acute Disease. 2020;9(1):33-9.

230. Moosavian M, Ahmadi K, Shoja S, Mardaneh J, Shahi F, Afzali M. Antimicrobial resistance patterns and their encoding genes among clinical isolates of Acinetobacter baumannii in Ahvaz, Southwest Iran. MethodsX. 2020;7:101031-Article No.: .

231. Mushtaq S, Sadouki Z, Vickers A, Livermore DM, Woodford N. In Vitro Activity of Cefiderocol, a Siderophore Cephalosporin, against Multidrug-Resistant Gram-Negative Bacteria. Antimicrobial Agents and Chemotherapy. 2020;64(12).

232. Jia P, Zhu Y, Zhang H, Cheng B, Guo P, Xu Y, Yang Q. In vitro activity of ceftaroline, ceftazidime-avibactam, and comparators against Gram-positive and -negative organisms in China: the 2018 results from the ATLAS program. Bmc Microbiology. 2022;22(1).

233. Tan TY, Hsu LY, Koh TH, Ng LS, Tee NWS, Krishnan P, et al. Antibiotic Resistance in Gram-negative Bacilli: A Singapore Perspective. Annals Academy of Medicine Singapore. 2008;37(10):819-25.

234. Uzunoglu E, Direkel S, Kocbiyik M, Uludag SK, Cicek AC. CO-EXISTANCE OF ISABA1/BLA(OXA-51/23) IS INCREASING IN CARBAPENEM RERSISTANT ACINETOBACTER BAUMANNII ISOLATES IN TURKEY. Acta Medica Mediterranea. 2017;33(6):1001-+.

235. Mohammadi F, Goudarzi H, Hashemi A, Nojookambari NY, Khoshnood S, Sabzehali F. Detection of ISAba1 in acinetobacter baumannii strains carrying OXA genes isolated from Iranian burns patients. Archives of Pediatric Infectious Diseases. 2017;5(2).

236. Loraine J, Heinz E, Soontarach R, Blackwell GA, Stabler RA, Voravuthikunchai SP, et al. Genomic and Phenotypic Analyses of Acinetobacter baumannii Isolates From Three Tertiary Care Hospitals in Thailand. Frontiers in Microbiology. 2020;11.

237. Hsieh Y-C, Wang S-H, Chen Y-Y, Lin T-L, Shie S-S, Huang C-T, et al. Association of capsular types with carbapenem resistance, disease severity, and mortality inAcinetobacter baumannii. Emerging Microbes & Infections. 2020;9(1):2094-104.

238. Abbasi E, Goudarzi H, Hashemi A, Chirani AS, Ardebili A, Goudarzi M, et al. Decreased carO gene expression and OXA-type carbapenemases among extensively drug-resistant Acinetobacter baumannii strains isolated from burn patients in Tehran, Iran. Acta Microbiologica Et Immunologica Hungarica. 2021;68(1):48-54.

239. Ranjbar R, Zayeri S, Afshar D, Farshad S. Detection of OXA beta lactamases among clinical isolates of Acinetobacter baumannii isolated from Tehran Hospitals, Iran. Open Microbiology Journal. 2019;13(1):68-72.

240. Sader HS, Rhomberg PR, Duncan LR, Locher HH, Dale GE, Flamm RK. Antimicrobial activity of POL7306 tested against clinical isolates of Gram-negative bacteria collected worldwide. Journal of Antimicrobial Chemotherapy. 2020;75(6):1518-24.

241. Karami F, Nazari R, Adeli H. Detection of Genes Encoding Metallo-beta-lactamases in Carbapenem Resistant Acinetobacter baumannii. Journal of Kerman University of Medical Sciences. 2021;28(6):559-67.

242. Coskun USS, Caliskan E, Cicek AC, Turumtay H, alli C. beta-lactamase genes in carbapenem resistance Acinetobacter baumannii isolates from a Turkish university hospital. Journal of Infection in Developing Countries. 2019;13(1):50-5.

243. Petazzoni G, Bellinzona G, Merla C, Corbella M, Samuelsen Ø, Cor, et al. The COVID-19 pandemic sparked off a large-scale outbreak of carbapenem-resistant Acinetobacter baumannii from the endemic strains of an Italian hospital. 2022.

244. Odewale G, Adefioye OJ, Ojo J, Adewumi FA, Olowe OA. Multidrug Resistance of Acinetobacter Baumannii in Ladoke Akintola University Teaching Hospital, Osogbo, Nigeria. European journal of microbiology & immunology. 2016;6(3):238-43.

245. Moghadam MN, Motamedifar M, Sarvari J, Sedigh E-SH, Mousavi SM, Moghadam FN. Emergence of Multidrug Resistance and Metallo-beta-lactamase Producing Acinetobacter baumannii Isolated from Patients in Shiraz, Iran. Annals of medical and health sciences research. 2016;6(3):162-7.

246. Ahmadikiya F, Mosadegh A, Moradi M, Hossieni-Nave H. Antimicrobial resistance patterns and frequency of extended-spectrum beta-lactamase genes among Acinetobacter baumannii. Journal of Babol University of Medical Sciences. 2017;19(7):28-34.

247. Atik TK, Özyurt M, Atik B, Bektöre B, Selek BM, Çetinkaya RA, et al. Identification of oxa-gene in acinetobacter baumannii isolates obtained from clinical specimens and the clonality between these isolates. Nobel Medicus. 2019;15(3):44-51.

248. Nogbou N-D, Phofa DT, Nchabeleng M, Musyoki AM. Investigating multi-drug resistant Acinetobacter baumannii isolates at a tertiary hospital in Pretoria, South Africa. Indian Journal of Medical Microbiology. 2021;39(2):218-23.

249. Ruekit S, Srijan A, Serichantalergs O, Margulieux KR, Mc Gann P, Mills EG, et al. Molecular characterization of multidrug-resistant ESKAPEE pathogens from clinical samples in Chonburi, Thailand (2017-2018). Bmc Infectious Diseases. 2022;22(1).

250. Maraki S, Mavros MN, Kofteridis DP, Samonis G, Falagas ME. Epidemiology and Antimicrobial Sensitivities of 536 Multi-Drug-Resistant Gram-Negative Bacilli Isolated from Patients Treated on Surgical Wards. Surgical Infections. 2012;13(5):326-31.

251. Safari M, Saidijam M, Bahador A, Jafari R, Alikhani MY. High prevalence of multidrug resistance and metallo-beta-lactamase (MbetaL) producing Acinetobacter baumannii isolated from patients in ICU wards, Hamadan, Iran. Journal of research in health sciences. 2013;13(2):162-7.

252. Cicek AC, Saral A, Iraz M, Ceylan A, Duzgun AO, Peleg AY, alli C. OXA- and GES-type beta-lactamases predominate in extensively drug-resistant Acinetobacter baumannii isolates from a Turkish University Hospital. Clinical Microbiology and Infection. 2014;20(5):410-5.

253. Lee H, Yoon E-J, Kim D, Jeong SH, Won EJ, Shin JH, et al. Antimicrobial resistance of major clinical pathogens in South Korea, May 2016 to April 2017: first one-year report from Kor-GLASS. Eurosurveillance. 2018;23(42):17-27.

254. Mohammadi M, Soroush S, Delfani S, Pakzad I, Abbaszadeh A, Bahmani M, et al. Distribution of Class D Carbapenemase and Extended-Spectrum beta-Lactamase Genes among Acinetobacter Baumannii Isolated from Burn Wound and Ventilator Associated Pneumonia Infections. Journal of clinical and diagnostic research : JCDR. 2017;11(7):DC19-DC23.

255. Sharma N, Thapa B, Acharya A, Raghubanshi BR. Meropenem Resistance among Acinetobacter Positive Clinical Samples in a Tertiary Care Centre in Nepal: A Descriptive Cross-sectional Study. Journal of Nepal Medical Association. 2021;59(241):853-7.

256. Soltani B, Heidari H, Ebrahim-Saraie HS, Hadi N, Mardaneh J, Motamedifar M. Molecular characteristics of multiple and extensive drug-resistant Acinetobacter baumannii isolates obtained from hospitalized patients in Southwestern Iran. Le infezioni in medicina. 2018;26(1):67-76.

257. Kirkgoz E, Zer Y. Clonal comparison of Acinetobacter strains isolated from intensive care patients and the intensive care unit environment. Turkish Journal of Medical Sciences. 2014;44(4):643-8.

258. Sultan AM, Seliem WA. Identifying Risk Factors for Healthcare-Associated Infections Caused by Carbapenem-Resistant Acinetobacter baumannii in a Neonatal Intensive Care Unit. Sultan Qaboos University medical journal. 2018;18(1):e75-e80.

259. Zhang Y, Ding F, Luo Y, Fan B, Tao Z, Li Y, Gu D. Distribution pattern of carbapenemases and solitary contribution to resistance in clinical strains of Acinetobacter baumannii. Annals of Palliative Medicine. 2021;10(8):9184-91.

260. Erdem I, Yildirim I, Safak B, Karaali R, Erdal B, Ardic E, et al. A 5-year surveillance of healthcare-associated infections in a university hospital: A retrospective analysis. SAGE open medicine. 2022;10:20503121221091789-.

261. Almaghrabi MK, Joseph MRP, Assiry MM, Hamid ME. Multidrug-Resistant Acinetobacter baumannii: An Emerging Health Threat in Aseer Region, Kingdom of Saudi Arabia. Canadian Journal of Infectious Diseases & Medical Microbiology. 2018;2018.

262. Scheetz MH, Qi C, Warren JR, Postelnick MJ, Zembower T, Obias A, Noskin GA. In vitro activities of various antimicrobials alone and in combination with tigecycline against carbapenem-intermediate or -resistant Acinetobacter baumannii. Antimicrob Agents Chemother. 2007;51(5):1621-6.

263. Sadr M, Fahimzad SA, Karimi A, Fallah F, Armin S, Tehrani NA, et al. Antimicrobial resistance and molecular epidemiology of virulence genes among multi-drug resistant Acinetobacter baumannii clinical isolates in Iran. Gene Reports. 2021;24:101281-Article No.: .

264. Xu X, Xu C, Salisu RB, Xu W. Beta-Lactamase Gene Expression Level of Hospital-Acquired CRAB Isolated from Children in Picu. Infection and Drug Resistance. 2021;14:3195-205.

265. Rani FM, Rahman NIA, Ismail S, Abdullah FH, Othman N, Alattraqchi AG, et al. Prevalence and antimicrobial susceptibilities of Acinetobacter baumannii and non-baumannii Acinetobacters from Terengganu, Malaysia and their carriage of carbapenemase genes. Journal of Medical Microbiology. 2018;67(11):1538-43.

266. Shi X, Wang H, Wang X, Jing H, Duan R, Qin S, et al. Molecular characterization and antibiotic resistance of Acinetobacter baumannii in cerebrospinal fluid and blood. Plos One. 2021;16(2).

267. Garcia-Rodriguez JA, Jones RN, Mystic Programme Study G. Antimicrobial resistance in gram-negative isolates from European intensive care units: Data from the Meropenem Yearly Susceptibility Test Information Collection (MYSTIC) Programme. Journal of Chemotherapy. 2002;14(1):25-32.

268. Kumar S, Patil PP, Singhal L, Ray P, Patil PB, Gautam V. Molecular epidemiology of carbapenem-resistant Acinetobacter baumannii isolates reveals the emergence of bla(OXA-23) and bla(NDM-1) encoding international clones in India. Infection Genetics and Evolution. 2019;75.

269. Liu P-Y, Lee Y-L, Lu M-C, Shao P-L, Lu P-L, Chen Y-H, et al. National Surveillance of Antimicrobial Susceptibility of Bacteremic Gram-Negative Bacteria with Emphasis on Community-Acquired Resistant Isolates: Report from the 2019 Surveillance of Multicenter Antimicrobial Resistance in Taiwan (SMART). Antimicrobial Agents and Chemotherapy. 2020;64(10).

270. Sonnevend A, Ghazawi A, Al Munthari N, Pitout M, Hamadeh MB, Hashmey R, et al. Characteristics of epidemic and sporadic strains of Acinetobacter baumannii isolated in Abu Dhabi hospitals. Journal of Medical Microbiology. 2013;62:582-90.

271. Chen Z, Liu W, Zhang Y, Li Y, Jian Z, Deng H, et al. Molecular epidemiology of carbapenem-resistant Acinetobacter spp. from XiangYa Hospital, in Hunan Province, China. Journal of Basic Microbiology. 2013;53(2):121-7.

272. Cuong Hoang Q, Thao Nguyen Thi P, Hai Nguyen D, Trung Tran L, Hang Tran Thi T, Si Nguyen T, Lan Phan T. Carbapenemase Genes and Multidrug Resistance of Acinetobacter Baumannii: A Cross Sectional Study of Patients with Pneumonia in Southern Vietnam. Antibiotics-Basel. 2019;8(3).

273. Ergin A, Hascelik G, Eser OK. Molecular characterization of oxacillinases and genotyping of invasive Acinetobacter baumannii isolates using repetitive extragenic palindromic sequence-based polymerase chain reaction in Ankara between 2004 and 2010. Scandinavian Journal of Infectious Diseases. 2013;45(1):26-31.

274. Bahrami S, Shafiee F, Hakamifard A, Fazeli H, Soltani R. Antimicrobial susceptibility pattern of carbapenemase-producing Gram-negative nosocomial bacteria at Al Zahra hospital, Isfahan, Iran. Iranian journal of microbiology. 2021;13(1):50-7.

275. Gao J, Song J. Clinical analysis of distribution and drug resistance of pathogenic bacteria in blood culture of Dalian Municipal Central Hospital from 2015 to 2019. Pakistan Journal of Medical Sciences. 2022;38(7).

276. Moradi N, Kazemi N, Ghaemi M, Mirzaei B. Frequency and antimicrobial resistance pattern of bacterial isolates from patients with COVID-19 in two hospitals of Zanjan. Iranian Journal of Microbiology. 2021;13(6):769-78.

277. Lucas Kurihara MN, de Sales RO, da Silva KE, Silva GD, Tazinazzo Mansano MC, Mahmoud FF, Simionatto S. High lethality rate of carbapenem-resistant Acinetobacter baumannii in Intensive Care Units of a Brazilian hospital: An epidemiologic surveillance study. Revista Da Sociedade Brasileira De Medicina Tropical. 2022;55.

278. Liu C-l, Xu M, Li X-g, Ming L. Distribution and drug resistance of pathogens in blood culture in a teaching hospital from 2014 to 2018. Zhongguo Kangshengsu Zazhi. 2020;45(6):589-95.

279. Lowings M, Ehlers MM, Dreyer AW, Kock MM. High prevalence of oxacillinases in clinical multidrug-resistant Acinetobacter baumannii isolates from the Tshwane region, South Africa - an update. Bmc Infectious Diseases. 2015;15.

280. Moazzen Z, Eslami G, Hashemi A, Nojookambari NY. Efflux pump inhibitor carbonyl cyanide 3-chlorophenylhydrazone (CCCP) effect on the minimum inhibitory concentration of ciprofloxacin in Acinetobacter baumannii strains. Archives of Clinical Infectious Diseases. 2018;13(2).

281. Zendegani E, Dolatabadi S. The Efficacy of Imipenem Conjugated with Synthesized Silver Nanoparticles Against Acinetobacter baumannii Clinical Isolates, Iran. Biological Trace Element Research. 2020;197(1):330-40.

282. Acer O, Ozudogru O, Bahce YG. Evaluation of bacterial agents isolated from endotracheal aspirate cultures of Covid-19 general intensive care patients and their antibiotic resistance profiles compared to pre-pandemic conditions. Microbial Pathogenesis. 2022;164.

283. You Q, Du X, Hu N, Zhang Y, Zhang N, Wang F, et al. Local characteristics of molecular epidemiolgy of Acinetobacter baumannii in Jilin province (northeast China). Bmc Microbiology. 2023;23(1).

284. Nie XM, Huang PN, Ye QF, Wan QQ. The Distribution, Drug Resistance, and Clinical Characteristics of Acinetobacter baumannii Infections in Solid Organ Transplant Recipients. Transplantation Proceedings. 2015;47(10):2860-4.

285. Kaur A, eep, Singh S. Prevalence of Extended Spectrum Betalactamase (ESBL) and Metallobetalactamase (MBL) Producing Pseudomonas aeruginosa and Acinetobacter baumannii Isolated from Various Clinical Samples. Journal of pathogens. 2018;2018:6845985-.

286. Kafshnouchi M, Safari M, Khodavirdipour A, Bahador A, Hashemi SH, Alikhani MS, et al. Molecular Detection of blaOXA -type Carbapenemase Genes and Antimicrobial Resistance Patterns among Clinical Isolates of Acinetobacter baumannii. Global medical genetics. 2022;9(2):118-23.

287. Mohajeri P, Farahani A, Feizabadi MM, Ketabi H, Abiri R, Najafi F. Antimicrobial susceptibility profiling and genomic diversity of Acinetobacter baumannii isolates: A study in western Iran. Iranian journal of microbiology. 2013;5(3):195-202.

288. Maspi H, Hosseini HM, Amin M, Fooladi AAI. High prevalence of extensively drug-resistant and metallo beta-lactamase-producing clinical Acinetobacter baumannii in Iran. Microbial Pathogenesis. 2016;98:155-9.

289. Hajjar Soudeiha M, Dahdouh E, Daoud Z, Sarkis DK. Phenotypic and genotypic detection of β-lactamases in Acinetobacter spp. isolates recovered from Lebanese patients over a 1-year period. J Glob Antimicrob Resist. 2018;12:107-12.

290. Levy-Blitchtein S, Roca I, Plasencia-Rebata S, Vicente-Taboada W, Velásquez-Pomar J, Muñoz L, et al. Emergence and spread of carbapenem-resistant Acinetobacter baumannii international clones II and III in Lima, Peru article. Emerging Microbes and Infections. 2018;7(1).

291. Chen Y, Yang Y, Liu L, Qiu G, Han X, Tian S, et al. High prevalence and clonal dissemination of OXA-72-producing Acinetobacter baumannii in a Chinese hospital: a cross sectional study. Bmc Infectious Diseases. 2018;18.

292. Gheorghe I, Cristea VC, Marutescu L, Popa M, Murariu C, Trusca BS, et al. Resistance and Virulence Features in Carbapenem-resistant Acinetobacter baumannii Community Acquired and Nosocomial Isolates in Romania. Revista De Chimie. 2019;70(10):3502-7.

293. Said KB, Alsolami A, Khalifa AM, Khalil NA, Moursi S, Osman A, et al. A Multi-Point Surveillance for Antimicrobial Resistance Profiles among Clinical Isolates of Gram-Negative Bacteria Recovered from Major Ha'il Hospitals, Saudi Arabia. Microorganisms. 2021;9(10).

294. Khrulnova SA, Korobova AG, Fedorova AV, Frolova IN, Klyasova GA. Change in the Clonal Structure of Carbapenem not Susceptible Acinetobacter baumannii Isolated from the Blood Culture of Patients with Hematological Malignancies. Molecular Genetics Microbiology and Virology. 2020;35(3):145-51.

295. Wei Z, Zhou S, Zhang Y, Zheng L, Zhao L, Cui Y, Xie K. Microbiological characteristics and risk factors on prognosis associated with Acinetobacter baumannii bacteremia in general hospital: A single-center retrospective study. Frontiers in Microbiology. 2022;13.

296. Somily AM, Absar MM, Arshad MZ, Al Aska AI, Shakoor ZA, Fatani AJ, et al. Antimicrobial susceptibility patterns of multidrug-resistant Pseudomonas aeruginosa and Acinetobacter baumannii against carbapenems, colistin, and tigecycline. Saudi Medical Journal. 2012;33(7):750-5.

297. de Azevedo FKSF, Dutra V, Nakazato L, Mello CM, Pepato MA, Hayakawa Ito de Sousa A, et al. Molecular epidemiology of multidrug-resistant Acinetobacter baumannii infection in two hospitals in Central Brazil: the role of ST730 and ST162 in clinical outcomes. Journal of Medical Microbiology. 2019;68(1):31-40.

298. Morrissey I, Olesky M, Hawser S, Lob SH, Karlowsky JA, Corey GR, et al. In Vitro Activity of Eravacycline against Gram-Negative Bacilli Isolated in Clinical Laboratories Worldwide from 2013 to 2017. Antimicrobial Agents and Chemotherapy. 2020;64(3).

299. Li Y, Wang B, Lu F, Ahn J, Zhang W, Cai L, et al. Synergistic Inhibitory Effect of Polymyxin B in Combination with Ceftazidime against Robust Biofilm Formed by Acinetobacter baumannii with Genetic Deficiency in AbaI/AbaR Quorum Sensing. Microbiology Spectrum. 2022;10(1).

300. Direkel S, Cicek AC, Karagoz A, Aydogan Ejder N, Oktay E, Delialioglu N, et al. Antimicrobial Susceptibility and Molecular Characterization of Multidrug-Resistant Acinetobacter baumannii Isolated in an University Hospital. Mikrobiyoloji Bulteni. 2016;50(4):522-34.

301. Shah MW, Yasir M, Farman M, Jiman-Fatani AA, Almasaudi SB, Alawi M, et al. Antimicrobial Susceptibility and Molecular Characterization of Clinical Strains of Acinetobacter baumannii in Western Saudi Arabia. Microbial Drug Resistance. 2019;25(9):1297-305.

302. Kim YJ, Kim SI, Kim YR, Hong KW, Wie SH, Park YJ, et al. Carbapenem-resistant Acinetobacter baumannii: diversity of resistant mechanisms and risk factors for infection. Epidemiology and Infection. 2012;140(1):137-45.

303. Karmostaj A, Peerayeh SN, Salmanian AH. Emergence of Tigecycline Resistant Acinetobacter baumannii From an Intensive Care Unit (ICU) in Tehran. Jundishapur Journal of Microbiology. 2013;6(3):215-9.

304. Nasrolahei M, Zahedi B, Bahador A, Saghi H, Kholdi S, Jalalv, et al. Distribution of bla (OXA-23), ISAba, Aminoglycosides resistant genes among burned & ICU patients in Tehran and Sari, Iran. Annals of Clinical Microbiology and Antimicrobials. 2014;13.

305. Subagdja MFM, Sugianli AK, Prodjosoewojo S, Hartantri Y, Parwati I. Antibiotic Resistance in COVID-19 with Bacterial Infection: Laboratory-Based Surveillance Study at Single Tertiary Hospital in Indonesia. Infection and Drug Resistance. 2022;15:5849-56.

306. Amin M, Navidifar T, Saleh Shooshtari F, Goodarzi H. Association of the genes encoding Metallo-β-Lactamase with the presence of integrons among multidrug-resistant clinical isolates of Acinetobacter baumannii. Infect Drug Resist. 2019;12:1171-80.

307. Selvi VTT, Girija SAS, Priyadharsini VJ. Co-occurrence of bap gene among multi-drug resistant strains of Acinetobacter baumannii from India. Acta Microbiologica Hellenica. 2021;66(2):145-54.

308. Thampithak A, Chaisiri K, Siangsuebchart O, Phengjaturat K, Aonjumras W, Hemapanpairoa J. Prescription Pattern of Intravenous Fosfomycin in a Provincial Hospital in Thailand. Infection & chemotherapy. 2022;54(4):699-710.

309. Esterly JS, Qi C, Malczynski M, Scheetz MH. Predictability of Doripenem Susceptibility in Acinetobacter baumannii Isolates Based on Other Carbapenem Susceptibilities and bla(OXA) Gene Status. Pharmacotherapy. 2010;30(4):354-60.

310. Zhang X, Gu B, Mei Y, Wen Y, Xia W. Increasing resistance rate to carbapenem among blood culture isolates of Klebsiella pneumoniae, Acinetobacter baumannii and Pseudomonas aeruginosa in a university-affiliated hospital in China, 2004-2011. Journal of Antibiotics. 2015;68(2):115-20.

311. Ghaima KK, Saadedin SMK, Jassim KA. Prevalence of BlaOXA like carbapenemase genes in multidrug resistant Acinetobacter baumannii isolated from burns and wounds in Baghdad hospitals. Research Journal of Pharmaceutical, Biological and Chemical Sciences. 2016;7(3):1247-54.

312. Ranjbar R, Zayeri S, Afshar D. High Frequency of AdeA, AdeB and AdeC Genes among Acinetobacter baumannii Isolates. Iranian Journal of Public Health. 2020;49(8):1539-45.

313. Wolfensberger A, Kuster SP, Marchesi M, Zbinden R, Hombach M. The effect of varying multidrug-resistence (MDR) definitions on rates of MDR gram-negative rods. Antimicrobial Resistance and Infection Control. 2019;8(1).

314. Siqueira MS, Sousa MAB, Bicalho PHN, Sousa LP, Mateo E, Ferreira ACS. Prevalence and antimicrobial susceptibility profile of micoorganisms isolated from lower respiratory tract infections in hospitalized patients of Belo Horizonte - Minas Gerais/Brazil. Clinical Chemistry. 2015;61(10):S145.

315. Whitley V, Kircher S, Gill T, Hindler JA, O'Rourke S, Cooper C, et al. Multicenter Evaluation of the BD Phoenix CPO Detect Test for Detection and Classification of Carbapenemase-Producing Organisms in Clinical Isolates. Journal of Clinical Microbiology. 2020;58(5).

316. Samonis G, Maraki S, Rafailidis PI, Kapaskelis A, Kastoris AC, Falagas ME. Antimicrobial susceptibility of Gram-negative nonurinary bacteria to fosfomycin and other antimicrobials. Future Microbiology. 2010;5(6):961-70.

317. Genteluci GL, de Souza PA, Cardoso Gomes DB, Sousa VS, de Souza MJ, Lannes Abib JR, et al. Polymyxin B Heteroresistance and Adaptive Resistance in Multidrug- and Extremely Drug-Resistant Acinetobacter baumannii. Current Microbiology. 2020;77(9):2300-6.

318. Naeimi Mazraeh F, Hasani A, Sadeghi J, Samadi Kafil H, Soroush Barhaghi MH, Yeganeh Sefidan F, et al. High frequency of blaPER-1 gene in clinical strains of Acinetobacter baumannii and its association with quorum sensing and virulence factors. Gene Reports. 2021;24.

319. Afshar ZM, Asadi S, Miladi R, Danesh C, Farshid S, Asadi E, et al. Molecular Analysis of Oxacillinase Genes and Identification of Drug Resistance Pattern in MDR Strains of Acinetobacter baumannii Isolated from Burn Wound Samples in Kermanshah, Iran. Journal of Clinical and Diagnostic Research. 2022;16(5):14-8.

320. Chakraborty M, ira, Sardar S, De R, Biswas M, Mascellino MT, et al. Current Trends in Antimicrobial Resistance Patterns in Bacterial Pathogens among Adult and Pediatric Patients in the Intensive Care Unit in a Tertiary Care Hospital in Kolkata, India. Antibiotics-Basel. 2023;12(3).

321. Lin H-R, Hu A, Lai M-J, Chiang C-W, Liao C-C, Chang K-C. Rapid and sensitive detection of carbapenemase activity in Acinetobacter baumannii using superficially porous liquid chromatography-tandem mass spectrometry. Journal of Microbiology Immunology and Infection. 2016;49(6):910-7.

322. Tran GM, Ho-Le TP, Ha DT, Tran-Nguyen CH, Nguyen TSM, Pham TTN, et al. Patterns of antimicrobial resistance in intensive care unit patients: a study in Vietnam. Bmc Infectious Diseases. 2017;17.

323. Armalyte J, Jurenas D, Krasauskas R, Cepauskas A, Suziedeliene E. The higBA Toxin-Antitoxin Module From the Opportunistic Pathogen Acinetobacter baumannii - Regulation, Activity, and Evolution. Frontiers in Microbiology. 2018;9.

324. Zhang T, Xu X, Xu C-F, Bilya SR, Xu W. Mechanical ventilation-associated pneumonia caused by Acinetobacter baumannii in Northeast China region: analysis of genotype and drug resistance of bacteria and patients' clinical features over 7 years. Antimicrobial Resistance and Infection Control. 2021;10(1).

325. Selim S, Faried OA, Almuhayawi MS, Mohammed OA, Saleh FM, Warrad M. Dynamic Gene Clusters Mediating Carbapenem-Resistant Acinetobacter baumannii Clinical Isolates. Antibiotics-Basel. 2022;11(2).

326. Li Z, Ding Z, Liu Y, Jin X, Xie J, Li T, et al. Phenotypic and Genotypic Characteristics of Biofilm Formation in Clinical Isolates of Acinetobacter baumannii. Infection and Drug Resistance. 2021;14:2613-24.

327. Anwar M, Ejaz H, Zafar A, Hamid H. Phenotypic Detection of Metallo-Beta-Lactamases in Carbapenem Resistant Acinetobacter baumannii Isolated from Pediatric Patients in Pakistan. Journal of pathogens. 2016;2016:8603964-.

328. Park YK, Jung S-I, Park K-H, Kim DH, Choi JY, Kim SH, Ko KS. Changes in antimicrobial susceptibility and major clones of Acinetobacter calcoaceticus-baumannii complex isolates from a single hospital in Korea over 7 years. Journal of Medical Microbiology. 2012;61(1):71-9.

329. Sieniawski K, Kaczka K, Rucinska M, Gagis L, Pomorski L. Acinetobacter baumannii nosocomial infections. Polski przeglad chirurgiczny. 2013;85(9):483-90.

330. Vranic-Ladavac M, Bedenic B, Min, ri F, Istok M, Bosnjak Z, et al. Carbapenem resistance and acquired class D beta-lactamases in Acinetobacter baumannii from Croatia 2009-2010. European Journal of Clinical Microbiology & Infectious Diseases. 2014;33(3):471-8.

331. Asgin N, Otlu B, Cakmakliogullari EK, Celik B. High prevalence of TEM, VIM, and OXA-2 beta-lactamases and clonal diversity among Acinetobacter baumannii isolates in Turkey. Journal of Infection in Developing Countries. 2019;13(9):794-801.

332. Japoni-Nejad A, Mood EH, Ehsani P, Sardari S, Heravi FS, Bouzari S, Shahrokhi N. Identification and characterization of the type II toxin-antitoxin systems in the carbapenem-resistant Acinetobacter baumannii. Microbial Pathogenesis. 2021;158.

333. Ababneh MA, Al Domi M, Rababa'h AM. Surveillance study of bloodstream infections, antimicrobial use, and resistance patterns among intensive care unit patients: A retrospective cross-sectional study. International journal of critical illness and injury science. 2022;12(2):82-90.

334. Al-Dabaibah N, Obeidat NM, Shehabi AA. Epidemiology features of Acinetobacter baumannii colonizing respiratory tracts of ICU patients. International Arabic Journal of Antimicrobial Agents. 2012;2(2).

335. Zhu L-J, Chen X-Y, Hou P-F. Mutation of CarO participates in drug resistance in imipenem-resistant Acinetobacter baumannii. Journal of Clinical Laboratory Analysis. 2019;33(8).

336. Konca C, Tekin M, Geyik M. Susceptibility Patterns of Multidrug-Resistant Acinetobacter baumannii. Indian Journal of Pediatrics. 2021;88(2):120-6.

337. Novovic K, Nedeljkovic SK, Poledica M, Nikolic G, Grujic B, Jovcic B, et al. Virulence potential of multidrug-resistant Acinetobacter baumannii isolates from COVID-19 patients on mechanical ventilation: The first report from Serbia. Frontiers in Microbiology. 2023;14.

338. Norozi B, Farahani A, Mohajeri P, Davoodabadi A. Molecular epidemiology of hospital acquired OXA-carbapenemase-producing Acinetobacter baumannii in Western Iran. Asian Pacific Journal of Tropical Disease. 2014;4:S803-S7.

339. Gundeslioglu OO, Gokmen TG, Horoz OO, Aksaray N, Koksal F, Yaman A, et al. Molecular epidemiology and antibiotic susceptibility pattern of Acinetobacter baumannii isolated from children in a Turkish university hospital. Turkish Journal of Pediatrics. 2014;56(4):360-7.

340. Jimenez-Guerra G, Heras-Canas V, Gutierrez-Soto M, del Pilar Aznarte-Padial M, Exposito-Ruiz M, Maria Navarro-Mari J, et al. Urinary tract infection by Acinetobacter baumannii and Pseudomonas aeruginosa: evolution of antimicrobial resistance and therapeutic alternatives. Journal of Medical Microbiology. 2018;67(6):790-7.

341. Sohrabi N, Farajnia S, Akhi MT, Nahaei MR, Naghili B, Peymani A, et al. Prevalence of OXA-Type beta-Lactamases Among Acinetobacter baumannii Isolates from Northwest of Iran. Microbial Drug Resistance. 2012;18(4):385-9.

342. Xi J, Jia P, Zhu Y, Yu W, Zhang J, Gao H, et al. Antimicrobial susceptibility to polymyxin B and other comparators against Gram-negative bacteria isolated from bloodstream infections in China: Results from CARVIS-NET program. Frontiers in Microbiology. 2022;13.

343. Pourabdollah M, Askari E, Mansoury L, Mansoury H. Prevalence of carbapenem-resistant gram-negative bacilli producing carbapenemase by modified carbapenem inactivation method in an educational hospital in Tehran. Immunopathologia Persa. 2023;9(1).

344. Kock MM, Bellomo AN, Storm N, Ehlers MM. Prevalence of carbapenem resistance genes in Acinetobacter baumannii isolated from clinical specimens obtained from an academic hospital in South Africa. Southern African Journal of Epidemiology and Infection. 2013;28(1):28-32.

345. Azizi O, Shakibaie MR, Modarresi F, Shahcheraghi F. Molecular Detection of Class-D OXA Carbapenemase Genes in Biofilm and Non-Biofilm Forming Clinical Isolates of Acinetobacter baumannii. Jundishapur Journal of Microbiology. 2015;8(1).

346. Ari H, Aydoğan O, Demirci M, Köksal Çakirlar F. Distribution of AdeABC efflux system genes in Acinetobacter baumannii isolated from blood cultures of hospitalized patients and their relationship with carbapenem and aminoglycoside resistance. Mediterranean Journal of Infection, Microbes and Antimicrobials. 2019;8.

347. Brink A, Moolman J, da Silva MC, Botha M, Natl Antibiotic Surveillance F. Antimicrobial susceptibility profile of selected bacteraemic pathogens from private institutions in South Africa. Samj South African Medical Journal. 2007;97(4):273-9.

348. Gholami M, Hashemi A, Hakemi-Vala M, Goudarzi H, Hallajzadeh M. Efflux Pump Inhibitor Phenylalanine-Arginine B-Naphthylamide Effect on the Minimum Inhibitory Concentration of Imipenem in Acinetobacter baumannii Strains Isolated From Hospitalized Patients in Shahid Motahari Burn Hospital, Tehran, Iran. Jundishapur Journal of Microbiology. 2015;8(10).

349. Agarwal S, Kakati B, Kh, uri S, Gupta S. Emergence of Carbapenem Resistant Non-Fermenting Gram-Negative Bacilli Isolated in an ICU of a Tertiary Care Hospital. Journal of clinical and diagnostic research : JCDR. 2017;11(1):DC04-DC7.

350. Pan S, Huang X, Wang Y, Li L, Zhao C, Yao Z, et al. Efficacy of intravenous plus intrathecal/intracerebral ventricle injection of polymyxin B for post-neurosurgical intracranial infections due to MDR/XDR Acinectobacter baumannii: a retrospective cohort study. Antimicrobial Resistance and Infection Control. 2018;7.

351. Azizi O, Fereshteh S, Nasiri O, Ghorbani M, Barzi SM, Badmasti F. The Occurrence and Characterization of Class I, II, and III Integrons Among Carbapenemase-Producing Clinical Strains of Acinetobacter baumannii in Tehran, Iran. Jundishapur Journal of Microbiology. 2021;14(6).

352. Mumcuoglu I, Caglar H, Erdem D, Aypak A, Gun P, Kursun S, et al. Secondary bacterial infections of the respiratory tract in COVID-19 patients. Journal of Infection in Developing Countries. 2022;16(7):1131-7.

353. Wareham DW, Momin MHFA, Phee LM, Hornsey M, St, ing JF. Cefepime/sulbactam as an enhanced antimicrobial combination therapy for the treatment of MDR Gram-negative infections. Journal of Antimicrobial Chemotherapy. 2020;75(1):135-9.

354. An NV, Hoang LH, Le HHL, Son NT, Hong LT, Viet TT, et al. Distribution and Antibiotic Resistance Characteristics of Bacteria Isolated from Blood Culture in a Teaching Hospital in Vietnam During 2014-2021. Infection and Drug Resistance. 2023;16:1677-92.

355. Peleg AY, Franklin C, Bell JM, Spelman DW. Emergence of carbapenem resistance in Acinetobacter baumannii recovered from blood cultures in Australia. Infection Control and Hospital Epidemiology. 2006;27(7):759-61.

356. Trang Dinh V, Quynh-Dao D, Phu Dinh V, Trung Vu N, Ca Van P, Trinh Tuyet D, et al. Antibiotic susceptibility and molecular epidemiology of Acinetobacter calcoaceticus-baurnannii complex strains isolated from a referral hospital in northern Vietnam. Journal of Global Antimicrobial Resistance. 2014;2(4):318-21.

357. Ramette A, Kronenberg A, Swiss Ctr Antibiotic Resistance AN. Prevalence of carbapenem-resistant Acinetobacter baumannii from 2005 to 2016 in Switzerland. Bmc Infectious Diseases. 2018;18.

358. Aljindan R, Elhadi N. Genetic Relationship of Multi-Resistant Acinetobacter baumannii Isolates in Kingdom of Saudi Arabia. Journal of Pure and Applied Microbiology. 2018;12(4):1951-8.

359. Nigro SJ, Hall RM. Does the intrinsic oxaAb (bla(OXA-51-like)) gene of Acinetobacter baumannii confer resistance to carbapenems when activated by ISAba1? Journal of Antimicrobial Chemotherapy. 2018;73(12):3518-20.

360. Massik A, Hibaoui L, Arhoune B, Yahyaoui G, Oumokhtar B, Mahmoud M. Detection of metallo-beta lactamases and oxacillinase genes in carbapenem-resistant Acinetobacter baumannii strains isolated in Morocco. The Pan African medical journal. 2021;40:210-.

361. Jin X, Zhang H, Wu S, Qin X, Jia P, Tenover FC, et al. Multicenter Evaluation of Xpert Carba-R Assay for Detection and Identification of the Carbapenemase Genes in Rectal Swabs and Clinical Isolates. Journal of Molecular Diagnostics. 2021;23(1):111-9.

362. Elnasser Z, Elsamarneh R, Obeidat H, Amarin Z, Jaradat S, Kaplan N. In-vitro activity of tigecycline against multidrug-resistant Gram negative bacteria: The experience of a university hospital. Journal of Infection and Public Health. 2021;14(4):478-83.

363. Depka D, Mikucka A, Bogiel T, Rzepka M, Zawadka P, Gospodarek-Komkowska E. Conventional and Real-Time PCR Targeting bla(OXA) Genes as Reliable Methods for a Rapid Detection of Carbapenem-Resistant Acinetobacter baumannii Clinical Strains. Antibiotics-Basel. 2022;11(4).

364. Martins HSI, Bomfim MRQ, Franca RO, Farias LM, Carvalho MAR, Serufo JC, Santos SG. Resistance Markers and Genetic Diversity in Acinetobacter baumannii Strains Recovered from Nosocomial Bloodstream Infections. International Journal of Environmental Research and Public Health. 2014;11(2):1465-78.

365. El-Sokkary R, Uysal S, Erdem H, Kullar R, Pekok AU, Amer F, et al. Profiles of multidrug-resistant organisms among patients with bacteremia in intensive care units: an international ID-IRI survey. European Journal of Clinical Microbiology and Infectious Diseases. 2021;40(11):2323-34.

366. Rahman A, Styczynski A, Khaleque A, Hossain SA, Sadique A, Hossain A, et al. Genomic landscape of prominent XDR Acinetobacter clonal complexes from Dhaka, Bangladesh. BMC Genomics. 2022;23(1).

367. Feizabadi MM, Fathollahzadeh B, Taherikalani M, Rasoolinejad M, Sadeghifard N, Aligholi M, et al. Antimicrobial susceptibility patterns and distribution of bla(OXA) genes among Acinetobacter spp. isolated from patients at Tehran hospitals. Japanese Journal of Infectious Diseases. 2008;61(4):274-8.

368. Peymani A, Nahaei M-R, Farajnia S, Hasani A, Mirsalehian A, Sohrabi N, Abbasi L. High Prevalence of Metallo-beta-Lactamase-Producing Acinetobacter baumannii in a Teaching Hospital in Tabriz, Iran. Japanese Journal of Infectious Diseases. 2011;64(1):69-71.

369. Viana GF, dos Santos Saalfeld SM, Brondani Moreira RR, Menegucci TC, Garcia LB, Cardoso CL, et al. Can ampicillin/sulbactam resistance in Acinetobacter baumannii be predicted accurately by disk diffusion? Journal of Global Antimicrobial Resistance. 2013;1(4):221-3.

370. Slavcovici A, Maier C, Radulescu A. ANTIMICROBIAL RESISTANCE OF ESKAPE-PATHOGENS IN CULTURE-POSITIVE PNEUMONIA. Farmacia. 2015;63(2):201-5.

371. Al-Hassan L, Zafer MM, El-Mahalla H. Multiple sequence types responsible for healthcare-associated Acinetobacter baumannii dissemination in a single centre in Egypt. Bmc Infectious Diseases. 2019;19(1).

372. Turner PJ, Greenhalgh JM, Grp MS. The activity of meropenem and comparators against Acinetobacter strains isolated from European hospitals, 1997-2000. Clinical Microbiology and Infection. 2003;9(6):563-7.

373. Cai Y, Li R, Liang B, Bai N, Liu Y, Wang R. In Vitro Antimicrobial Activity and Mutant Prevention Concentration of Colistin against Acinetobacter baumannii. Antimicrobial Agents and Chemotherapy. 2010;54(9):3998-9.

374. Dally S, Lemuth K, Kaase M, Rupp S, Knabbe C, Weile J. DNA Microarray for Genotyping Antibiotic Resistance Determinants in Acinetobacter baumannii Clinical Isolates. Antimicrobial Agents and Chemotherapy. 2013;57(10):4761-8.

375. Bocanegra-Ibarias P, Pena-Lopez C, Camacho-Ortiz A, Llaca-Diaz J, Silva-Sanchez J, Barrios H, et al. Genetic characterisation of drug resistance and clonal dynamics of Acinetobacter baumannii in a hospital setting in Mexico. International Journal of Antimicrobial Agents. 2015;45(3):309-13.

376. Kara SS, Polat M, Tapisiz A, Kalkan G, Simsek H, Tezer H. Ventilator associated pneumonia due to carbapenem resistant microorganisms in children. Minerva Pediatrica. 2019;71(4):349-57.

377. Pei F, Chu J, Liu Y, Wang L, Zhang F, Ji M, et al. A Surveillance of Antimicrobial Resistance in a University-affiliated Hospital in North China in 2012. Journal of Pure and Applied Microbiology. 2013;7(4):3077-83.

378. Chen H, Wang Z, Li H, Wang Q, Zhao C, He W, et al. In Vitro Analysis of Activities of 16 Antimicrobial Agents against Gram-Negative Bacteria from Six Teaching Hospitals in China. Japanese Journal of Infectious Diseases. 2015;68(4):263-7.

379. Yang Y, Wei L, Zou F, Liu G, Wei C, Wu L, et al. Surveillance of antibiotic resistance in bacterial isolates from gansu provincial hospital in 2013. Chinese Journal of Infection and Chemotherapy. 2015;15(4):335-40.

380. Nojookambari NY, Sadredinamin M, Dehbanipour R, Ghalav, Z, Eslami G, et al. Prevalence of beta-lactamase-encoding genes and molecular typing of Acinetobacter baumannii isolates carrying carbapenemase OXA-24 in children. Annals of Clinical Microbiology and Antimicrobials. 2021;20(1).

381. Gogou V, Pournaras S, Giannouli M, Voulgari E, Piperaki E-T, Zarrilli R, Tsakris A. Evolution of multidrug-resistant Acinetobacter baumannii clonal lineages: a 10 year study in Greece (2000-09). Journal of Antimicrobial Chemotherapy. 2011;66(12):2767-72.

382. Tawfeeq HR, Rasheed MN, Hassan RH, Musleh MH, Nader MI. MOLECULAR DETECTION OF BLAOXA GENES IN ACINETOBACTER BAUMANNII COLLECTED FROM PATIENTS WITH VARIOUS INFECTIONS. Biochemical and Cellular Archives. 2020;20(1):1233-9.

383. Li J, Wang J, Yang Y, Cai P, Cao J, Cai X, Zhang Y. Etiology and antimicrobial resistance of secondary bacterial infections in patients hospitalized with COVID-19 in Wuhan, China: a retrospective analysis. Antimicrobial Resistance and Infection Control. 2020;9(1).

384. Lasarte-Monterrubio C, Vazquez-Ucha JC, Maneiro M, Arca-Suarez J, Alonso I, Guijarro-Sanchez P, et al. Activity of Imipenem, Meropenem, Cefepime, and Sulbactam in Combination with the beta-Lactamase Inhibitor LN-1-255 against Acinetobacter spp. Antibiotics-Basel. 2021;10(2).

385. Bian X, Liu X, Zhang X, Li X, Zhang J, Zheng H, et al. Epidemiological and genomic characteristics of Acinetobacter baumannii from different infection sites using comparative genomics. Bmc Genomics. 2021;22(1).

386. Shabazi S, Shivaee A, Nasiri M, Mirshekar M, Sabzi S, Saria OK. Zinc oxide nanoparticles impact the expression of the genes involved in toxin–antitoxin systems in multidrug-resistant Acinetobacter baumannii. Journal of Basic Microbiology. 2022.

387. Hou PF, Chen XY, Yan GF, Wang YP, Ying CM. Study of the Correlation of Imipenem Resistance with Efflux Pumps AdeABC, AdelJK, AdeDE and AbeM in Clinical Isolates of Acinetobacter baumannii. Chemotherapy. 2012;58(2):152-8.

388. Cerezales M, Xanthopoulou K, Wille J, Bustamante Z, Seifert H, Gallego L, Higgins PG. Acinetobacter baumannii analysis by core genome multi-locus sequence typing in two hospitals in Bolivia: endemicity of international clone 7 isolates (CC25). International Journal of Antimicrobial Agents. 2019;53(6):844-9.

389. Karah N, Khalid F, Wai SN, Uhlin BE, Ahmad I. Molecular epidemiology and antimicrobial resistance features of Acinetobacter baumannii clinical isolates from Pakistan. Annals of Clinical Microbiology and Antimicrobials. 2020;19(1).

390. Banoub NG, Saleh SE, Helal HS, Aboshanab KM. Antibiotics Combinations and Chitosan Nanoparticles for Combating Multidrug Resistance Acinetobacter baumanni. Infection and Drug Resistance. 2021;14:3327-39.

391. Chen T, Fu Y, Hua X, Xu Q, Lan P, Jiang Y, et al. Acinetobacter baumannii strains isolated from cerebrospinal fluid (CSF) and bloodstream analysed by cgMLST: the dominance of clonal complex CC92 in CSF infections. International Journal of Antimicrobial Agents. 2021;58(4).

392. Ashuthosh KC, Hegde A, Rao P, Manipura R. Multidrug-Resistant Acinetobacter baumannii - The Modern Menace: A Retrospective Study in a Tertiary Hospital in Mangalore. Infection and Drug Resistance. 2020;13:2181-7.

393. Bankan N, Koka F, Vijayaraghavan R, Basireddy SR, Jayaraman S. Overexpression of the adeb efflux pump gene in tigecycline-resistant acinetobacter baumannii clinical isolates and its inhibition by (+)usnic acid as an adjuvant. Antibiotics. 2021;10(9).

394. Karthika RU, Rao RS, Sahoo S, Shashikala P, Kanungo R, Jayach, et al. Phenotypic and genotypic assays for detecting the prevalence of metallo-beta-lactamases in clinical isolates of Acinetobacter baumannii from a South Indian tertiary care hospital. Journal of Medical Microbiology. 2009;58(4):430-5.

395. Mostachio AK, Levin AS, Rizek C, Rossi F, Zerbini J, Costa SF. High prevalence of OXA-143 and alteration of outer membrane proteins in carbapenem-resistant Acinetobacter spp. isolates in Brazil. International Journal of Antimicrobial Agents. 2012;39(5):396-401.

396. Tada T, Miyoshi-Akiyama T, Shimada K, Shimojima M, Kirikae T. Dissemination of 16S rRNA Methylase ArmA-Producing Acinetobacter baumannii and Emergence of OXA-72 Carbapenemase Coproducers in Japan. Antimicrobial Agents and Chemotherapy. 2014;58(5):2916-20.

397. Girija SA, Priyadharsini JV, Paramasivam A. Prevalence of carbapenem-hydrolyzing OXA-type β-lactamases among Acinetobacter baumannii in patients with severe urinary tract infection. Acta Microbiol Immunol Hung. 2019;67(1):49-55.

398. Girija As S, Priyadharsini J V. CLSI based antibiogram profile and the detection of MDR and XDR strains of Acinetobacter baumannii isolated from urine samples. Medical journal of the Islamic Republic of Iran. 2019;33:3-.

399. Fatima A, Gohar H, Dawood K, Siddiqui HZ, Sajjad M, Naseem S. Bacteriological Profile and Antimicrobial Susceptibility Pattern of Pus Isolates from Tertiary Care Hospital. Journal of the Liaquat University of Medical and Health Sciences. 2022;21(3):190-5.

400. Santoso P, Sung M, Hartantri Y, Andriyoko B, Sugianli AK, Alisjahbana B, et al. MDR Pathogens Organisms as Risk Factor of Mortality in Secondary Pulmonary Bacterial Infections Among COVID-19 Patients: Observational Studies in Two Referral Hospitals in West Java, Indonesia. International Journal of General Medicine. 2022;15:4741-51.

401. Shayea RH, Ali MR. ERIC-PCR Genotyping and Clonal Genetic linkage Between Carbapenem-Resistant Acinetobacter baumannii Isolates. Jordan Journal of Biological Sciences. 2022;15(4):689-96.

402. Ceyhan-Guvensen N, Keskin D, Sankur F. ANTIBIOTIC RESISTANCE RATIO OF ACINETOBACTER BAUMANNII AGAINST TO TEN ANTIBIOTICS AND MULTIDRUG RESISTANCE INDEX. Fresenius Environmental Bulletin. 2017;26(12):701-5.

403. Tarafdar F, Jafari B, Azimi T. Evaluating the antimicrobial resistance patterns and molecular frequency of bla(oxa-48) and bla(GES-2) genes in Pseudomonas aeruginosa and Acinetobacter baumannii strains isolated from burn wound infection in Tehran, Iran. New Microbes and New Infections. 2020;37:100686-Article No.: .

404. Kim C-K, Lee Y, Lee H, Woo G-J, Song W, Kim M-N, et al. Prevalence and diversity of carbapenemases among imipenem-nonsusceptible Acinetobacter isolates in Korea. emergence of a novel OXA-182. Diagnostic Microbiology and Infectious Disease. 2010;68(4):432-8.

405. Abdalhamid B, Hassan H, Itbaileh A, Shorman M. Characterization of carbapenem-resistant Acinetobacter baumannii clinical isolates in a tertiary care hospital in Saudi Arabia. New Microbiologica. 2014;37(1):65-73.

406. Khalili H, Shojaei L, Mohammadi M, Beigmohammadi M-T, Abdollahi A, Doomanlou M. Meropenem/colistin versus meropenem/ampicillin-sulbactam in the treatment of carbapenem-resistant pneumonia. Journal of Comparative Effectiveness Research. 2018;7(9):901-11.

407. Dehbalaei MA, Najar-Peerayeh S, Behmanesh M, Taherikalani M. Polyclonal Distribution of blaOXA-23 Gene Among Acinetobacter baumannii Isolated from Intensive Care Unit Patients in Tehran; Pulsed-Field Gel Electrophoresis Analysis. Jundishapur Journal of Microbiology. 2018;11(1).

408. Valadan Tahbaz S, Azimi L, Asadian M, Lari AR. Evaluation of synergistic effect of tazobactam with meropenem and ciprofloxacin against multi-drug resistant Acinetobacter baumannii isolated from burn patients in Tehran. GMS hygiene and infection control. 2019;14:Doc08-Doc.

409. Noyal MJC, Menezes GA, Harish BN, Sujatha S, Parija SC. Simple screening tests for detection of carbapenemases in clinical isolates of nonfermentative Gram-negative bacteria. Indian Journal of Medical Research. 2009;129(6):707-12.

410. Long YB, Faoagali J, Bodman J, George N, McKay D, Katouli M. Persistence of Multiple Antibiotic Resistant Strains of Acinetobacter baumannii Carrying Class 1 Integron in a Hospital Setting. Microbial Drug Resistance. 2009;15(3):167-72.

411. Fang F, Wang S, Dang YX, Wang X, Yu GQ. Molecular characterization of carbapenemase genes in Acinetobacter baumannii in China. Genetics and Molecular Research. 2016;15(1).

412. Chen Q, Zhou J-w, Fan J-z, Wu S-h, Xu L-h, Jiang Y, et al. Simultaneous emergence and rapid spread of three OXA-23 producing Acinetobacter baumannii ST208 strains in intensive care units confirmed by whole genome sequencing. Infection Genetics and Evolution. 2018;58:243-50.

413. Nazir A. Multidrug-resistant Acinetobacter septicemia in neonates: A study from a teaching hospital of Northern India. Journal of laboratory physicians. 2019;11(1):23-8.

414. Huang Y, Qian M, Zhang S, Xu J, Li H, Chen Y, Zhou Q. ANALYSIS OF DRUG RESISTANCE OF CARBAPENEM-RESISTANT ACINETOBACTER BAUMANNII IN CHILDREN AND ITS RISK FACTORS. Acta Medica Mediterranea. 2019;35(6):3417-22.

415. Thao Nguyen V, Byun J-H, D'Souza R, Pinto NA, Le Phuong N, Yong D, Chong Y. Adjustment of Modified Carbapenem Inactivation Method Conditions for Rapid Detection of Carbapenemase-Producing Acinetobacter baumannii. Annals of Laboratory Medicine. 2020;40(1):21-6.

416. Rusul HS, Suhad SM. FIRST REPORT IN IRAQ: AMINO ACID SUBSTITUTION IN PMRCAB GENES AND THERE CORELLATION WITH COLISTIN RESISTANCE AMONG A.BAUMANNII ISOLATES. Iraqi Journal of Agricultural Sciences. 2022;53(2):237-51.

417. Ma Z, Zhou LQ, Wang H, Luo LP. Investigations on the genomic diversity of OXA from isolated Acinetobacter baumannii. Genetics and Molecular Research. 2015;14(4):14711-6.

418. Han L, Lei J, Xu J, Han S. bla(OXA-23-like) and bla(TEM) rather than bla(OXA-51-like) contributed to a high level of carbapenem resistance in Acinetobacter baumannii strains from a teaching hospital in Xi'an, China. Medicine. 2017;96(48).

419. Babaie Z, Delfani S, Rezaei F, Norolahi F, Mahdian S, Shakib P. Molecular Detection of Carbapenem Resistance in Acinetobacter Baumannii Isolated From Patients in Khorramabad City, Iran. Infectious Disorders - Drug Targets. 2020;20(4):543-9.

420. Namaei MH, Yousefi M, Askari P, Roshanravan B, Hashemi A, Rezaei Y. High prevalence of multidrug-resistant non-fermentative Gram-negative bacilli harboring blaIMP-1 and blaVIM-1 metallo-beta-lactamase genes in Birjand, south-east Iran. Iranian journal of microbiology. 2021;13(4):470-9.

421. Ahmed NJ, Haseeb A, Mahmoud S, Khan AH. Emergence of High Drug Resistant Bacterial Isolates in Al-Kharj. Journal of Young Pharmacists. 2021;13(4):386-91.

422. ar WP, Saw S, Kumar AMV, Camara BS, Sein MM. Wounds, antimicrobial resistance and challenges of implementing a surveillance system in myanmar: A mixed-methods study. Tropical Medicine and Infectious Disease. 2021;6(2).

423. Farzana R, Swedberg G, Giske CG, Hasan B. Molecular and genetic characterization of emerging carbapenemase-producing Acinetobacter baumannii strains from patients and hospital environments in Bangladesh. Infection prevention in practice. 2022;4(2):100215-.

424. Al-Ouqaili MTS, Jaloot AS, Badawy AS. Identification of an OprD and blaIMP Gene-mediated Carbapenem Resistance in Acinetobacter baumannii and Pseudomonas aeruginosa among Patients with Wound Infections in Iraq. Asian Journal of Pharmaceutics. 2018;12(3):S959-S65.

425. Al-Hassan LL, Al-Madboly LA. Molecular characterisation of an Acinetobacter baumannii outbreak. Infection prevention in practice. 2020;2(2):100040-.

426. Qiu Y, Yang J, Chen Y, Yang J, Zhu Q, Zhu C, et al. Microbiological profiles and antimicrobial resistance patterns of pediatric bloodstream pathogens in China, 2016-2018. European Journal of Clinical Microbiology & Infectious Diseases. 2021;40(4):739-49.

427. Zarrilli R, Crispino M, Bagattini M, Barretta E, Di Popolo A, Triassi M, Villari P. Molecular epidemiology of sequential outbreaks of Acinetobacter baumannii in an intensive care unit shows the emergence of carbapenem resistance. Journal of Clinical Microbiology. 2004;42(3):946-53.

428. Khodier AA, Saafan A, Bakeer W, Khairalla AS. Molecular Characterization of Multiple Antibiotic-Resistant Acinetobacter baumannii Isolated from Egyptian Patients. Journal of Pure and Applied Microbiology. 2020;14(4):2399-405.

429. Golli A-L, Cristea OM, Zlatian O, Glodeanu A-D, Balasoiu AT, Ionescu M, Popa S. Prevalence of Multidrug-Resistant Pathogens Causing Bloodstream Infections in an Intensive Care Unit. Infection and Drug Resistance. 2022;15:5981-92.

430. Forde BM, Bergh H, Cuddihy T, Hajkowicz K, Hurst T, Playford EG, et al. Clinical implementation of routine whole-genome sequencing for hospital infection control of multi-drug resistant pathogens. 2022.

431. Mahajan G, Sheemar S, Chopra S, Kaur J, Chowdhary D, Makhija SK. Carbapenem resistance and phenotypic detection of carbapenemases in clinical isolates of acinetobacter baumannii. Indian Journal of Medical Sciences. 2010;65(1):18-25.

432. Lean S-S, Suhaili Z, Ismail S, Rahman NIA, Othman N, Abdullah FH, et al. Prevalence and Genetic Characterization of Carbapenem- and Polymyxin-Resistant Acinetobacter baumannii Isolated from a Tertiary Hospital in Terengganu, Malaysia. ISRN microbiology. 2014;2014:953417-.

433. Jajoo M, Manch, a V, Chaurasia S, Sankar MJ, Gautam H, et al. Alarming rates of antimicrobial resistance and fungal sepsis in outborn neonates in North India. Plos One. 2018;13(6).

434. Shahari AS, Palanisamy NK, Rustam FRM, Zain ZM, Kiong BLP, Soh TST, Nor FM. Emergence of class D β-lactamase (blaOXA-23 and blaOXA-24) genes among multidrug-resistant Acinetobacter baumannii isolated from a tertiary hospital in Malaysia. Malaysian Journal of Pathology. 2019;41(3):406.

435. Zhang Y, Du M, Johnston JM, Andres EB, Suo J, Yao H, et al. Estimating length of stay and inpatient charges attributable to hospital-acquired bloodstream infections. Antimicrobial Resistance and Infection Control. 2020;9(1).

436. Kar M, Dubey A, Singh R, Sahu C, Patel SS, Fatima N. Acinetobacter Meningitis: A Retrospective Study on its Incidence and Mortality Rates in Postoperative Patients at a Tertiary Care Centre in Northern India. Journal of Clinical and Diagnostic Research. 2023;17(1):DC01-DC6.

437. Jean SS, Hsueh PR, Lee WS, Chang HT, Chou MY, Chen IS, et al. Nationwide surveillance of antimicrobial resistance among non-fermentative Gram-negative bacteria in Intensive Care Units in Taiwan: SMART programme data 2005. International Journal of Antimicrobial Agents. 2009;33(3):266-71.

438. Al-Sweih NA, Al-Hubail M, Rotimi VO. Three distinct clones of carbapenem-resistant Acinetobacter baumannii with high diversity of carbapenemases isolated from patients in two hospitals in Kuwait. Journal of infection and public health. 2012;5(1):102-8.

439. Jara MC, Frediani AV, Zehetmeyer FK, Bruhn FRP, Müller MR, Miller RG, Nascente PDS. Multidrug-Resistant Hospital Bacteria: Epidemiological Factors and Susceptibility Profile. Microbial Drug Resistance. 2021;27(3):433-40.

440. Maleki A, Kaviar VH, Koupaei M, Haddadi MH, Kalani BS, Valadbeigi H, et al. Molecular typing and antibiotic resistance patterns among clinical isolates of Acinetobacter baumannii recovered from burn patients in Tehran, Iran. Frontiers in Microbiology. 2022;13.

441. Ergonul O, Tokca G, Keske Ş, Donmez E, Madran B, Kömür A, et al. Elimination of healthcare-associated Acinetobacter baumannii infection in a highly endemic region. International Journal of Infectious Diseases. 2022;114:11-4.

442. Dong S-X, Wang J-T, Chang S-C. Activities of doripenem against nosocomial bacteremic drug-resistant Gram-negative bacteria in a medical center in Taiwan. Journal of Microbiology Immunology and Infection. 2012;45(6):459-64.

443. Ghajav, H, Esfahani BN, Havaei SA, Moghim S, Fazeli H. Molecular identification of Acinetobacter baumannii isolated from intensive care units and their antimicrobial resistance patterns. Advanced biomedical research. 2015;4:110-.

444. Tolba STM, El-Shatoury EH, Abo-Elnasr NM. Prevalence of Carbapenem Resistant Acinetobacter baumannii (CRAB) in some Egyptian Hospitals: Evaluation of the Use of bla(OXA-51-like) Gene as Species Specific Marker for CRAB. Egyptian Journal of Botany. 2019;59(3):723-33.

445. Adjei AY, Vasaikar S, D. e, Apalata T, Okuthe EG, Songca S, Phinda i. Phylogenetic analysis of carbapenem-resistant Acinetobacter baumannii isolated from different sources using Multilocus Sequence Typing Scheme. Infection Genetics and Evolution. 2021;96.

446. Al-Hassan L, Elbadawi H, Osman E, Ali S, Elhag K, Cantillon D, et al. Molecular Epidemiology of Carbapenem-Resistant Acinetobacter baumannii From Khartoum State, Sudan. Frontiers in Microbiology. 2021;12.

447. Donadu MG, Zanetti S, Nagy AL, Barrak I, Gajdacs M. Insights on carbapenem-resistant Acinetobacter baumannii: phenotypic characterization of relevant isolates. Acta Biologica Szegediensis. 2021;65(1):85-92.

448. Joseph NM, Sistla S, Dutta TK, Badhe AS, Rasitha D, Parija SC. Reliability of Kirby-Bauer disk diffusion method for detecting meropenem resistance among non-fermenting gram-negative bacilli. Indian Journal of Pathology and Microbiology. 2011;54(3):556-60.

449. Fouad M, Attia AS, Tawakkol WM, Hashem AM. Emergence of carbapenem-resistant Acinetobacter baumannii harboring the OXA-23 carbapenemase in intensive care units of Egyptian hospitals. International Journal of Infectious Diseases. 2013;17(12):E1252-E4.

450. Teresa Reguero M, Esther Medina O, Hern A, ez M, Vanessa Florez D, Maria Valenzuela E, Ramon Mantilla J. Antibiotic resistance patterns of Acinetobacter calcoaceticus-A. baumannii complex species from Colombian hospitals. Enfermedades Infecciosas Y Microbiologia Clinica. 2013;31(3):142-6.

451. Li YJ, Pan CZ, Zhao ZW, Zhao ZX, Chen HL, Lu WB. Effects of a combination of amlodipine and imipenem on 42 clinical isolates of Acinetobacter baumannii obtained from a teaching hospital in Guangzhou, China. Bmc Infectious Diseases. 2013;13.

452. Makke G, Bitar I, Salloum T, Panossian B, Alousi S, Arabaghian H, et al. Whole-Genome-Sequence-Based Characterization of Extensively Drug-Resistant Acinetobacter baumannii Hospital Outbreak. Msphere. 2020;5(1).

453. Wang J, Zhou M, Huang G, Guo Z, Sauser J, Metsini A, et al. Antimicrobial resistance in southern China: results of prospective surveillance in Dongguan city, 2017. Journal of Hospital Infection. 2020;105(2):188-96.

454. El-Badawy MF, Abou-Elazm FI, Omar MS, El-Naggar ME, Maghrabi IA. The first saudi study investigating the plasmid-borne aminoglycoside and sulfonamide resistance among acinetobacter baumannii clinical isolates genotyped by rapd-pcr: The declaration of a novel allelic variant called aac(6ʹ)-sl and three novel mutations in the sul1 gene in the acinetobacter plasmid (s). Infection and Drug Resistance. 2021;14:4739-56.

455. Alcantar-Curiel MD, Huerta-Cedeno M, Jarillo-Quijada MD, Gayosso-Vazquez C, Fern, ez-Vazquez JL, et al. Gram-negative ESKAPE bacteria bloodstream infections in patients during the COVID-19 pandemic. PeerJ. 2023;11:e15007-e.

456. Purohit M, Mendiratta DK, Deotale VS, Madhan M, Manoharan A, Narang P. Detection of metallo-beta-lactamases producing Acinetobacter baumannii using microbiological assay, disc synergy test and PCR. Indian Journal of Medical Microbiology. 2012;30(4):456-61.

457. Carvalhaes CG, Cayo R, Assis DM, Martins ER, Juliano L, Juliano MA, Gales AC. Detection of SPM-1-Producing Pseudomonas aeruginosa and Class D beta-Lactamase-Producing Acinetobacter baumannii Isolates by Use of Liquid Chromatography-Mass Spectrometry and Matrix-Assisted Laser Desorption Ionization-Time of Flight Mass Spectrometry. Journal of Clinical Microbiology. 2013;51(1):287-90.

458. Khan DM, Moosabba MS, Rao IV. Changing antibiogram profile of Acinetobacter baumannii in diabetic and non-diabetic foot ulcer infections. Journal of Clinical and Diagnostic Research. 2018;12(5):DC12-DC6.

459. Rahman M, Prasad KN, Gupta S, Singh S, Singh A, Pathak A, et al. Prevalence and Molecular Characterization of New Delhi Metallo-Beta-Lactamases in Multidrug-Resistant Pseudomonas aeruginosa and Acinetobacter baumannii from India. Microbial Drug Resistance. 2018;24(6):792-8.

460. Basatian-Tashkan B, Niakan M, Khaledi M, Afkhami H, Sameni F, Bakhti S, Mirnejad R. Antibiotic resistance assessment of Acinetobacter baumannii isolates from Tehran hospitals due to the presence of efflux pumps encoding genes (adeA and adeS genes) by molecular method. BMC research notes. 2020;13(1):543-.

461. Hazhirkamal M, Zarei O, Movahedi M, Karami P, Shokoohizadeh L, Taheri M. Molecular typing, biofilm production, and detection of carbapenemase genes in multidrug-resistant Acinetobacter baumannii isolated from different infection sites using ERIC-PCR in Hamadan, west of Iran. Bmc Pharmacology & Toxicology. 2021;22(1).

462. Mostafa SH, Saleh SE, Hamed SM, Aboshanab KM. Febrile illness of bacterial etiology in a public fever hospital in Egypt: High burden of multidrug resistance and WHO priority Gram negative pathogens. Germs. 2022;12(1):75-85.

463. Akin F, Yazar A, Dogan M. Determining the Infectious Pathogens and Their Resistance to Antibiotics in a Pediatric Intensive Care Unit. Journal of Pediatric Infectious Diseases. 2018;13(1):42-5.

464. Kondratiuk V, Jones BT, Kovalchuk V, Kovalenko I, Ganiuk V, Kondratiuk O, Frantsishko A. Phenotypic and genotypic characterization of antibiotic resistance in military hospital-associated bacteria from war injuries in the Eastern Ukraine conflict between 2014 and 2020. Journal of Hospital Infection. 2021;112:69-76.

465. Balkhair A, Saadi KA, Adawi BA. Epidemiology and mortality outcome of carbapenem- and colistin-resistant Klebsiella pneumoniae, Escherichia coli, Acinetobacter baumannii, and Pseudomonas aeruginosa bloodstream infections. IJID regions. 2023;7:1-5.

466. Park S, Kim H-S, Lee KM, Yoo JS, Yoo JI, Lee YS, Chung GT. Molecular and Epidemiological Characterization of Carbapenem-Resistant Acinetobacter baumannii in Non-Tertiary Korean Hospitals. Yonsei Medical Journal. 2013;54(1):177-82.

467. Petrova AP, Stanimirova ID, Ivanov IN, Petrov MM, Miteva-Katr, zhieva TM, et al. Carbapenemase Production of Clinical Isolates Acinetobacter baumannii and Pseudomonas aeruginosa from a Bulgarian University Hospital. Folia medica. 2017;59(4):413-22.

468. Girija SAS, Jayaseelan VP, Arumugam P. PREVALENCE OF VIM- AND GIM-PRODUCING ACINETOBACTER BAUMANNII FROM PATIENTS WITH SEVERE URINARY TRACT INFECTION. Acta Microbiologica Et Immunologica Hungarica. 2018;65(4):539-50.

469. Tellapragada C, Hasan B, Antonelli A, Maruri A, de Vogel C, Gijon D, et al. Isothermal microcalorimetry minimal inhibitory concentration testing in extensively drug resistant Gram-negative bacilli: a multicentre study. Clinical Microbiology and Infection. 2020;26(10).

470. Kumari M, Verma S, Venkatesh V, Gupta P, Tripathi P, Agarwal A, et al. Emergence of blaNDM-1 and blaVIM producing Gram-negative bacilli in ventilator-associated pneumonia at AMR Surveillance Regional Reference Laboratory in India. Plos One. 2021;16(9).

471. Kiffer CRV, Mendes C, Kuti JL, Nicolau DP. Pharmacodynamic comparisons of antimicrobials against nosocomial isolates of Escherichia coli, Klebsiella pneumoniae, Acinetobacter baumannii and Pseudomonas aeruginosa from the MYSTIC surveillance program: the OPTAMA Program, South America 2002. Diagnostic Microbiology and Infectious Disease. 2004;49(2):109-16.

472. Wang X, Qiao F, Yu R, Gao Y, Zong Z. Clonal diversity of Acinetobacter baumannii clinical isolates revealed by a snapshot study. Bmc Microbiology. 2013;13.

473. Kadhom HA, Ali MR. Epidemiological Molecular Analysis of Acinetobacter baumannii isolates using a multilocus sequencing typing and Global lineage. Bionatura. 2022;7(1).

474. Pourajam S, Kalantari E, Talebzadeh H, Mellali H, Sami R, Soltaninejad F, et al. Secondary Bacterial Infection and Clinical Characteristics in Patients With COVID-19 Admitted to Two Intensive Care Units of an Academic Hospital in Iran During the First Wave of the Pandemic. Frontiers in Cellular and Infection Microbiology. 2022;12.

475. Franolic-Kukina I, Bedenic B, Budimir A, Herljevic Z, Vranes J, Higgins PG. Clonal spread of carbapenem-resistant OXA-72-positive Acinetobacter baumannii in a Croatian university hospital. International Journal of Infectious Diseases. 2011;15(10):E706-E9.

476. Ozseven AG, Cetin ES, Aridogan BC, Ozseven L. In vitro synergistic activity of carbapenems in combination with other antimicrobial agents against multidrug-resistant Acinetobacter baumannii. African Journal of Microbiology Research. 2012;6(12):2985-92.

477. Yürüken Z, İşeri L, Ünaldi Ö, Durmaz R. Analysis of cross-transmission and antimicrobial resistance of Pseudomonas aeruginosa and Acinetobacter baumannii isolates causing nosocomial infection in an intensive care unit. Bir yoğun bakım ünitesinde hastane enfeksiyonuna neden olan Pseudomonas aeruginosa ve Acinetobacter baumannii izolatlarının çapraz taşınımı ve antimikrobiyal direncinin analizi. 2016;36(1):1-6.

478. Sadeghi-Haddad-Zavareh M, Jouybari H, Javanian M, Shokri M, Bayani M, Roushan MR, et al. Antimicrobial resistance pattern in ventilator - Associated pneumonia in an intensive care unit of Babol, northern Iran. Journal of Acute Disease. 2018;7(2):74-7.

479. Wasfi R, Rasslan F, Hassan SS, Ashour HM, Abd El-Rahman OA. Co-Existence of Carbapenemase-Encoding Genes in Acinetobacter baumannii from Cancer Patients. Infectious Diseases and Therapy. 2021;10(1):291-305.

480. Mohamed SER, Ahmed Z, Mubarak T, Mohamed S, Higazi H, Ali S. Detection of the blaVIM-2 Gene in Carbapenem-Resistant Acinetobacter baumannii Clinical Isolates in Sudan. International Journal of Biomedicine. 2022;12(4):636-9.

481. Gu Y, Zhang W, Lei J, Zhang L, Hou X, Tao J, et al. Molecular epidemiology and carbapenem resistance characteristics of Acinetobacter baumannii causing bloodstream infection from 2009 to 2018 in northwest China. Frontiers in Microbiology. 2022;13.

482. Bedenic B, Bratic V, Mihaljevic S, Lukic A, Vidovic K, Reiner K, et al. Multidrug-Resistant Bacteria in a COVID-19 Hospital in Zagreb. Pathogens. 2023;12(1).

483. Wei X-L, Zeng Q-L, Xie M, Bao Y. Pathogen Distribution, Drug Resistance Risk Factors, and Construction of Risk Prediction Model for Drug-Resistant Bacterial Infection in Hospitalized Patients at the Respiratory Department During the COVID-19 Pandemic. Infection and Drug Resistance. 2023;16:1107-21.

484. Huang J, Chen EZ, Qu HP, Mao EQ, Zhu ZG, Ni YX, et al. Sources of multidrug-resistant Acinetobacter baumannii and its role in respiratory tract colonization and nosocomial pneumonia in intensive care unit patients. Chin Med J (Engl). 2013;126(10):1826-31.

485. Akrami F, Shah, ashti EF, Yahyapour Y, Sadeghi M, Khafri S, et al. Integron types, gene cassettes and antimicrobial resistance profile of Acinetobacter baumannii isolated from BAL samples in Babol, north of Iran. Microbial Pathogenesis. 2017;109:35-8.

486. Cerezales M, Ocampo-Sosa AA, Alvarez Montes L, Diaz Rios C, Bustamante Z, Santos J, et al. High Prevalence of Extensively Drug-resistant Acinetobacter baumannii at a Children Hospital in Bolivia. Pediatric Infectious Disease Journal. 2018;37(11):1118-23.

487. Koca Ö. Antibiotic resistance profiles of endotracheal aspirates in intensive care unit patients. Annals of Clinical and Analytical Medicine. 2019;10(2):243-6.

488. Li TY, Chan YJ. Comparison of antibiotic resistance between A. baumannii and A. nosocomialis. Clinica Chimica Acta. 2019;493:S549.

489. Chhatwal P, Ebadi E, Schwab F, Ziesing S, Vonberg R-P, Simon N, et al. Epidemiology and infection control of carbapenem resistant Acinetobacter baumannii and Klebsiella pneumoniae at a German university hospital: a retrospective study of 5 years (2015-2019). Bmc Infectious Diseases. 2021;21(1).

490. Hsueh PR, Liu YC, Yang D, Yan JJ, Wu TL, Huang WK, et al. Multicenter surveillance of antimicrobial resistance of major bacterial pathogens in intensive care units in 2000 in Taiwan. Microbial Drug Resistance. 2001;7(4):373-82.

491. Sheng W-H, Wang J-T, Li S-Y, Lin Y-C, Cheng A, Chen Y-C, Chang S-C. Comparative in vitro antimicrobial susceptibilities and synergistic activities of antimicrobial combinations against carbapenem-resistant Acinetobacter species: Acinetobacter baumannii versus Acinetobacter genospecies 3 and 13TU. Diagnostic Microbiology and Infectious Disease. 2011;70(3):380-6.

492. Hakyemez IN, Kucukbayrak A, Tas T, Yikilgan AB, Akkaya A, Yasayacak A, Akdeniz H. Nosocomial Acinetobacter baumannii infections and changing Antibiotic Resistance. Pakistan Journal of Medical Sciences. 2013;29(5):1245-8.

493. Akhtar J, Saleem S, Shahzad N, Waheed A, Jameel I, Rasheed F, Jahan S. Prevalence of Metallo-beta-Lactamase IMP and VIM Producing Gram Negative Bacteria in Different Hospitals of Lahore, Pakistan. Pakistan Journal of Zoology. 2018;50(6):2343-9.

494. Fonseca EL, Caldart RV, Freitas F, S. a, Morgado SM, Rocha LT, et al. Emergence of extensively drug-resistant international clone IC-6 Acinetobacter baumannii carrying bla(OXA-72) and bla(CTX-M-115) in the Brazilian Amazon region. Journal of Global Antimicrobial Resistance. 2020;20:18-21.

495. Ozkul C, Hazirolan G. Oxacillinase Gene Distribution, Antibiotic Resistance, and Their Correlation with Biofilm Formation inAcinetobacter baumanniiBloodstream Isolates. Microbial Drug Resistance. 2021;27(5):637-46.

496. Jayanthi MK, Jatin VK, Firdose N, Manu G, Raj R, Ramu R. Evaluation of the Emerging Multidrug-Resistant Acinetobacter baumannii in Clinical Samples from Tertiary Care Hospitals in Mysore, India - a Cohort Study. Asian Journal of Pharmaceutics. 2021;15(3):372-3778.

497. Sung JY, Kwon KC, Park JW, Kim YS, Kim JM, Shin KS, et al. Dissemination of IMP-1 and OXA Type β-Lactamase in Carbapenem-resistant Acinetobacter baumannii. Korean Journal of Laboratory Medicine. 2008;28(1):16-23.

498. Cetin ES, Durmaz R, Tetik T, Otlu B, Kaya S, Caliskan A. Epidemiologic characterization of nosocomial Acinetobacter baumannii infections in a Turkish university hospital by pulsed-field gel electrophoresis. American Journal of Infection Control. 2009;37(1):56-64.

499. Asadollahi K, Alizadeh E, Akbari M, Taherikalani M, Niakan M, Maleki A, et al. The role of bla(OXA-like carbapenemase) and their insertion sequences (ISS) in the induction of resistance against carbapenem antibiotics among Acinetobacter baumannii isolates in Tehran hospitals. Roumanian archives of microbiology and immunology. 2011;70(4):153-8.

500. Sonbol FI, El-Banna TES, Attia NA. Prevalence of carbapenemases among imipenem resistant Acinetobacter Baumannii isolates in Tanta, Egypt. International Research Journal of Pharmacy. 2019;10(12):77-80.

501. Chusri S, Chongsuvivatwong V, Silpapojakul K, Singkhamanan K, Hortiwakul T, Charernmak B, Doi Y. Clinical characteristics and outcomes of community and hospital-acquired Acinetobacter baumannii bacteremia. Journal of Microbiology Immunology and Infection. 2019;52(5):796-806.

502. Sobouti B, Mirshekar M, Fallah S, Tabaei A, Fallah Mehrabadi J, Darb, i A. Pan drug-resistant Acinetobacter baumannii causing nosocomial infections among burnt children. Medical journal of the Islamic Republic of Iran. 2020;34:24-.

503. Zaniani FR, Moazen J, Anaam M. Detection of Extended-spectrum Beta-lactamases (ESBLs), Carbapenemase, Metallo-β-lactamase Production Bacteria and Antibiotic Susceptibility Pattern in Hospitalized Patients with Ventilator-associated Pneumonia. Jundishapur Journal of Microbiology. 2022;15(9).

504. Alshami HGA, Shaye MA, Bahreini M, Sharifmoghadam MR. Prevalence of Extended-Spectrum beta-Lactamase Genes and Antibiotic Resistance Pattern in Clinical Isolates of Acinetobacter baumannii from Patients Hospitalized in Mashhad, Iran. Jundishapur Journal of Microbiology. 2022;15(3).

505. Jean SS, Teng LJ, Hsueh PR, Ho SW, Luh KT. Antimicrobial susceptibilities among clinical isolates of extended-spectrum cephalosporin-resistant Gram-negative bacteria in a Taiwanese University Hospital. Journal of Antimicrobial Chemotherapy. 2002;49(1):69-76.

506. Turton JF, Kaufmann ME, Warner M, Coelho J, Dijkshoorn L, van der Reijden T, Pitt TL. A prevalent, multiresistant clone of Acinetobacter baumannii in Southeast England. Journal of Hospital Infection. 2004;58(3):170-9.

507. Mahdian S, Sadeghifard N, Pakzad I, Ghanbari F, Soroush S, Azimi L, et al. Acinetobacter baumannii clonal lineages I and II harboring different carbapenem-hydrolyzing-beta-lactamase genes are widespread among hospitalized burn patients in Tehran. Journal of infection and public health. 2015;8(6):533-42.

508. Ramadan RA, Gebriel MG, Kadry HM, Mosallem A. Carbapenem-resistant Acinetobacter baumannii and Pseudomonas aeruginosa: characterization of carbapenemase genes and E-test evaluation of colistin-based combinations. Infection and Drug Resistance. 2018;11:1261-9.

509. Uc-Cachon AH, Gracida-Osorno C, Luna-Chi IG, Jimenez-Guillermo JG, Molina-Salinas GM. High Prevalence of Antimicrobial Resistance Among Gram-Negative Isolated Bacilli in Intensive Care Units at a Tertiary-Care Hospital in Yucatan Mexico. Medicina-Lithuania. 2019;55(9).

510. El-Badawy MF, Abdelwahab SF, Alghamdi SA, Shohayeb MM. Characterization of phenotypic and genotypic traits of carbapenem-resistant Acinetobacter baumannii clinical isolates recovered from a tertiary care hospital in Taif, Saudi Arabia. Infection and Drug Resistance. 2019;12:3113-24.

511. Niu X, Shi X, Li Q. Differences in clinical characteristics of bloodstream infections caused by Escherichia coli and Acinetobacter baumannii. International Journal of Clinical and Experimental Medicine. 2019;12(4):4330-8.

512. Paramita K, Esa T, Rusli B. Detection of blaimp and blaoxa-23-like genes in acinetobacter baumannii isolates at dr. Wahidin sudirohusodo hospital. Indian Journal of Public Health Research and Development. 2020;11(6):1330-5.

513. Al-Sultan AA. Prevalence of High-Risk Antibiotic Resistant Acinetobacter baumannii in the Holy Cities of Makkah and Al-Madinah. Open Microbiology Journal. 2021;15:145-51.

514. Cabral BG, Brasiliense DM, Furlaneto IP, Rodrigues YC, Batista Lima KV. Surgical Site Infection Following Caesarean Section by Acinetobacter Species: A Report from a Hyperendemic Setting in the Brazilian Amazon Region. Microorganisms. 2021;9(4).

515. Peerayeh SN, Karmostaji A. Molecular Identification of Resistance Determinants, Integrons and Genetic Relatedness of Extensively Drug Resistant Acinetobacter baumannii Isolated From Hospitals in Tehran, Iran. Jundishapur Journal of Microbiology. 2015;8(7).

516. Chatterjee S, Datta S, Roy S, Ramanan L, Saha A, Viswanathan R, et al. Carbapenem Resistance in Acinetobacter baumannii and Other Acinetobacter spp. Causing Neonatal Sepsis: Focus on NDM-1 and Its Linkage to ISAba125. Frontiers in Microbiology. 2016;7.

517. Bado I, Papa-Ezdra R, Delgado-Blas JF, Gaudio M, Gutierrez C, Cordeiro NF, et al. Molecular Characterization of Carbapenem-Resistant Acinetobacter baumannii in the Intensive Care Unit of Uruguay's University Hospital Identifies the First rmtC Gene in the Species. Microbial Drug Resistance. 2018;24(7):1012-9.

518. Yeongdon J, Jeong KY, Chulhun C, Go-eun C, Yae HK. Relationship between AdeABC Efflux Pump Genes and Carbapenem in Multidrug-resistant Acinetobacter baumannii. Biomedical Science Letters. 2021;27(2):59-68.

519. Shi N, Kang J, Wang S, Song Y, Yin D, Li X, et al. Bacteriological Profile and Antimicrobial Susceptibility Patterns of Gram-Negative Bloodstream Infection and Risk Factors Associated with Mortality and Drug Resistance: A Retrospective Study from Shanxi, China. Infection and Drug Resistance. 2022;15:3561-78.

520. Fonseca EL, Scheidegger E, Freitas FS, Cipriano R, Vicente ACP. Carbapenem-resistant Acinetobacter baumannii from Brazil: Role of carO alleles expression and bla §ssub§OXA-23§esub§ gene. BMC Microbiology. 2013;13(1).

521. Fan L, Wang Z, Wang Q, Xiong Z, Xu Y, Li D, et al. Increasing rates of Acinetobacter baumannii infection and resistance in an oncology department. Journal of Cancer Research and Therapeutics. 2018;14(1):68-71.

522. Hamza MM, Hadi OM. Detection of qnr A and New Delhi metallo- beta- lactamase-1 (bla NDM-1) in Acinetobacter baumannii isolated from clinical samples in Hillah hospitals. Annals of Tropical Medicine and Public Health. 2020;23(14).

523. Osman M, Halimeh FB, Rafei R, Mallat H, Tom JE, Raad EB, et al. Investigation of an XDR-Acinetobacter baumannii ST2 outbreak in an intensive care unit of a Lebanese tertiary care hospital. Future Microbiology. 2020;15(16):1535-42.

524. Rizvi A, Saeed MU, Nadeem A, Yaqoob A, Rabaan AA, Bakhrebah MA, et al. Evaluation of Bi-Lateral Co-Infections and Antibiotic Resistance Rates among COVID-19 Patients in Lahore, Pakistan. Medicina-Lithuania. 2022;58(7).

525. Mahich S, Angurana SK, Sundaram V, Gautam V. Epidemiology, microbiological profile, and outcome of culture positive sepsis among outborn neonates at a tertiary hospital in Northern India. Journal of Maternal-Fetal & Neonatal Medicine. 2022;35(25):7948-56.

526. Kotsakis SD, Petinaki E, Scopes E, Siatravani E, Miriagou V, Tzelepi E. Laboratory evaluation of Brilliance™ CRE Agar for screening carbapenem-resistant Enterobacteriaceae: Performance on a collection of characterised clinical isolates from Greece. Journal of Global Antimicrobial Resistance. 2013;1(2):85-90.

527. Vu Dinh P, Nadjm B, Nguyen Hoang Anh D, Dao Xuan C, Nguyen Thi Hoang M, Dao Tuyet T, et al. Ventilator-associated respiratory infection in a resource-restricted setting: impact and etiology. Journal of Intensive Care. 2017;5.

528. Jain M, Sharma A, Sen MK, Rani V, ana, Gaind R, Suri JC. Phenotypic and molecular characterization of Acinetobacter baumannii isolates causing lower respiratory infections among ICU patients. Microbial Pathogenesis. 2019;128:75-81.

529. Caldart RV, Fonseca EL, Freitas F, Rocha L, Vicente AC. Acinetobacter baumannii infections in Amazon Region driven by extensively drug resistant international clones, 2016-2018. Memorias Do Instituto Oswaldo Cruz. 2019;114.

530. Xu Q, Hua X, He J, Zhang D, Chen Q, Zhang L, et al. The distribution of mutations and hotspots in transcription regulators of resistance-nodulation-cell division efflux pumps in tigecycline non-susceptible Acinetobacter baumannii in China. International Journal of Medical Microbiology. 2020;310(8).

531. Jaloot AS, Owaid MN. Antibiotic resistance pattern and prevalence of multi-drug and extensive resistant acinetobacter baumannii isolates from clinical specimens after military operations Western Iraq. Gazi Medical Journal. 2021;32(3):381-8.

532. Azad M, Das S, Sarfraz A. RETROSPECTIVE ANALYSIS OF BLOOD STREAM INFECTIONS CAUSED BY ACINETOBACTER SPECIES AND ANTIBIOTIC SUSCEPTIBILITY PATTERN IN A TERTIARY CARE CENTER OF BIHAR. Asian Journal of Microbiology, Biotechnology and Environmental Sciences. 2021;23(3):365-71.

533. Altun HU, Yagci S, Bulut C, Sahin H, Kinikli S, Adiloglu AK, Demiroz AP. Antimicrobial Susceptibilities of Clinical Acinetobacter baumannii Isolates With Different Genotypes. Jundishapur Journal of Microbiology. 2014;7(12).

534. Choe YJ, Lee HJ, Choi EH. Risk Factors for Mortality in Children with Acinetobacter baumannii Bacteremia in South Korea: The Role of Carbapenem Resistance. Microbial Drug Resistance. 2019;25(8):1210-8.

535. Rahbar M, Kabeh-Monnavar M, Vatan KK, Fadaei-Haqi A, Shakerian F. Carbapenem resistance in gram-negative bacilli isolates in an Iranian 1000-bed tertiary hospital. Pakistan Journal of Medical Sciences. 2008;24(4):537-40.

536. Asghar AH, Faidah HS. Frequency and antimicrobial susceptibility of Gram-negative bacteria isolated from 2 hospitals in Makkah, Saudi Arabia. Saudi Medical Journal. 2009;30(8):1017-23.

537. Gilani M, Munir T, Latif M, Gilani M, Rehman S, Ansari M, et al. In Vitro Efficacy of Doripenem against Pseudomonas aeruginosa and Acinetobacter baumannii by E-Test. Jcpsp-Journal of the College of Physicians and Surgeons Pakistan. 2015;25(10):726-9.

538. Vicentini C, Quattrocolo F, D'Ambrosio A, Corcione S, Ricchizzi E, Moro ML, et al. Point prevalence data on antimicrobial usage in Italian acute-care hospitals: Evaluation and comparison of results from two national surveys (2011-2016). Infection Control and Hospital Epidemiology. 2020;41(5):579-84.

539. Nguyen LP, Park CS, Pinto NA, Lee H, Seo HS, Vu TN, et al. In vitro activity of a novel siderophore-cephalosporin LCB10-0200 (GT-1), and LCB10-0200/avibactam, against carbapenem-resistant Escherichia coli, Llebsiella pneumoniae, Acinetobacter baumannii, and pseudomonas aeruginosa strains at a tertiary hospital in Korea. Pharmaceuticals. 2021;14(4).

540. Mohsin S, Bakir WAE, Arsheed M. Determination of the prevalence of blaoxa-like gene and ISAba1 elements among extensive-drug resistant (XDR) Acinetobacter boumannii isolates. Bionatura. 2021;6(4):2284-91.

541. Schuertz KF, Tuon FF, Palmeiro JK, Conte D, Telles JPM, Trevisoli LE, Dalla-Costa LM. Bacteremia and meningitis caused by OXA-23-producing Acinetobacter baumannii - molecular characterization and susceptibility testing for alternative antibiotics. Braz J Microbiol. 2018;49:199-204.

542. Tamburro M, Bagnoli D, Di Tella D, Fanelli I, Sammarco ML, Guerrizio G, Ripabelli G. Carbapenemases and efflux pumps in clinical Acinetobacter baumannii: biomolecular characterization of multi-drug resistant isolates from a hospital in Central Italy. International Journal of Infectious Diseases. 2019;79:44-.

543. Iregui A, ro, Khan Z, man D, Quale J. Activity of Cefiderocol Against Enterobacterales, Pseudomonas aeruginosa, and Acinetobacter baumannii Endemic to Medical Centers in New York City. Microbial Drug Resistance. 2020;26(7):722-6.

544. Iregui A, ro, man D, Quale J. Activity of Omadacycline and Other Tetracyclines Against Contemporary Gram-Negative Pathogens from New York City Hospitals. Microbial Drug Resistance. 2021;27(2):190-5.

545. Shah AA, Ahmad I, Shafique M, Siddique AB, Aslam B, Qamar MU. Antibacterial activity of silver nanoparticles against carbapenem-resistant Acinetobacter baumannii clinical isolates. Pakistan Journal of Pharmaceutical Sciences. 2022;35(1):203-8.

546. Mun SJ, Kim S-H, Kim H-T, Moon C, Wi YM. The epidemiology of bloodstream infection contributing to mortality: the difference between community-acquired, healthcare-associated, and hospital-acquired infections. Bmc Infectious Diseases. 2022;22(1).

547. Park YK, Lee GH, Baek JY, Chung DR, Peck KR, Song J-H, Ko KS. A Single Clone of Acinetobacter baumannii, ST22, Is Responsible for High Antimicrobial Resistance Rates of Acinetobacter Spp. Isolates That Cause Bacteremia and Urinary Tract Infections in Korea. Microbial Drug Resistance. 2010;16(2):143-9.

548. Shin KS, seong-bok H, Kyudong H, 정혜원, 손보라, 신동익, 류동희. Characterization of Acinetobacter baumannii Co-producing Carbapenemases OXA-23 and OXA-66, and armA 16S Ribosomal RNA Methylase at a University Hospital in South Korea. Annals of Clinical Microbiology. 2011;14(2):67-73.

549. Mathlouthi N, Areig Z, Al Bayssari C, Bakour S, El Salabi AA, Ben Gwierif S, et al. Emergence of Carbapenem-Resistant Pseudomonas aeruginosa and Acinetobacter baumannii Clinical Isolates Collected from Some Libyan Hospitals. Microbial Drug Resistance. 2015;21(3):335-41.

550. Singkham-in U, Chatsuwan T. In vitro activities of carbapenems in combination with amikacin, colistin, or fosfomycin against carbapenem-resistant Acinetobacter baumannii clinical isolates. Diagnostic Microbiology and Infectious Disease. 2018;91(2):169-74.

551. Licata F, Quirino A, Pepe D, Matera G, Bianco A, Collaborative G. Antimicrobial Resistance in Pathogens Isolated from Blood Cultures: A Two-Year Multicenter Hospital Surveillance Study in Italy. Antibiotics (Basel). 2020;10(1).

552. El Hafa H, Nayme K, Sbiti M, Timinouni M, Belhaj A. Evaluation of genetic diversity of carbapenem-resistant Acinetobacter baumannii isolates using ERIC-PCR. Gene Reports. 2020;21:100829-Article No.: .

553. Cherukuri B. Demographics and antimicrobial susceptibility patterns of lower respiratory tract infections in intensive care unit of a teaching hospital in South India. Anaesthesia, Pain and Intensive Care. 2021;25(6):757-62.

554. Chmielarczyk A, Higgins PG, Wojkowska-Mach J, Synowiec E, er E, Romaniszyn D, et al. Control of an outbreak of Acinetobacter baumannii infections using vaporized hydrogen peroxide. Journal of Hospital Infection. 2012;81(4):239-45.

555. Dehghani M, Masjedian F, Mirnejad R, Fooladi AAI, Haghighat S. Antimicrobial Susceptibility Patterns and Distribution of bla(kpc) Genes among Acinetobacter baumannii Isolated from Patients at Tehran - Iran Hospitals. Journal of Pure and Applied Microbiology. 2012;6(2):707-12.

556. Moon C, Kwak YG, Kim B-N, Kim ES, Lee C-S. Implications of postneurosurgical meningitis caused by carbapenem-resistant Acinetobacter baumannii. Journal of Infection and Chemotherapy. 2013;19(5):916-9.

557. Mirnejad R, Mostofi S, Masjedian F. Antibiotic resistance and carriage class 1 and 2 integrons in clinical isolates of Acinetobacter baumannii from Tehran, Iran. Asian Pacific Journal of Tropical Biomedicine. 2013;3(2):140-5.

558. Pan Y, Xu Y, Huang Y, Wang Z, Shen J. Surveillance of antibiotic resistance in clinical isolates from the first affiliated Hospital of Anhui Medical University during 2017. Chinese Journal of Infection and Chemotherapy. 2018;18(6):627-33.

559. Giannella M, Bussini L, Pascale R, Bartoletti M, Malagrino M, Pancaldi L, et al. Prognostic Utility of the New Definition of Difficult-to-Treat Resistance Among Patients With Gram-Negative Bloodstream Infections. Open Forum Infectious Diseases. 2019;6(12).

560. Radhi SH, Al-Charrakh AH. Occurrence of MBLs and carbapenemases among MDR and XDR Acinetobacter baumannii isolated from hospitals in Iraq. Indian Journal of Public Health Research and Development. 2019;10(7):668-74.

561. Ali IR, Majeed SH. DISTRIBUTION OF TWO-COMPONENT QS GENE IN ACINETOBACTER BAUMANNII. Biochemical and Cellular Archives. 2021;21(2):4829-32.

562. Wu H-N, Yuan E-Y, Li W-B, Peng M, Zhang Q-Y, Xie K-l. Microbiological and Clinical Characteristics of Bloodstream Infections in General Intensive Care Unit: A Retrospective Study. Frontiers in Medicine. 2022;9.

563. Devian MK, Suranadi IW, Hartawan IGAGU, Aryabiantara IW. Bacterial Patterns and Sensitivity to Antibiotics in Patients Treated with Ventilators at the Intensive Care Unit of Sanglah Hospital Denpasar, Bali, Indonesia. Open Access Macedonian Journal of Medical Sciences. 2022;10:250-4.

564. Sharma S, Banerjee T, Yadav G, Chaurasia RC, ra. Role of early foldscopy (microscopy) of endotracheal tube aspirates in deciding restricted empirical therapy in ventilated patients. Indian Journal of Medical Microbiology. 2022;40(1):96-100.

565. Metan G, Zarakolu P, Otlu B, Tekin I, Aytac H, Bolek EC, et al. Emergence of colistin and carbapenem-resistant Acinetobacter calcoaceticus-Acinetobacter baumannii (CCR-Acb) complex in a neurological intensive care unit followed by successful control of the outbreak. Journal of Infection and Public Health. 2020;13(4):564-70.

566. de Freitas SB, Amaral SC, Ferreira MRA, Roloff BC, Jr CM, Conceicao FR, Hartwig DD. Molecular Characterization of Carbapenem-Resistant Acinetobacter baumannii Associated with Nosocomial Infection in the Pelotas, RS, Brazil. Current Microbiology. 2020;77(10):2724-34.

567. Kaur C, Sharma S. Bacteriological Profile and their Antibiotic Susceptibility Pattern in Bloodstream infections in a tertiary Care Hospital in North india. Journal of Pure and Applied Microbiology. 2022;16(4):2756-63.

568. Slimene K, Ali AA, Mohamed EA, El Salabi A, Suliman FS, Elbadri AA, et al. Isolation of Carbapenem and Colistin Resistant Gram-Negative Bacteria Colonizing Immunocompromised SARS-CoV-2 Patients Admitted to Some Libyan Hospitals. Microbiology spectrum. 2023:e0297222-e.

569. Mugnier PD, Poirel L, Naas T, Nordmann P. Worldwide dissemination of the blaOXA-23 Carbapenemase gene of Acinetobacter baumannii1. Emerging Infectious Diseases. 2010;16(1):35-40.

570. Lin Y-C, Hsia K-C, Chen Y-C, Sheng W-H, Chang S-C, Liao M-H, Li S-Y. Genetic Basis of Multidrug Resistance in Acinetobacter Clinical Isolates in Taiwan. Antimicrobial Agents and Chemotherapy. 2010;54(5):2078-84.

571. Lee S-C, Huang S-S, See L-C, Tsai M-H, Shieh W-B. In vitro activities of nine current antibiotics against culprit bacteria in nosocomial infections in an institution in Northern Taiwan. Chang Gung medical journal. 2011;34(6):580-9.

572. Terzi H-A, Atasoy A-R, Aykan S-B, Karakece E, Asik G, Ciftci I-H. Association of doripenem resistance with OXA-type carbapenemases in Acinetobacter baumannii isolates. Saudi Medical Journal. 2016;37(1):43-7.

573. Tafreshi N, Babaeekhou L, Ghane M. Antibiotic resistance pattern of Acinetobacter baumannii from burns patients: increase in prevalence of blaOXA-24-like and blaOXA-58-like genes. Iranian journal of microbiology. 2019;11(6):502-9.

574. ic-Pavlovic D, Zah-Bogovic T, Zizek M, Bielen L, Bratic V, Hrabac P, et al. Gram-negative bacteria as causative agents of ventilator-associated pneumonia and their respective resistance mechanisms. Journal of Chemotherapy. 2020;32(7):344-58.

575. Raheem HQ, Al-Hasnawy HH. MOLECULAR INVESTIGATION OF ANTIBIOTIC RESISTANCE GENES IN EXTENSIVE DRUG RESISTANT (XDR) ACINETOBACTER BAUMANNII ISOLATED FROM CLINICAL SPECIMENS IN BABYLON PROVINCE, IRAQ. Biochemical and Cellular Archives. 2020;20:4349-56.

576. Kadom SM, Abid IN. Detection of blaOXA-51-like and blaVIM carbapenemase genes in acinetobacter baumannii isolated from burn patients. International Journal of Pharmaceutical Research. 2020;12(2):1812-9.

577. Zafer MM, Hussein AFA, Al-Agamy MH, Radwan HH, Hamed SM. Genomic Characterization of Extensively Drug-Resistant NDM-Producing Acinetobacter baumannii Clinical Isolates With the Emergence of Novel bla(ADC-257). Frontiers in Microbiology. 2021;12.

578. Lu L, Xu C, Tang Y, Wang L, Cheng Q, Chen X, et al. The Threat of Carbapenem-Resistant Gram-Negative Bacteria in Patients with Hematological Malignancies: Unignorable Respiratory Non-Fermentative Bacteria-Derived Bloodstream Infections. Infection and Drug Resistance. 2022;15:2901-14.

579. Legese MH, Asrat D, Swedberg G, Hasan B, Mekasha A, Getahun T, et al. Sepsis: emerging pathogens and antimicrobial resistance in Ethiopian referral hospitals. Antimicrobial Resistance and Infection Control. 2022;11(1).

580. Marti S, Sanchez-Cespedes J, Alba V, Vila J. In vitro activity of doripenem against Acinetobacter baumannii clinical isolates. International Journal of Antimicrobial Agents. 2009;33(2):181-2.

581. Virginia Villegas M, Felipe Briceno D, Jamil Ruiz S, Furtado GH, Nicolau DP. Assessing the pharmacodynamic profile of intravenous antibiotics against prevalent Gram-negative organisms collected in Colombia. Brazilian Journal of Infectious Diseases. 2011;15(5):413-9.

582. Rakhi NN, Ul Alam ASMR, Sultana M, Rahaman MM, Hossain MA. Diversity of carbapenemases in clinical isolates: The emergence of bla(VIM-5) in Bangladesh. Journal of Infection and Chemotherapy. 2019;25(6):444-51.

583. Carcione D, Siracusa C, Sulejmani A, Migliavacca R, Mercato A, ra, et al. In Vitro Antimicrobial Activity of the Siderophore Cephalosporin Cefiderocol against Acinetobacter baumannii Strains Recovered from Clinical Samples. Antibiotics-Basel. 2021;10(11).

584. Zahra N, Zeshan B, Qadri MMA, Ishaq M, Afzal M, Ahmed N. Phenotypic and Genotypic Evaluation of Antibiotic Resistance of Acinetobacter baumannii Bacteria Isolated from Surgical ICU Patients in Pakistan. Jundishapur Journal of Microbiology. 2021;14(4).

585. Di Carlo P, Serra N, Lo Sauro S, Carelli VM, Giarratana M, Signorello JC, et al. Epidemiology and Pattern of Resistance of Gram-Negative Bacteria Isolated from Blood Samples in Hospitalized Patients: A Single Center Retrospective Analysis from Southern Italy. Antibiotics-Basel. 2021;10(11).

586. Shali AAK, Jalal PJ, Arif S, K. Dissemination and Genetic Relatedness of Multidrug-Resistant and Extensively Drug-Resistant Acinetobacter baumannii Isolates from a Burn Hospital in Iraq. Canadian Journal of Infectious Diseases & Medical Microbiology. 2022;2022.

587. Aydemir O, Aydemir Y, Sahin EO, Sahin F, Koroglu M, Erdem AF. Secondary bacterial infections in patients with coronavirus disease 2019-associated pneumonia. Revista Da Associacao Medica Brasileira. 2022;68(2):142-6.

588. Jean S-S, Lee Y-L, Liu P-Y, Lu M-C, Ko W-C, Hsueh P-R. Multicenter surveillance of antimicrobial susceptibilities and resistance mechanisms among Enterobacterales species and non-fermenting Gram-negative bacteria from different infection sources in Taiwan from 2016 to 2018. Journal of Microbiology Immunology and Infection. 2022;55(3):463-73.

589. Fadda G, Spanu T, Ardito F, Taddei C, Santangelo R, Siddu A, et al. Antimicrobial resistance among non-fermentative Gram-negative bacilli isolated from the respiratory tracts of Italian inpatients: a 3-year surveillance study by the Italian Epidemiological Survey. International Journal of Antimicrobial Agents. 2004;23(3):254-61.

590. Karunasagar A, Maiti B, Shekar M, Shenoy SM, Karunasagar I. Prevalence of OXA-type carbapenemase genes and genetic heterogeneity in clinical isolates of Acinetobacter spp. from Mangalore, India. Microbiology and Immunology. 2011;55(4):239-46.

591. Zhong L, Men TY, Li H, Peng ZH, Gu Y, Ding X, et al. Multidrug-resistant gram-negative bacterial infections after liver transplantation - Spectrum and risk factors. Journal of Infection. 2012;64(3):299-310.

592. Martinez P, Mattar S. IMIPENEM-RESISTANT ACINETOBACTER BAUMANNII CARRYING THE ISABA1-BLA(OXA-23,) (51) AND ISABA1-BLA(ADC-7) GENES IN MONTERIA, COLOMBIA. Brazilian Journal of Microbiology. 2012;43(4):1274-80.

593. Gao F, Ye Q, Wan Q, Liu S, Zhou J, ang. Distribution and resistance of pathogens in liver transplant recipients with Acinetobacter baumannii infection (Publication with Expression of Concern. See vol. 16, pg. 347, 2020). Therapeutics and Clinical Risk Management. 2015;11:501-5.

594. Van PH, Binh PT, Ngan LLB, Huong PT. The resistance of the gram-negative rods isolated from clinical cases in vietnam to doripenem. International Journal of Antimicrobial Agents. 2017;50:S104.

595. Olowo-okere A, Ibrahim YKE, Olayinka BO, Ehinmidu JO, Mohammed Y, Nabti LZ, et al. Phenotypic and genotypic characterization of clinical carbapenem-resistant Enterobacteriaceae isolates from Sokoto, northwest Nigeria. New Microbes and New Infections. 2020;37:100727-Article No.: .

596. Ribeiro EA, Gales AC, Oliveira APS, Coelho DD, Oliveira RA, Pfrimer IAH, Carmo Filho JRD. Molecular epidemiology and drug resistance of Acinetobacter baumannii isolated from a regional hospital in the Brazilian Amazon region. Rev Soc Bras Med Trop. 2020;54:e20200087.

597. Bori M, Gohatre A, Kumar A. A Hospital-Based Observational Assessment of the Demographics and Antimicrobial Susceptibility Patterns of Lower Respiratory Tract Infections in Intensive Care Unit. International Journal of Pharmaceutical and Clinical Research. 2023;15(3):544-51.

598. Khalifa R, Ismail B, Al-Jahdali H, Al Ghamdi H, Joharjy H, Eibani K, et al. Secondary multidrug resistant bacterial pneumonia among adult COVID-19 patients: A molecular study. Microbes and Infectious Diseases. 2023;4(1):11-26.

599. Bogaerts P, Naas T, Wybo I, Bauraing C, Soetens O, Pierard D, et al. Outbreak of infection by carbapenem-resistant Acinetobacter baumannii producing the carbapenemase OXA-58 in Belgium. Journal of Clinical Microbiology. 2006;44(11):4189-92.

600. Matsui M, Suzuki M, Suzuki M, Yatsuyanagi J, Watahiki M, Hiraki Y, et al. Distribution and Molecular Characterization of Acinetobacter baumannii International Clone II Lineage in Japan. Antimicrobial Agents and Chemotherapy. 2018;62(2).

601. Ravi NS, An, an S, Vijayakumar S, Gopi R, Lopes BS, Veeraraghavan B. The potential of different molecular biology methods in tracking clones of Acinetobacter baumannii in an ICU setting. Journal of Medical Microbiology. 2018;67(9):1340-7.

602. Duszynska W, Litwin A, Rojek S, Szczesny A, er, Ciasullo A, Gozdzik W. Analysis of Acinetobacter baumannii hospital infections in patients treated at the intensive care unit of the University Hospital, Wroclaw, Poland: a 6-year, single-center, retrospective study. Infection and Drug Resistance. 2018;11:629-35.

603. Ayar G, Yakut HI, Atmaca YM, Emeksiz S, Parlakay AO. Evaluation of Healthcare-Acquired Infection Rates in a Pediatric Intensive Care Unit in Turkey. Journal of Pediatric Infectious Diseases. 2019;14(5):235-41.

604. Trapaidze N, Farlow J, Latif N, Nozadze M, Aptsiauri T, Mitaishvili N, et al. Genetic determinants supporting the multi-drug resistance of Acinetobacter spp. in Georgia. International Journal of Infectious Diseases. 2019;79:52.

605. y A, Almaeen AH. Pathogenic spectrum of blood stream infections and resistance pattern in Gram-negative bacteria from Aljouf region of Saudi Arabia. Plos One. 2020;15(6).

606. Ingti B, Upadhyay S, Hazarika M, Khyriem AB, Paul D, Bhattacharya P, et al. Distribution of carbapenem resistant Acinetobacter baumannii with bla ADC-30 and induction of ADC-30 in response to beta-lactam antibiotics. Research in Microbiology. 2020;171(3):128-33.

607. Ghimire U, el R, Neupane M, Shrestha S, Sudeep KC, Khanal S, Joshi DR. Biofilm Formation and blaOXA Genes Detection Among Acinetobacter baumannii from Clinical Isolates in a Tertiary Care Kirtipur Hospital, Nepal. Progress in Microbes and Molecular Biology. 2021;4(1).

608. San T, Aung MS, San N, Aung MMZ, Mon WLY, Thazin TE, Kobayashi N. Bacterial Species and Antimicrobial Resistance of Clinical Isolates from Pediatric Patients in Yangon, Myanmar, 2020. Infectious Disease Reports. 2022;14(1):26-32.

609. Arbune M, Gurau G, Niculet E, Iancu AV, Lupasteanu G, Fotea S, et al. Prevalence of Antibiotic Resistance of ESKAPE Pathogens Over Five Years in an Infectious Diseases Hospital from South-East of Romania. Infection and Drug Resistance. 2021;14:2369-78.

610. Aedh AII, Al-Swedan AD, Mohammed AA, Alwadai BM, Alyami AY, Alsaaed EA, et al. Occurrence of Multidrug-Resistant Strains of Acinetobacter spp.: An Emerging Threat for Nosocomial-Borne Infection in Najran Region, KSA. Tropical Medicine and Infectious Disease. 2023;8(2).

611. Alotaibi BS, Tantry BA, Farhana A, Alammar MA, Shah NN, Mohammed AH, et al. Resistance Pattern in Mostly Gram-negative Bacteria Causing Urinary Tract Infections. Infectious Disorders - Drug Targets. 2023;23(2):56-64.

612. Cheng NC, Hsueh PR, Liu YC, Shyr JM, Huang WK, Teng LJ, Liu CY. In vitro activities of tigecycline, ertapenem, isepamicin, and other antimicrobial agents against clinically isolated organisms in Taiwan. Microbial Drug Resistance. 2005;11(4):330-41.

613. Ling TKW, Ying CM, Lee CC, Liu ZK. Comparison of antimicrobial resistance of Acinetobacter baumannii clinical isolates from Shanghai and Hong kong. Medical Principles and Practice. 2005;14(5):338-41.

614. Mostofi S, Mirnejad R, Masjedian F. Multi-drug resistance in Acinetobacter baumannii strains isolated from the clinical specimens of three hospitals in Tehran-Iran. African Journal of Microbiology Research. 2011;5(26):4467-70.

615. Zhong Q, Xu W, Wu Y, Xu H. Clonal Spread of Carbapenem Non-susceptible Acinetobacter baumannii in an Intensive Care Unit in a Teaching Hospital in China. Annals of Laboratory Medicine. 2012;32(6):413-9.

616. Todorova B, Velinov T, Ivanov I, Dobreva E, Kantardjiev T. First detection of OXA-24 carbapenemase-producing Acinetobacter baumannii isolates in Bulgaria. World Journal of Microbiology & Biotechnology. 2014;30(4):1427-30.

617. Sjol, er I, Hansen F, Elmanama A, Khayyat R, Abu-Zant A, et al. Detection of NDM-2-producing Acinetobacter baumannii and VIM-producing Pseudomonas aeruginosa in Palestine. Journal of Global Antimicrobial Resistance. 2014;2(2):93-7.

618. Ahmed SS, Dinc G, Rossella B, Alp E, Ulu-Kilic A, Melchers WJG, et al. Molecular Characterization of Carbapenem Resistant Acinetobacter baumannii and Investigation of Genetic Diversity Between Local and International Clones. Journal of Pure and Applied Microbiology. 2016;10(3):1675-82.

619. Abesamis GMM, Cruz JJV. Bacteriologic Profile of Burn Wounds at a Tertiary Government Hospital in the Philippines-UP-PGH ATR Burn Center. Journal of Burn Care & Research. 2019;40(5):658-68.

620. Kabrah A. Extended-Spectrum Beta-Lactamase and Carbapenem-Resistant Gram-Negative Pathogens in Makkah, Saudi Arabia. Ethiopian journal of health sciences. 2022;32(6):1221-30.

621. Balazs B, Toth Z, Nagy JB, Majoros L, Toth A, Kardos G. Faecal Carriage of Carbapenem-Resistant Acinetobacter baumannii: Comparison to Clinical Isolates from the Same Period (2017-2019). Pathogens. 2022;11(9).

622. Liu D, Niu J, Chen G, Xu L. Treatment of Carbapenem-Resistant Multidrug-Resistant Gram-Negative Bacilli with Intracerebroventricular Injection of Polymyxin B A Retrospective study. Infection and Drug Resistance. 2022;15:7653-66.

623. Lavrinenko A, Kolesnichenko S, Kadyrova I, Turmukhambetova A, Akhmaltdinova L, Klyuyev D. Bacterial Co-Infections and Antimicrobial Resistance in Patients Hospitalized with Suspected or Confirmed COVID-19 Pneumonia in Kazakhstan. Pathogens. 2023;12(3).

624. Meng Q, Li W, Jiang H, Yan H, Wang H, Ye B, et al. Comparison of the Distribution and Changes in the Antibiotic Resistance of Clinical Bacterial Isolates from the Lower Respiratory Tract of Children in Shenzhen Before the Epidemic, During the Epidemic, and During the Period of Normalized Prevention and Control of COVID-19. Infectious Diseases and Therapy. 2023;12(2):563-75.

625. Shareek PS, Sureshkumar D, Ramgopalakrishnan, Ramasubramanian V, Abdul Ghafur K, Thirunarayanan MA. Antibiotic sensitivity pattern of Blood isolates of Acinetobacter species in a tertiary Care Hospital: A retrospective analysis. American Journal of Infectious Diseases. 2012;8(1):65-9.

626. Hashmi MA, Lodhi MA, Toor KM, Tahir A, Khan HS, Aziz R. Emerging Antimicrobial Resistance in Neonatal Sepsis. Jcpsp-Journal of the College of Physicians and Surgeons Pakistan. 2020;30(12):1312-5.

627. Talpur MTH, Shabir KU, Shabir KU, Katbar MT, Yaqoob U, Kashif S. Antibiotic susceptibility pattern in an intensive care unit of a tertiary care hospital of Pakistan. Rawal Medical Journal. 2020;45(1):17-21.

628. Mohamed HA, Gad GFM, Mohamed MF, Ahmed HH, Elfarash AE, Fahmy NF. Genotyping of carbapenem resistant Acinetobacter baumannii isolated from Egyptian patients. Novel Research in Microbiology Journal. 2022;6(6):1801-20.

629. Petersen K, Cannegieter SC, van der Reijden TJ, Van Strijen B, You DM, Babel BS, et al. Diversity and Clinical Impact of Acinetobacter baumannii Colonization and Infection at a Military Medical Center. Journal of Clinical Microbiology. 2011;49(1):159-66.

630. Wang X, Zhang L, Sun A, Yang X, Sang W, Jiang Y, et al. Acinetobacter baumannii bacteraemia in patients with haematological malignancy: a multicentre retrospective study from the Infection Working Party of Jiangsu Society of Hematology. European Journal of Clinical Microbiology & Infectious Diseases. 2017;36(7):1073-81.

631. Chen Y, Li F, Zhu M, Liu L, Luo Y. Outcome and factors of patients with nosocomial meningitis by multi-drug-resistant Gram-negative bacteria in a tertiary hospital in China: a retrospective study. British Journal of Neurosurgery. 2020;34(3):324-8.

632. Sewunet T, Asrat D, Woldeamanuel Y, Aseffa A, Giske CG. Molecular epidemiology and antimicrobial susceptibility of Pseudomonas spp. and Acinetobacter spp. from clinical samples at Jimma medical center, Ethiopia. Frontiers in Microbiology. 2022;13.

633. Chakraborty B, Banerjee D, Chakraborty B. Acinetobacter baumannii: no more a choosy intruder? Indian journal of medical sciences. 2011;65(8):344-8.

634. Sacha P, Wieczorek P, Ojdana D, Czaban S, Klosowska W, Jurczak A, Tryniszewska E. Susceptibility, phenotypes of resistance, and extended-spectrum beta-lactamases in Acinetobacter baumannii strains. Folia Histochemica Et Cytobiologica. 2012;50(1):46-51.

635. Nishida S, Ono Y. Comparative analysis of the pathogenicity between multidrug-resistant Acinetobacter baumannii clinical isolates: isolation of highly pathogenic multidrug-resistant A. baumannii and experimental therapeutics with fourth-generation cephalosporin cefozopran. Infection and Drug Resistance. 2018;11:1715-22.

636. Wong MH-y, Chan BK-w, Chan EW-c, Chen S. Over-Expression of ISAba1-Linked Intrinsic and Exogenously Acquired OXA Type Carbapenem-Hydrolyzing-Class D-ss-Lactamase-Encoding Genes Is Key Mechanism Underlying Carbapenem Resistance in Acinetobacter baumannii. Frontiers in Microbiology. 2019;10.

637. Chang J-b, Chen Y, Wang H, Ma X, Zhang X, Wu H, et al. Combined Strategy for Post-Operative Patients with Central Nervous System Infections Caused by Extensively Drug-Resistant/Pan-Drug-Resistant Acinetobacter baumannii: A Retrospective Study. Surgical Infections. 2020;21(10):853-8.

638. Shabban M, Fahim NAE, Montasser K, El Magd NMA. Resistance to Colistin Mediated by mcr-1 among Multidrug Resistant Gram Negative Pathogens at a Tertiary Care Hospital, Egypt. Journal of Pure and Applied Microbiology. 2020;14(2):1125-32.

639. Al Meani SAL, Ahmed MM, Abdulkareem AH. Synergistic effect between zingiber officinale volatile oil and meropenem against acinetobacter baumannii producing-carbapenemase isolated from neurosurgery in Iraq. Systematic Reviews in Pharmacy. 2020;11(9):920-5.

640. El-Kazzaz W, Metwally L, Yahia R, Al-Harbi N, El-Taher A, Hetta HF. Antibiogram, Prevalence of OXA Carbapenemase Encoding Genes, and RAPD-Genotyping of Multidrug-ResistantAcinetobacter baumanniiIncriminated in Hidden Community-Acquired Infections. Antibiotics-Basel. 2020;9(9).

641. Li Z-J, Zhang D-F, Zhang W-H. Analysis of Nosocomial Infection and Risk Factors in Patients with ECMO Treatment. Infection and Drug Resistance. 2021;14:2403-10.

642. Mohamed AH, Omar NMS, Osman MM, Mohamud HA, Eraslan A, Gur M. Antimicrobial Resistance and Predisposing Factors Associated with Catheter-Associated UTI Caused by Uropathogens Exhibiting Multidrug-Resistant Patterns: A 3-Year Retrospective Study at a Tertiary Hospital in Mogadishu, Somalia. Tropical Medicine and Infectious Disease. 2022;7(3).

643. Odih EE, Oaikhena AO, Underwood A, Hounmanou YMG, Oduyebo OO, Fadeyi A, et al. High genetic diversity and blaNDM-1 prevalence among Acinetobacter baumannii in Nigerian hospitals. 2023.

644. Paj, O, Rezaee MA, Nahaei MR, Mahdian R, Aghazadeh M, et al. Study of the carbapenem resistance mechanisms in clinical isolates of Acinetobacter baumannii: Comparison of burn and non-burn strains. Burns. 2013;39(7):1414-9.

645. Sam JE, Lim CL, Sharda P, Wahab NA. The Organisms and Factors Affecting Outcomes of External Ventricular Drainage Catheter-Related Ventriculitis: A Penang Experience. Asian journal of neurosurgery. 2018;13(2):250-7.

646. Yang L, Wu C-m, Li Q-r, Wang Y-m, Ji G, Shan B. Analysis of species distribution and drug resistance of 2,073 strains of bacteria isolated from burn wounds. Zhongguo Kangshengsu Zazhi. 2018;43(5):577-82.

647. Tehrani S, Saffarfar V, Hashemi A, Abolghasemi S. A Survey of Genotype and Resistance Patterns of Ventilator-Associated Pneumonia Organisms in ICU Patients. Tanaffos. 2019;18(3):215-22.

648. Pal A, Tripathi A. 4-Chloromercuribenzoic acid enhances carbapenem sensitivity among pathogenic Gram negative bacteria by altering bla(VIM), adeB and ompC expression. Journal of Infection and Public Health. 2020;13(5):806-14.

649. El-Kattan N, Allam KAM. The antibacterial activity of nano-encapsulated basil and cinnamon essential oils against certain multidrug-resistant bacteria recovered from infected wounds. Novel Research in Microbiology Journal. 2021;5(6):1447-62.

650. Zhang J, Diao S, Liu Y, Wang H, Liu Y, Zhu S, et al. The combination effect of meropenem/sulbactam/polymyxin-B on the pharmacodynamic parameters for mutant selection windows against carbapenem-resistant Acinetobacter baumannii. Frontiers in Microbiology. 2022;13.

651. Ioannou P, Maraki S, Koumaki D, Manios GAA, Koumaki V, Kassotakis D, et al. A Six-Year Retrospective Study of Microbiological Characteristics and Antimicrobial Resistance in Specimens from a Tertiary Hospital's Surgical Ward. Antibiotics-Basel. 2023;12(3).

652. Giamarellos-Bourboulis EJ, Xirouchaki E, Giamarellou H. Interactions of colistin and rifampin on multidrug-resistant Acinetobacter baumannii. Diagnostic Microbiology and Infectious Disease. 2001;40(3):117-20.

653. Goel N, Chaudhary U, Aggarwal R, Bala K. Antibiotic sensitivity pattern of gram negative bacilli isolated from the lower respiratory tract of ventilated patients in the Intensive care unit. Indian journal of critical care medicine : peer-reviewed, official publication of Indian Society of Critical Care Medicine. 2009;13(3):148-51.
[truncated: 35,193 more chars]
